# Preparation of 3-Substituted Isoindolin-1-one, Cinnoline, and 1,2,4-[*e*]-Benzotriazine Derivatives

**DOI:** 10.1021/acsomega.2c03045

**Published:** 2022-07-20

**Authors:** Fatat B. El Dhaibi, Ali Youssef, James C. Fettinger, Mark J. Kurth, Makhluf J. Haddadin

**Affiliations:** † Department of Chemistry, American University of Beirut, Riad El Solh, 1107 2020Beirut, Lebanon; ‡ Department of Chemistry, University of California, One Shields Avenue, Davis, California95616, United States

## Abstract

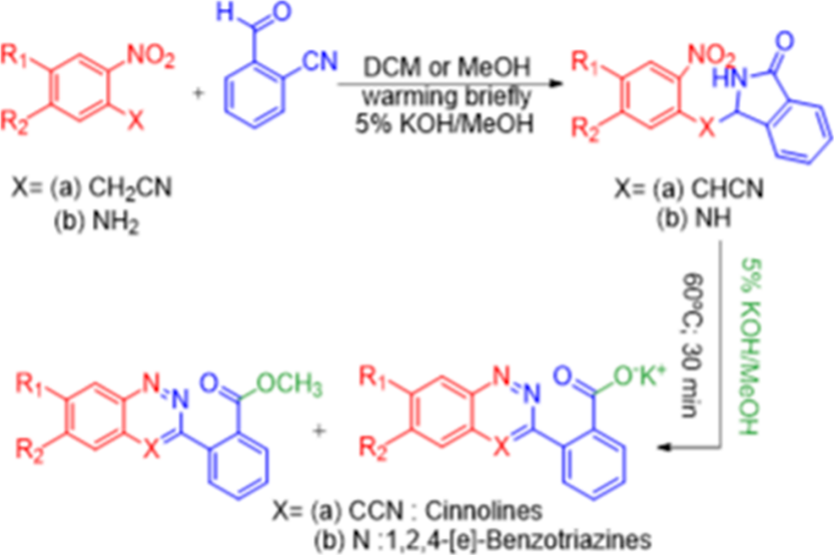

Herein, we report a new approach to synthesize a series of 1,2,4-[*e*]-benzotriazine and cinnoline derivatives from 3-substituted isoindolin-1-one. All the reported products are obtained through an economical two-step synthetic procedure resulting in fair-to-high yields. Cinnolines (a) and 1,2,4-[*e*]-benzotriazines (b) result from an intramolecular cyclization of the corresponding 3-substituted isoindolin-1-ones, which, in turn, are prepared by an addition reaction from 2-cyanobenzaldehyde and 2-(2-nitrophenyl) acetonitrile (a) or 2-nitroaniline derivatives (b). A proposed mechanism for this transformation is presented.

## Introduction

**Figure 1:**
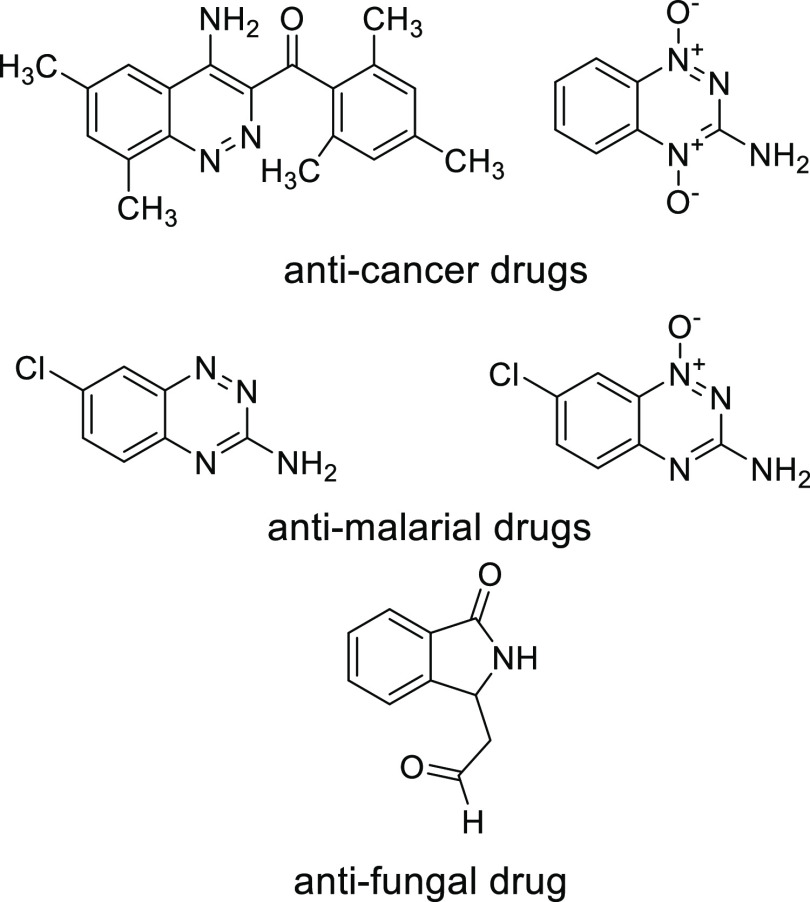
Cinnoline, 1,2,4-[*e*]-benzotriazine and isoindolinone drugs.

Synthetic heterocyclic chemistry has made notable progress in the last few decades.^1−4^ Isoindolinones, cinnolines, and 1,2,4-benzotriazines represent important classes of nitrogen-containing compounds.^5^ In fact, it is reported that seven of the top ten selling drugs in the world are nitrogen-containing heterocycles.^6^ Consequently, these heterocyclic compounds have received considerable attention in organic chemistry ranging from their methods of preparation to studies of their physical, chemical, and biological properties.^1,2^ The growing interest in these N-containing compounds is the result of their wide ranging biological activities such as antimalarial,^7,8^ antibacterial,^9,10^ antiviral, antifungal,^11^ anthelmintic, and anticancer properties for application in pharmaceutical fields (Figure 1).^12−14^

**Figure 2:**
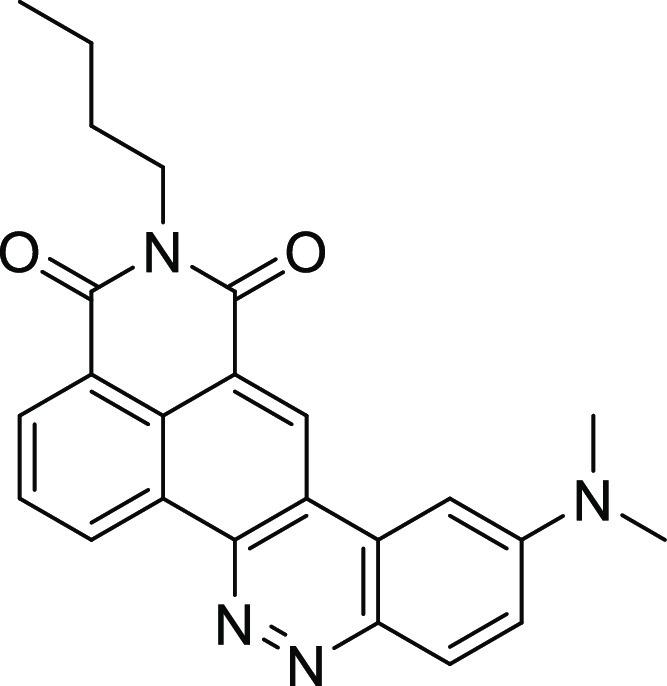
CinNapht dye.

In addition, some analogues of these heterocycles have demonstrated electro-optical activities, and others have been used as dyes (Figure 2).^15^

**Scheme 1:**
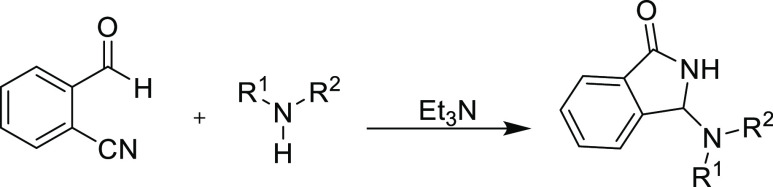
Sato et al.’s Synthesis of 3-N Substituted Isoindolin-1-ones

**Scheme 2:**
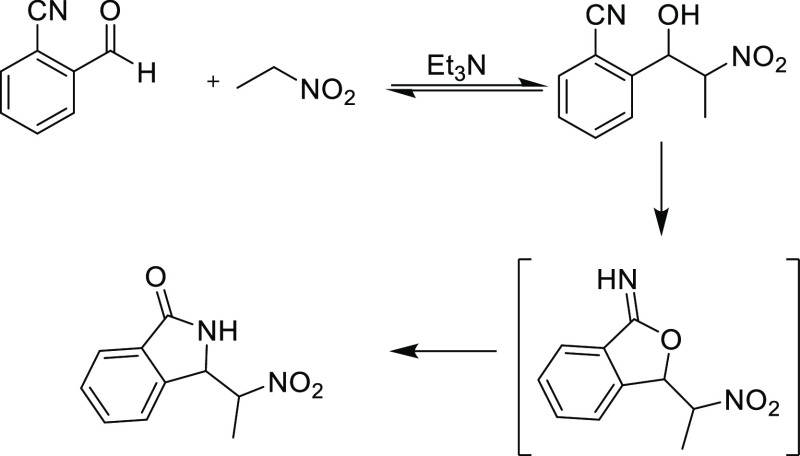
Angelin et al.’s Mechanism-Based Synthesis of 3-Nitrosubstituted Isoindolinones

**Scheme 3:**
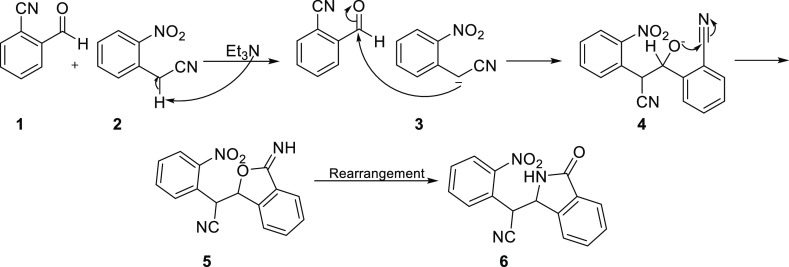
Mechanism for the Synthesis of 2-(2-Nitrophenyl)-2-(3-oxoisoindolin-1-yl)acetonitrile (**6**)

**Scheme 4:**
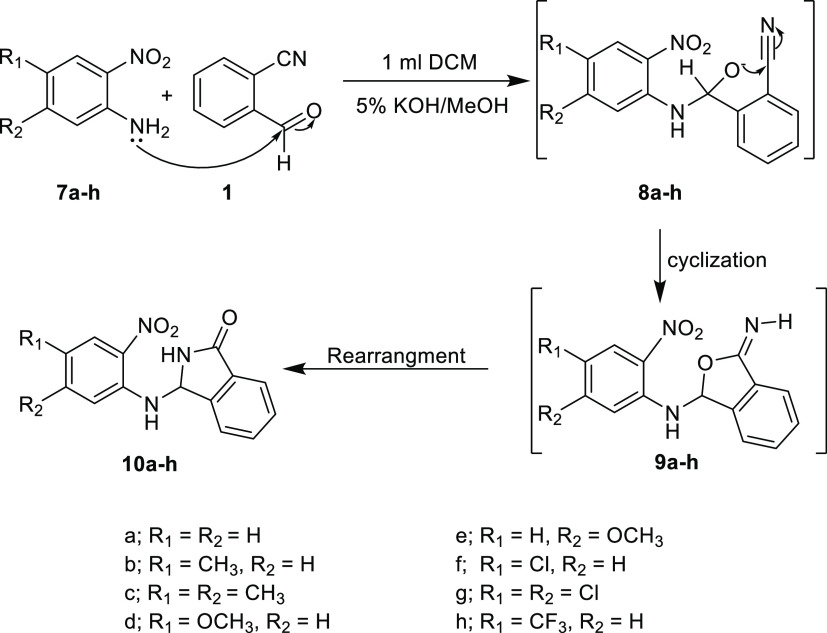
Sato Mechanism Applied to the Synthesis of 3-Aminoisoindolin-1-ones **10a–h**

Taking into consideration all these previous applications, 1,2,4-[*e*]-benzotriazine and cinnoline heterocycles have been synthesized through various multistep reactions over the past several years.^1^ Given their importance, we have developed a simple synthetic method based on reactions and mechanisms reported by Sato et al. (Scheme 1)^16^ and Angelin et al. (Scheme 2),^17,18^ where 2-cyanobenzaldehyde **(1)**, 2-(2-nitrophenyl) acetonitrile (**2**; see Scheme 3), or 2-nitroaniline substituents (**7a–h**; see Scheme 4) are employed as starting materials.

We succeeded in synthesizing a series of substituted isoindolin-1-ones as well as their corresponding novel cinnolines and 1,2,4-[*e*]-benzotriazines. In this paper, we present the synthesis of the latter compounds through a two-step reaction, which is economical and produces good yield.

## Results and Discussion

The synthesis of novel 2-(2-nitrophenyl)-2-(3-oxoisoindolin-1-yl)acetonitrile **(6)** was accomplished through a nucleophilic addition reaction **(1 + 3 → 4)**, followed by a cyclization **(4 → 5)** and subsequent rearrangement **(5 → 6)** process between 2-cyanobenzaldehyde **(1)** and 2-(2-nitrophenyl) acetonitrile **(2)**. The mechanism for this one-pot process is illustrated in Scheme 3.

^1^H NMR, ^13^C NMR, and ^13^C NMR DEPT 135 spectra were consistent with the structure of **6**. Similar to the work reported by Angelin et al., triethylamine was proved most desirable for the reaction.^18^ It is important to abstract the proton at the α-position to the nitro group or between the two withdrawing groups as described in the literature.^17,18^ In our case, Et_3_N abstracted α-H to the nitrile group and generated a nucleophilic specie in the medium. In fact, replacing Et_3_N with 5% KOH in methanol led to several undesired side products. In addition, the amount of solvent used, methanol in this reaction, was an important factor affecting the product yield; it should be minimized in order to precipitate isoindolin-1-one **6** as it forms. Formation of the isoinolin-1-one carbonyl group was shown by a peak at 1703 cm^–1^ in the IR spectra, compared to that of the 2-cyanobenzaldehyde carbonyl group at 1693 cm^–1^. This isoindolin-1-one **(6)** was isolated as a pure white powder, which was sensitive to light turning the material dark brown. This observation might be explained by the reaction of the nitro group oxygen with the benzylic proton of the cyano group. This reaction also applies to 2-nitrobenzaldehyde yielding 2-nitrosobenzoic acid.^19^

The synthesis of 3-aminoisoindolin-1-one derivatives **10a–h** (Scheme 4) has been achieved following the same procedure as described for product **6**: specifically, a nucleophilic addition reaction between the aldehyde function of the 2-cyanobenzaldehyde **(1)** and the amine function of the 2-nitroaniline derivatives **(7a–h)**, followed by cyclization and rearrangement.

In keeping with the mechanism reported by the Sato et al. group in 1984,^16^ the aniline nitrogen lone pair attacks the carbonyl, and the resulting alkoxide anion then attacks the cyano group to form the cyclic intermediate products **9a–h**. A simple subsequent rearrangement occurs to give the lactam isoindolin-1-ones **10a–h**.

In contrast to the reaction described in Scheme 3, nitrogen base; triethylamine Et_3_N in our case blocked the reaction and stopped the progress of the isoindolinone formation. Instead, a few drops of the strong base methanolic KOH (5%) initiated the formation of the products **10a–h**. Starting from a 1:1.2 mmol equivalent of nitroaniline derivatives, 0.4 mL of 2-cyanobenzaldehyde was sufficient for the isolation of the product. Yet, increasing the volume of the base led to some undesired side reactions that decreased the yield of our isoindolinone intermediates. In addition, the solvent nature was found to have an effect on product formation: when using methanol, ethyl acetate, chloroform, and even dimethylformamide, the yields were very low. Fortunately, adding a small amount of dichloromethane (1 mL) resulted in maximum isolation of the desired 3-substituted isoindolin-1-ones **10a–h** as yellow pastes, which were subsequently filtrated and washed with cold methanol. The isolated % yield of product **10c** using various solvents are shown in Table 1.

**Table 1:** Isolated % Yields of **10c** in Different Solvents

solvent	DCM	EtOAc	MeOH	CHCl_3_	DMF
% yield of **10c**	76	39	36	42	65

**Scheme 5:**
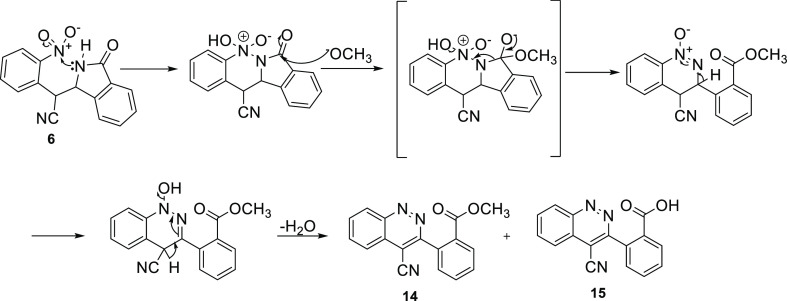
Synthesis of Cinnolines **14** and **15** through the Formation of Isoindolinone **6**

**Scheme 6:**
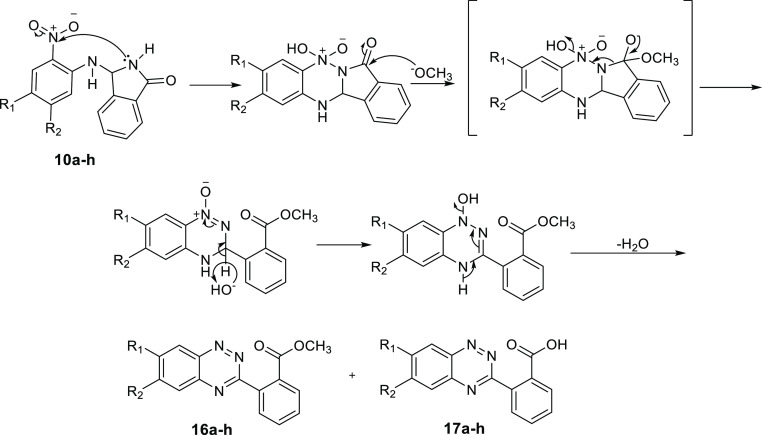
Synthesis of 1,2,4-[*e*]-Benzotriazines **16a–h** and 1**7a–h** from 3-Substituted Isoindolinones **10a–h**

Finally, upon heating in 5% methanolic KOH, compound **6** gave the corresponding cinnolines **14** and **15**, and compounds **10a–h** produced the 1,2,4-[*e*]-benzotriazines **16a–h** and **17a–h.** The postulated mechanisms for the formation of **14–17** are described in Schemes 5 and 6. All structures were supported by full spectral data characterization (^1^H NMR, ^13^C NMR, ^13^C NMR DEPT 135, IR, and HR-MS) as well as melting points.

**Figure 3:**
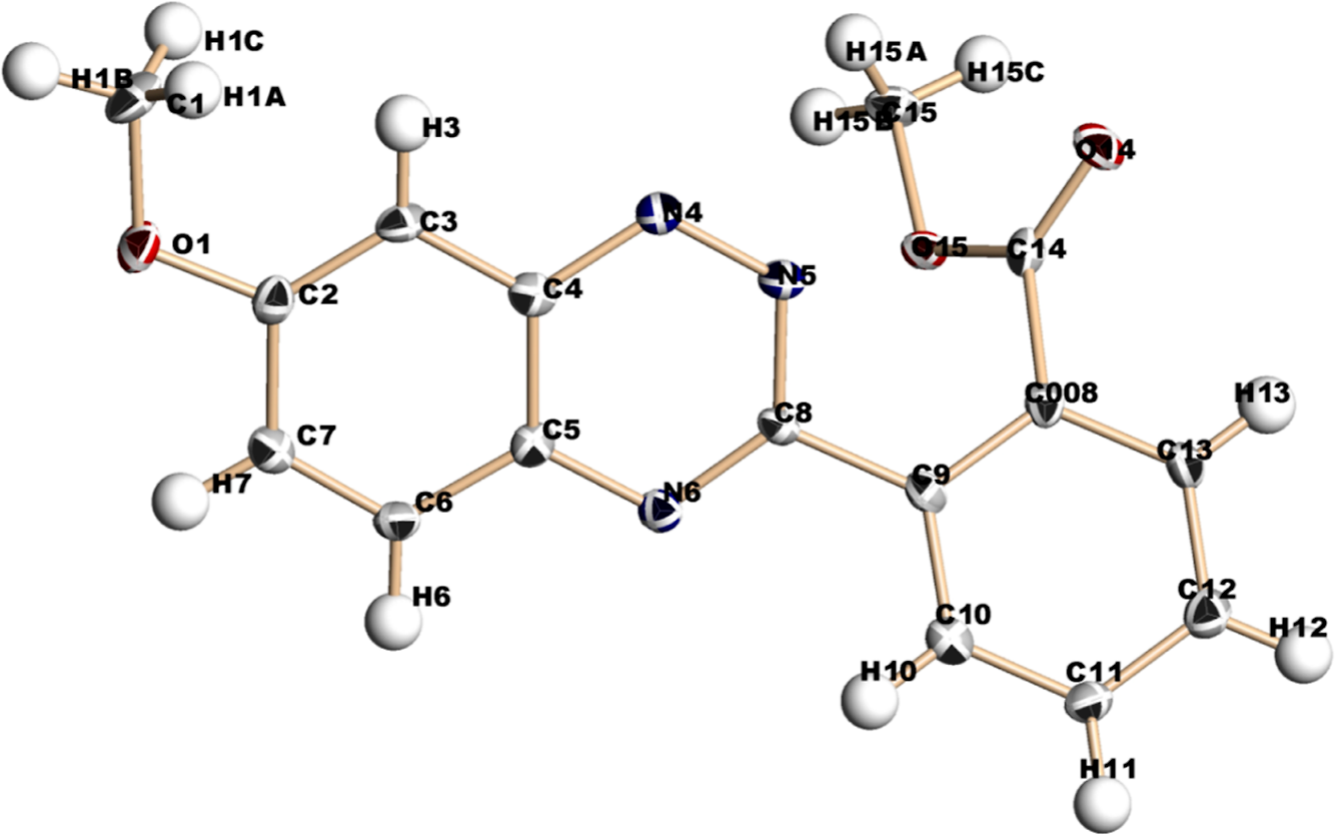
X-ray crystallography of product **16d**.

In addition, the structure of methyl 2-(7-methoxybenzo[e][1,2,4] triazin-3-yl)benzoate **(16d)** was also established by X-ray crystallography (Figure 3).

While esters **(14** and **16a–h)** were the anticipated products from intramolecular cyclization of the isoindolin-1-ones, thin layer chromatography showed the presence of two spots and, indeed, two products were obtained—the esters **(14** and **16a–h)** as well as the much more polar hydrolyzed acids **(15** and **17a–h)**. In fact, heating the reaction for 30 min caused the ester product to rapidly and completely hydrolyze the carboxylate salt. This is a direct consequence of the increased reaction temperature (∼65 °C) and 1 h heating at 60–65 °C. The formation of **15** from **14** is evident from the isolated yields of **14** and **15** as presented in Table 2.

**Table 2:** Isolated % Yields of the Cinnoline Ester **14** and Cinnoline Acid **15** Products Upon Heating

heating time	% yield of **14**	% yield of **15**	combined % yield of **14** and **15**
15 min	82	15	97
30 min	60	34	94
1 h	17	76	93

**Scheme 7:**
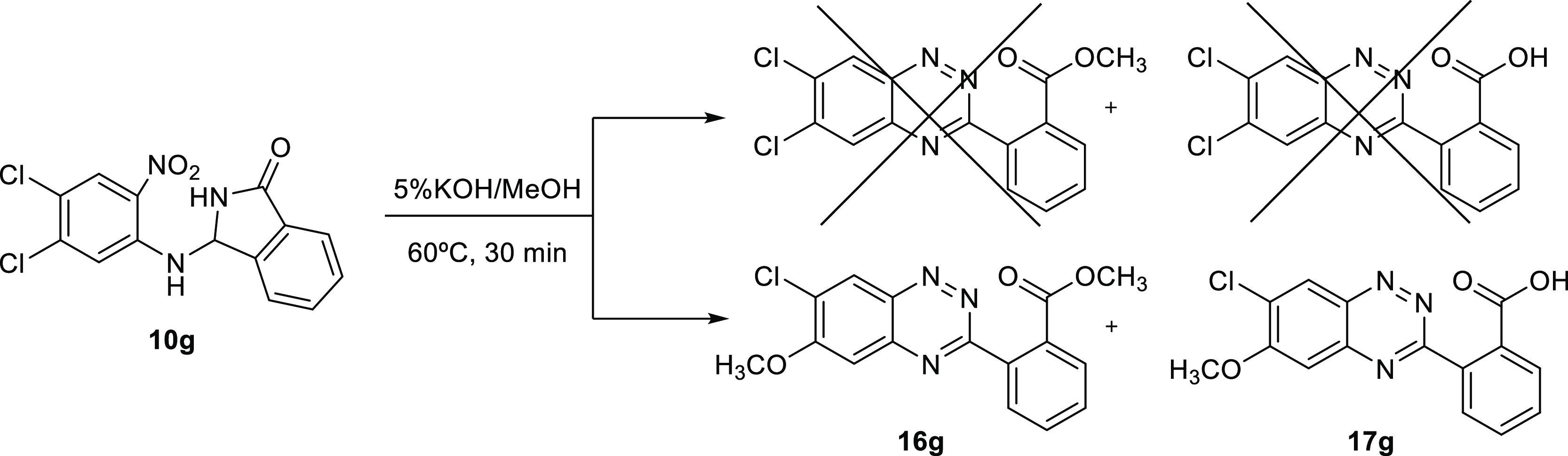
Synthesis of Methyl 2-(7-Chloro-6-methoxybenzo[*e*][1,2,4]triazin-3-yl)benzoate **16g** and 2-(7-Chloro-6-methoxybenzo[*e*][1,2,4]triazin-3-yl)benzoic Acid **17g**

Additionally, in the course of this investigation, a serendipitous reaction was found to occur with isoindolinone **10g**. It formed benzotriazines **16g** and **17g** by electrophilic aromatic substitution of the chloro group para to the nitro moiety. Indeed, the ^1^H and ^13^C NMR data were incompatible with the expected dichloro structures. Theoretically, the dichloro products should show three methoxy protons for the ester at ∼4 ppm in ^1^H NMR as well as a OCH_3_ carbon at ∼50 ppm in the ^13^C NMR. Experimentally, six methoxy protons appeared as two singlets at 4.13 and 4.03 ppm and two OCH_3_ carbons at 57.25 and 57.23 ppm, in addition to the other expected peaks. This result can be explained by chloride displacement by methoxide upon heating of **10g**, where CH_3_O^–^ displaces the chlorine at position 6, para to the nitro group (Scheme 7).

## Conclusions

A total of 27 compounds were successfully synthesized, identified, and characterized by melting points, ^1^H NMR, ^13^C NMR, ^13^C NMR DEPT 135, FT-IR, and HR-MS spectroscopy. The synthesis was accomplished through new, concise, efficient, and low-cost reactions, resulting in fair-to-high yields of the products.

## Experimental Section

Melting points were determined using a DigiMelt digital melting point apparatus and were uncorrected. ^1^H MNR, ^13^C NMR, and Dept 135 spectra were determined in CDCl_3_ or DMSO-*d*_6_ using a Bruker AM 500 NMR spectrometer. Chemical shifts were recorded in ppm (δ). Infrared spectra were collected using a Thermo Scientific iD3 ATR for Nicolet iS5 FT-IR spectrometer in cm^–1^. High-resolution mass spectra (HR-MS) were recorded using a SCIEX X500R HPLC/QTOF mass spectrometer. Thin layer chromatography (TLC) was performed on TLC silica gel 60 F254. Required starting materials were commercially available.

### 2-(2-Nitrophenyl)-2-(3-oxoisoindolin-1-yl)acetonitrile (**6**)

*o*-Cyanobenzaldehyde (0.32 g; 2.50 mmol) and 2-(2-nitrophenyl)acetonitrile (0.34 g; 2.08 mmol) were dissolved in 3 mL of MeOH. A volume of 0.5 mL of Et_3_N was added while stirring the mixture at room temperature. After 2 min, a white precipitate appeared. The product was collected by suction filtration and was washed with cold ethanol (0.48 g; 79%). Melting point: 203 °C; ^1^H NMR (500 MHz, DMSO-*d*_6_): δ 8.25 (d, *J* = 8.0, 1.0 Hz, 1H), 7.89 (dd, *J* = 7.5, 1.5 Hz, 1H), 7.85 (d, *J* = 7.5 Hz, 1H), 7.72–7.75 (m, 3H), 7.60–7.67 (m, 2H), 7.48 (s, 1H), 5.51 (d, *J* = 3 Hz, 1H), 5.22 (d, *J* = 2.5 Hz, 1H); ^13^C NMR (126 MHz, DMSO-*d*_6_): δ 171.11, 147.38, 143.27, 135.03, 133.07, 132.09, 131.42, 130.58, 129.94, 127.34, 126.39, 124.33, 122.89, 116.04, 58.38, 40.23 ppm; DEPT 135 (126 MHz, DMSO-*d*_6_): δ 135.03, 133.07, 131.42, 130.58, 129.94, 126.39, 124.33, 122.89, 58.38, 40.24; FTIR (cm^–1^): 2361 (m), 2343 (w), 1703 (s), 1615 (w), 1532 (s), 1470 (m), 1348 (s), 1306 (w), 1138 (m), 858 (m), 758 (m), 721 (s), 703 (s); *m*/*z*: calcd for C_16_H_11_N_3_O_3_ [M + H]^+^, 294.08732; found, 294.0874, [M + Na]^+^, calcd 316.06926; found, 316.0693, [M + K]^+^, calcd 332.0432; found, 332.0418.

### General Procedure A

Derivatives of 3-((nitrophenyl)amino)isoindolin-1-one were prepared from 2-cyanobenzaldehyde (0.32 g; 2.50 mmol) and 2-nitroaniline derivatives (1 mmol) dissolved in 1 mL of DCM. The mixture was warmed to ensure total dissolution of all the starting materials for 1 min. The reaction mixture was then cooled to room temperature, and 0.4 mL of 5% KOH in MeOH was added. The solution color turned red, and heat was released just before a yellow paste formed. The product was collected by suction filtration and washed with water and cold methanol.

### 3-((Nitrophenyl)amino)isoindolin-1-one (**10a**)

This product was synthesized according to general procedure A as a yellow solid (0.21 g; 79%). Melting point: 235 °C; ^1^H NMR (500 MHz, DMSO-*d*_6_): δ 9.29 (s, 1H), 8.20 (d, *J* = 8.5 Hz, 1H), 8.12 (dd, *J* = 8.5, 1.5 Hz, 1H), 7.76 (d, *J* = 7.5 Hz, 1H), 7.69 (dt, *J* = 7.0, 1.5 Hz, 1H), 7.65–7.67 (m, 1H), 7.62 (dt, *J* = 7.0, 1.5 Hz, 1H), 7.56–7.60 (m, 1H), 7.21 (d, *J* = 8.5 Hz, 1H), 6.85–6.88 (m, 1H), 6.51 (d, *J* = 8 Hz, 1H); ^13^C NMR (126 MHz, DMSO-*d*_6_): δ 169.34, 145.03, 143.66, 136.91, 133.16, 133.05, 132.81, 130.19, 126.76, 123.95, 123.53, 117.82, 116.12, 64.35 ppm; ^13^C NMR DEPT 135 (126 MHz, DMSO-*d*_6_): δ 136.91, 133.05, 130.20, 126.76, 123.95, 123.53, 117.82, 116.13, 64.35; FTIR (cm^–1^): 1714 (s), 1619 (m), 1577 (m), 1498 (m), 1470 (w), 1446 (m), 1409 (w), 1346 (m), 1262 (m), 1228 (m), 1138 (w), 1122 (m), 1069 (w), 869 (w), 738 (s), 696 (w); *m*/*z*: calcd for C_14_H_11_N_3_O_3_ [M + H]^+^, 270.08732; found, 270.1003, [M + Na]^+^, calcd 292.06926; found, 292.0696, [M + K]^+^, calcd 308.0432; found, 308.0528.

### 3-((4-Methyl-2-nitrophenyl)amino)isoindolin-1-one (**10b**)

This product was synthesized according to the general procedure A as a yellow solid (0.20 g; 70%). Melting point 209 °C; ^1^H NMR (500 MHz, DMSO-*d*_6_): δ 9.26 (s, 1H), 8.08 (d, *J* = 8.0 Hz, 1H), 7.93 (d, *J* = 1.0 Hz, 1H), 7.75 (d, *J* = 7.0 Hz, 1H), 7.68 (dt, *J* = 7.5, 1.0 Hz, 1H), 7.64 (d, *J* = 7.0 Hz, 1H), 7.61 (dt, *J* = 7.5, 1.0 Hz, 1H), 7.42 (dd, *J* = 9.0, 2.0 Hz 1H), 7.13 (d, *J* = 8.5 Hz, 1H), 6.48 (d, *J* = 8.0 Hz, 1H), 2.26 (s, 3H); ^13^C NMR (126 MHz, DMSO-*d*_6_): δ 169.32, 145.14, 141.79, 138.15, 133.01, 132.88, 132.81, 130.14, 127.12, 125.84, 123.90, 123.50, 116.23, 64.49, 19.91 ppm; DEPT 135 (126 MHz, DMSO-*d*_6_): δ 138.16, 133.01, 130.14, 127.12, 125.84, 123.90, 123.50, 116.23, 64.49, 19.91; FTIR (cm^–1^): 1710 (s), 1633 (w), 1567 (w), 1524 (m), 1470 (w), 1443 (w), 1409 (w), 1348 (m), 1316 (w), 1273 (m), 1237 (w), 1206 (m), 1156 (w), 1124 (w), 1063 (w), 924 (w), 792 (w), 762 (m), 741 (m), 706 (m); *m*/*z*: calcd for C_15_H_13_N_3_O_3_ [M + H]^+^, 284.10297, found 284.1037, [M + Na]^+^, calcd 306.08491; found, 306.0855, [M + K]^+^, calcd 322.05885; found, 322.0588.

### 3-((4.5-Dimethyl-2-nitrophenyl)amino)isoindolin-1-one (**10c**)

This product was synthesized according to general procedure A as a yellow solid (0.23 g; 77%). Melting point: 257 °C; ^1^H NMR (500 MHz, DMSO-*d*_6_): δ 9.31 (s, 1H), 8.06 (d, *J* = 8.5 Hz, 1H), 7.91 (s, 1H), 7.75 (d, *J* = 7.5 Hz, 1H), 7.70 (dt, *J* = 7.5, 1.0 Hz, 1H), 7.66 (d, *J* = 7.0 Hz, 1H), 7.62 (dt, *J* = 7.5, 1.0 Hz, 1H), 7.15 (s, 1H), 6.49 (d, *J* = 8.0 Hz, 1H), 2.26 (s, 3H), 2.20 (s, 3H); ^13^C NMR (126 MHz, DMSO-*d*_6_): δ 169.34, 147.88, 145.14, 142.21, 133.07, 132.76, 130.86, 130.21, 126.75, 126.06, 123.93, 123.48, 116.54, 64.27, 20.65, 18.56 ppm; DEPT 135 (126 MHz, DMSO-*d*_6_): δ 133.07, 130.21, 126.06, 123.93, 123.93, 123.48, 116.54, 64.27, 20.65, 18.56; FTIR (cm^–1^): 1707 (s), 1632 (w), 1569 (w), 1508 (m), 1471 (w), 1446 (w), 1409 (w), 1330 (w), 1279 (m), 1248 (m), 1210 (w), 1119 (m), 1054 (w), 846 (w), 733 (m); *m*/*z*: calcd for C_16_H_15_N_3_O_3_ [M + H]^+^, 298.11862; found, 298.1318, [M + Na]^+^, calcd 320.10056; found, 320.1008, [M + K]^+^, calcd 336.0745; found, 336.0748.

### 3-((4-Methoxy-2-nitrophenyl)amino)isoindolin-1-one (**10d**)

This product was synthesized according to general procedure A as an orange solid (0.25 g; 83%). Melting point: 208 °C; ^1^H NMR (500 MHz, DMSO-*d*_6_): δ 9.24 (s, 1H), 8.03 (d, *J* = 8.5 Hz, 1H), 7.74 (d, *J* = 7.5 Hz, 1H), 7.68 (t, *J* = 7.0 Hz, 1H), 7.64 (d, *J* = 7.0 Hz, 1H), 7.61 (t, *J* = 7.5 Hz, 1H), 7.57 (d, *J* = 3 Hz, 1H), 7.30 (dd, *J* = 9.5, 3.0 Hz, 1H), 7.18 (d, *J* = 9.5 Hz, 1H), 6.47 (d, *J* = 8.5 Hz, 1H), 3.77 (s, 3H); ^13^C NMR (126 MHz, DMSO-*d*_6_): δ 169.27, 150.88, 145.19, 138.94, 133.01, 132.82, 132.73, 130.15, 126.66, 123.91, 123.51, 117.86, 107.76, 64.74, 56.21 ppm; ^13^C NMR DEPT 135 (126 MHz, DMSO-*d*_6_): δ 133.01, 130.15, 126.66, 123.91, 123.51, 117.86, 107.76, 64.74, 56.21; FTIR (cm^–1^): 1707 (s), 1576 (w), 1526 (m), 1509 (w) 1416 (w), 1343 (w), 1237 (m), 1206 (w), 1058 (m), 1037 (m), 741 (m); *m*/*z*: calcd for C_15_H_13_N_3_O_4_ [M + H]^+^, 300.09788; found, 300.0993, [M + Na]^+^, calcd 322.07983; found, 322.0805, [M + K]^+^, calcd 338.05376; found, 338.0540.

### 3-((5-Methoxy-2-nitrophenyl)amino)isoindolin-1-one (**10e**)

This product was synthesized according to general procedure A as a pale yellow solid (0.27 g; 90%). Melting point: 257 °C; ^1^H NMR (500 MHz, DMSO-*d*_6_): δ 9.37 (s, 1H), 8.50 (d, *J* = 8.0 Hz, 1H), 8.09 (d, *J* = 9.5 Hz, 1H), 7.77 (d, *J* = 7.5 Hz, 1H), 7.70 (dd, *J* = 6.5, 1.0 Hz, 2H), 7.63 (dt, *J* = 7.0, 1.5 Hz, 1H), 6.57 (d, *J* = 8.0 Hz, 2H), 6.45 (dd, *J* = 9.5, 2.5 Hz, 1H), 3.83 (s, 3H); ^13^C NMR (126 MHz, DMSO-*d*_6_): δ 169.35, 165.94, 146.29, 144.85, 133.16, 132.77, 130.28, 129.10, 127.09, 123.98, 123.55, 107.24, 97.70, 64.17, 56.46 ppm; ^13^C NMR DEPT 135 (126 MHz, DMSO-*d*_6_): δ 133.16, 130.28, 129.10, 123.98, 123.55, 107.24, 97.70, 64.18, 56.46; FTIR (cm^–1^): 1709 (s), 1615 (m), 1584 (m), 1499 (m), 1421 (m), 1365 (w), 1313 (w), 1236 (s), 1122 (m), 1088 (w), 1071 (m), 845 (s), 785 (m), 735 (m); *m*/*z*: calcd for C_15_H_13_N_3_O_4_ [M + H]^+^, 300.09788; found, 300.1072, [M + Na]^+^, calcd 322.07983; found, 322.0800, [M + K]^+^, calcd 338.05376; found, 338.0541.

### 3-((4-Chloro-2-nitrophenyl)amino)isoindolin-1-one (**10f**)

This product was synthesized according to general procedure A as a yellow solid (0.24 g; 79%). Melting point: 236 °C; ^1^H NMR (500 MHz, DMSO-*d*_6_): δ 9.26 (s, 1H), 8.20 (d, *J* = 8.0 Hz, 1H), 8.12 (d, *J* = 2.5 Hz, 1H), 7.75 (d, *J* = 7.5 Hz, 1H), 7.68 (dt, *J* = 7.5, 1.0 Hz, 1H), 7.65 (d, *J* = 2.5 Hz, 1H), 7.63 (t, *J* = 2.5 Hz, 1H), 7.61 (dd, *J* = 7.0, 1.0 Hz, 1H), 7.23 (d, *J* = 9.5 Hz, 1H), 6.51 (d, *J* = 8 Hz, 1H); ^13^C NMR (126 MHz, DMSO-*d*_6_): δ 169.29, 144.80, 142.53, 136.46, 133.33, 133.04, 132.84, 130.22, 125.63, 123.96, 123.54, 120.96, 118.18, 64.46 ppm; DEPT 135 (126 MHz, DMSO-*d*_6_): δ 136.46, 133.04, 130.22, 125.63, 123.96, 123.54, 118.18, 64.46; FTIR (cm^–1^): 1708 (s), 1615 (w), 1564 (w), 1520 (m), 1501 (m), 1442 (w), 1409 (m), 1345 (m), 1298 (w), 1265 (s), 1210 (w), 1155 (m), 1123 (w), 1057 (w), 893 (m), 818 (m), 729 (m), 704 (m); *m*/*z*: calcd for C_14_H_10_ClN_3_O_3_ [M + H]^+^, 304.04835; found, 304.1496, [M + Na]^+^, calcd 326.03029; found, 326.0305, [M + K]^+^, calcd 342.00423; found, 342.0034.

### 3-((4,5-Dichloro-2-nitrophenyl)amino)isoindolin-1-one (**10g**)

This product was synthesized according to general procedure A as a yellow solid (0.30 g; 87%). Melting point: 257 °C; ^1^H NMR (500 MHz, DMSO-*d*_6_): δ 9.32 (s, 1H), 8.32 (s, 1H), 8.18 (d, *J* = 8.5 Hz, 1H), 7.75 (d, *J* = 7.5 Hz, 1H), 7.70 (dt, *J* = 7.5, 1.0 Hz, 1H), 7.66 (d, *J* = 7 Hz, 1H), 7.63 (dt, *J* = 7.5, 1.0 Hz, 1H), 7.57 (s, 1H), 6.56 (d, *J* = 8.5 Hz, 1H); ^13^C NMR (126 MHz, DMSO-*d*_6_): δ 169.32, 144.50, 142.82, 139.66, 133.06, 132.79, 132.33, 130.31, 127.79, 124.06, 123.50, 119.11, 117.65, 64.33 ppm; DEPT 135 (126 MHz, DMSO-*d*_6_): δ 133.09, 130.31, 127.79, 124.06, 123.50, 117.65, 64.33; FTIR (cm^–1^): 1713 (s), 1615 (m), 1553 (m), 1469 (s), 1331 (w), 1276 (m), 1260 (s), 1218 (m), 1072 (m), 756 (s), 633 (s); *m*/*z*: calcd for C_14_H_9_Cl_2_N_3_O_3_ [M + H]^+^, 338.00937; found, 338.0095, [M + Na]^+^, calcd 359.99132; found, 359.9914, [M + K]^+^, calcd 375.96525; found, 375.9636.

### 3-((2-Nitro-4-(trifluoromethyl)phenyl)amino)isoindolin-1-one (**10h**)

This product was synthesized according to general procedure A as a yellow solid (0.13 g; 39%). Melting point: 209 °C; ^1^H NMR (500 MHz, DMSO-*d*_6_): δ 9.28 (s, 1H), 8.50 (d, *J* = 8.5 Hz, 1H), 8.37 (d, *J* = 1.5 Hz, 1H), 7.88 (dd, *J* = 9.0, 2.0 Hz, 1H), 7.76 (d, *J* = 7.5 Hz, 1H), 7.69 (dt, *J* = 7.5, 1.0 Hz, H), 7.66 (d, *J* = 6.5 Hz, 1H), 7.63 (dt, *J* = 7.5, 1.5 Hz, 1H), 7.37 (d, *J* = 9.5 Hz, 1H), 6.59 (d, *J* = 8 Hz, 1H); ^13^C NMR (126 MHz, DMSO-*d*_6_): δ 169.35, 145.76, 144.55, 133.07, 132.84, 132.43, 130.28, 125.17, 124.47, 124.43, 124.00, 123.58, 117.59, 117.36, 64.32 ppm; DEPT 135 (126 MHz, DMSO-*d*_6_): δ 133.07, 132.43, 130.28, 124.47, 124.43, 124.00, 123.58, 117.36, 64.32; FTIR (cm^–1^): 1709 (s), 1635 (m), 1571 (w), 1534 (m), 1469 (w), 1446 (w), 1426 (w), 1335 (s), 1303 (w), 1271 (m), 1238 (w), 1211 (w), 1159 (m), 1111 (s), 1089 (w), 1064 (w), 914 (w), 763 (w), 744 (m), 715 (m); *m*/*z*: calcd for C_15_H_10_F_3_N_3_O_3_ [M + H]^+^, 338.0747; found, 338.0761, [M + Na]^+^, calcd 360.0567; found, 360.0571, [M + K]^+^, calcd 376.0306; found, 376.0296.

### General Procedure B

The isoindolin-1-one derivative (0.1 g; 0.3 mmol) was dissolved in 10 mL of 5% KOH in MeOH, and the mixture was heated for 30 min. The color of the solution turned brown, the reaction was quenched with water, and extraction with ethyl acetate was performed. The organic layer was then dried over anhydrous MgSO_4_, and the solvent was evaporated under reduced pressure. The resulting product was purified and identified as the cinnoline or benzotriazine ester.

### General Procedure C

The isoindolin-1-one derivative (0.1 g; 0.3 mmol) was dissolved in 10 mL of 5% KOH in MeOH, and the mixture was heated for 30 min. The color of the solution turned brown, the reaction was quenched with water, and extraction with ethyl acetate was performed. The aqueous layer was then acidified with concentrated HCl. The crude precipitate was filtrated using a Buchner funnel. The product was recrystallized in 2 mL of methanol, collected by vacuum filtration, and washed with cold methanol and identified as the cinnoline or benzotriazine acid.

### Methyl 2-(4-Cyanocinnolin-3-yl)benzoate (**14**)

This product was synthesized according to general procedure B. It was purified by recrystallization in 2 mL of ethanol and collected by filtration as yellow crystals (0.62 mg; 60%). Melting point: 144 °C; ^1^H NMR (500 MHz, CDCl_3_): δ 8.72–8.74 (m, 1H), 8.21–8.23 (m, 2H), 8.00–8.02 (m, 2H), 7.77 (dt, *J* = 7.5, 1.0 Hz, 1H), 7.66–7.71 (m, 2H), 3.70 (s, 3H); ^13^C NMR (126 MHz, CDCl_3_): δ 166.67, 156.64, 148.74, 136.69, 134.13, 132.47, 131.77, 131.45, 131.09, 130.47, 130.30, 124.23, 123.87, 114.04, 106.84, 52.40 ppm; DEPT 135 (126 MHz, CDCl_3_): δ 134.13, 132.47, 131.77, 131.45, 131.09, 130.96, 130.30, 124.23, 52.41; FTIR (cm^–1^): 1716 (s), 1564 (w), 1434 (w), 1294 (w), 1272 (s), 1128 (m), 1084 (m), 1064 (w), 1048 (w), 1030 (m), 772 (s), 771 (m); *m*/*z*: calcd for C_17_H_11_N_3_O_2_ [M + H]^+^, 290.0924; found, 290.0921, [M + Na]^+^, calcd 312.07435; found, 312.0741, [M + K]^+^, calcd 328.04828; found, 328.0484.

### 2-(4-Cyanocinnolin-3-yl)benzoic Acid (**15**)

This product was synthesized according to general procedure C as yellow crystals (0.33 mg; 34%). Melting point: 205 °C; ^1^H NMR (500 MHz, CDCl_3_): δ 8.71–8.72 (m, 1H), 8.24 (d, *J* = 8 Hz, 1H), 8.19–8.22 (m, 1H), 8.00–8.02 (m, 2H), 7.78 (dt, *J* = 7.25, 1.0 Hz, 1H), 7.69 (d, *J* = 8 Hz, 1H), 7.65 (dt, *J* = 8.0, 0.5 Hz, 1H); ^13^C NMR (126 MHz, CDCl_3_): δ 169.46, 156.45, 148.70, 136.91, 134.17, 133.02, 131.84, 131.78, 131.57, 130.89, 130.35, 129.63, 124.24, 123.95, 113.95, 106.99 ppm; DEPT 135 (126 MHz, CDCl_3_): δ 134.18, 133.03, 131.85, 131.79, 131.57, 130.89, 130.36, 124.25; FTIR (cm^–1^): 1700 (w), 1684 (s), 1679 (s), 1673 (s), 1662 (s), 1279 (m), 1148 (w), 1136 (w), 727 (s); *m*/*z*: calcd for C_16_H_9_N_3_O_2_ [M + H]^+^, 276.07675; found, 276.0766, [M + Na]^+^, calcd 298.0587; found, 298.0587, [M + K]^+^, calcd 314.03263; found, 314.0241.

### Methyl 2-(benzo[*e*][1,2,4]triazin-3-yl)benzoate (**16a**)

This product was synthesized according to general procedure B. It was purified by recrystallization in 2 mL of ethanol and collected by filtration as yellow crystals (0.46 mg; 45%). Melting point: 145 °C; ^1^H NMR (500 MHz, CDCl_3_): δ 8.59 (qd, *J* = 8.5, 0.5 Hz, 1H), 8.21 (dd, *J* = 7.5, 1.0 Hz, 1H), 8.11 (qd, *J* = 8.5, 0.5 Hz, 1H), 8.00–8.03 (m, 1H), 7.89–7.92 (m, 2H), 7.71 (dt, *J* = 7.5, 1.5 Hz, 1H), 7.64 (dt, *J* = 7.5, 1.5 Hz, 1H), 3.73 (s, 3H); ^13^C NMR (126 MHz, CDCl_3_): δ 169.04, 161.41, 145.94, 140.61, 136.44, 135.68, 132.83, 131.38, 130.89, 130.69, 130.27, 129.68, 129.68, 128.99, 52.31 ppm; DEPT 135 (126 MHz, CDCl_3_): δ 135.68, 131.38, 130.89, 130.70, 130.27, 129.68, 129.68, 128.99, 52.31; FTIR (cm^–1^): 2923 (w), 1735 (s), 1683 (m), 1625 (w), 1604 (w), 1572 (w), 1524 (m), 1509 (w), 1260 (w), 1096 (w), 867 (w), 778 (m), 743 (m), 705 (s); *m*/*z*: calcd for C_15_H_11_N_3_O_2_ [M + H]^+^, 266.0924; found, 266.0926, [M + Na]^+^, calcd 288.07435; found, 288.0743, [M + K]^+^, calcd 304.04828; found, 304.0485.

### 2-(Benzo[e][1,2,4]triazine-3-yl)benzoic Acid (**17a**)

This product was synthesized according to the general procedure C as yellow crystals (0.48 mg; 51%). Melting point: 180 °C; ^1^H NMR (500 MHz, CDCl_3_): δ 8.54 (dd, *J* = 8.5, 0.5 Hz, 1H), 8.10 (d, *J* = 8.5 Hz, 2H), 7.97–8.01 (m, 2H), 7.86–7.89 (m, 1H), 7.73 (dt, *J* = 7.5, 1.0 Hz, 1H), 7.63 (dt, *J* = 7.5, 1.0 Hz, 1H); ^13^C NMR (126 MHz, CDCl_3_): δ 172.25, 161.55, 145.85, 140.60, 136.97, 135.83, 131.99, 131.77, 131.15, 130.83, 130.43, 130.19, 129.56, 128.97 ppm; DEPT 135 (126 MHz, CDCl_3_): δ 135.83, 131.99, 131.15, 130.83, 130.43, 130.19, 129.55, 128.96; FTIR (cm^–1^): 2928 (w), 1705 (s), 1489 (w), 1451 (w), 1391 (w), 1238 (s) 1120 (m), 1015 (m), 1003 (m), 806 (m), 775 (s), 762 (s), 726 (s), 631 (m); *m*/*z*: calcd for C_14_H_9_N_3_O_2_ [M + H]^+^, 252.07675; found, 252.0767, [M + Na]^+^, calcd 274.0587; found, 274.0587, [M + K]^+^, calcd 290.03263; found, 290.0243.

### Methyl 2-(7-Methylbenzo[e][1,2,4]triazin-3-yl)benzoate (**16b**)

This product was synthesized according to general procedure B. It was purified by recrystallization in 2 mL of ethanol and collected by filtration as yellow crystals (0.20 mg; 20%). Melting point: 143 °C; ^1^H NMR (500 MHz, CDCl_3_): δ 8.33 (s, 1H), 8.20 (dd, *J* = 8.0, 1.0 Hz, 1H), 7.99 (d, *J* = 8.5 Hz, 1H), 7.88 (dd, *J* = 8.0, 1.5 Hz, 1H), 7.84 (dd, *J* = 8.5, 2.0 Hz, 1H), 7.70 (dt, *J* = 7.5, 1.5 Hz, 1H), 7.62 (dt, *J* = 7.5, 1.0 Hz, 1H), 3.72 (s, 3H), 2.68 (s, 3H); ^13^C NMR (126 MHz, CDCl_3_): δ 169.17, 160.95, 145.98, 141.65, 139.33, 138.29, 136.49, 132.82, 131.29, 130.77, 130.10, 129.61, 128.45, 127.88, 52.26, 22.05 ppm; DEPT 135 (126 MHz, CDCl_3_): δ 138.29, 131.29, 130.77, 130.10, 129.60, 128.45, 127.88, 52.26, 22.05; FTIR (cm^–1^): 1727 (s), 1558 (m), 1501 (m), 1431 (m), 1417 (m), 1318 (m), 1287 (s), 1249 (m), 1119 (m), 1087 (m), 827 (m), 734 (s); *m*/*z*: calcd for C_16_H_13_N_3_O_2_ [M + H]^+^, 280.10805; found, 280.1078, [M + Na]^+^, calcd 302.0900; found, 302.0898, [M + K]^+^, calcd 318.06393; found, 318.0640.

### 2-(7-Methylbenzo[*e*][1,2,4]triazin-3-yl)benzoic Acid (**17b**)

This product was synthesized according to general procedure C as yellow crystals (0.58 mg; 61%). Melting point: 196 °C; ^1^H NMR (500 MHz, CDCl_3_): δ 8.30 (d, *J* = 0.5 Hz, 1H), 8.13 (dd, *J* = 7.5, 0.5 Hz, 1H), 8.01 (t, *J* = 9.0 Hz, 2H), 7.83 (dd, *J* = 8.5, 1.5 Hz, 1H), 7.73 (dt, *J* = 7.5, 1.5 Hz, 1H), 7.63 (dt, *J* = 7.5, 0.5 Hz, 1H), 2.97 (s, 3H); ^13^C NMR (126 MHz, CDCl_3_): δ 171.60, 161.04, 145.93, 141.91, 139.33, 138.54, 136.92, 131.96, 161.67, 131.16, 130.56, 130.10, 128.40, 127.78, 22.06 ppm; DEPT 135 (126 MHz, CDCl_3_): δ 138.54, 131.96, 131.16, 130.56, 130.10, 128.40, 127.78, 22.07; FTIR (cm^–1^): 1690 (m), 1653 (w), 1263 (m), 1007 (w), 836 (m), 761 (m), 710 (w), 672 (w); *m*/*z*: calcd for C_15_H_11_N_3_O_2_ [M + H]^+^, 266.0924; found, 266.0925, [M + Na]^+^, calcd 288.07435; found, 288.0744, [M + K]^+^, calcd 304.04828; found, 304.0413.

### Methyl 2-(6,7-Dimethylbenzo[*e*][1,2,4]triazin-3-yl)benzoate (**16c**)

This product was synthesized according to general procedure B. It was purified by silica gel column chromatography (9:1 hexane/ethyl acetate) and obtained as a yellow solid (0.48 mg; 48%). Melting point: 150 °C; ^1^H NMR (500 MHz, CDCl_3_): δ 8.28 (s, 1H), 8.20 (dd, *J* = 8.0, 1.0 Hz, 1H), 7.87 (dd, *J* = 7.7, 1.0 Hz, 1H), 7.82 (s, 1H), 7.68 (dt, *J* = 7.5, 1.5 Hz, 1H), 7.60 (dt, *J* = 7.6, 1.5 Hz, 1H), 3.71 (s, 3H), 2.58 (s, 3H), 2.56 (s, 3H); ^13^C NMR (126 MHz, CDCl_3_): δ 169.30, 160.93, 147.74, 145.40, 141.94, 139.90, 136.62, 132.83, 131.22, 130.71, 129.99, 129.53, 128.05, 127.45, 52.26, 21.11, 20.55 ppm; DEPT 135 (126 MHz, CDCl_3_): δ 131.22, 130.71, 129.99, 129.52, 128.04, 127.45, 52.26, 21.11, 20.55; FTIR (cm^–1^): 2923 (m), 1723 (s), 1456 (w), 1377 (w), 1331 (w), 1286 (m), 1249 (w), 1114 (m), 1089 (m), 1049 (m), 1023 (w), 999 (w), 862 (w), 771 (m), 719 (s), 706 (w);*m*/*z*: calcd for C_17_H_15_N_3_O_2_ [M + H]^+^, 294.1237; found, 294.1234, [M + Na]^+^, calcd 316.10565; found, 316.1056, [M + K]^+^, calcd 332.07958; found, 322.0797.

### 2-(6,7-Dimethylbenzo[*e*][1,2,4]triazin-3-yl)benzoic Acid (**17c**)

This product was synthesized according to general procedure C as yellow crystals (0.32 mg; 34%). Melting point: 214 °C; ^1^H NMR (500 MHz, CDCl_3_): δ 8.28 (s, 1H), 8.20 (dd, *J* = 7.5, 1.0 Hz, 1H), 8.07 (dd, *J* = 7.5, 1.0 Hz, 1H), 7.85 (s, 1H), 7.73 (dt, *J* = 7.6, 1.5 Hz, 1H), 7.64 (dt, *J* = 7.6, 1.0 Hz, 1H), 2.58 (s, 3H), 2.56 (s, 3H); ^13^C NMR (126 MHz, CDCl_3_): δ 170.87, 160.94, 148.27, 145.37, 142.36, 139.81, 136.76, 131.90, 131.77, 131.25, 130.82, 130.10, 127.96, 127.33, 21.11, 20.57 ppm; DEPT 135 (126 MHz, CDCl_3_): δ 131.89, 131.25, 130.82, 130.09, 127.95, 127.32, 21.11, 20.57; FTIR (cm^–1^): 2925 (w), 1706 (s), 1653 (w), 1468 (w), 1442 (w), 1332 (w), 1260 (w), 1220 (m), 1126 (m), 1020 (w), 997 (w), 870 (m), 794 (m), 771 (s), 754 (w), 724 (s), 668 (s), 634 (m); *m*/*z*: calcd for C_16_H_13_N_3_O_2_ [M + H]^+^, 280.10805; found, 280.1078, [M + Na]^+^, calcd 302.0900; found, 302.0899, [M + K]^+^, calcd 318.06393; found, 318.0602.

### Methyl 2-(7-Methoxybenzo[*e*][1,2,4]triazin-3-yl)benzoate (**16d**)

3-((4-Methoxy-2-nitrophenyl)amino)isoindolin-1-one **10d** (0.1 g, 0.3 mmol) was dissolved in 10 mL of 5% KOH in MeOH, and the mixture was heated for 15 min. The color of the solution turned orange, the reaction was quenched with water, and extraction with ethyl acetate was performed. The organic layer was then dried with anhydrous MgSO_4_, and the solvent was evaporated under reduced pressure. The product was recrystallized in 2 mL of ethanol and collected by filtration as orange crystals (0.54 mg; 54%). Melting point: 151 °C; ^1^H NMR (500 MHz, CDCl_3_): δ 8.21 (dd, *J* = 7.5, 1.0 Hz, 1H), 7.99 (d, *J* = 9.5 Hz, 1H), 7.90 (dd, *J* = 7.5, 1.0 Hz, 1H), 7.78 (d, *J* = 2.5 Hz, 1H), 7.71 (dt, *J* = 7.5, 1.5, 1H), 7.67 (dd, *J* = 9.5, 2.5, 1H), 7.63 (dt, *J* = 7.5, 1.0 Hz, 1H), 4.09 (s, 3H), 3.75 (s, 3H); ^13^C NMR (126 MHz, CDCl_3_): δ 169.24, 161.03, 160.36, 147.33, 137.72, 136.50, 132.70, 131.27, 130.62, 130.35, 129.99, 129.94, 129.58, 105.05, 56.23, 52.25 ppm; DEPT 135 (126 MHz, CDCl_3_): δ 131.27, 130.62, 130.35, 129.99, 129.94, 129.58, 56.23, 52.25; FTIR (cm^–1^): 1726 (s), 1618 (w), 1506 (w), 1426 (s), 1290 (m), 1254 (w), 1199 (s), 1171 (w), 1089 (s), 1015 (m), 838 (s), 776 (m), 763 (s), 737 (m), 696 (m), 604 (s); *m*/*z*: calcd for C_16_H_13_N_3_O_3_ [M + H]^+^, 296.1029; found, 296.1027, [M + Na]^+^, calcd 318.0850; found, 318.0848, [M + K]^+^, calcd 334.0589; found, 334.0588.

### 2-(7-Methoxybenzo[*e*][1,2,4]triazin-3-yl)benzoic Acid (**17d**)

3-((4-Methoxy-2-nitrophenyl)amino)isoindolin-1-one **10d** (0.1 g, 0.3 mmol) was dissolved in 10 mL of 5% KOH in MeOH, and the mixture was heated for 15 min. The color of the solution turned orange, the reaction was quenched with water, and extraction with ethyl acetate was performed. The aqueous layer was then acidified with concentrated HCl and extracted again with ethyl acetate. The latter was dried with anhydrous MgSO_4_, and the solvent was evaporated under reduced pressure. The product was recrystallized in 2 mL of methanol and collected by filtration as yellow crystals (0.40 mg; 42%). Melting point: 230 °C; ^1^H NMR (500 MHz, DMSO-*d*_6_): δ 12.88 (s, 1H), 8.08 (d, *J* = 9.5 Hz, 1H), 7.96 (dd, *J* = 7.5, 1.0 Hz, 1H), 7.92 (d, *J* = 2.5 Hz, 1H), 7.89 (dd, *J* = 7.5, 1.0 Hz, 1H), 7.85 (dd, *J* = 9.0, 2.5 Hz, 1H), 7.76 (dt, *J* = 7.5, 1.5 Hz, 1H), 7.69 (dt, *J* = 7.5, 1.0 Hz, 1H), 4.05 (s, 3H); ^13^C NMR (126 MHz, DMSO-*d*_6_): δ 169.55, 161.32, 161.06, 147.27, 137.31, 137.10, 133.85, 131.69, 131.05, 131.02, 130.40, 130.35, 129.78, 105.46, 56.92 ppm; DEPT 135 (126 MHz, DMSO-*d*_6_): δ 131.70, 131.05, 131.02, 130.40, 130.35, 129.78, 105.46, 56.92; FTIR (cm^–1^): 1705 (s), 1620 (w), 1501 (m), 1432 (m), 1288 (w), 1228 (s), 1204 (s), 1178 (w), 1117 (m), 1042 (m), 1015 (s), 850 (s), 773 (s), 723 (m), 668 (m); *m*/*z*: calcd for C_15_H_11_N_3_O_3_ [M + H]^+^, 282.0867; found, 282.0870, [M + Na]^+^, calcd 304.0692; found, 304.0692, [M + K]^+^, calcd 320.0432; found, 320.0368.

### Methyl 2-(6-Methoxybenzo[*e*][1,2,4]triazin-3-yl)benzoate (**16e**)

3-((5-Methoxy-2-nitrophenyl)amino)isoindolin-1-one **10e** (0.1 g, 0.3 mmol) was dissolved in 10 mL of 5% KOH in MeOH, and the mixture was heated for 15 min. The color of the solution turned light brown, the reaction was quenched with water, and extraction with ethyl acetate was performed. The organic layer was then dried by anhydrous MgSO_4_, and the solvent was evaporated under reduced pressure. The product was purified by silica gel column chromatography (6:4 hexane/ethyl acetate) and obtained as an oily/sticky brown solid (0.48 mg; 47%). ^1^H NMR (500 MHz, CDCl_3_): δ 8.39 (d, *J* = 9.5 Hz, 1H), 8.15 (dd, *J* = 7.5, 1.0 Hz, 1H), 7.89 (dd, *J* = 7.5, 1.0 Hz, 1H), 7.69 (dt, *J* = 7.5, 1.0 Hz, 1H), 7.61 (dt, *J* = 7.5, 1.5 Hz, 1H), 7.49 (dd, *J* = 10.0, 2.5 Hz, 1H), 7.24 (d, *J* = 2.5 Hz, 1H), 4.03 (s, 3H), 3.72 (s, 3H); ^13^C NMR (126 MHz, CDCl_3_): δ 169.13, 165.18, 161.70, 143.67, 143.52, 136.79, 132.75, 131.29, 130.95, 130.72, 130.07, 129.61, 125.33, 104.56, 56.33, 52.28 ppm; DEPT 135 (126 MHz, CDCl_3_): δ 131.29, 130.95, 130.72, 130.07, 129.61, 125.33, 104.56, 56.33, 52.28; FTIR (cm^–1^): 1724 (s), 1615 (s), 1510 (w), 1468 (s), 1432 (w), 1410 (s), 1318 (w), 1291 (m), 1241 (w), 1219 (s), 1180 (w), 1113 (m), 1088 (m), 1048 (w), 1013 (m), 836 (m), 770 (m), 728 (s), 708 (m); *m*/*z*: calcd for C_16_H_13_N_3_O_3_ [M + H]^+^, 296.10297; found, 296.1027, [M + Na]^+^, calcd 318.0849; found, 318.0850, [M + K]^+^, calcd 334.0588; found, 334.0590.

### 2-(6-Methoxybenzo[*e*][1,2,4]triazin-3-yl)benzoic Acid (**17e**)

3-((5-Methoxy-2-nitrophenyl) amino)isoindolin-1-one **10e** (0.1 g, 0.3 mmol) was dissolved in 10 mL of 5% KOH in MeOH, and the mixture was heated for 15 min. The color of the solution turned light brown, the reaction was quenched with water, and extraction with ethyl acetate was performed. The aqueous layer was then acidified with concentrated HCl and extracted again with ethyl acetate. The latter was dried with anhydrous MgSO_4_, and the solvent was evaporated under reduced pressure. The product was recrystallized in 2 mL of methanol and collected by filtration as yellow crystals (0.43 mg; 45%). Melting point: 212 °C; ^1^H NMR (500 MHz, DMSO-*d*_6_): δ 12.90 (s, 1H), 8.46 (d, *J* = 9.5 Hz, 1H), 7.93 (dd, *J* = 7.5, 1.0 Hz, 1H), 7.89 (dd, *J* = 7.5, 1.0 Hz, 1H), 7.75 (dt, *J* = 7.5, 1.5 Hz, 1H), 7.66–7.71 (m, 2H), 7.44 (d, *J* = 2.5 Hz, 1H), 4.05 (s, 3H); ^13^C NMR (126 MHz, DMSO-*d*_6_): δ; 168.30, 164.38, 161.36, 142.39, 142.23, 136.30, 132.88, 130.52, 129.94, 129.94, 129.35, 128.67, 124.91, 104.27, 56.12 ppm; DEPT 135 (126 MHz, DMSO-*d*_6_): δ 130.52, 129.94, 129.94, 129.36, 128.67, 124.91, 104.27, 56.12; FTIR (cm^–1^): 1717 (s), 1615 (s), 1469 (s), 1319 (w), 1221 (m), 1179 (w), 1116 (m), 1089 (w), 1013 (m), 837 (m), 769 (m), 726 (m); *m*/*z*: calcd for C_15_H_11_N_3_O_2_ [M + H]^+^, 282.08732; found, 282.0872, [M + Na]^+^, calcd 304.06926; found, 304.0694, [M + K]^+^, calcd 320.0432; found, 320.0400.

### Methyl 2-(7-Chlorobenzo[*e*][1,2,4]triazin-3-yl)benzoate (**16f**)

This product was synthesized according to general procedure B. It was purified by recrystallization in 2 mL of ethanol and collected by filtration as orange crystals (0.82 mg; 78%). Melting point: 113 °C; ^1^H NMR (500 MHz, CDCl_3_): δ 8.58 (d, *J* = 2.0 Hz, 1H), 8.18 (dd, *J* = 7.5, 1.0 Hz, 1H), 8.06 (d, *J* = 9.0 Hz, 1H), 7.94 (dd, *J* = 9.0, 2.5 Hz, 1H), 7.91 (dd, *J* = 7.5, 1.0 Hz, 1H), 7.72 (dt, *J* = 7.5, 1.5 Hz, 1H), 7.65 (dt, *J* = 7.5, 1.5 Hz, 1H), 3.73 (s, 3H); ^13^C NMR (126 MHz, CDCl_3_): δ 168.87, 161.62, 145.76, 139.24, 136.89, 136.56, 136.09, 132.79, 131.44, 130.88, 130.53, 130.48, 129.77, 128.25, 52.34 ppm; DEPT 135 (126 MHz, CDCl_3_): δ 136.90, 131.44, 130.88, 130.53, 130.48, 129.77, 128.25, 52.34; FTIR (cm^–1^): 1729 (s), 1689 (w), 1506 (m), 1413 (m), 1288 (s), 1118 (m), 1084 (m), 1043 (w), 1011 (m), 902 (m), 849 (m), 839 (m), 774 (m), 741 (m), 721 (s), 705 (m); *m*/*z*: calcd for C_15_H_10_ClN_3_O_2_ [M + H]^+^, 300.05343; found, 300.0534, [M + Na]^+^, calcd 322.03537; found, 322.0351, [M + K]^+^, calcd 338.00931; found, 338.0094.

### 2-(7-Chlorobenzo[*e*][1,2,4]triazin-3-yl)benzoic Acid (**17f**)

This product was synthesized according to general procedure C as yellow crystals (0.19 mg; 19%). Melting point: 177 °C; ^1^H NMR (500 MHz, CDCl_3_): δ 8.53 (d, *J* = 2.5 Hz, 1H), 8.04–8.09 (m, 2H), 8.00 (d, *J* = 7.5 Hz, 1H), 7.92 (dd, *J* = 9.0, 2.0 Hz, 1H), 7.74 (dt, *J* = 7.5, 1.0 Hz, 1H), 7.65 (dt, *J* = 7.5, 1.0 Hz, 1H); ^13^C NMR (126 MHz, CDCl_3_): δ 172.14, 161.77, 145.70, 139.24, 137.01, 136.74, 136.70, 132.09, 131.63, 131.10, 130.56, 130.43, 130.38, 128.10 ppm; DEPT 135 (126 MHz, CDCl_3_): δ 137.01, 132.09, 131.10, 130.56, 130.43, 130.38, 128.11; FTIR (cm^–1^): 1704 (s), 1488 (w), 1431 (w), 1417 (w), 1224 (s), 1206 (m), 1117 (m), 1043 (m), 1019 (m), 1006 (m), 847 (s), 804 (w), 772 (s), 720 (m), 668 (s), 652 (m), 647 (m), 636 (m); *m*/*z*: calcd for C_14_H_8_ClN_3_O_2_ [M + H]^+^, 286.03778; found, 286.0378, [M + Na]^+^, calcd 308.01972; found, 308.0198, [M + K]^+^, calcd 323.99366; found, 323.0094.

### Methyl 2-(7-Chloro-6-methoxybenzo[*e*][1,2,4]triazin-3-yl)benzoate (**16g**)

This product was synthesized according to general procedure B. It was purified by recrystallization in 2 mL of ethanol and collected by filtration as yellow crystals (0.42 mg; 42%). Melting point: 165 °C; ^1^H NMR (500 MHz, CDCl_3_): δ 8.55 (s, 1H), 8.13 (dd, *J* = 8.0, 1.0 Hz, 1H), 7.90 (dd, *J* = 8.0, 1.0 Hz, 1H), 7.69 (dt, *J* = 7.6, 1.5 Hz, 1H), 7.62 (dt, *J* = 7.6, 1.5 Hz, 1H), 7.33 (s, 1H), 4.13 (s, 3H), 3.72 (s, 3H); ^13^C NMR (126 MHz, CDCl_3_): δ 168.99, 161.78, 160.67, 142.73, 142.12, 136.49, 132.69, 131.37, 130.70, 130.25, 130.20, 129.87, 129.70, 105.74, 57.25, 52.32 ppm; DEPT 135 (126 MHz, CDCl_3_): δ 131.37, 130.70, 130.25, 129.87, 129.70, 105.74, 57.25, 52.33; FTIR (cm^–1^): 1727 (s), 1695 (w), 1602 (w), 1474 (m), 1410 (m), 1282 (m), 1242 (w), 1226 (m), 1116 (w), 1089 (m), 1040 (m), 1023 (w), 999 (w), 880 (w), 841 (m), 732 (s); *m*/*z*: calcd for C_16_H_12_ClN_3_O_3_ [M + H]^+^, 330.0640; found, 330.0637, [M + Na]^+^, calcd 352.04594; found, 352.0459, [M + K]^+^, calcd 368.01988; found, 368.0198.

### 2-(7-Chloro-6-methoxybenzo[*e*][1,2,4]triazin-3-yl)benzoic Acid (**17g**)

This product was synthesized according to general procedure C as yellow crystals (0.46 mg; 48%). Melting point: 215 °C; ^1^H NMR (500 MHz, CDCl_3_): δ 8.49 (s, 1H), 8.11 (dd, *J* = 8.0, 1.0 Hz, 1H), 7.98 (dd, *J* = 7.5, 1.0 Hz, 1H), 7.72 (dt, *J* = 7.6, 1.5 Hz, 1H), 7.63 (dt, *J* = 7.6, 1.0 Hz, 1H), 7.37 (s, 1H), 4.03 (s, 3H); ^13^C NMR (126 MHz, CDCl_3_): δ 171.88, 161.63, 160.82, 142.71, 142.11, 136.68, 131.91, 131.86, 131.03, 130.49, 130.42, 130.29, 129.71, 105.79, 57.23 ppm; DEPT 135 (126 MHz, CDCl_3_): δ 131.92, 131.03, 130.42, 130.29, 129.71, 105.76, 57.23; FTIR (cm^–1^): 1705 (s), 1598 (w), 1474 (s), 1413 (s), 1264 (m), 1244 (s), 1225 (m), 1123 (w), 1041 (m), 1021 (m), 997 (m), 768 (s); *m*/*z*: calcd for C_15_H_10_ClN_3_O_3_ [M + H]^+^, 316.04835; found, 316.0483 [M + Na]^+^, calcd 338.03029; found, 338.0305, [M + K]^+^, calcd 354.00423; found, 353.9991.

### Methyl 2-(7-(Trifluoromethyl)benzo[e][1,2,4]triazin-3-yl)benzoate (**16h**)

This product was synthesized according to general procedure B. It was purified by silica gel column chromatography (9:1 hexane/ethyl acetate) and obtained as an oily/sticky yellow solid (0.44 mg, 44%). ^1^H NMR (500 MHz, CDCl_3_): δ 8.91 (s, 1H), 8.24 (d, *J* = 9 Hz, 1H), 8.18 (d, *J* = 7.5 Hz, 1H), 8.16 (dd, *J* = 8.75, 2.0 Hz, 1H), 7.94 (d, *J* = 7.5 Hz, 1H), 7.73 (t, *J* = 7.5 Hz, 1H), 7.67 (t, *J* = 8 Hz, 1H), 3.74 (s, 3H); ^13^C NMR (126 MHz, CDCl_3_): δ 168.69, 162.71, 144.65, 141.56, 135.91, 132.87, 132.22, 131.56, 131.06, 130.81, 130.72, 129.88, 127.92, 124.10, 121.93, 52.40 ppm; DEPT 135 (126 MHz, CDCl_3_): δ 132.87, 132.22, 131.56, 131.06, 130.81, 130.72, 129.89, 127.92, 52.40; FTIR (cm^–1^): 1731 (s), 1684 (m), 1561 (m), 1421 (m), 1322 (s), 1186 (m), 1149 (m), 1122 (s), 1092 (m), 1056 (m), 772 (m), 745 (m), 708 (s), 614 (s); *m*/*z*: calcd for C_16_H_10_F_3_N_3_O_2_ [M + H]^+^, 334.07979; found, 334.0796, [M + Na]^+^, calcd 356.06173; found, 356.0614, [M + K]^+^, calcd 372.03567; found, 372.0358.

### 2-(7-(Trifluoromethyl)benzo[*e*][1,2,4]triazin-3-yl)benzoic Acid (**17h**)

This product was synthesized according to general procedure C as yellow crystals (0.49 mg; 51%). Melting point: 161 °C; ^1^H NMR (500 MHz, CDCl_3_): δ 8.88 (s, 1H), 8.27 (d, *J* = 8.5 Hz, 1H), 8.16 (dd, *J* = 9.0, 1.5 Hz, 1H), 8.12 (d, *J* = 7.5 Hz, 1H), 8.04 (d, *J* = 7.5 Hz, 1H), 7.77 (t, *J* = 7.5 Hz, 1H), 7.68 (t, *J* = 7.5 Hz, 1H); ^13^C NMR (126 MHz, CDCl_3_): δ 171.77, 162.80, 144.67, 141.55, 136.61, 132.35, 132.27, 131.50, 131.30, 131.16, 130.76, 130.71, 130.53, 127.87, 127.84 ppm; DEPT 135 (126 MHz, CDCl_3_): δ 132.28, 131.32, 131.18, 130.77, 130.71, 130.54, 127.87; FTIR (cm^–1^): 1722 (m), 1423 (w), 1323 (s), 1235 (m), 1188 (m), 1155 (w), 1123 (s), 1057 (w), 1009 (w), 847 (m), 771 (m), 730 (m), 654 (m); *m*/*z*: calcd for C_15_H_8_F_3_N_3_O_2_ [M + H]^+^, 320.06414; found, 320.0643, [M + Na]^+^, calcd 342.04608; found, 342.0463, [M + K]^+^, calcd 358.02002; found, 358.0122.

## Supporting Information Available

Additional ^1^H NMR, ^13^C NMR, ^13^C NMR DEPT 135, FTIR, HRMS spectra and single X-ray crystallography (PDF)
Crystallographic data and structure of product: methyl 2-(7-methoxybenzo[e][1,2,4]triazin-3-yl)benzoate (**16d**). (CIF)

## Supplementary Material

ao2c03045_si_001.pdf

ao2c03045_si_002.cif
