# Supplementary material for: Preparation of 3-Substituted Isoindolin-1-one, Cinnoline, and 1,2,4-[e]-Benzotriazine Derivatives
Source: ACS Omega. 2022 Jul 20;7(30):26871–80. doi: 10.1021/acsomega.2c03045 (PMC9352262; doi:10.1021/acsomega.2c03045)

Preparation of 3-substituted isoindolin-1-one, cinnoline and 1,2,4-[*e*]-benzotriazine derivatives

Fatat B. El Dhaibi, † Ali Youssef, † James C. Fettinger, ‡ Mark J. Kurth, ‡ Makhluf J. Haddadin. *,†

† Department of Chemistry, American University of Beirut, Riad El Solh, 1107 2020 Beirut, Lebanon

‡ Department of Chemistry, University of California, One Shields Avenue, Davis, California 95616, United State

Corresponding Author

*Email: haddadin@aub.edu.lb.

TABLE OF CONTENTS

Appendix A: ^{1}H, ^{13}C, ^{13}C DEPT 135 NMR spectroscopy S2

Appendix B: IR spectroscopy S83

Appendix C: HR-MS spectroscopy: S110

Appendix D: X-Ray Crystallography of 16d: S124

APPENDIX A: ^{1}H, ^{13}C, ^{13}C DEPT 135 NMR SPECTROSCOPY

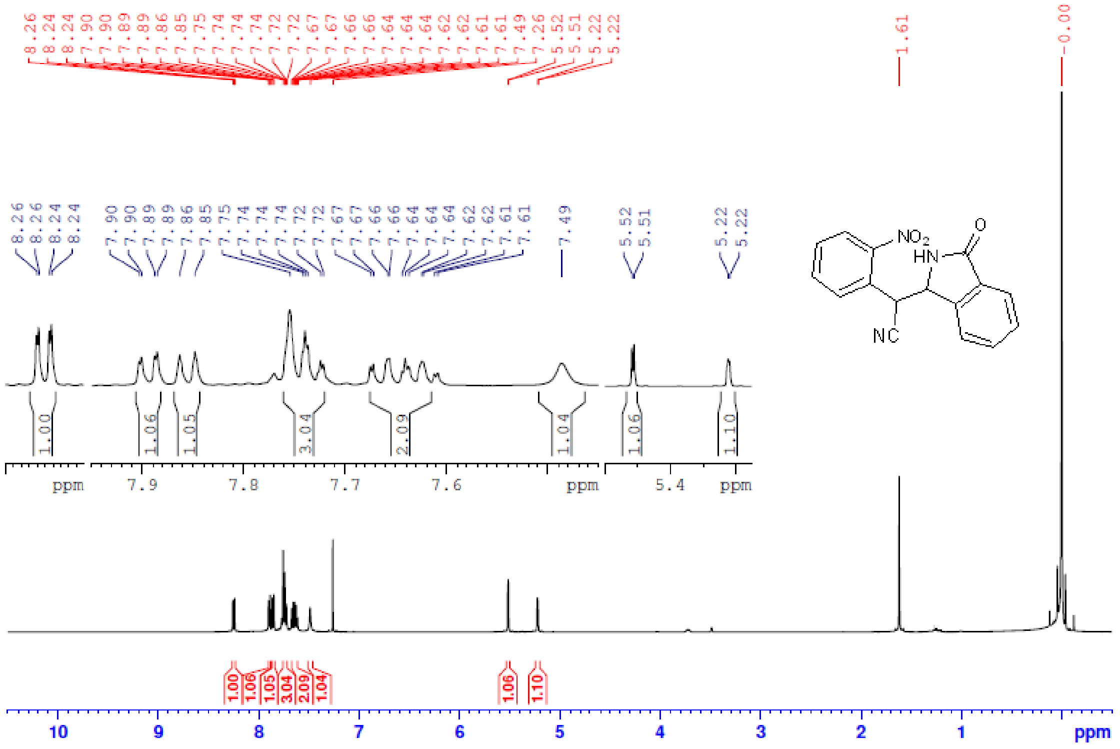

^{1}H-NMR of **6** in $CDCl_3$ at room temperature.

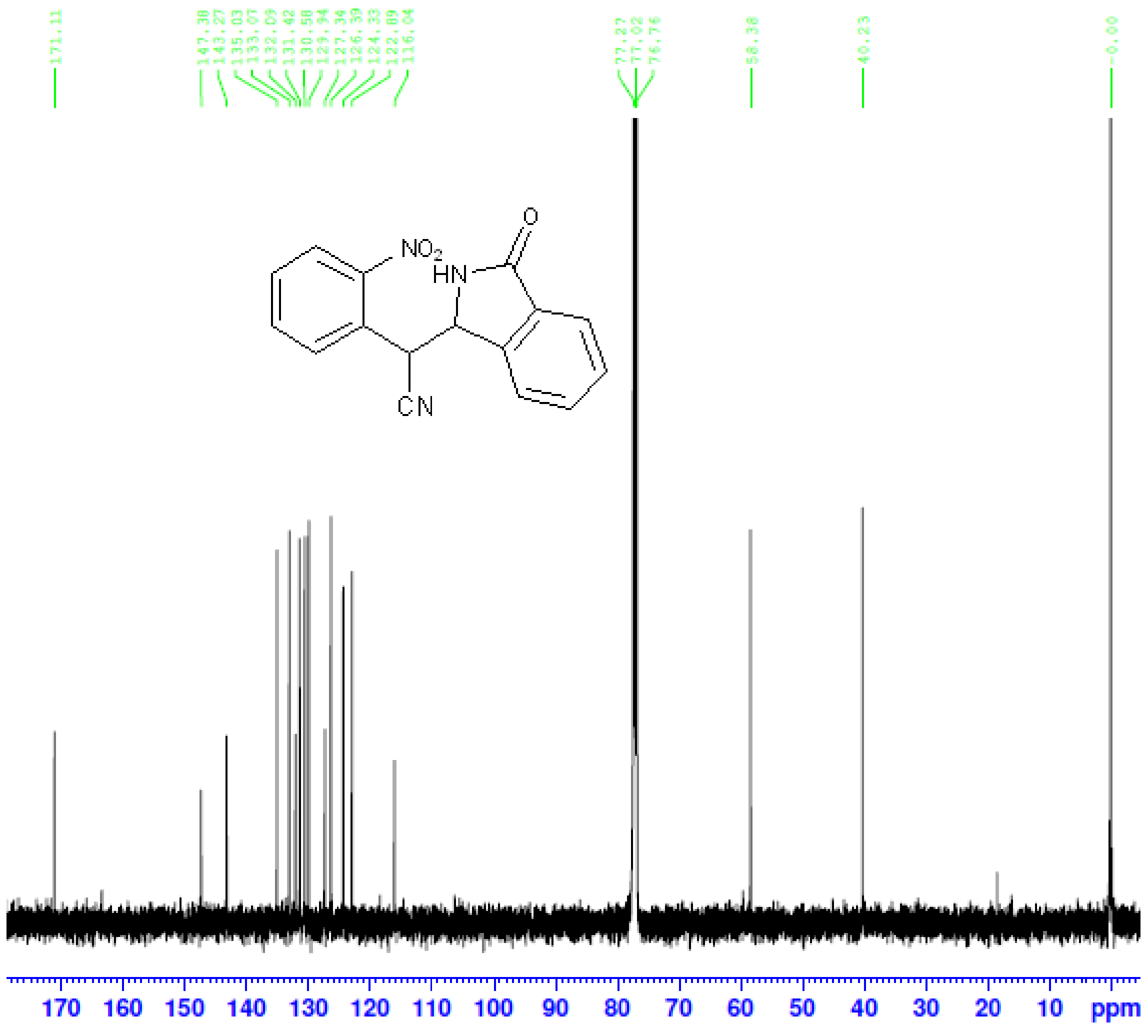

^{13}C-NMR of **6** in $CDCl_3$ at room temperature.

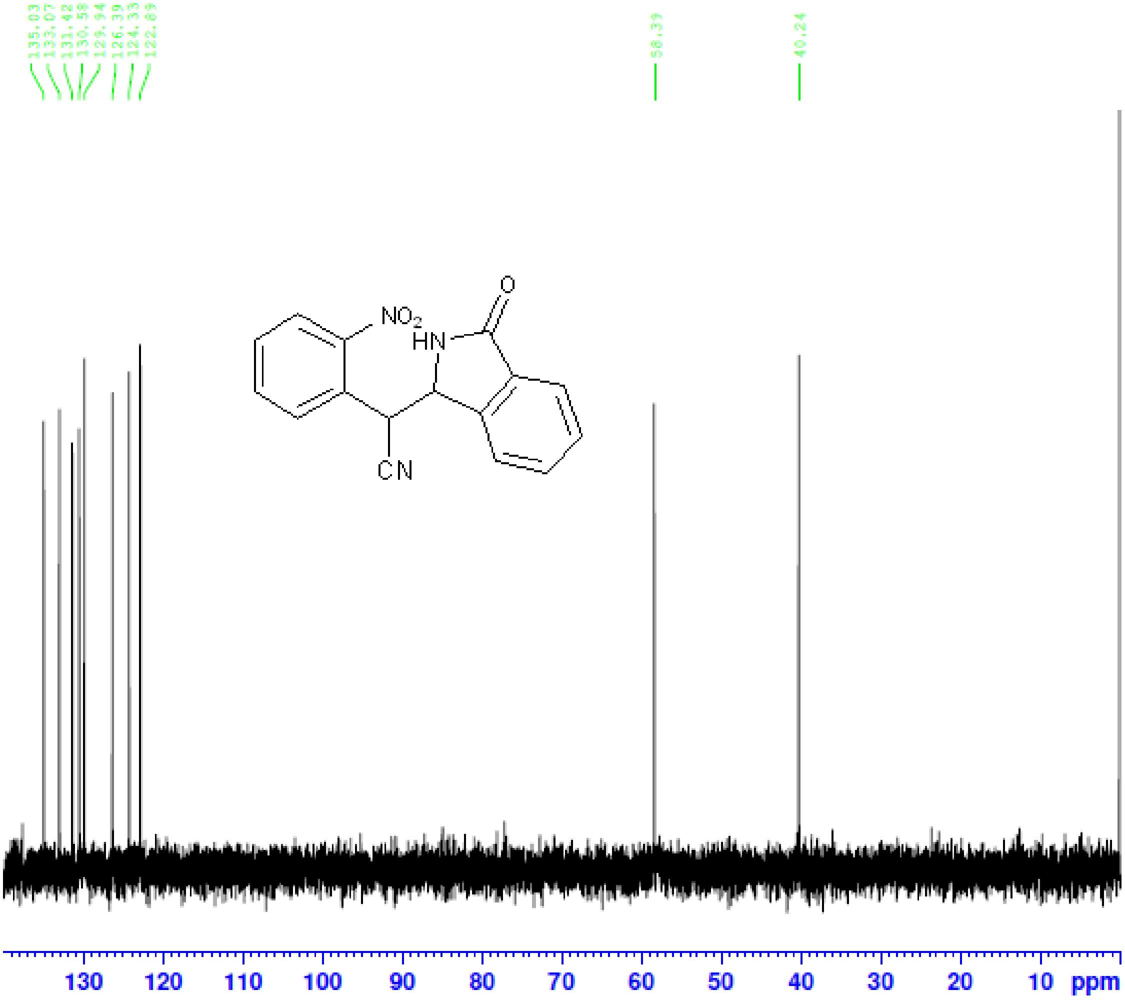

^{13}C DEPT 135-NMR of **6** in $CDCl_3$ at room temperature.

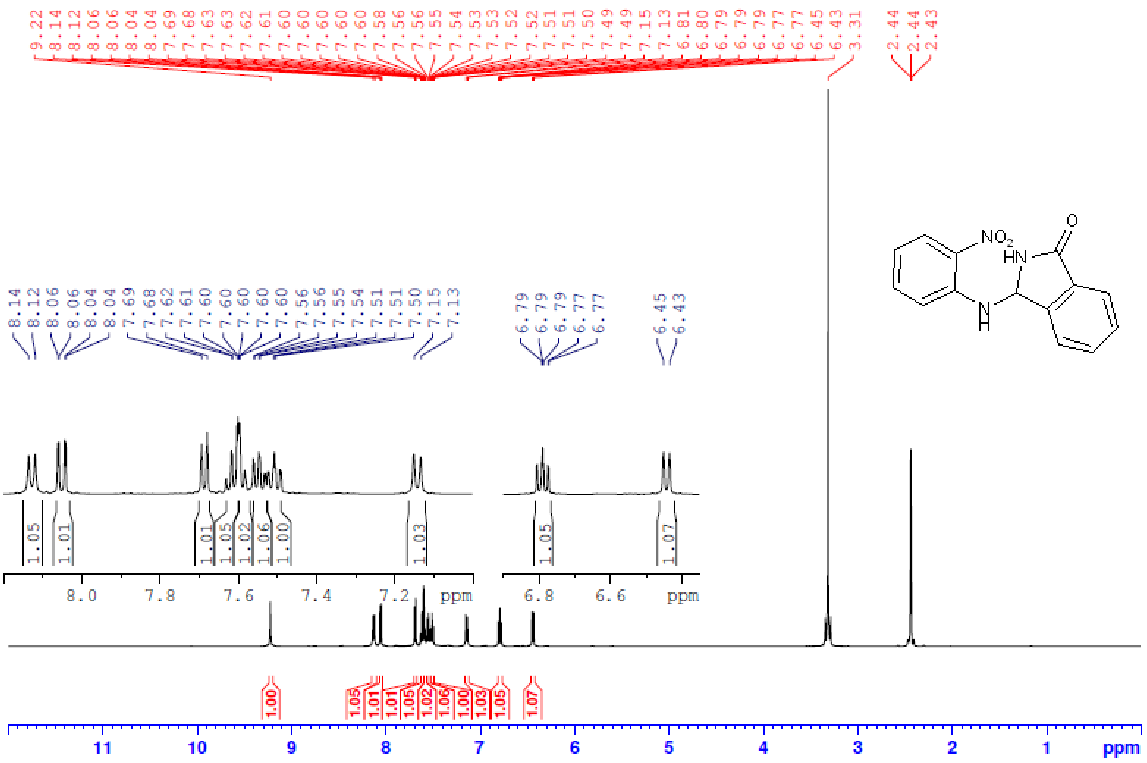

^{1}H-NMR of **10a** in DMSO-*d6* at room temperature.

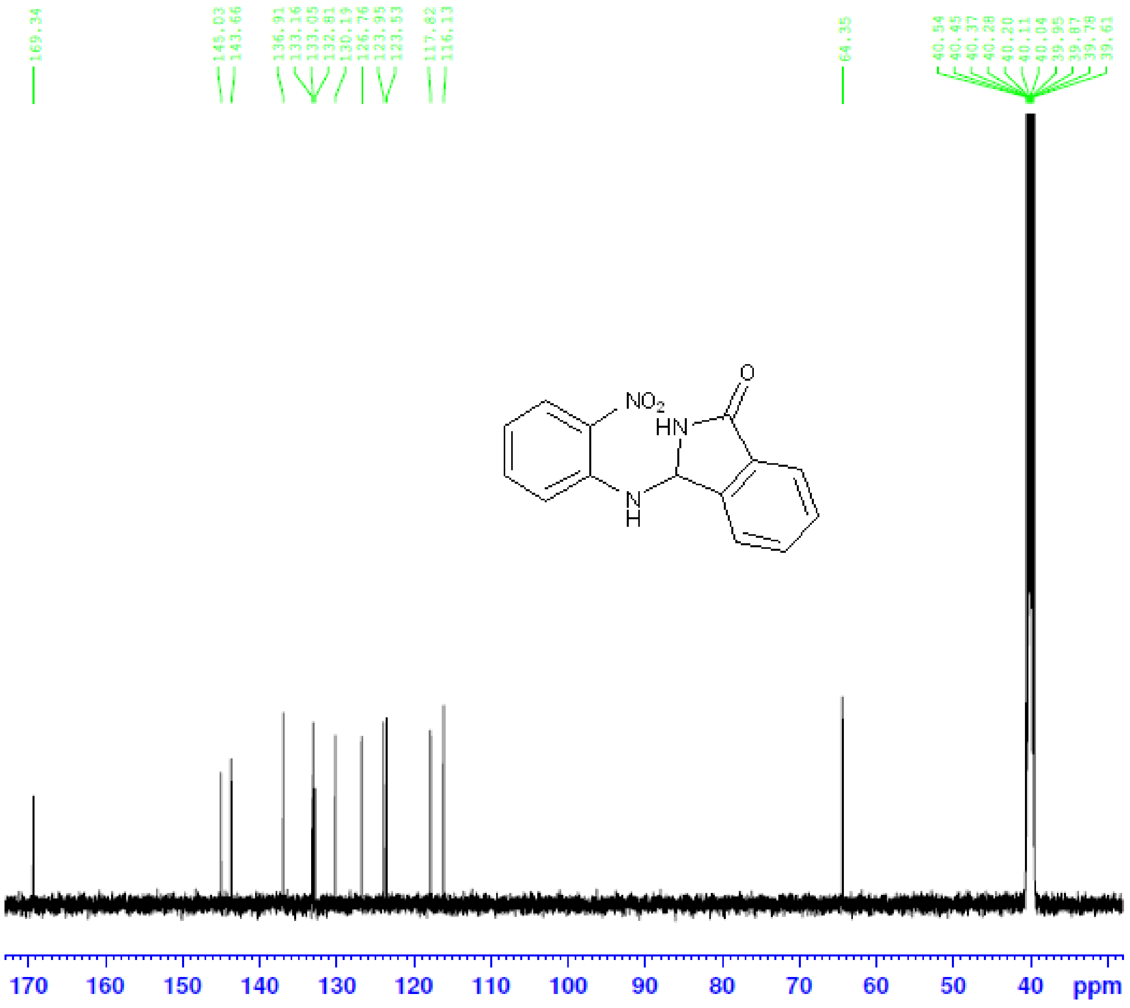

^{13}C-NMR of **10a** in DMSO-*d6* at room temperature.

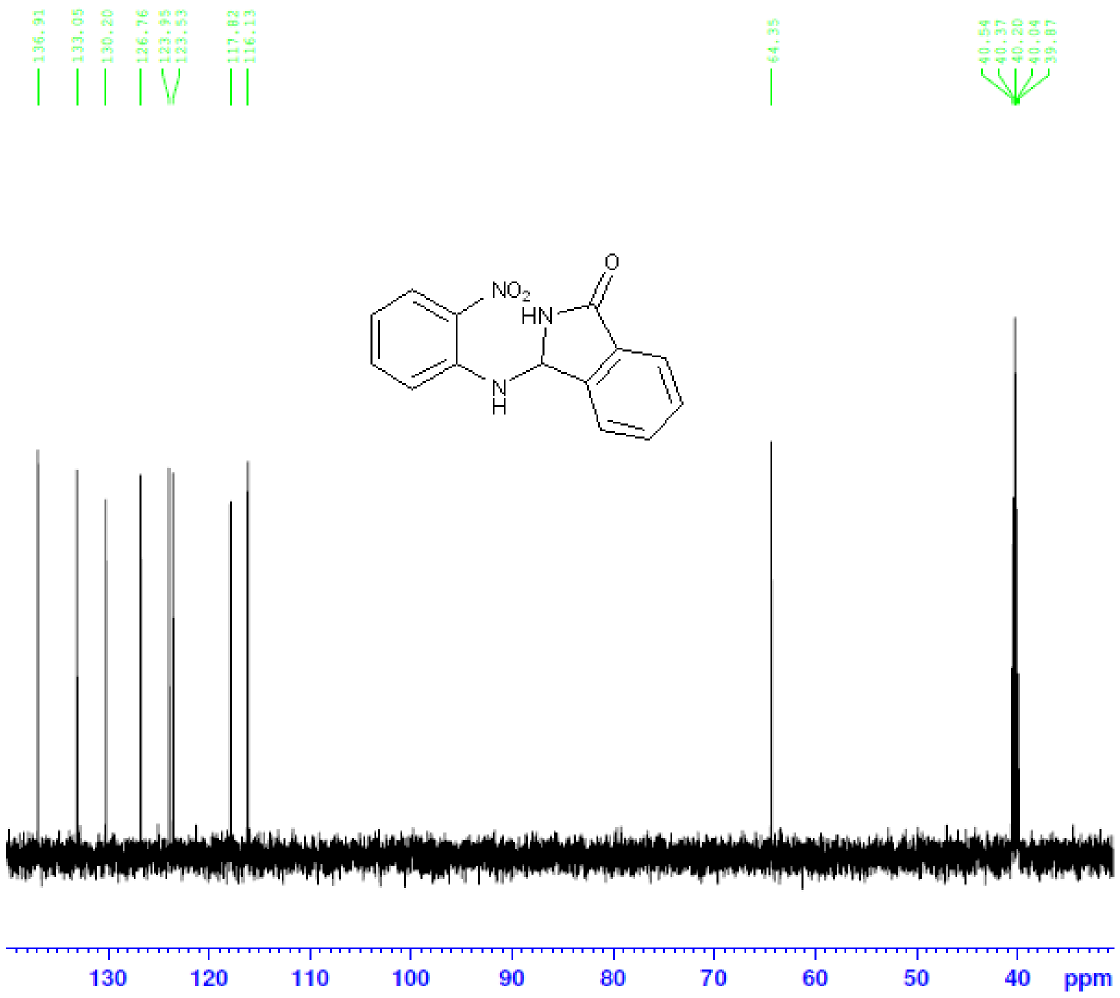

^{13}C DEPT 135-NMR of **10a** in DMSO-*d6* at room temperature.

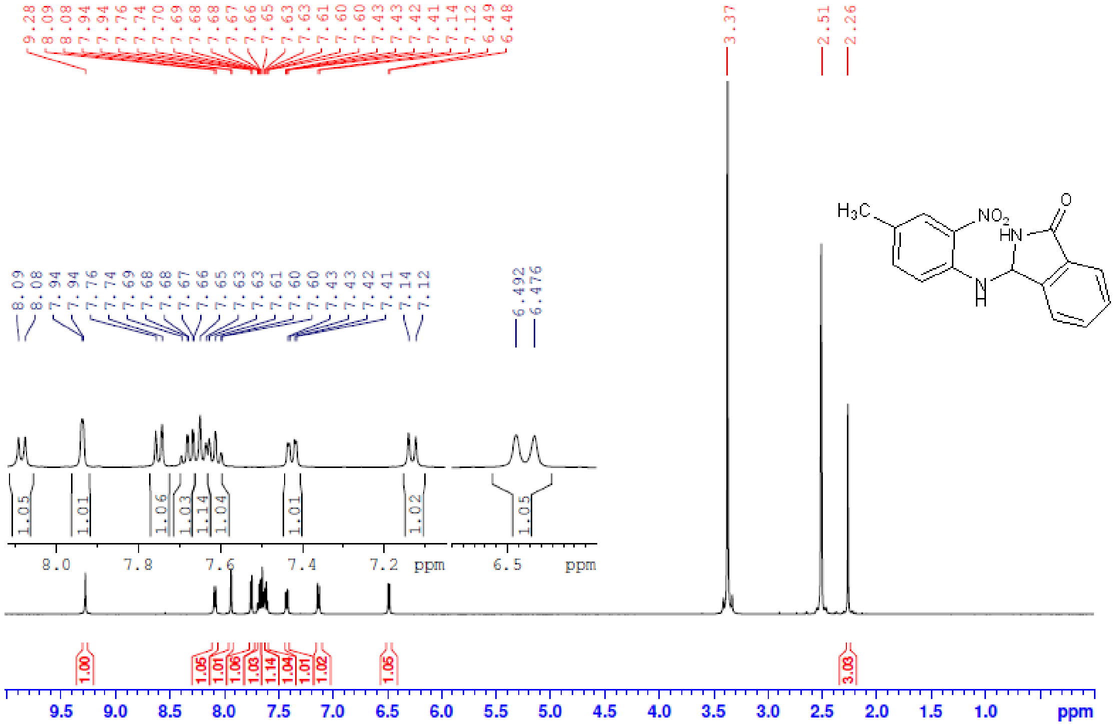

^{1}H-NMR of **10b** in DMSO-*d6* at room temperature.

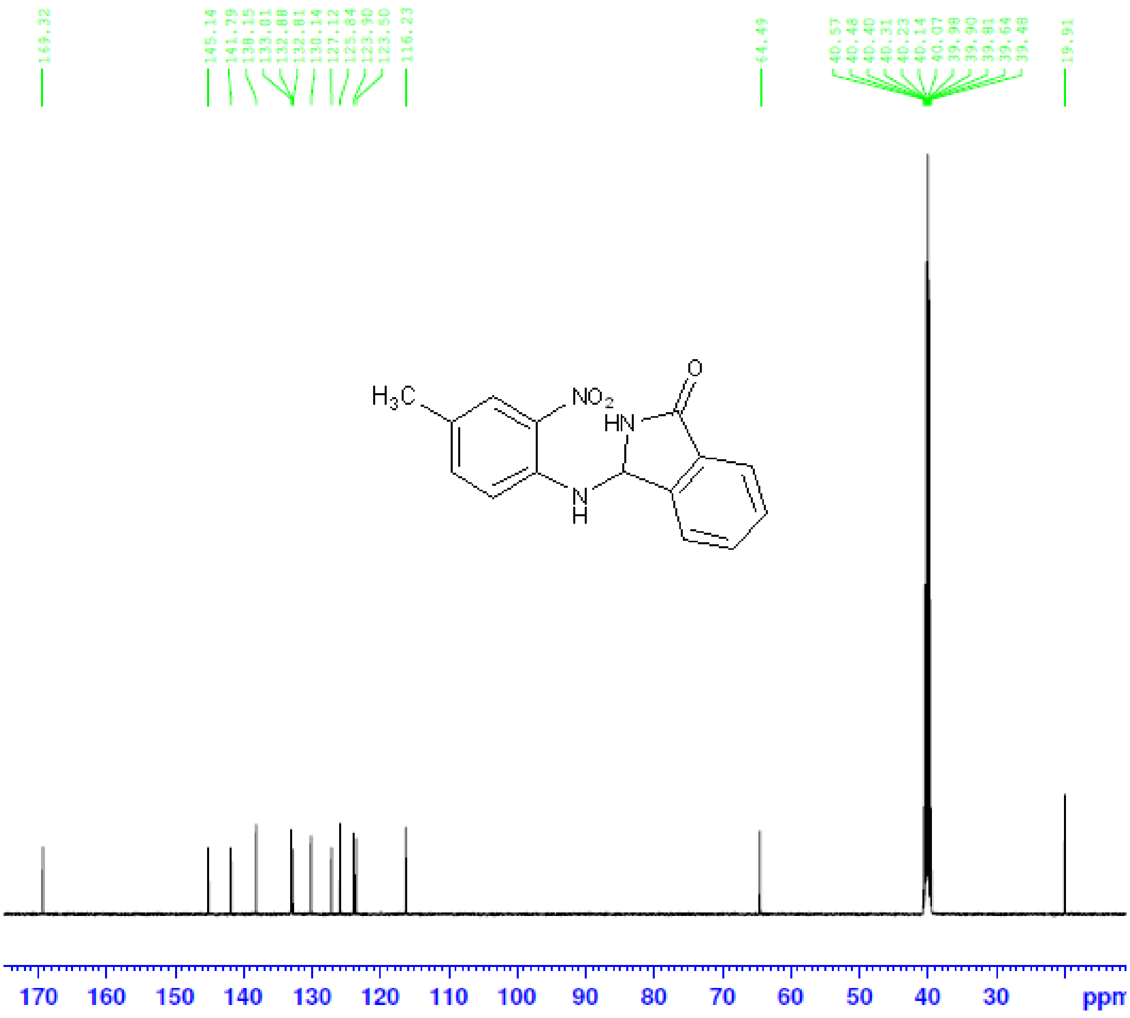

^{13}C-NMR of **10b** in DMSO-*d6* at room temperature.

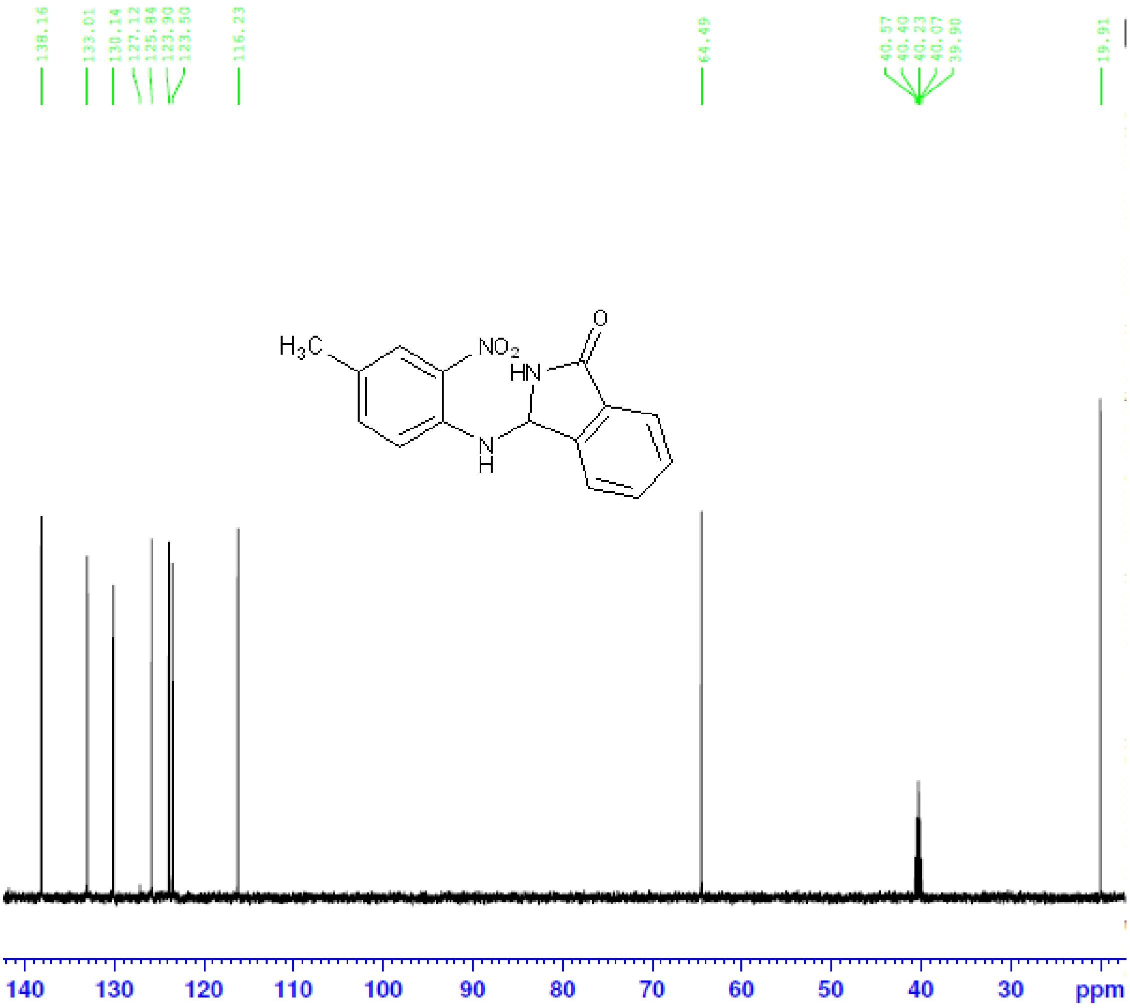

^{13}C DEPT 135-NMR of **10b** in DMSO-*d6* at room temperature.

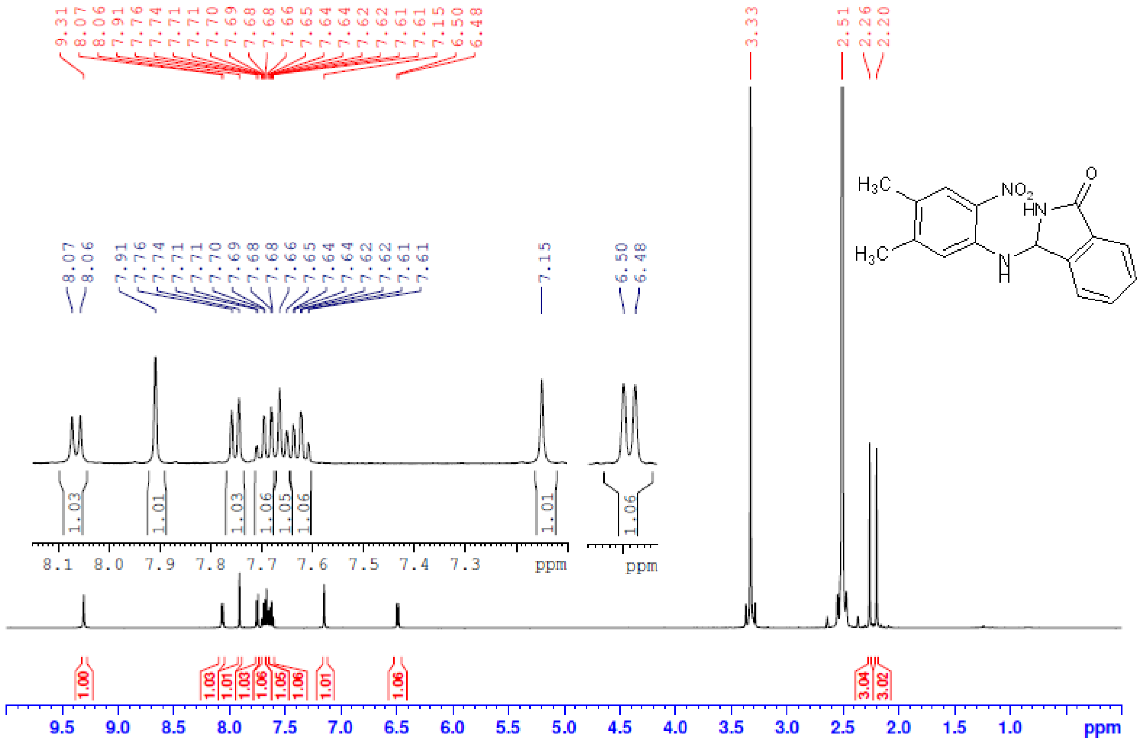

^{1}H-NMR of **10c** in DMSO-*d6* at room temperature.

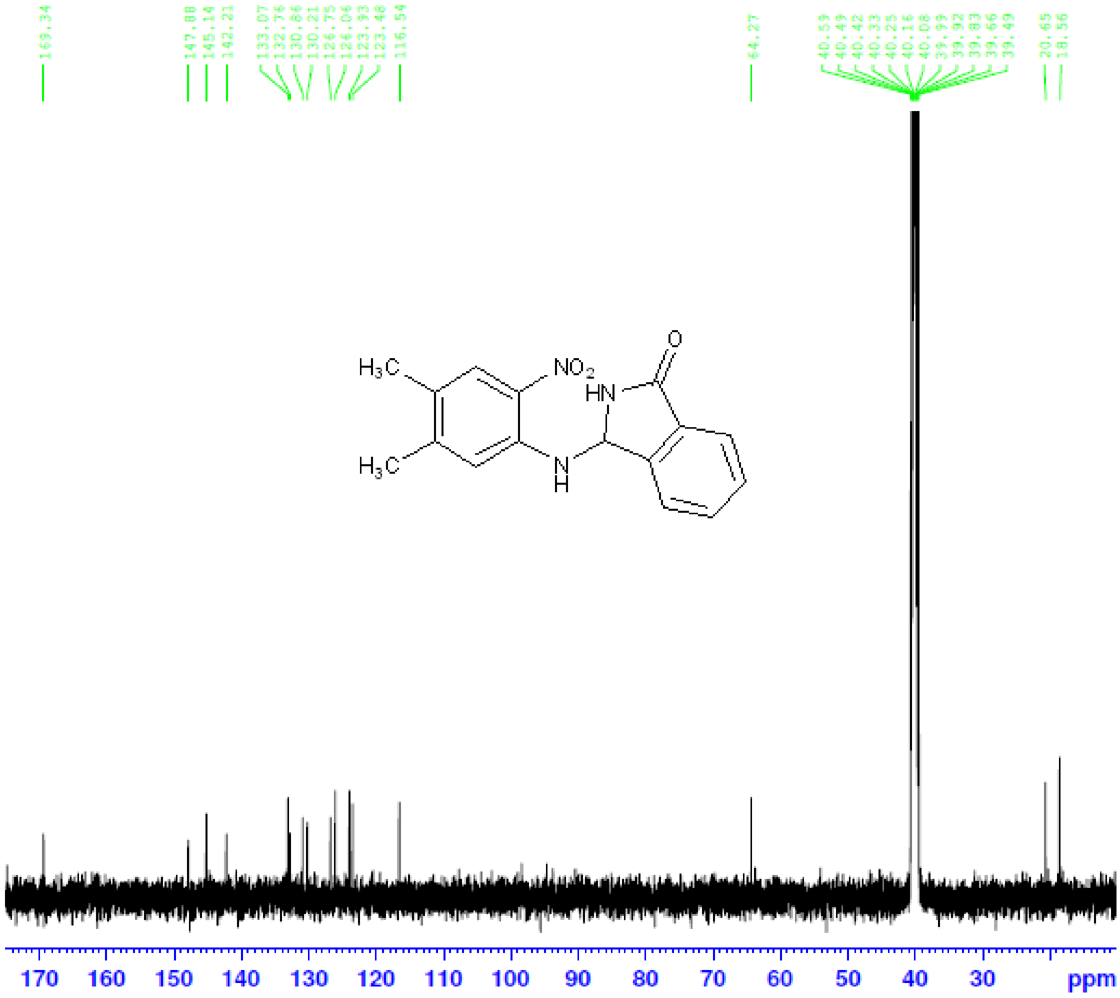

^{13}C-NMR of **10c** in DMSO-*d6* at room temperature.

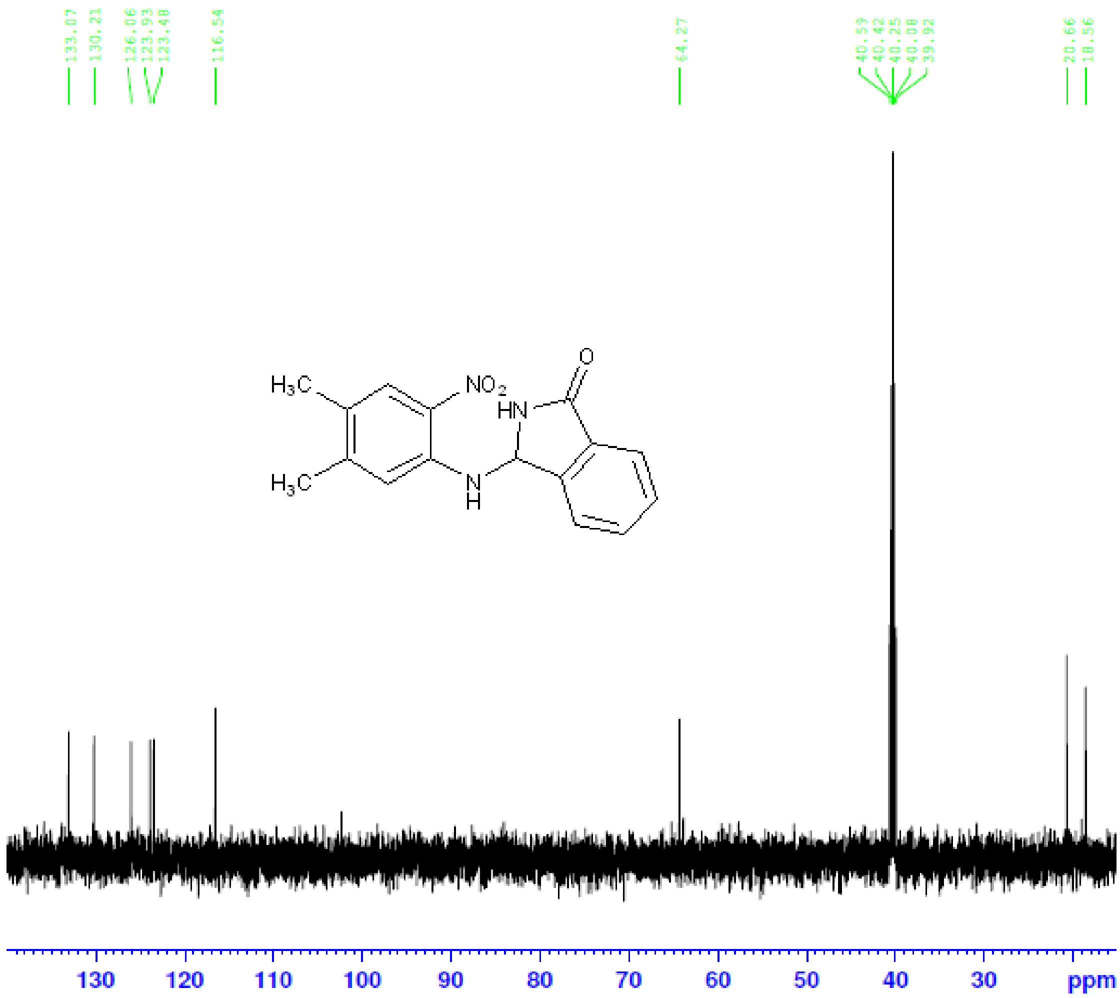

^{13}C DEPT 135-NMR of **10c** in DMSO-*d6* at room temperature.

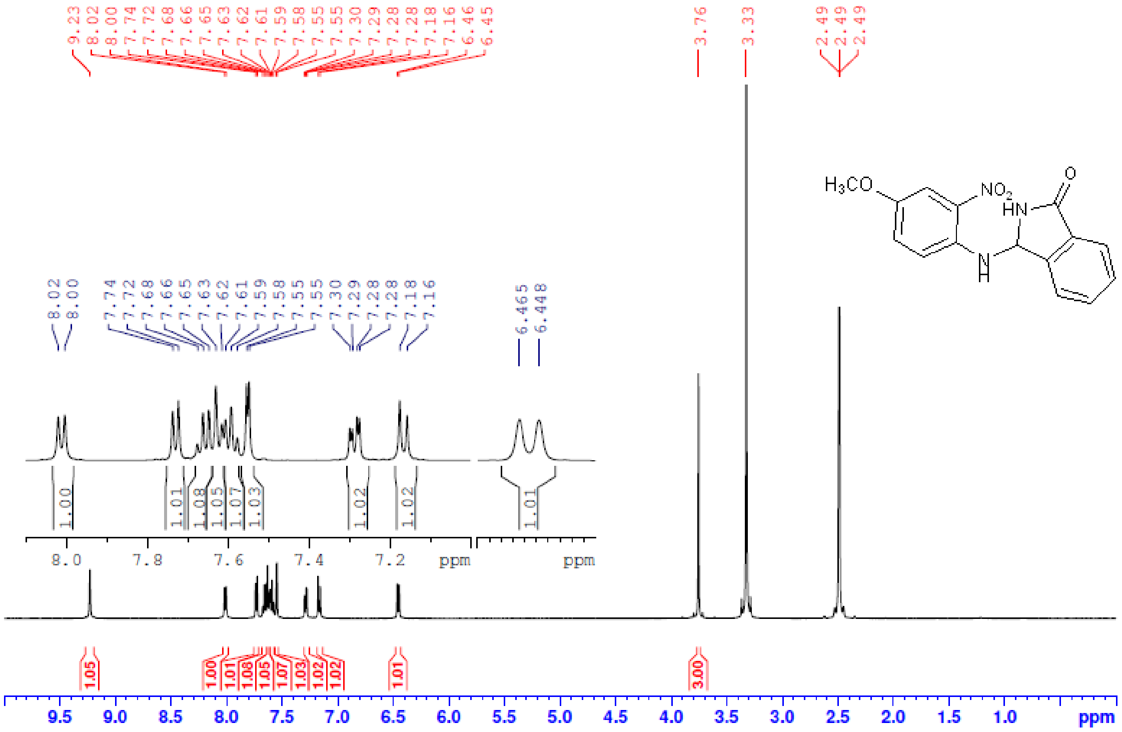

^{1}H-NMR of **10d** in DMSO-*d6* at room temperature.

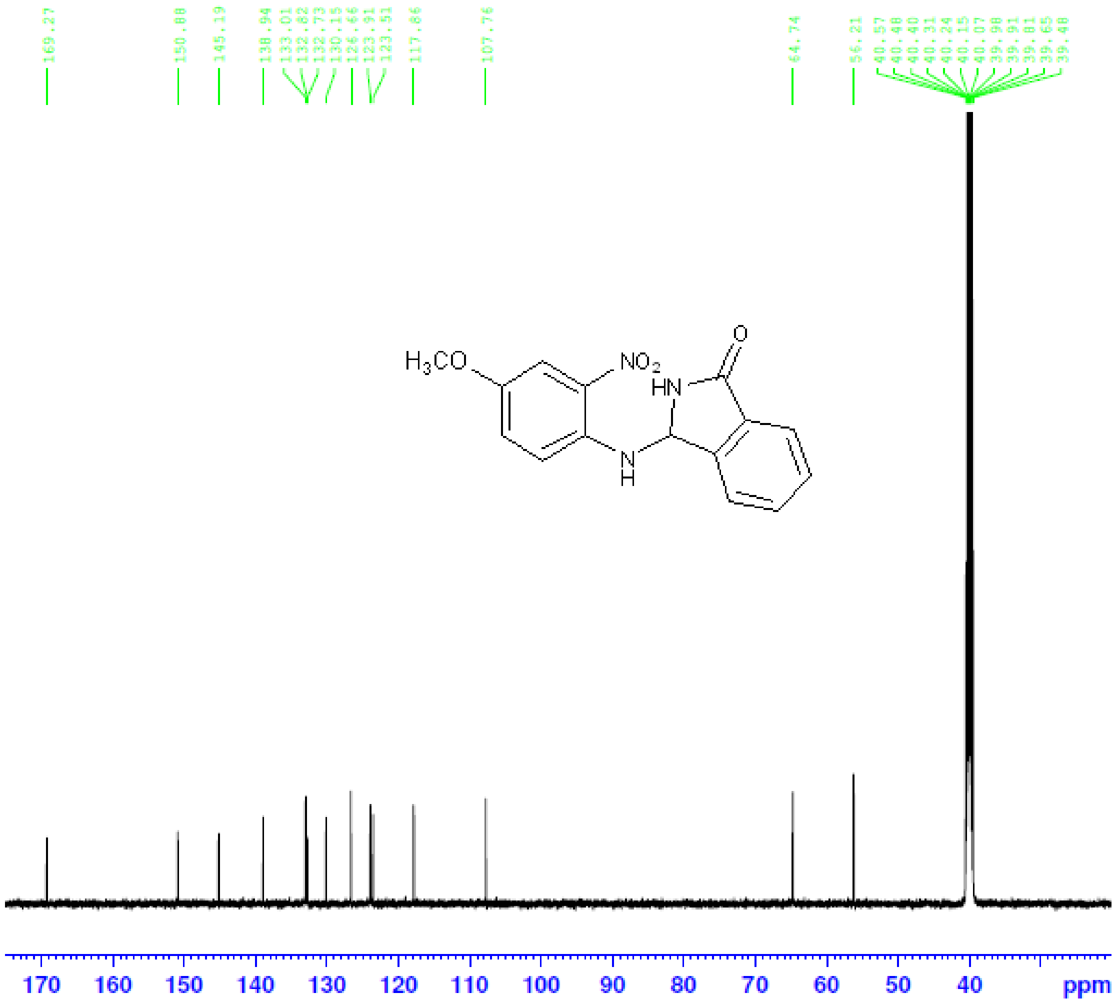

^{13}C-NMR of **10d** in DMSO-*d6* at room temperature.

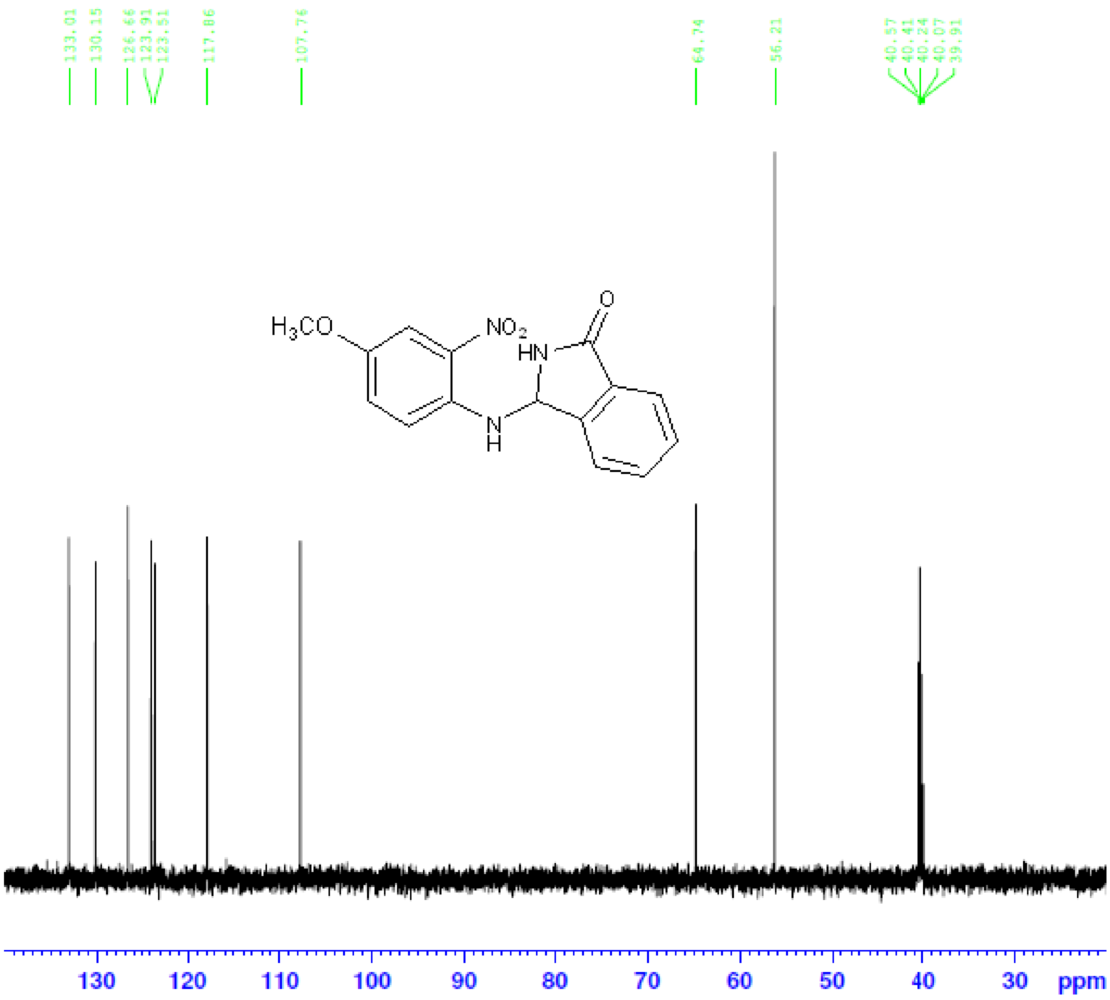

^{13}C DEPT 135-NMR of **10d** in DMSO-*d6* at room temperature.

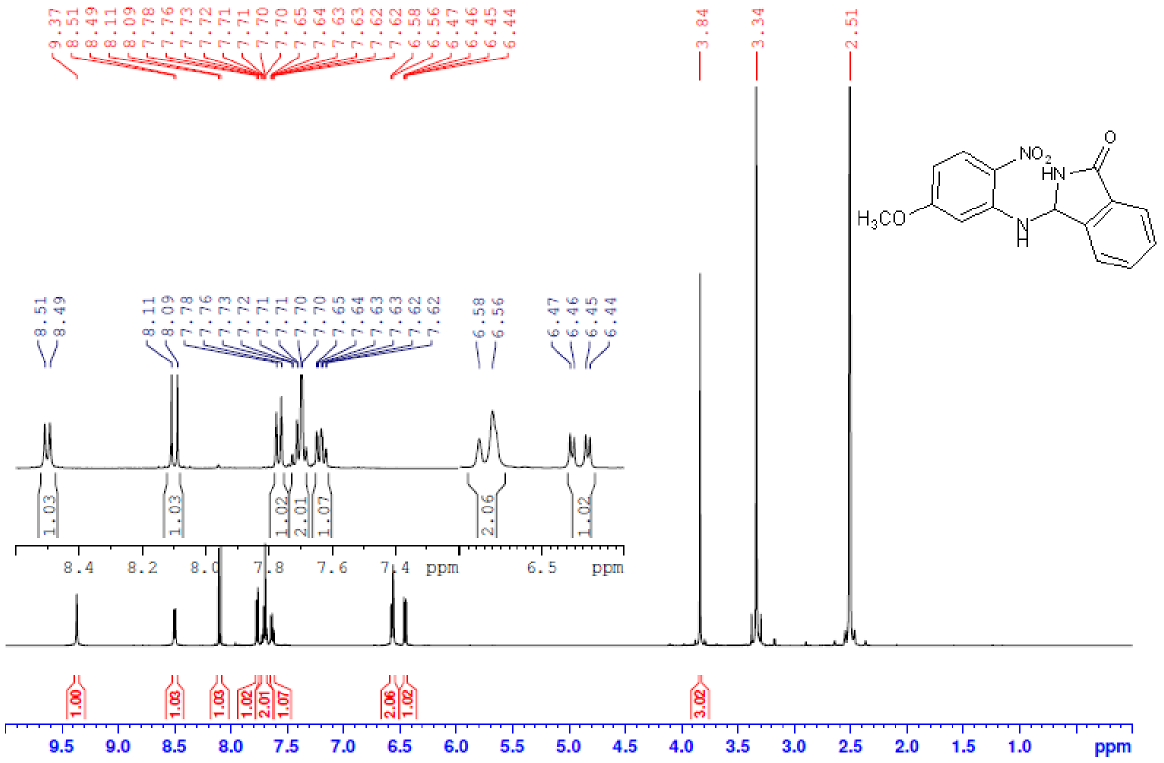

^{1}H-NMR of **10e** in DMSO-*d6* at room temperature.

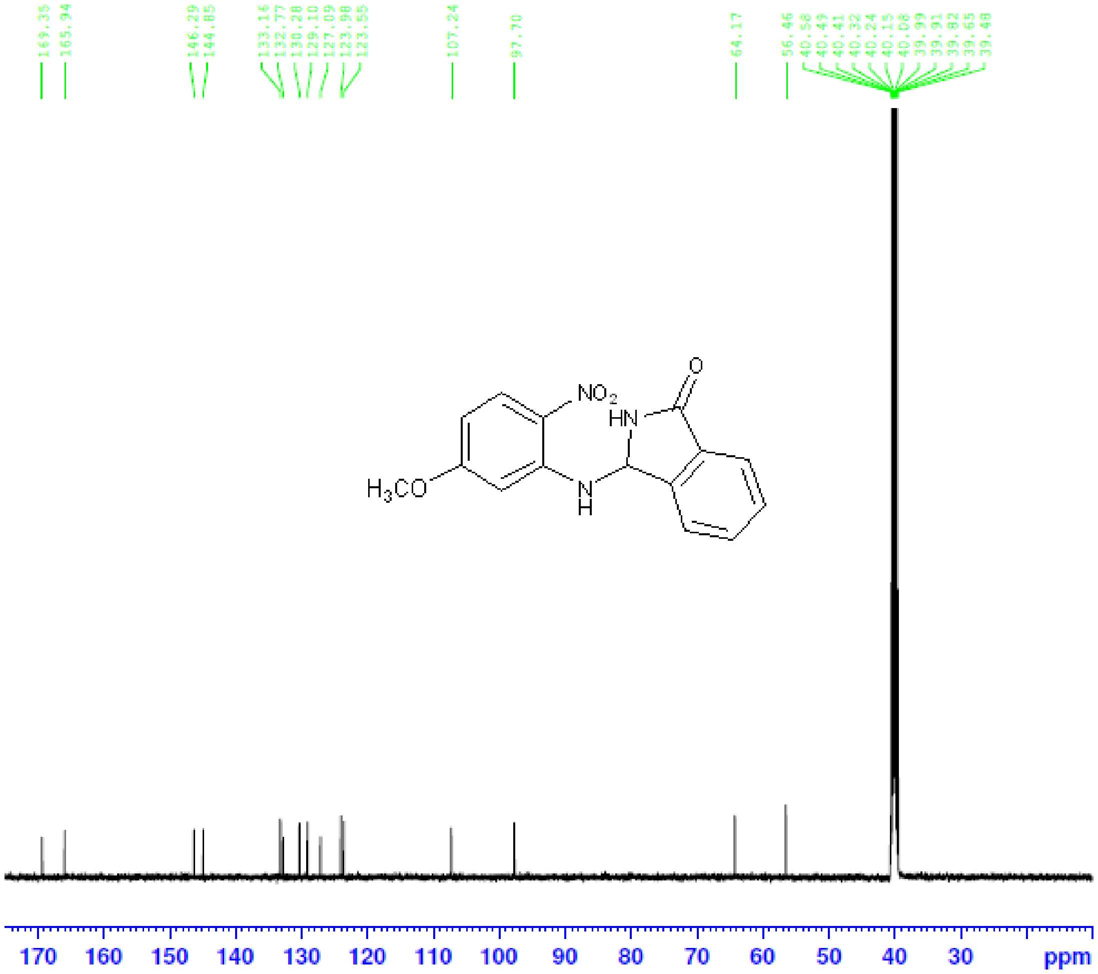

^{13}C-NMR of **10e** in DMSO-*d6* at room temperature.

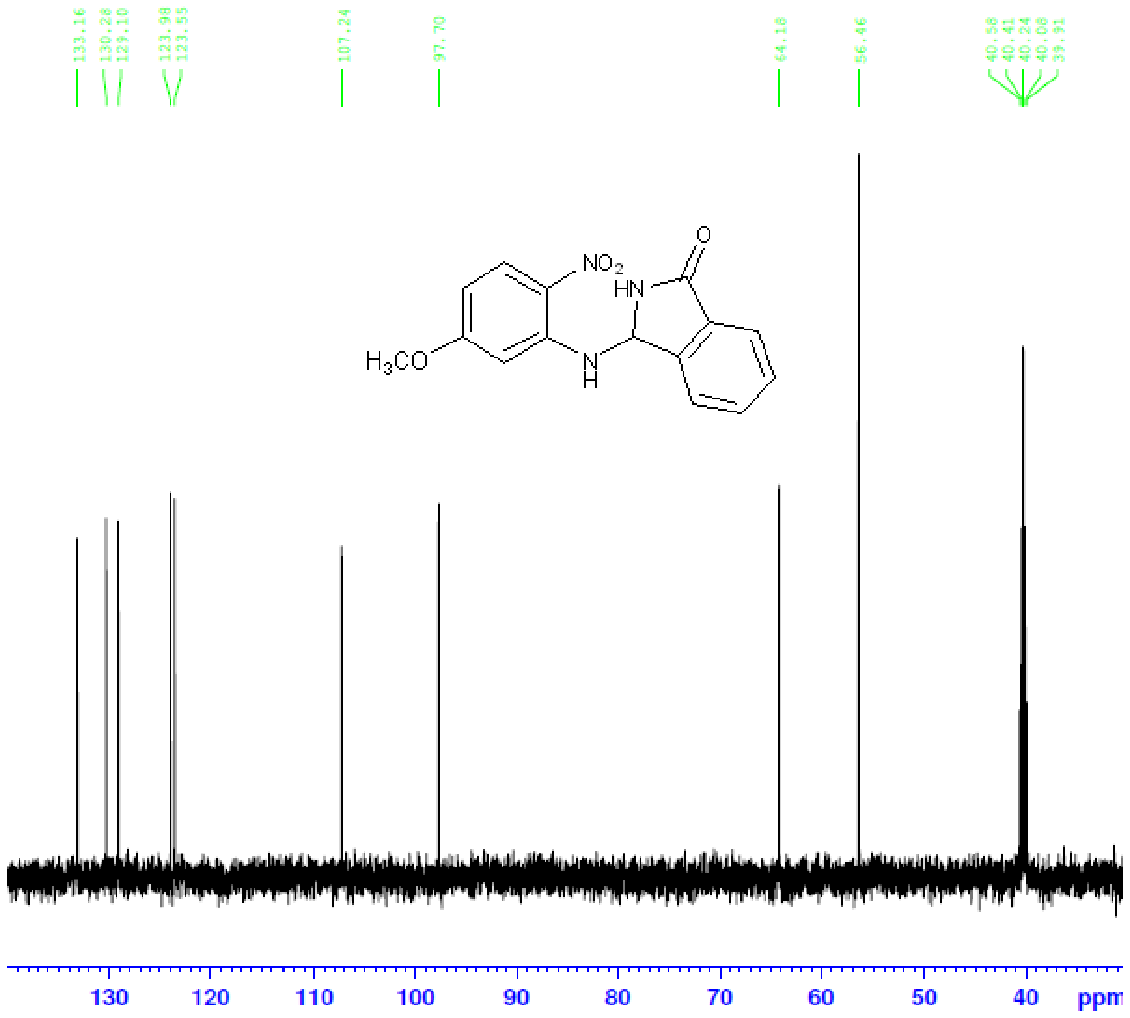

^{13}C DEPT 135-NMR of **10e** in DMSO-*d6* at room temperature.

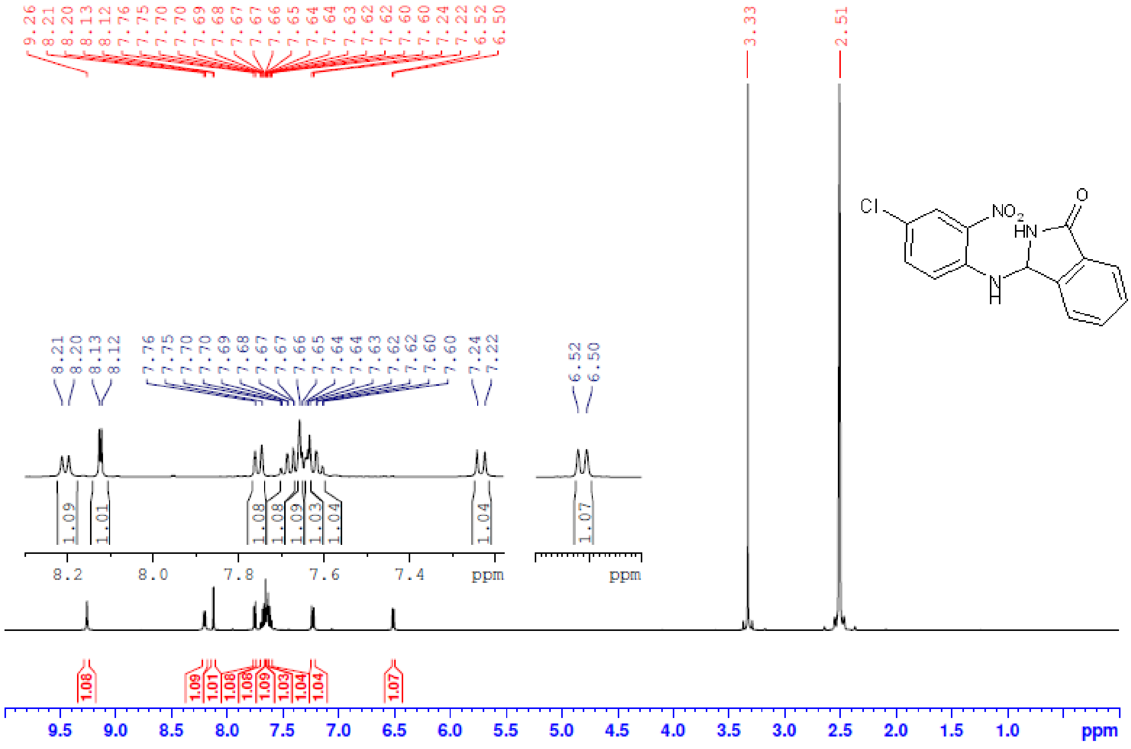

^{1}H-NMR of **10f** in DMSO-*d6* at room temperature.

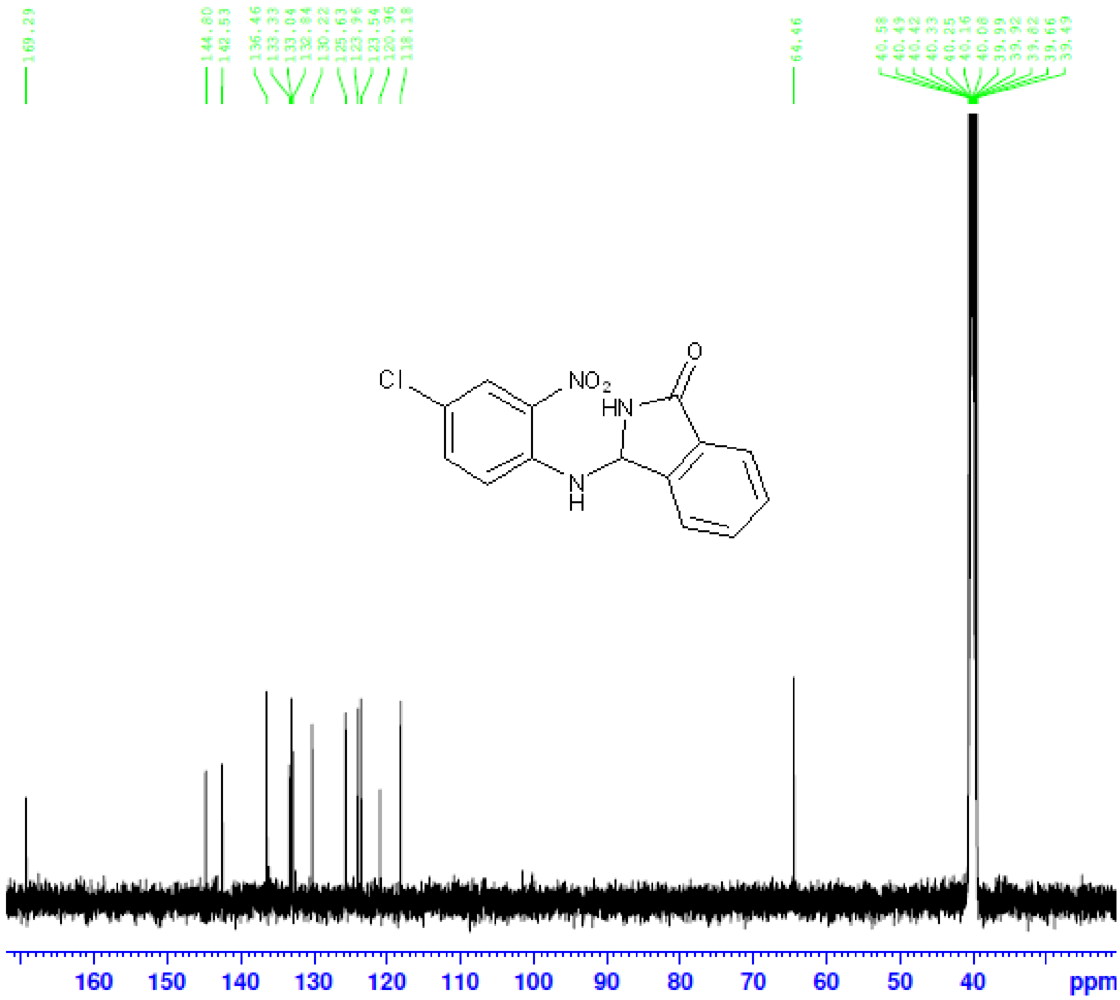

^{13}C-NMR of **10f** in DMSO-*d6* at room temperature.

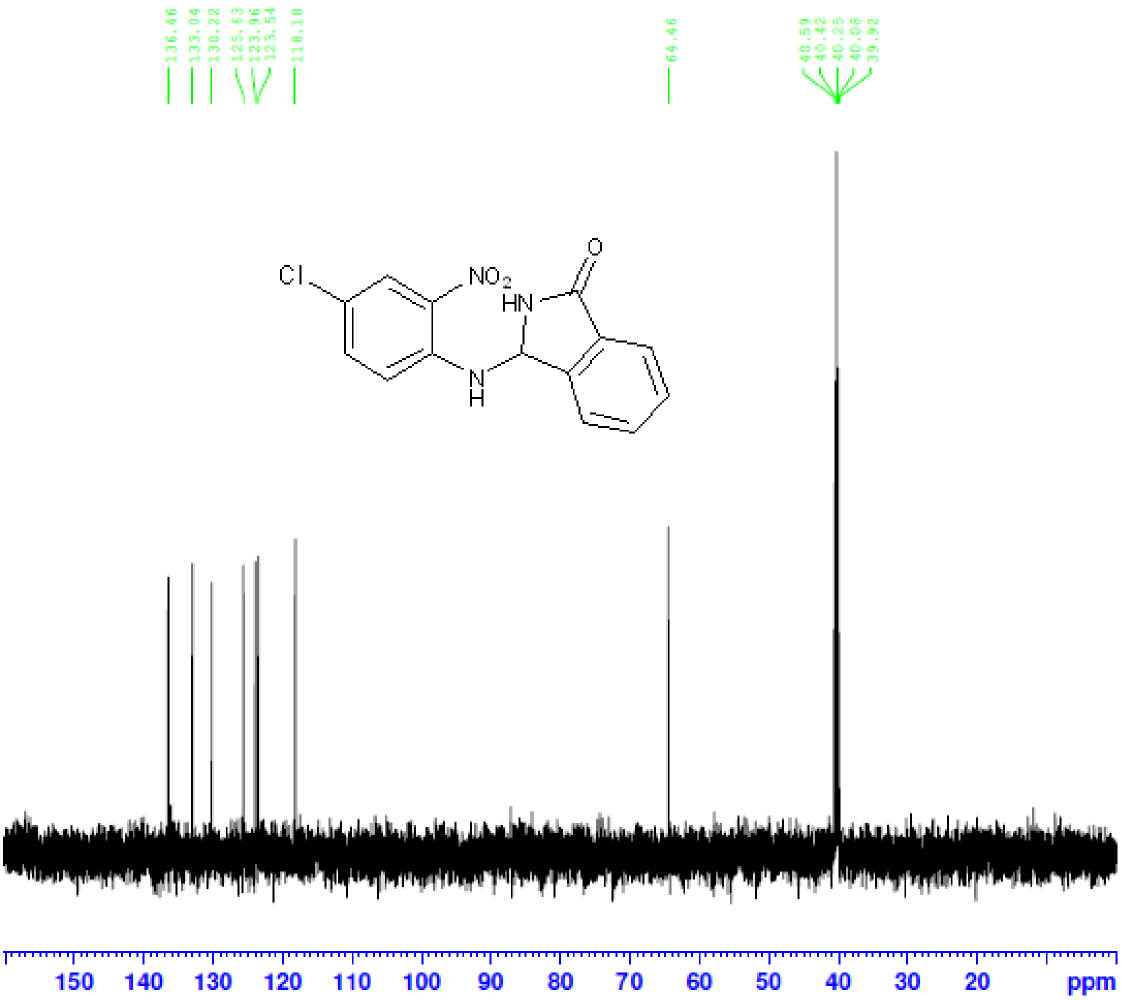

^{13}C DEPT 135-NMR of **10f** in DMSO-*d6* at room temperature.

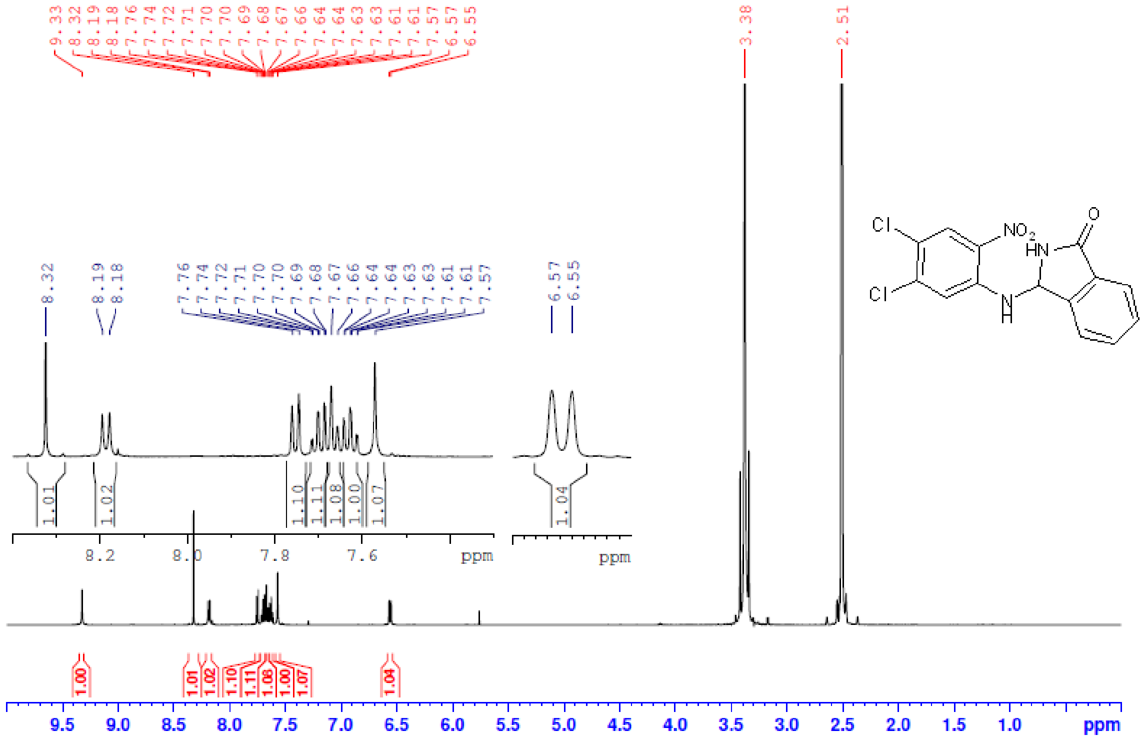

^{1}H-NMR of **10g** in DMSO-*d6* at room temperature.

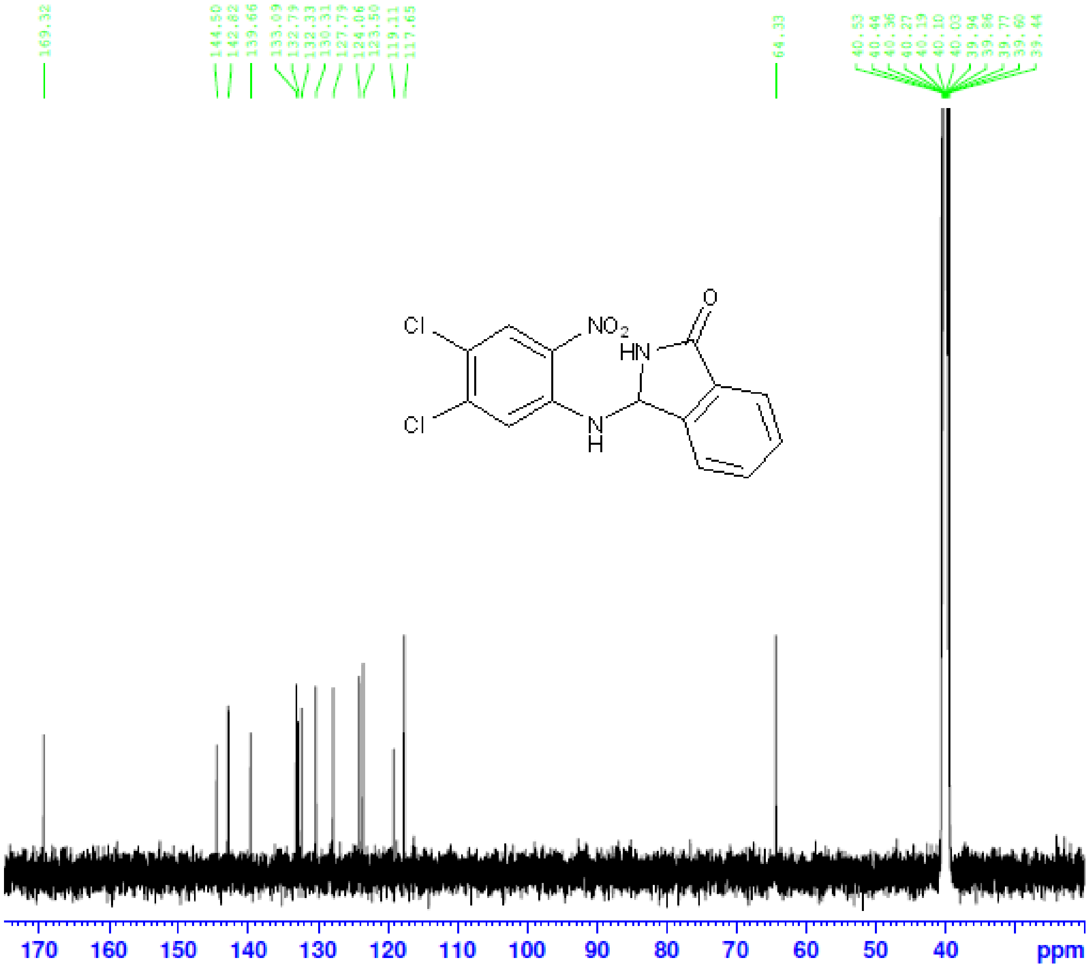

^{13}C-NMR of **10g** in DMSO-*d6* at room temperature.

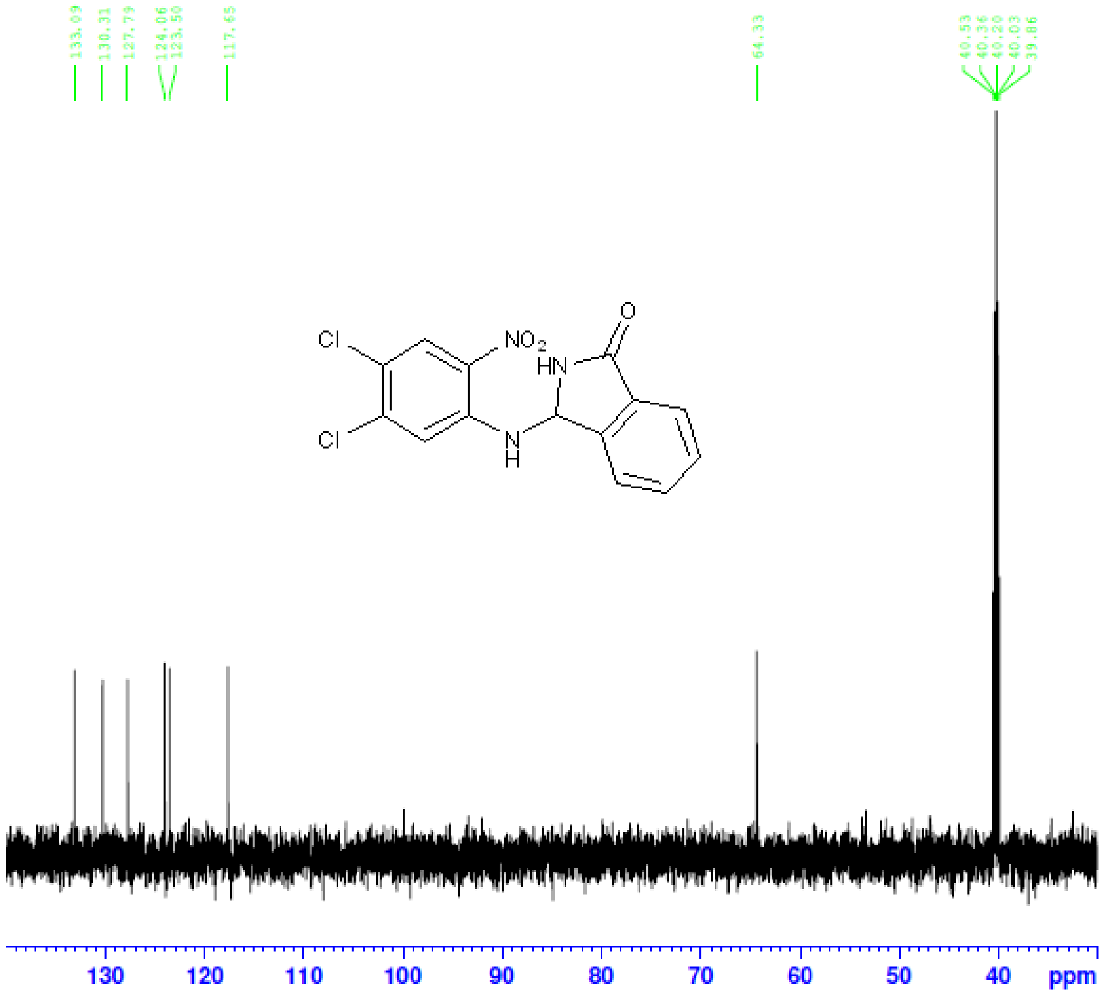

^{13}C DEPT 135-NMR of **10g** in DMSO-*d6* at room temperature.

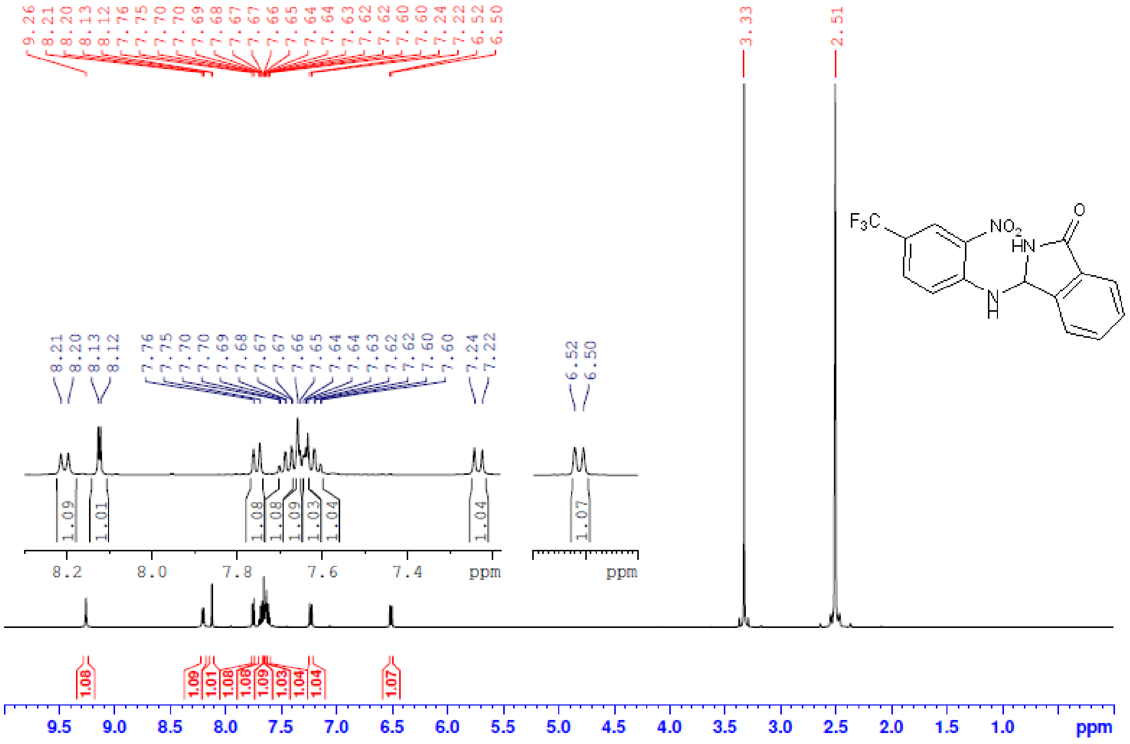

^{1}H-NMR of **10h** in DMSO-*d6* at room temperature.

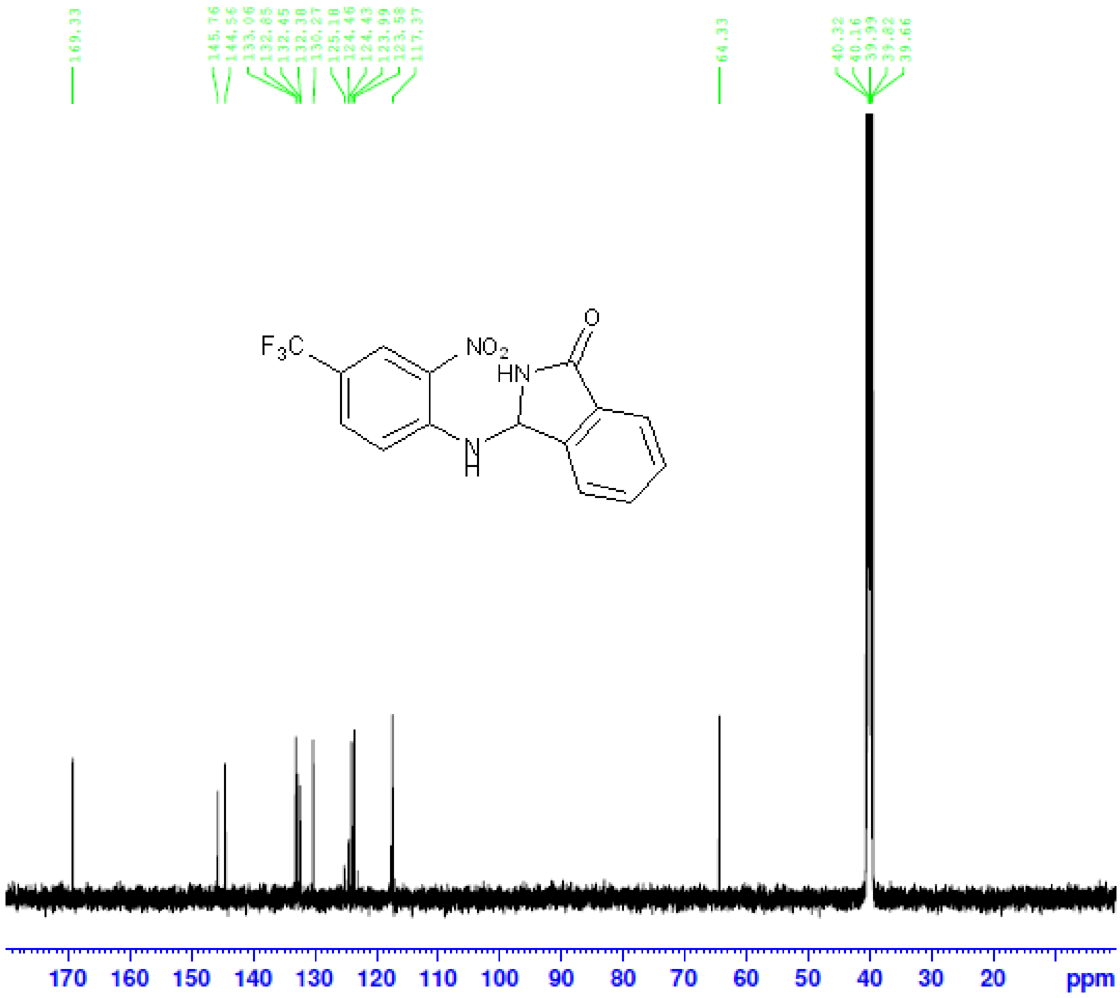

^{13}C-NMR of **10h** in DMSO-*d6* at room temperature.

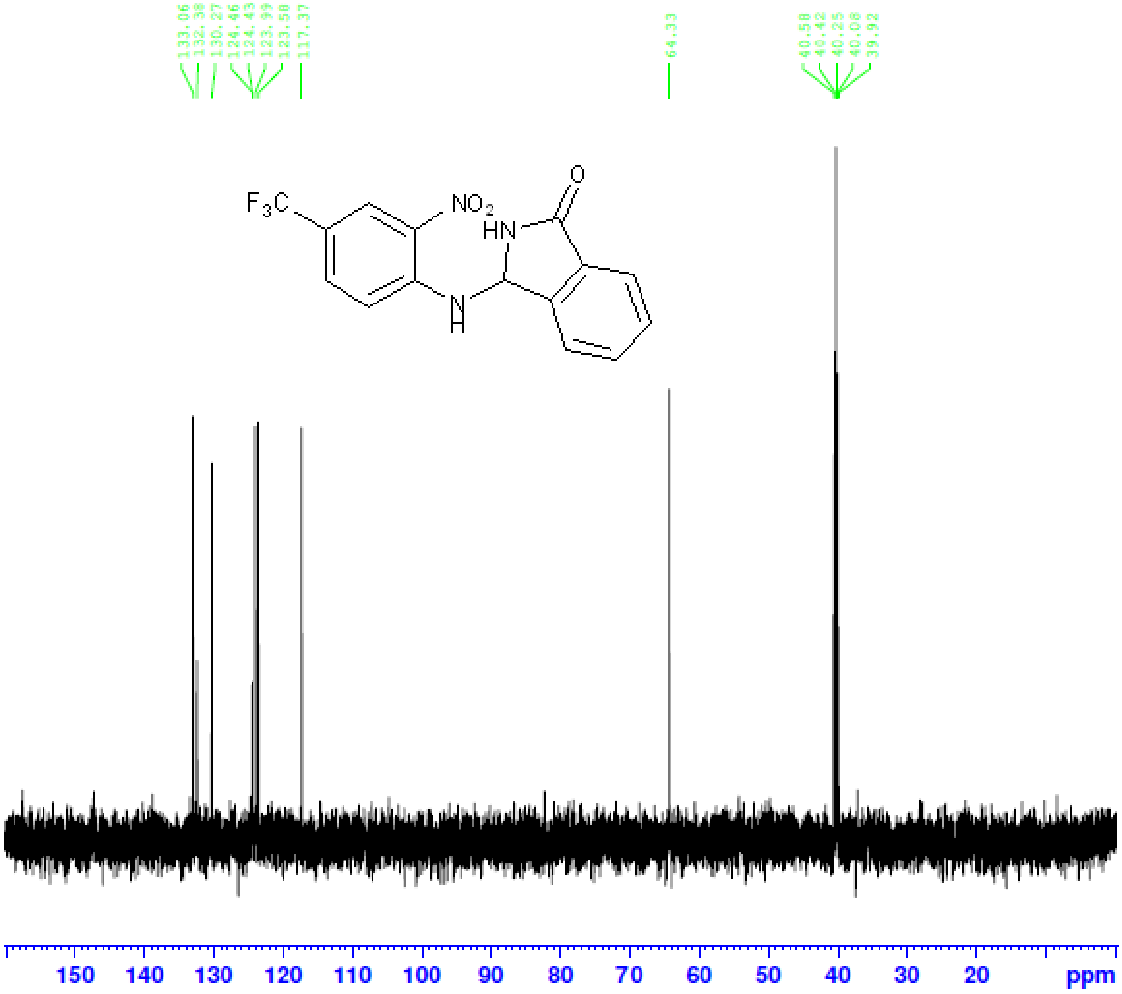

^{13}C DEPT 135-NMR of **10h** in DMSO-*d6* at room temperature.

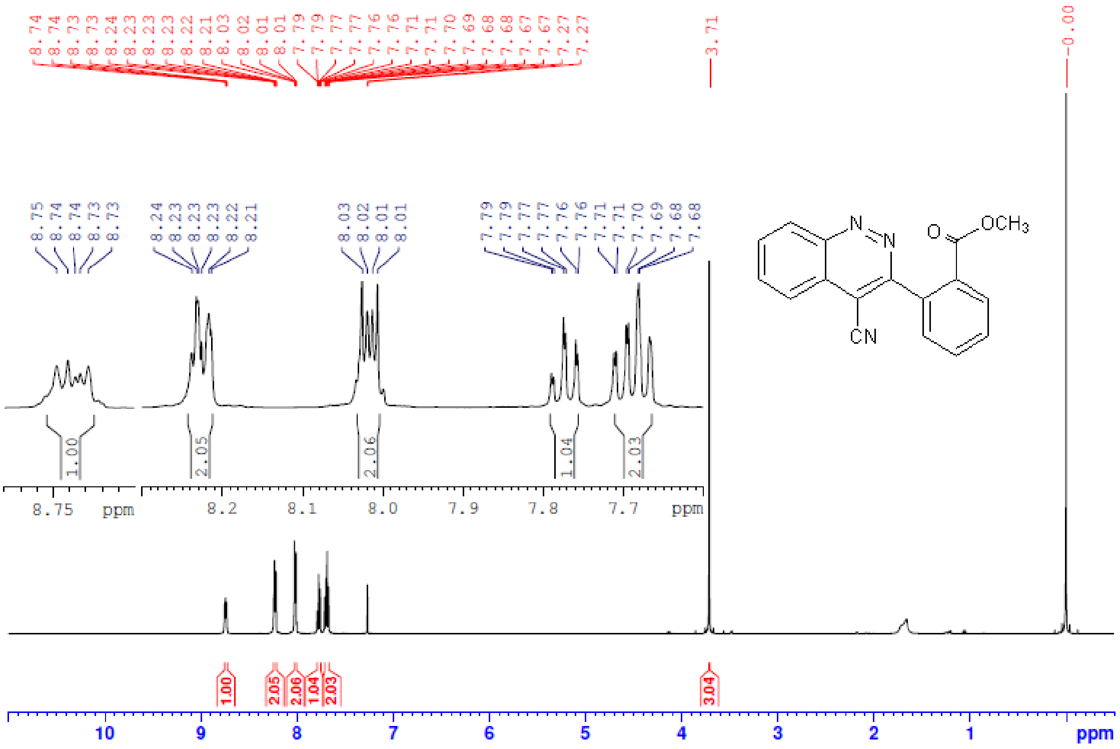

^{1}H-NMR of **14** in $CDCl_3$ at room temperature.

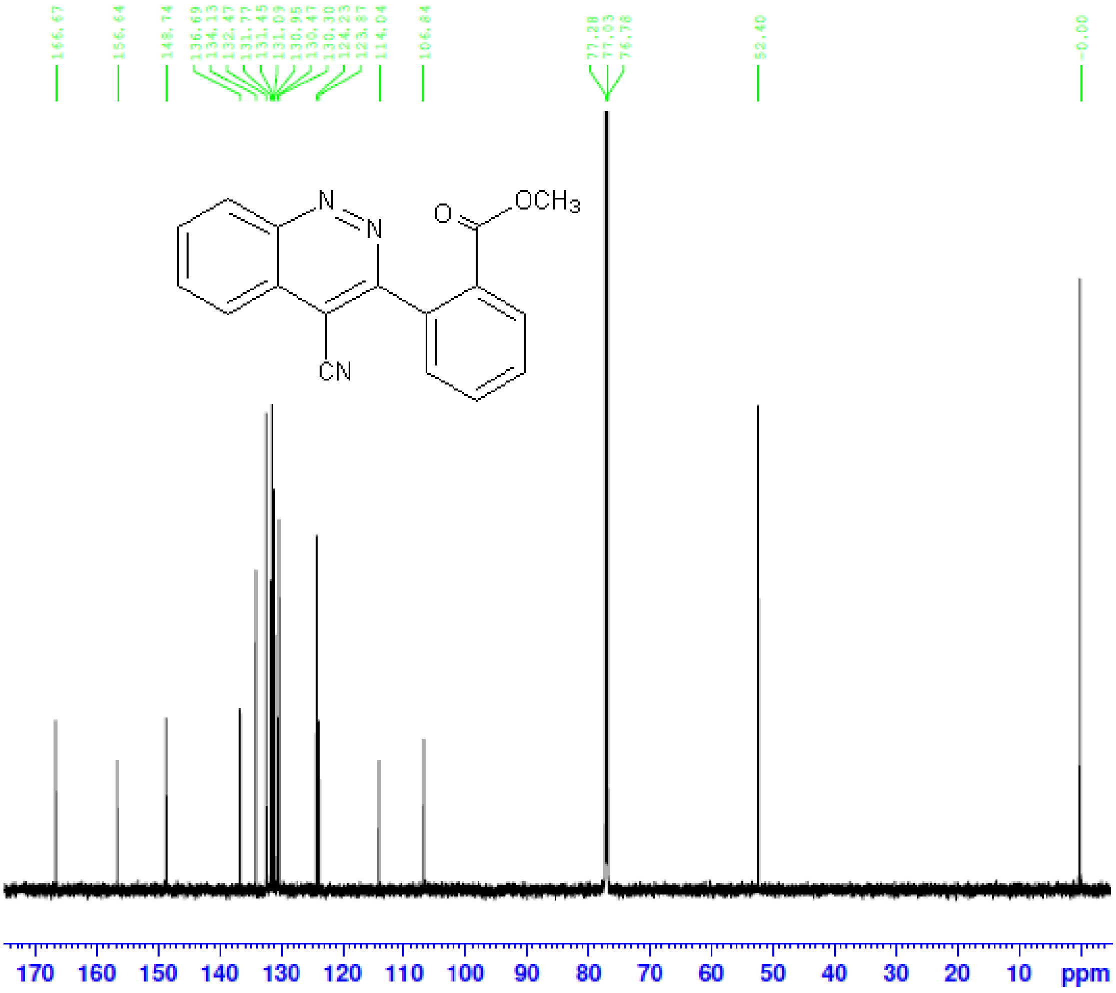

^{13}C-NMR of **14** in $CDCl_3$ at room temperature.

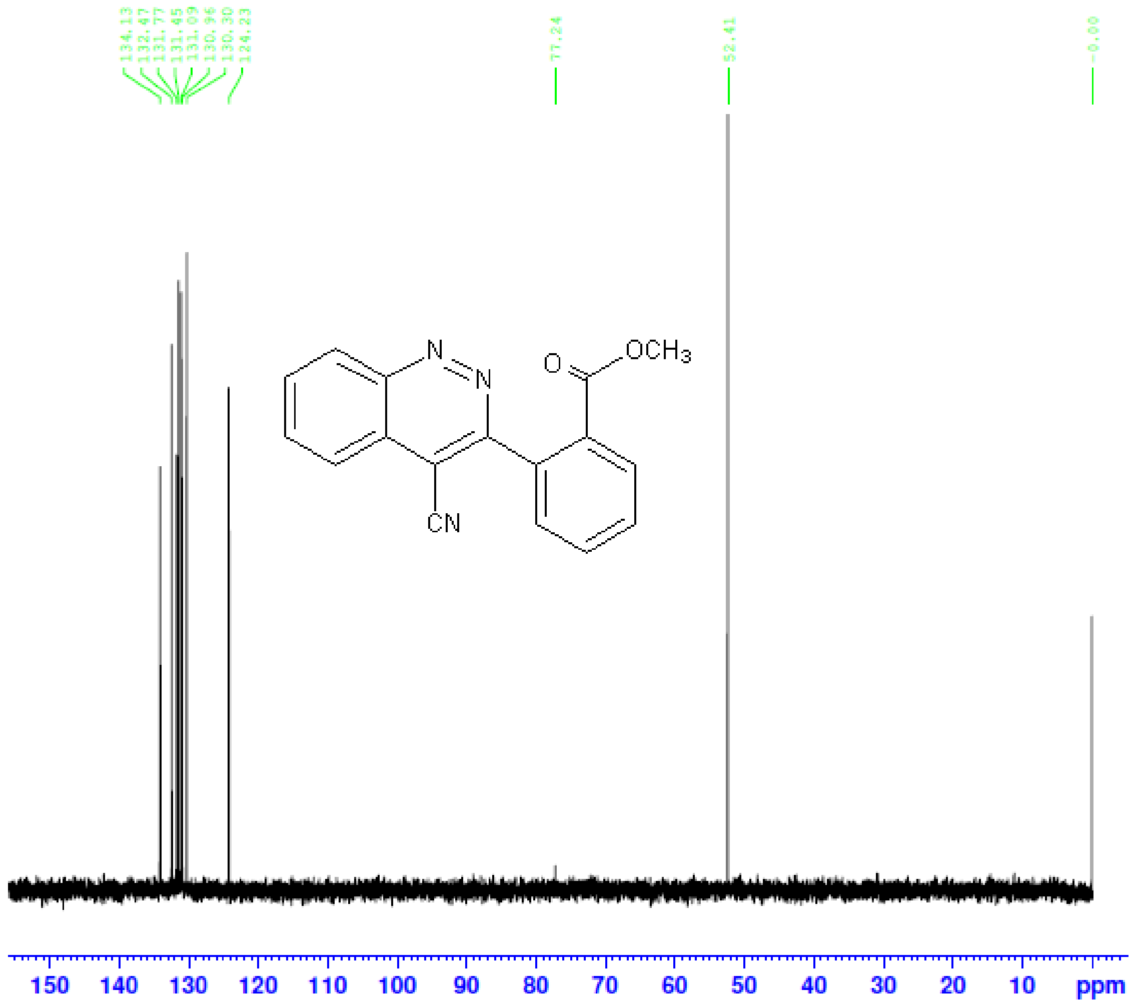

^{13}C DEPT 135-NMR of **14** in $CDCl_3$ at room temperature.

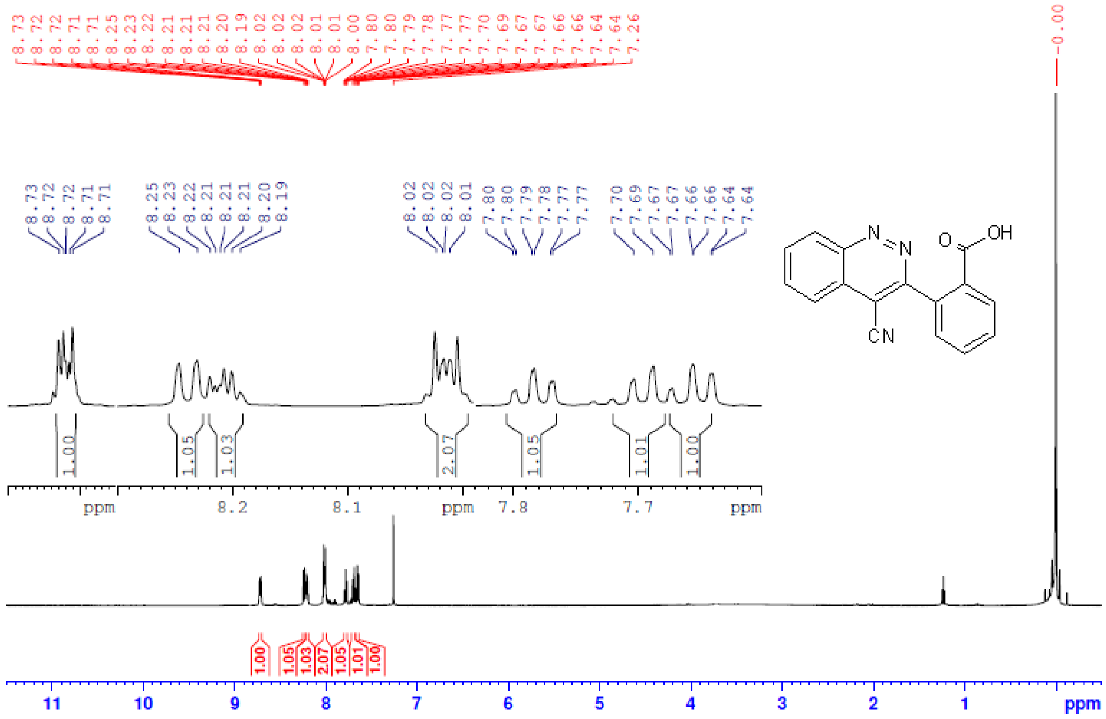

^{1}H-NMR of **15** in $CDCl_3$ at room temperature.

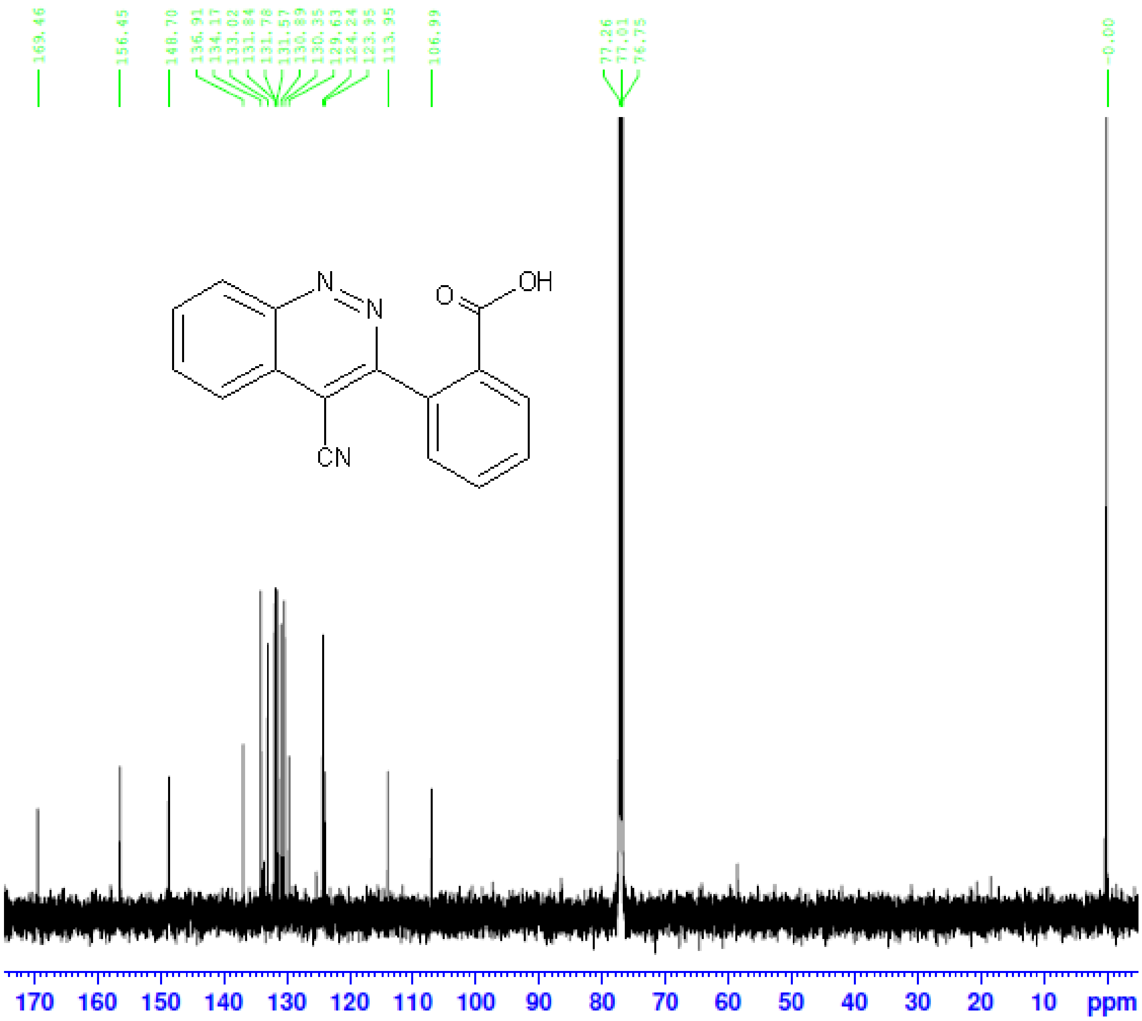

^{13}C-NMR of **15** in $CDCl_3$ at room temperature.

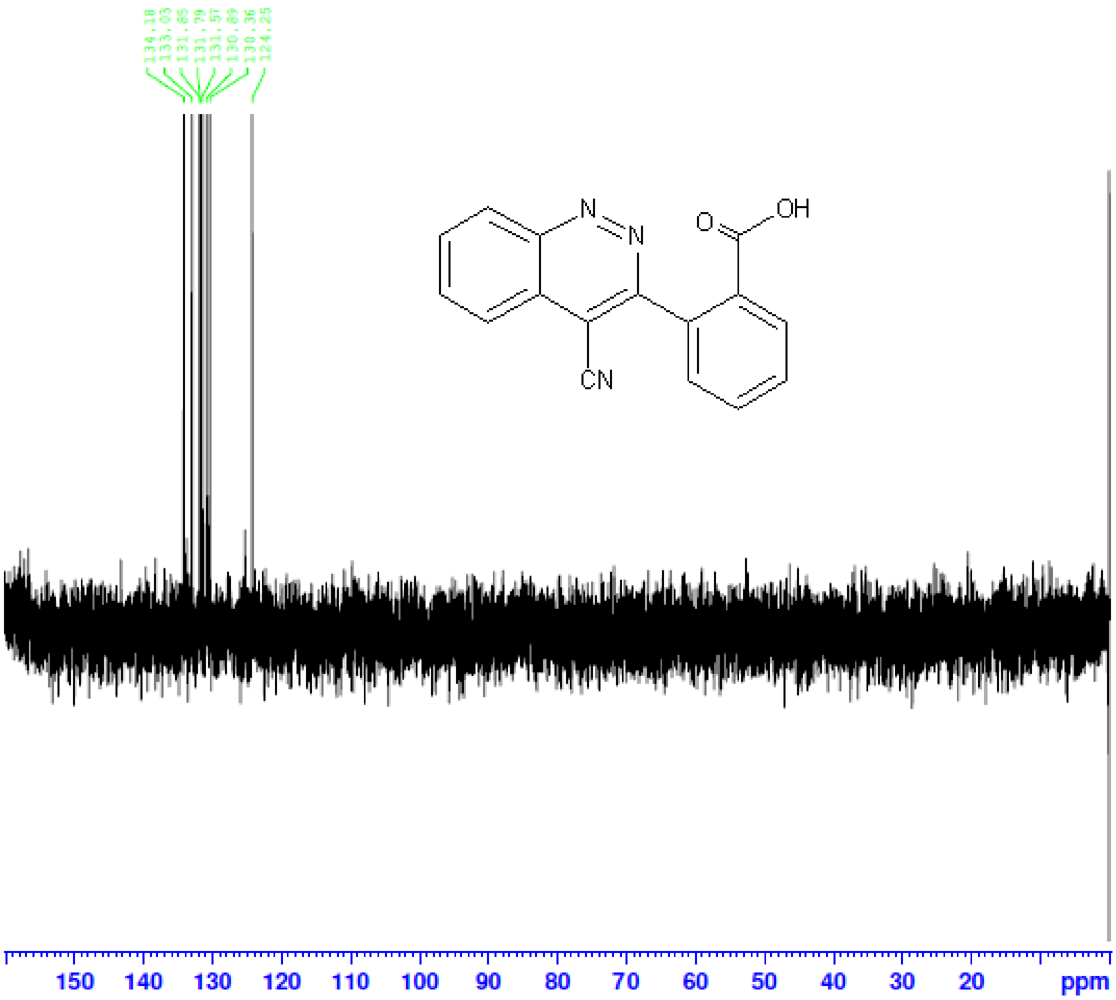

^{13}C DEPT 135-NMR of **15** in $CDCl_3$ at room temperature.

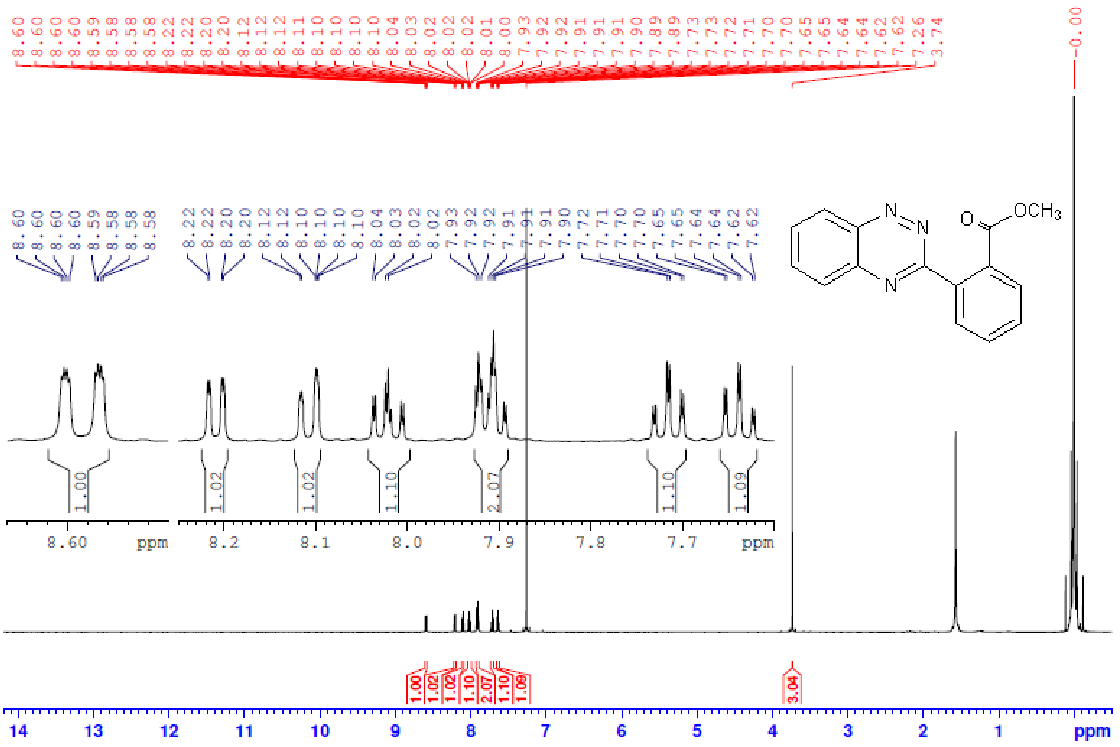

^{1}H-NMR of **16a** in $CDCl_3$ at room temperature.

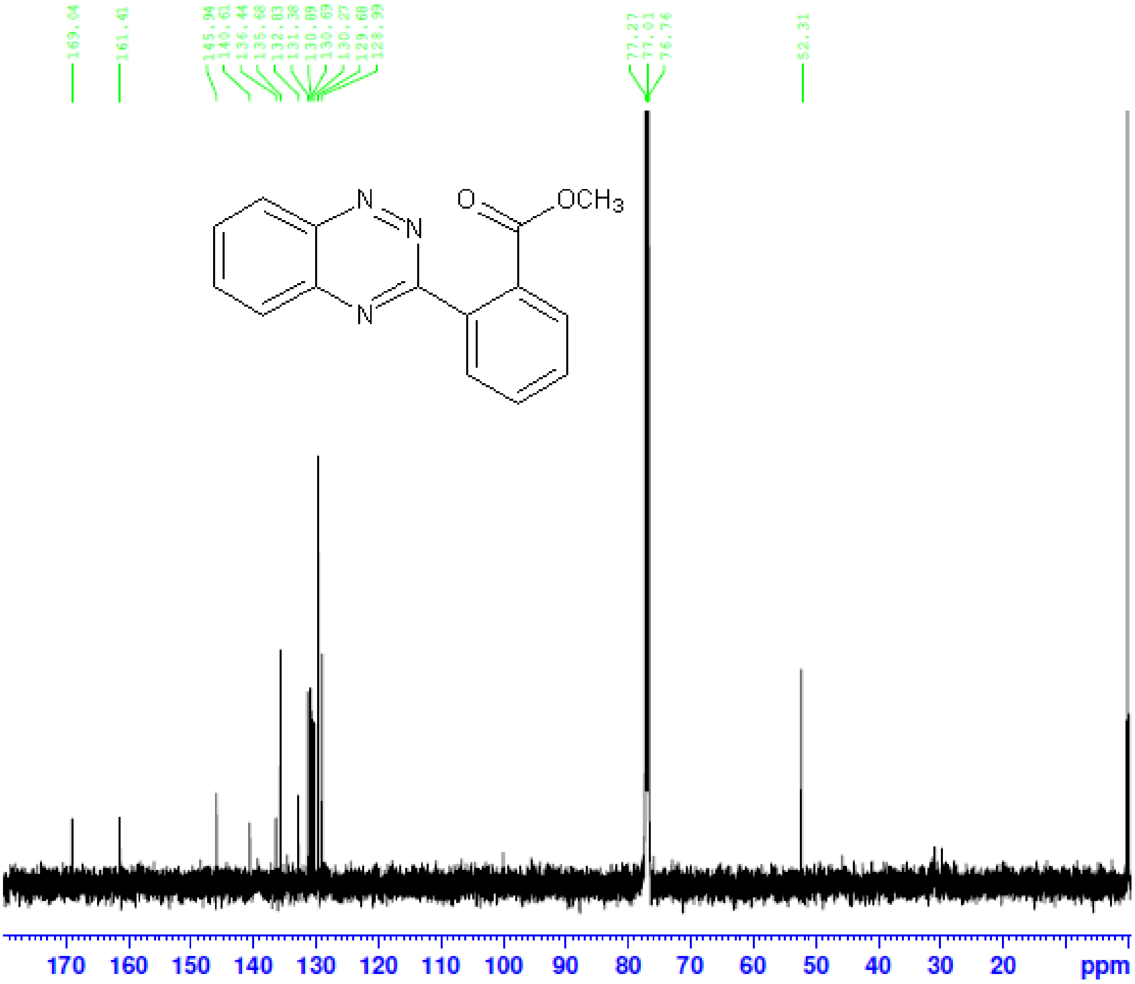

^{13}C-NMR of **16a** in $CDCl_3$ at room temperature.

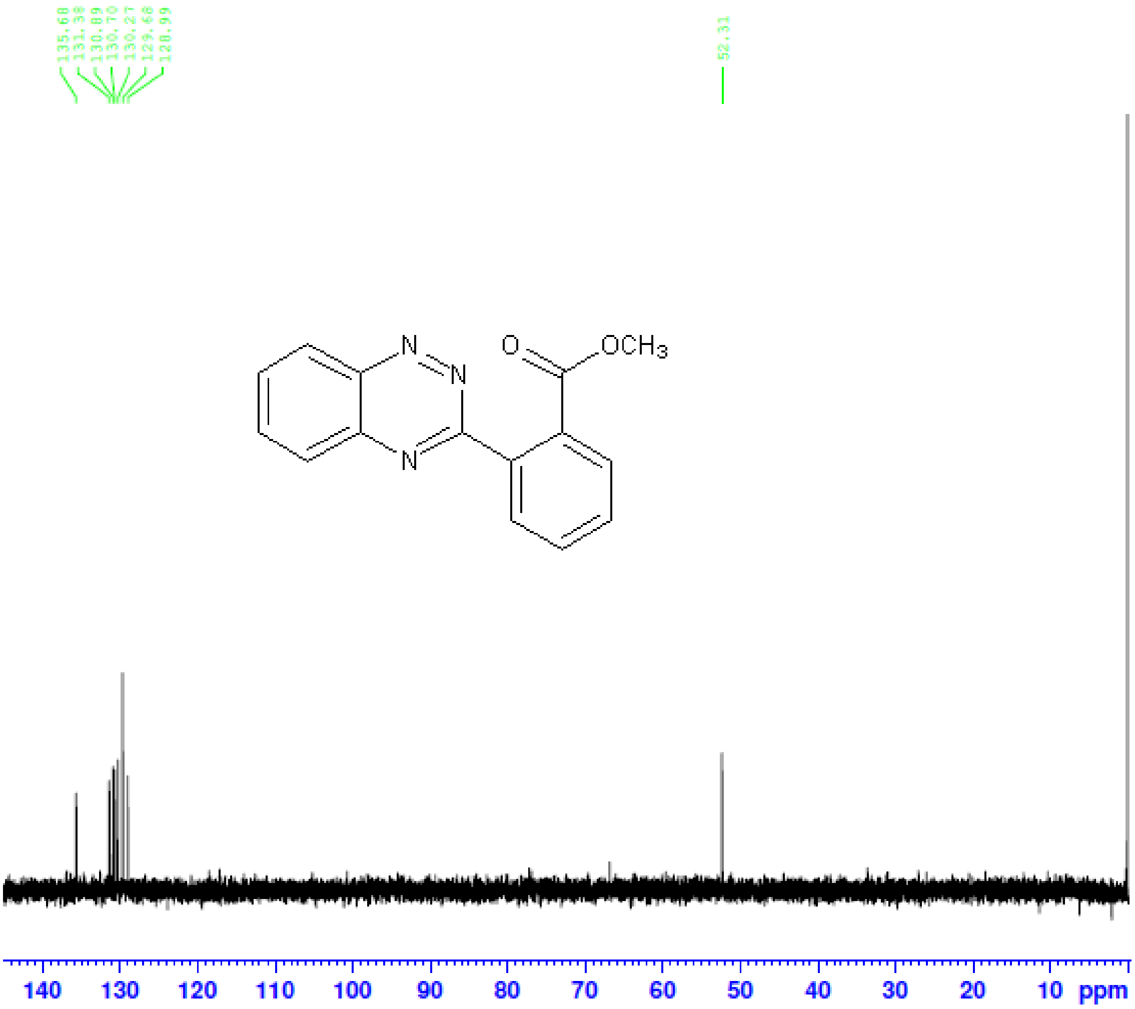

^{13}C DEPT 135-NMR of **16a** in $CDCl_3$ at room temperature.

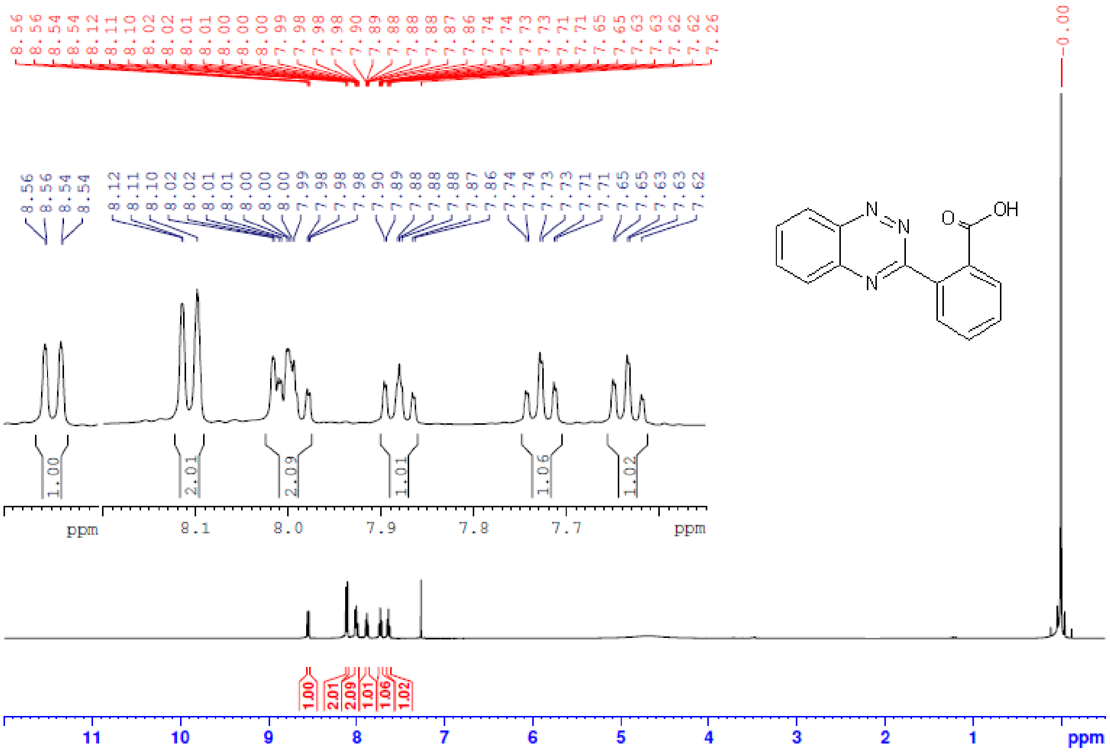

^{1}H-NMR of **17a** in $CDCl_3$ at room temperature.

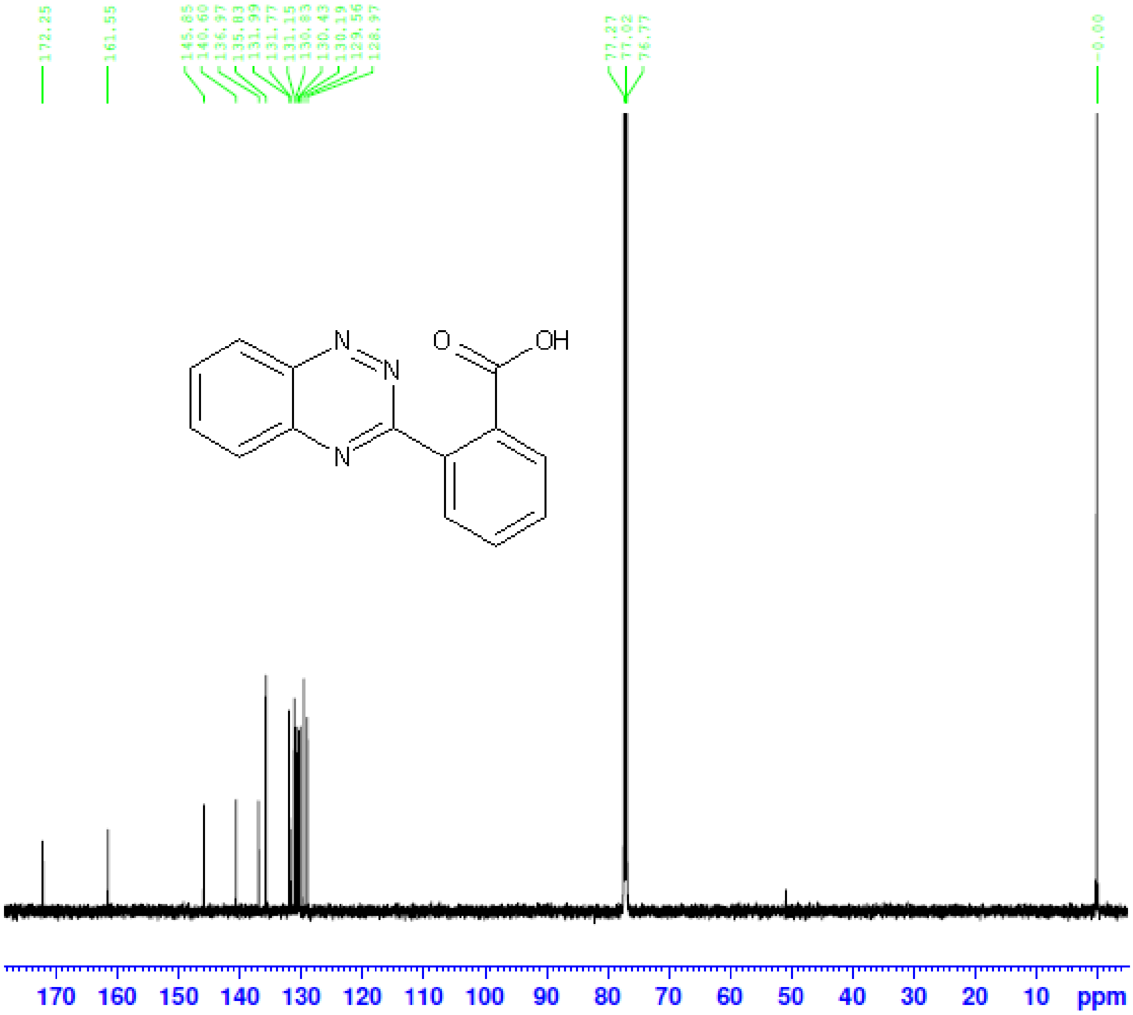

^{13}C-NMR of **17a** in $CDCl_3$ at room temperature.

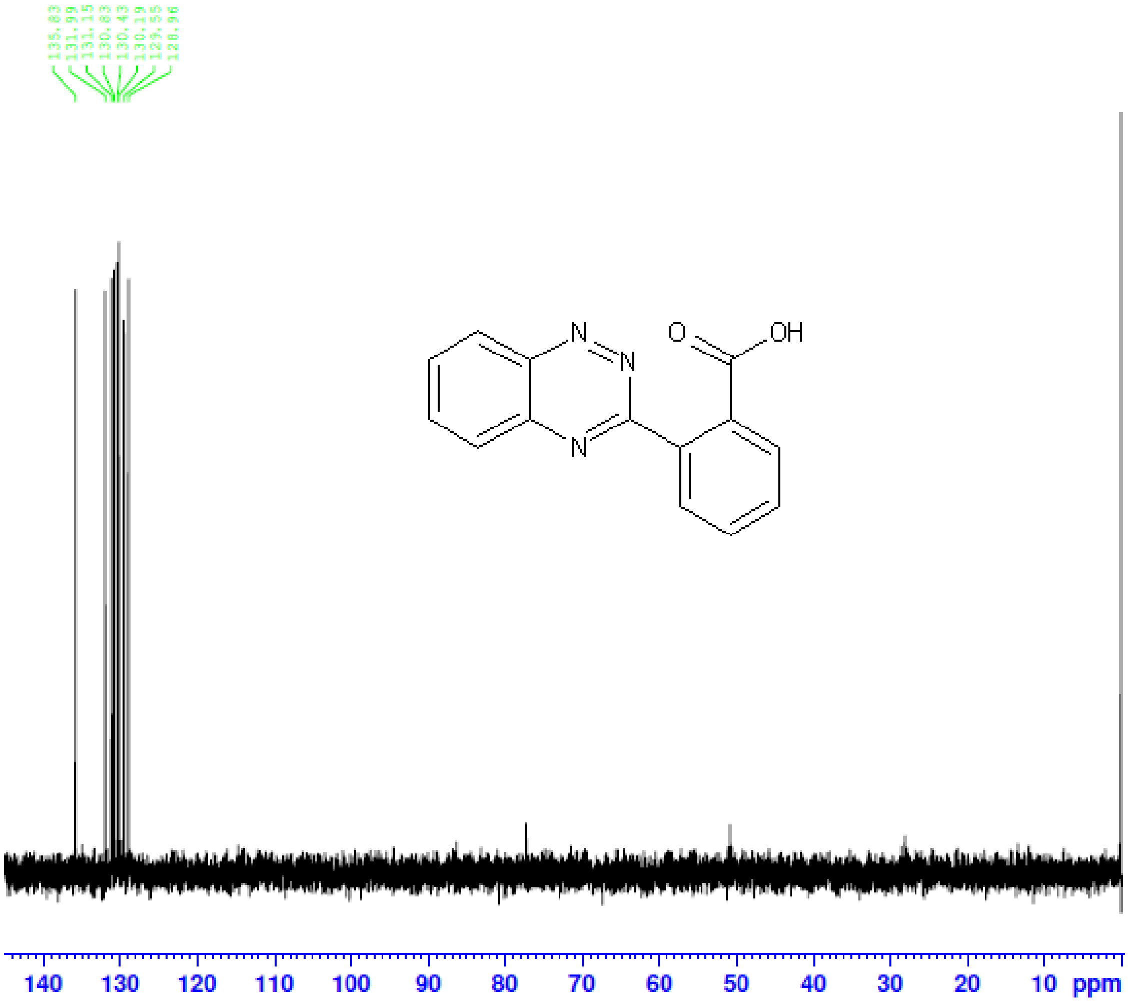

^{13}C DEPT 135-NMR of **17a** in $CDCl_3$ at room temperature.

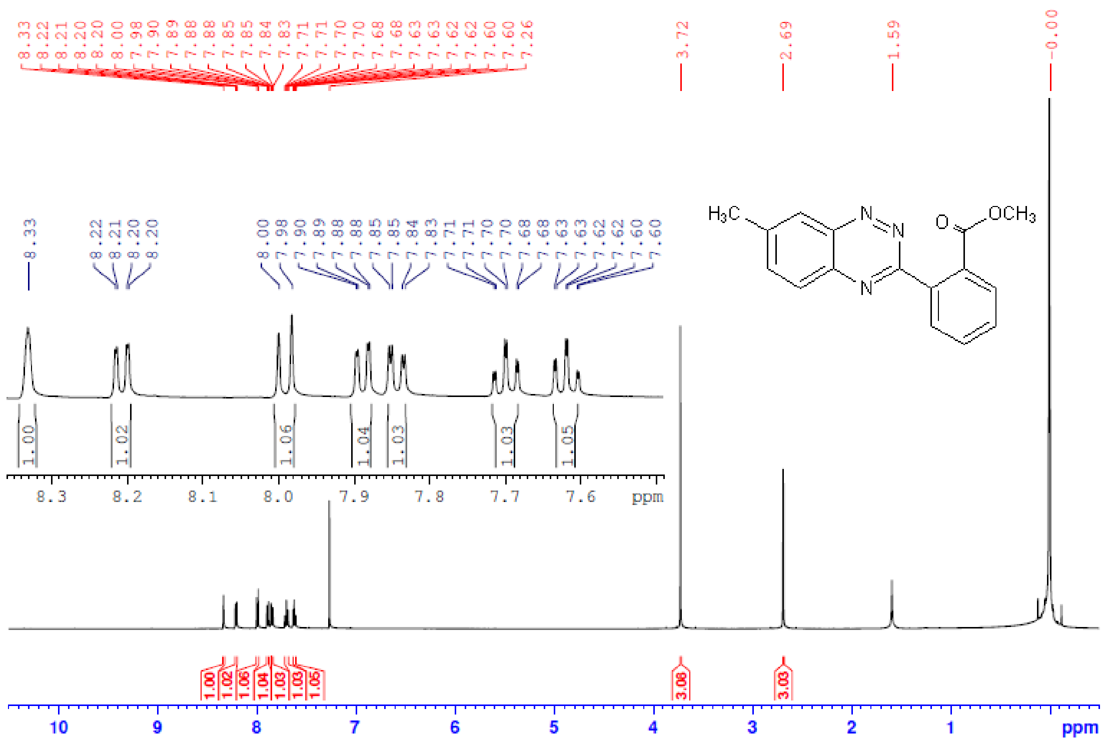

^{1}H-NMR of **16b** in $CDCl_3$ at room temperature.

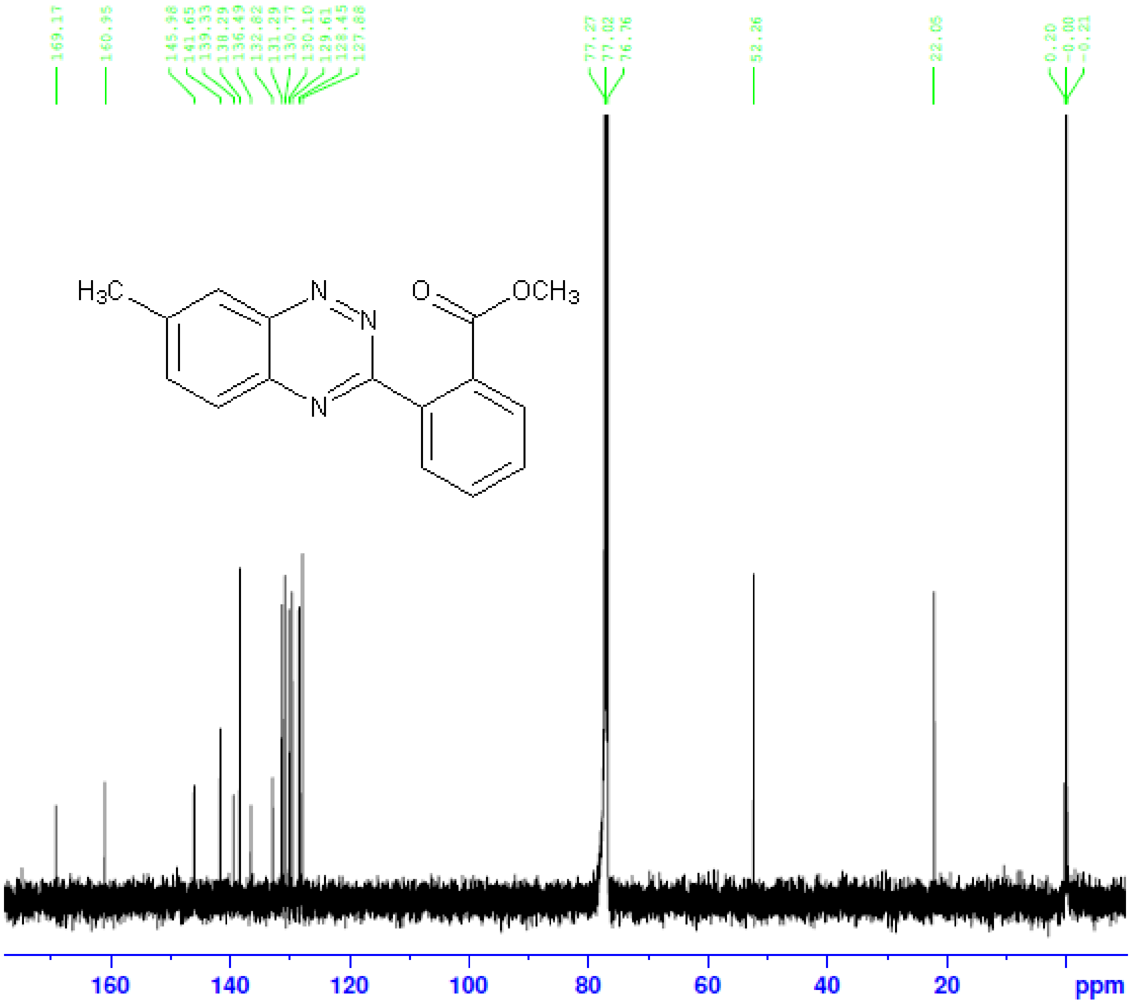

^{13}C-NMR of **16b** in $CDCl_3$ at room temperature.

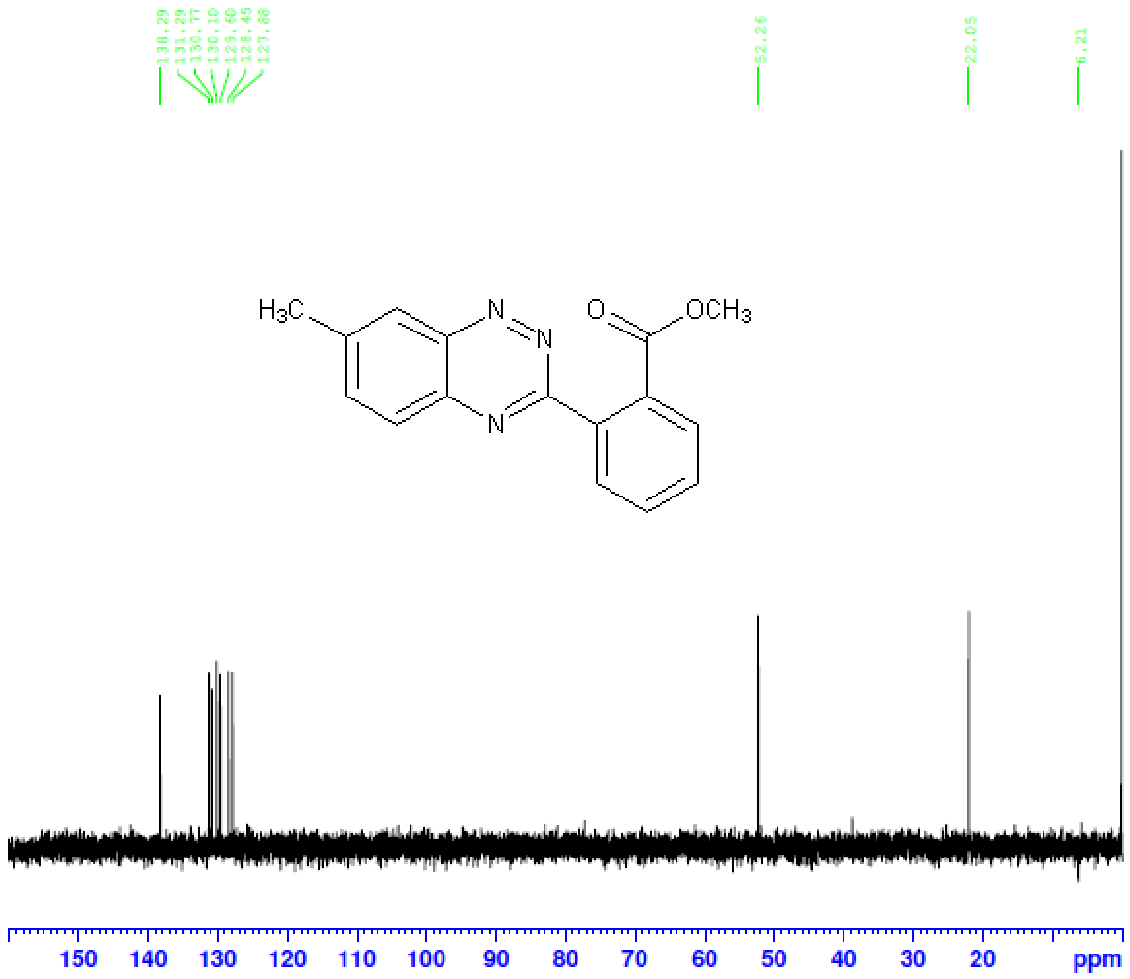

^{13}C DEPT 135-NMR of **16b** in $CDCl_3$ at room temperature.

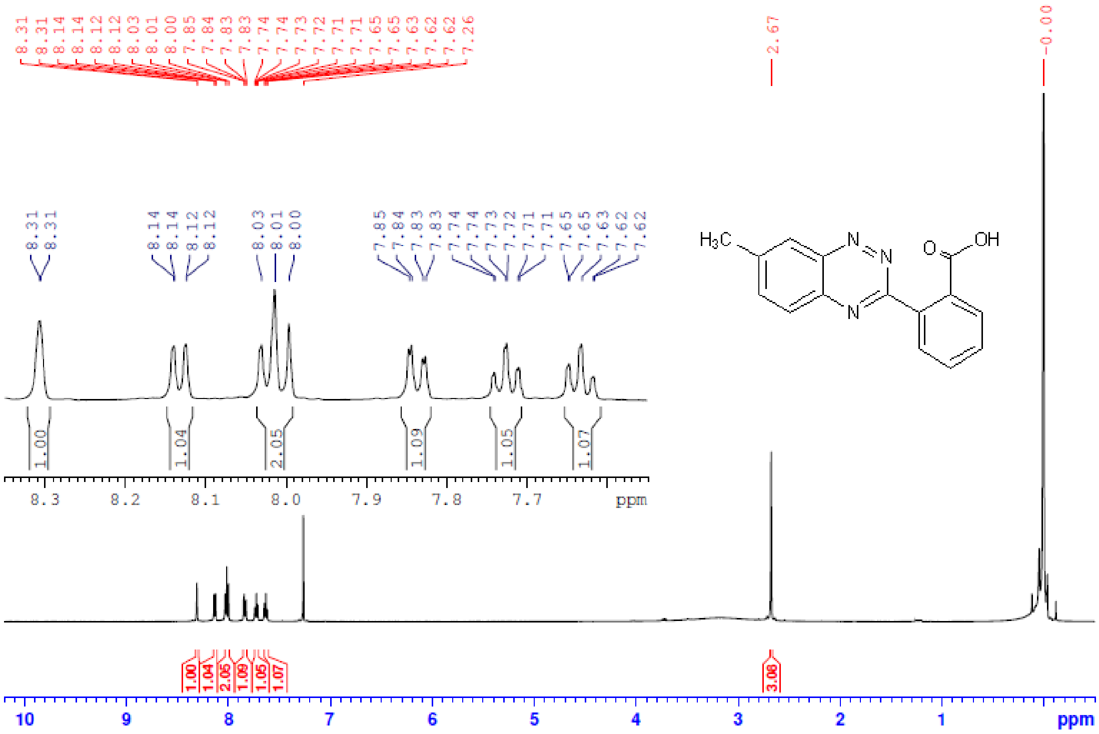

^{1}H-NMR of **17b** in $CDCl_3$ at room temperature.

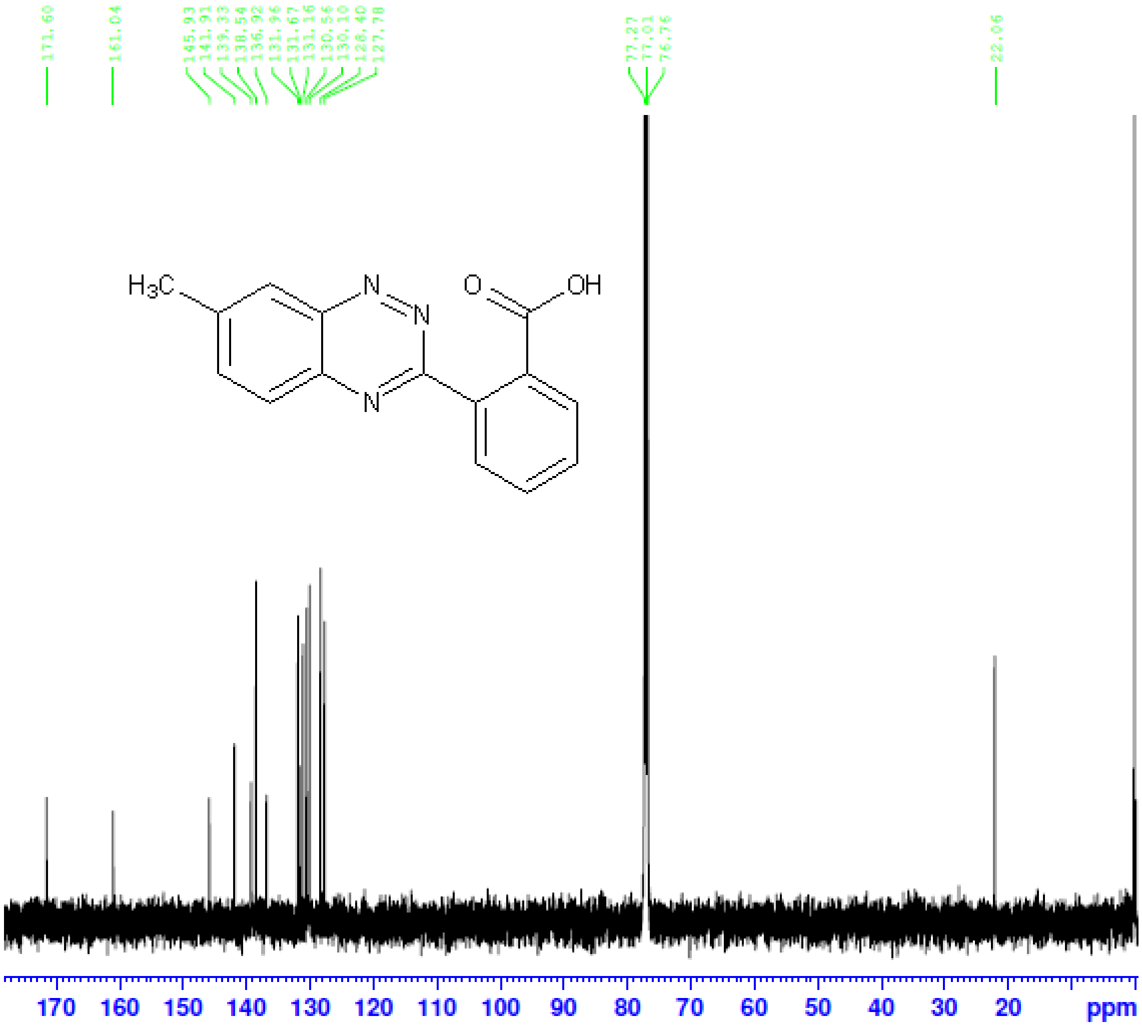

^{13}C-NMR of **17b** in $CDCl_3$ at room temperature.

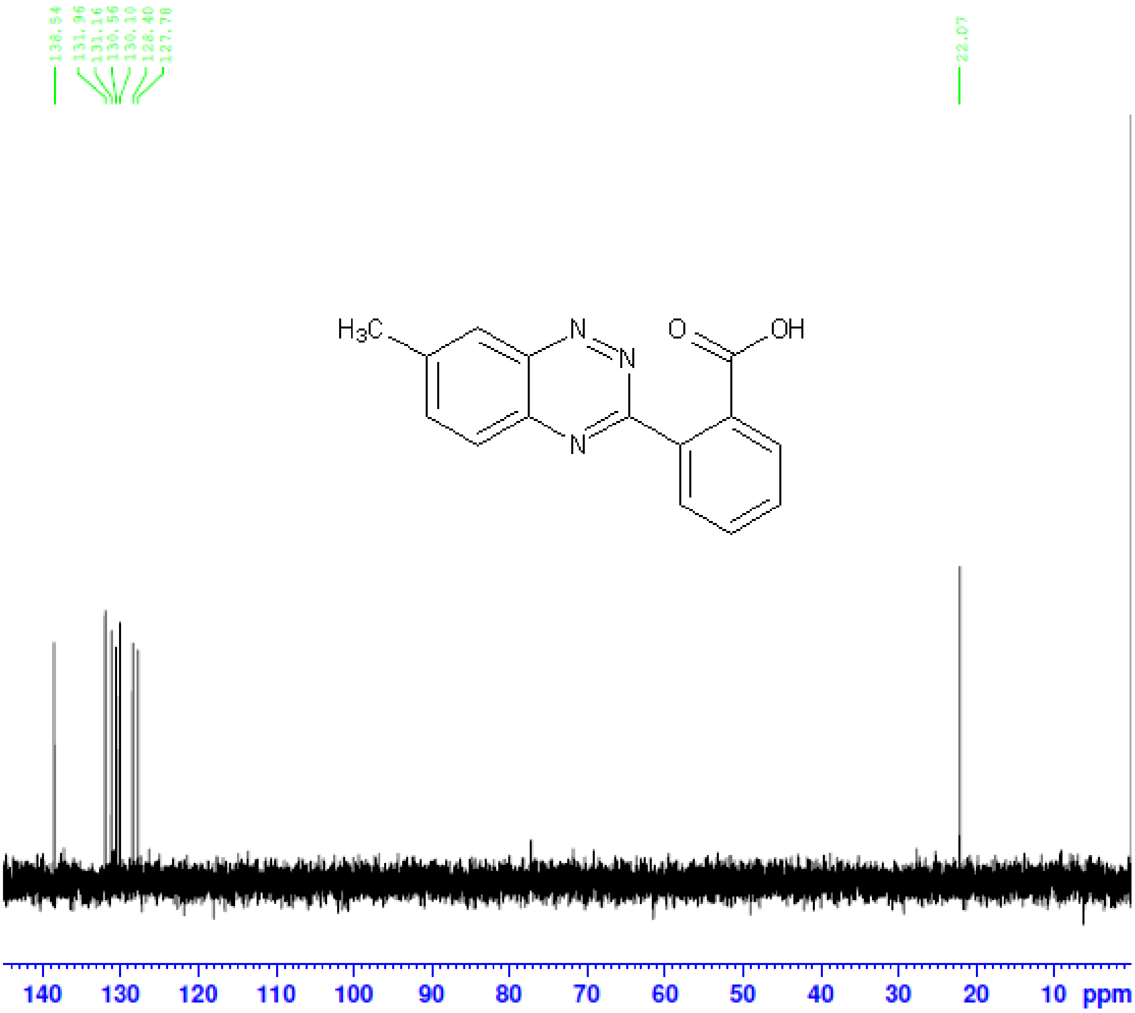

^{13}C DEPT 135-NMR of **17b** in $CDCl_3$ at room temperature.

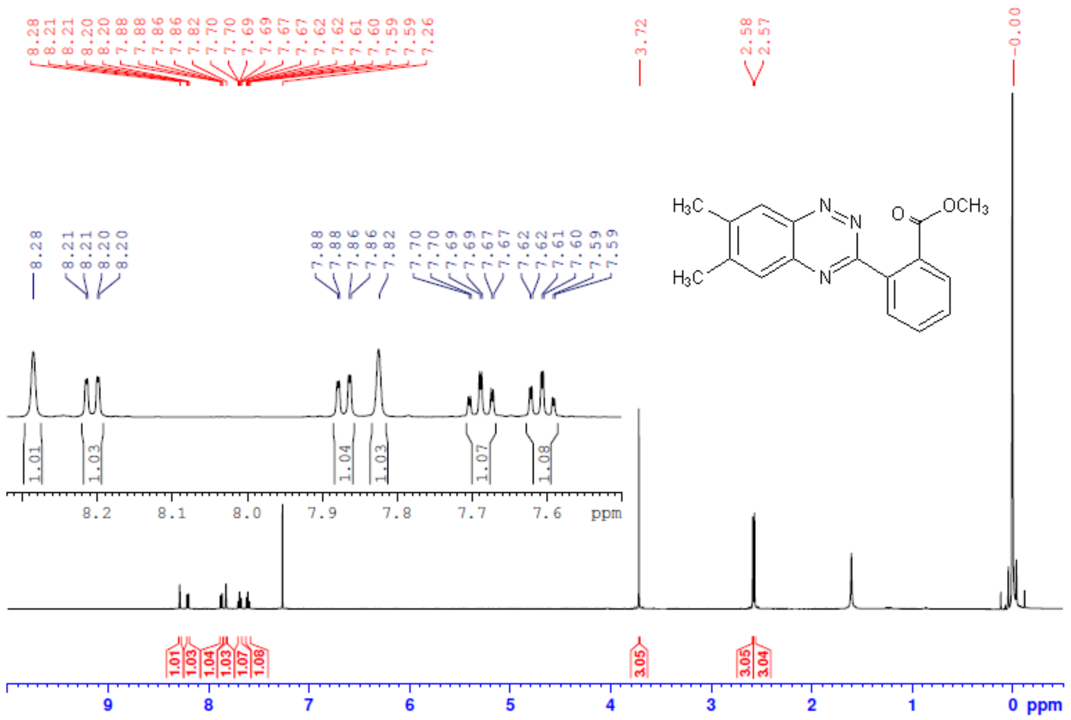

^{1}H-NMR of **16c** in $CDCl_3$ at room temperature.

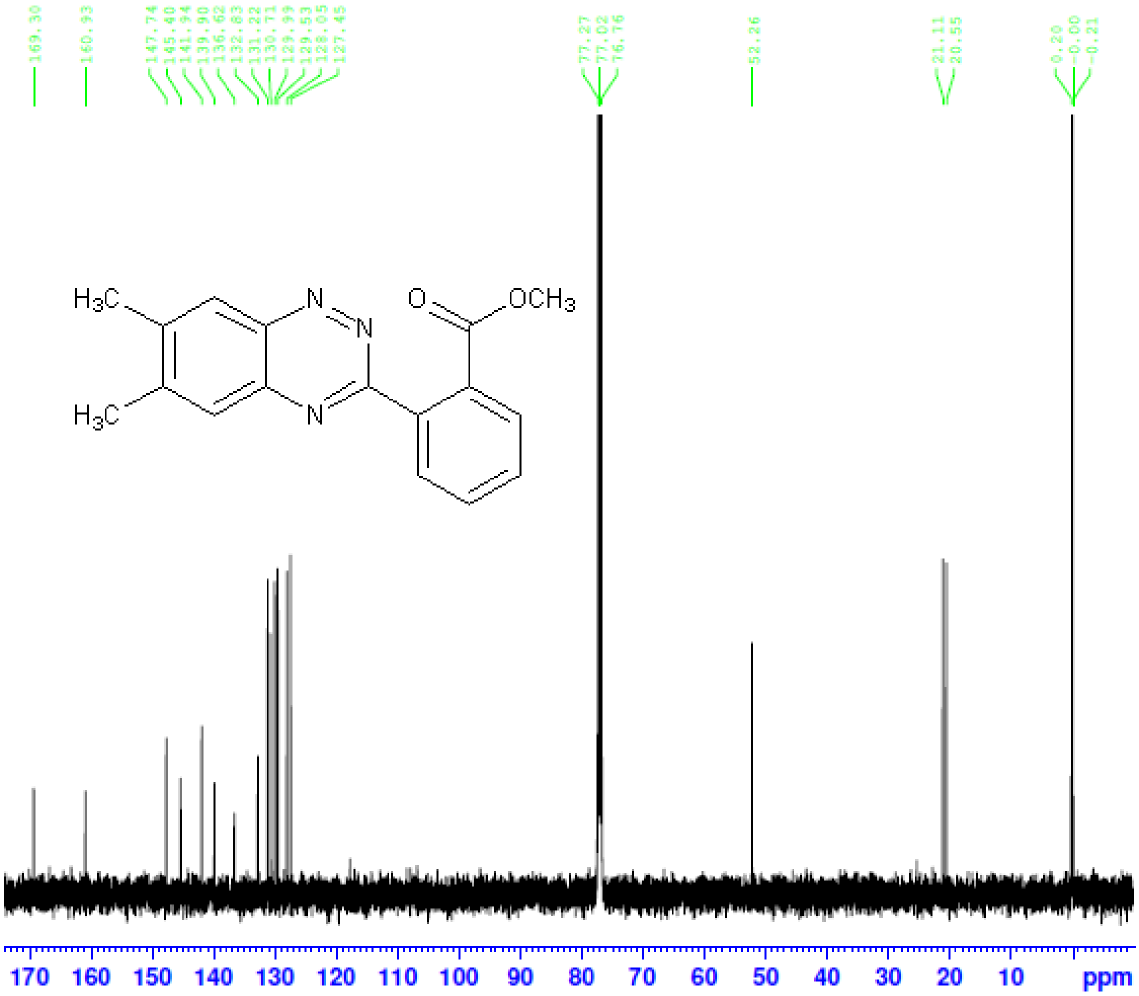

^{13}C-NMR of **16c** in $CDCl_3$ at room temperature.

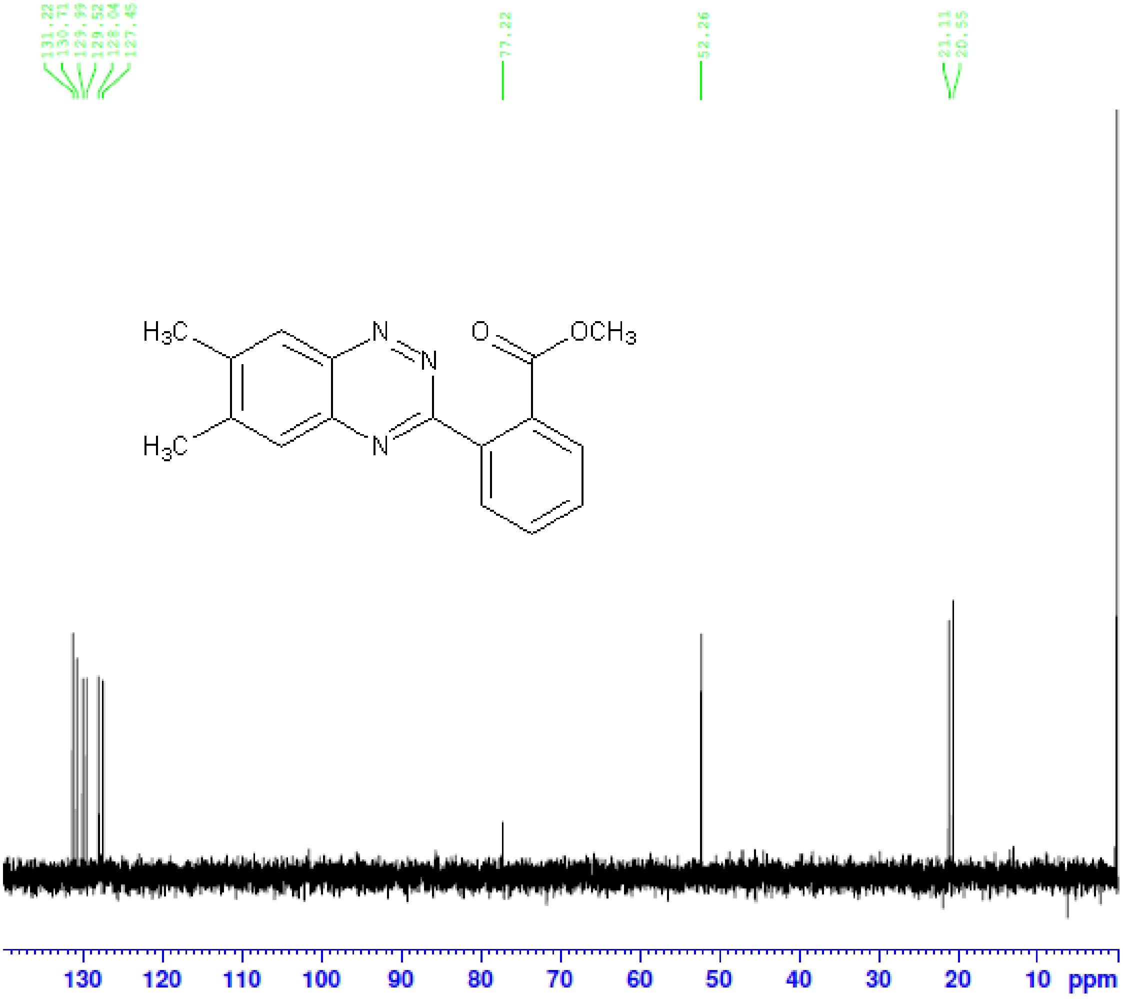

^{13}C DEPT 135-NMR of **16c** in $CDCl_3$ at room temperature.

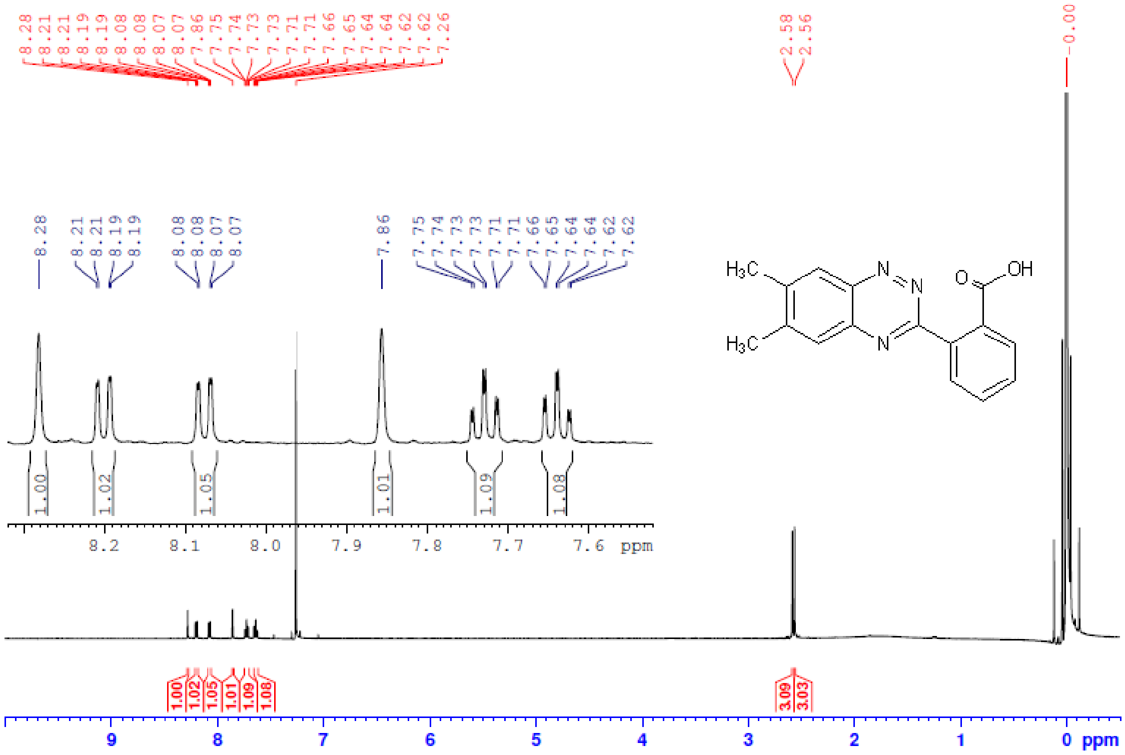

^{1}H-NMR of **17c** in $CDCl_3$ at room temperature.

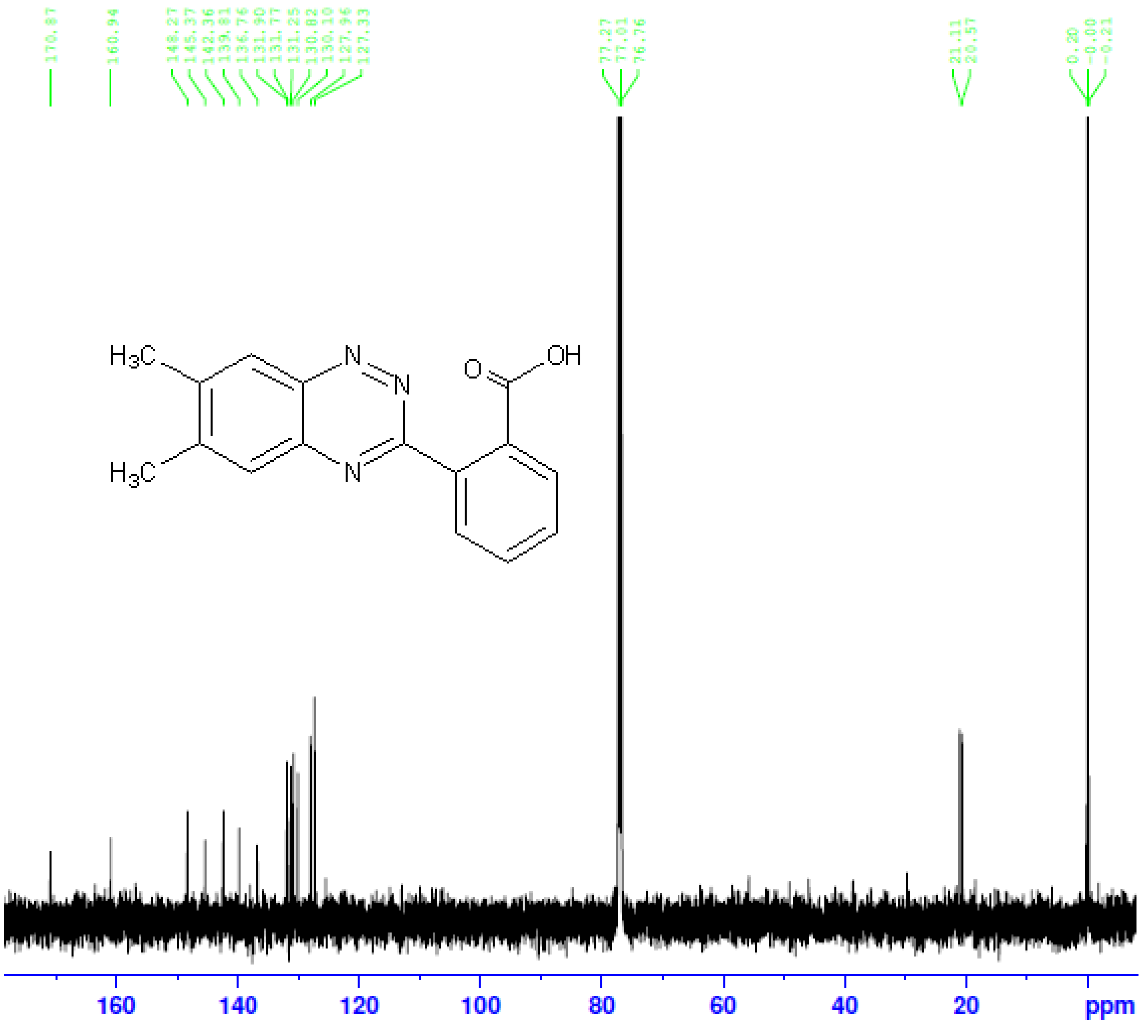

^{13}C-NMR of **17c** in $CDCl_3$ at room temperature.

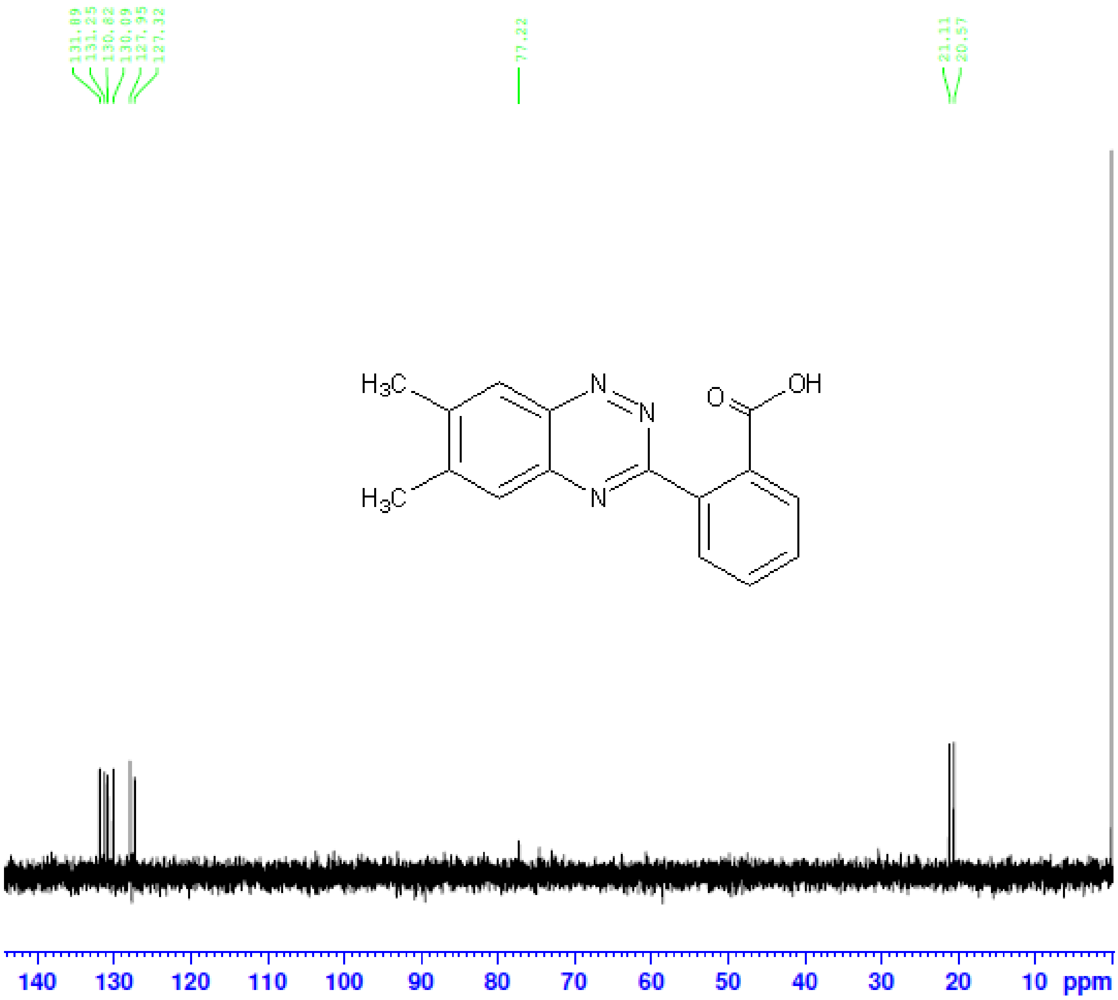

^{13}C DEPT 135-NMR of **17c** in $CDCl_3$ at room temperature.

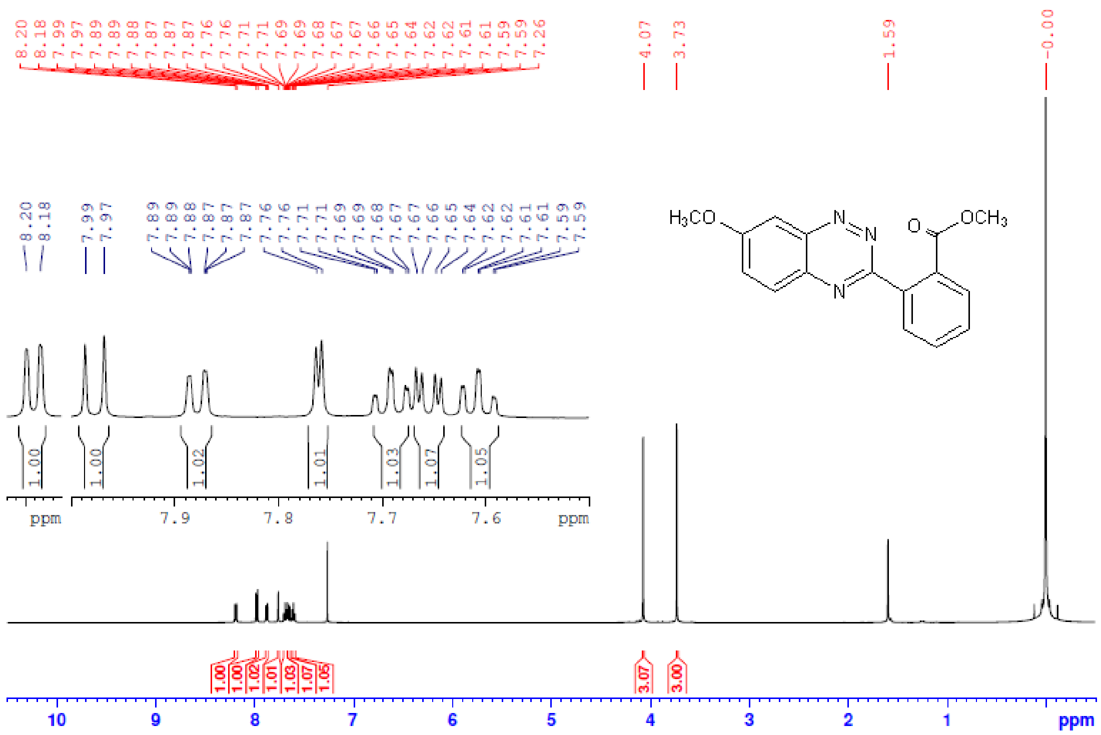

^{1}H-NMR of **16d** in $CDCl_3$ at room temperature.

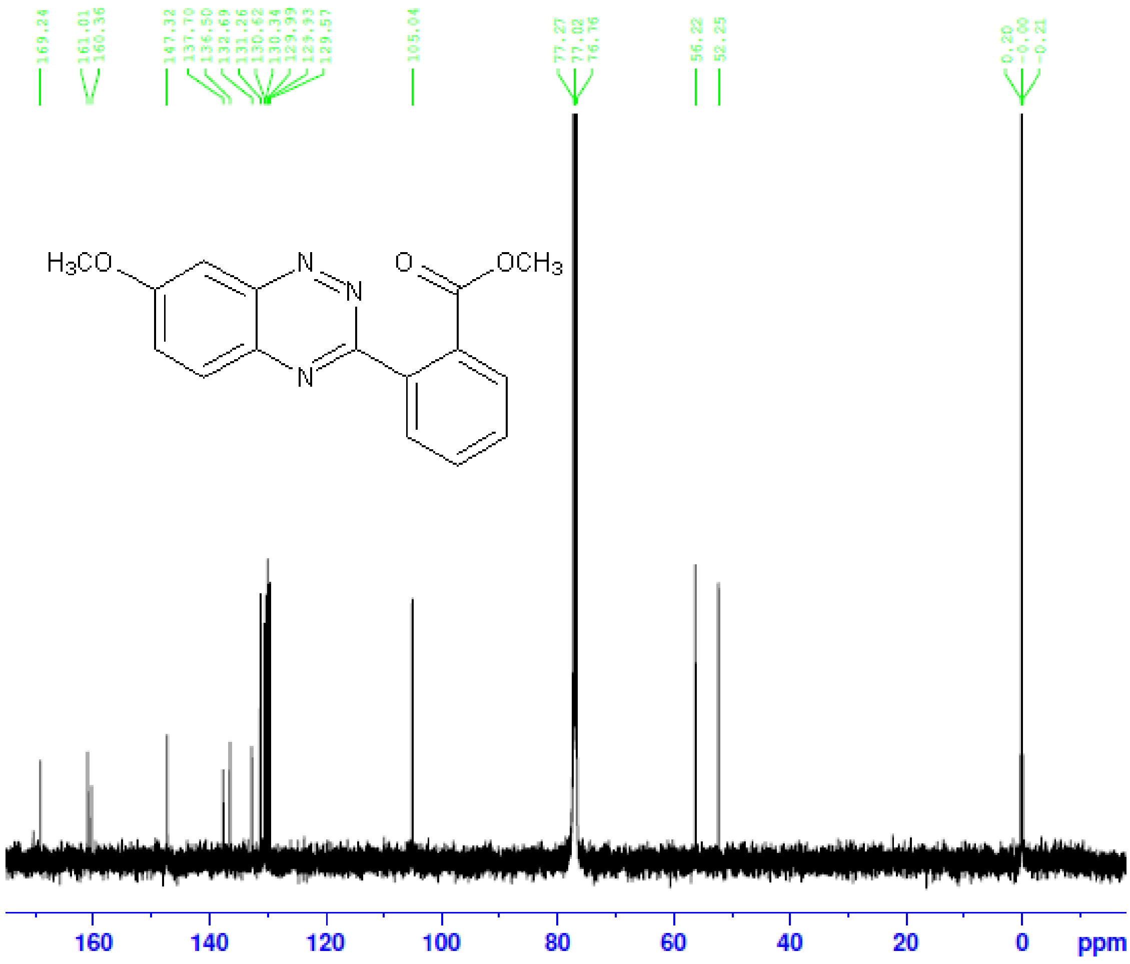

^{13}C-NMR of **16d** in $CDCl_3$ at room temperature.

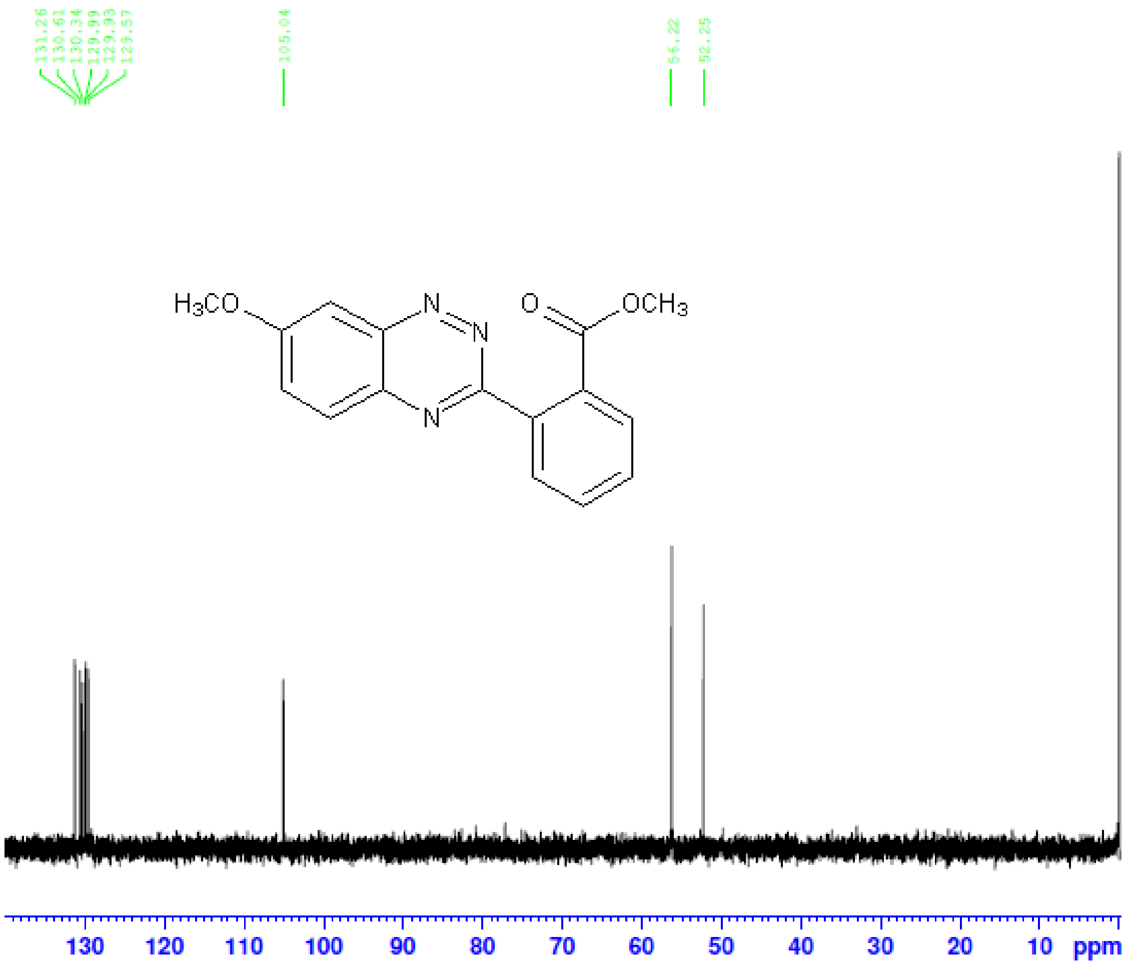

^{13}C DEPT 135-NMR of **16d** in $CDCl_3$ at room temperature.

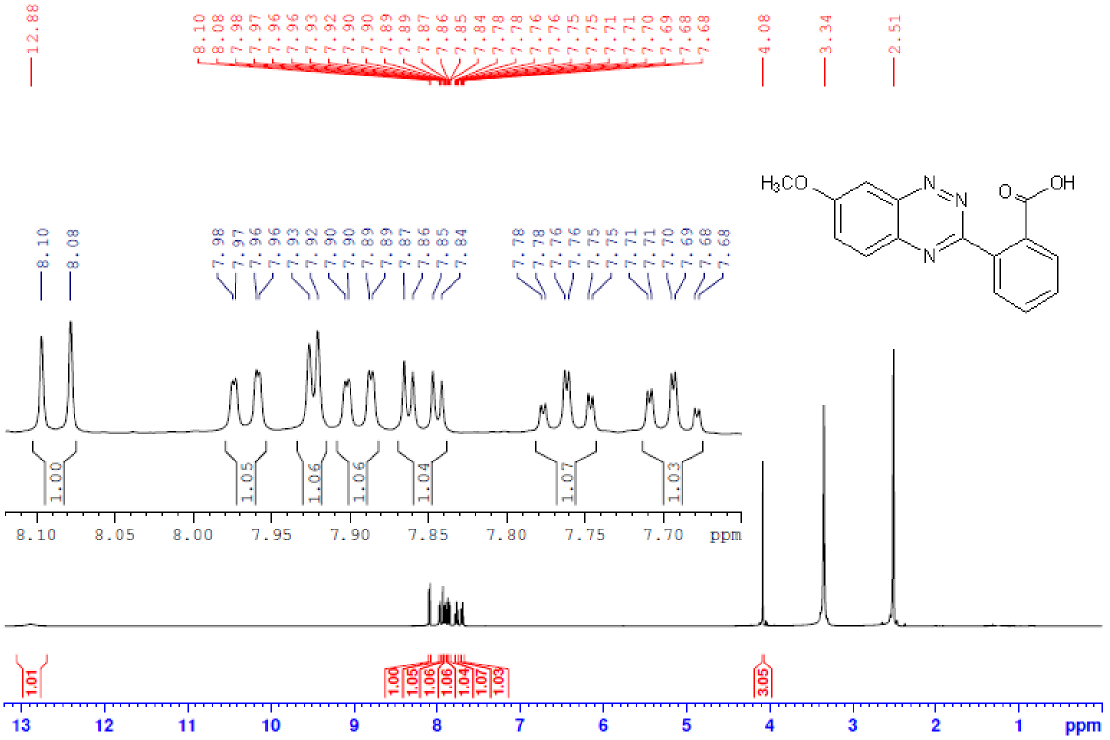

^{1}H-NMR of **17d** in DMSO-*d6* at room temperature.

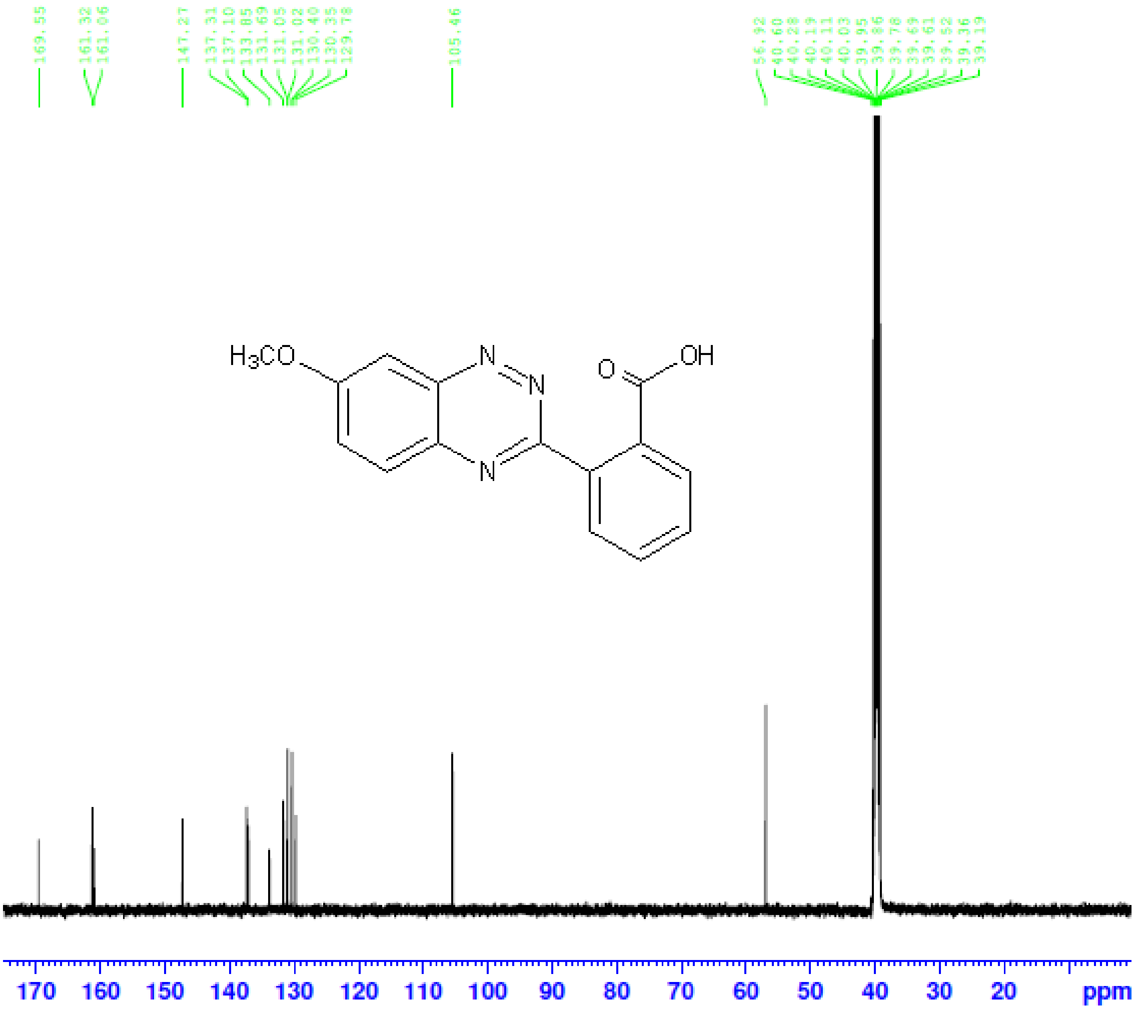

^{13}C-NMR of **17d** in DMSO-*d6* at room temperature.

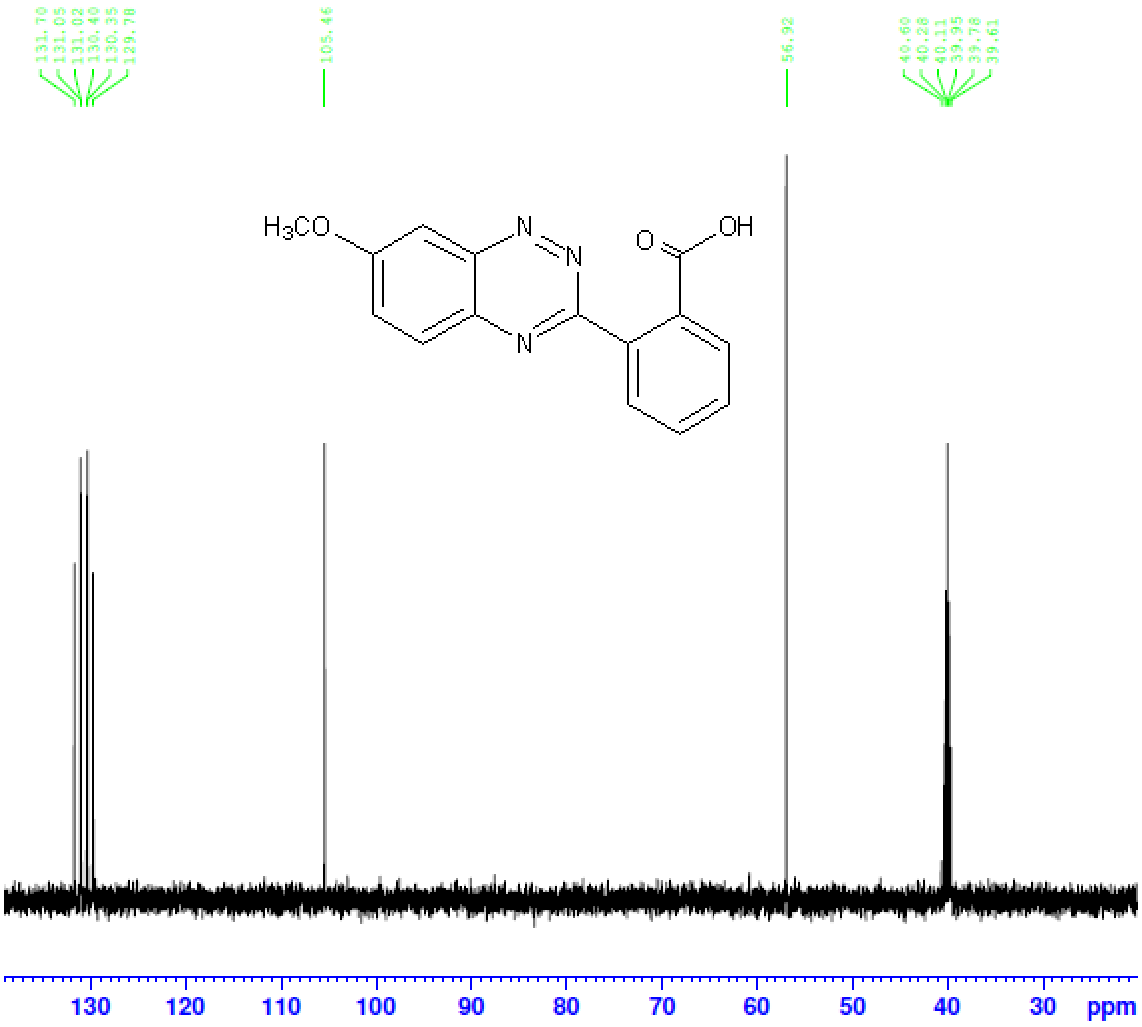

^{13}C DEPT 135-NMR of **17d** in DMSO-*d6* at room temperature.

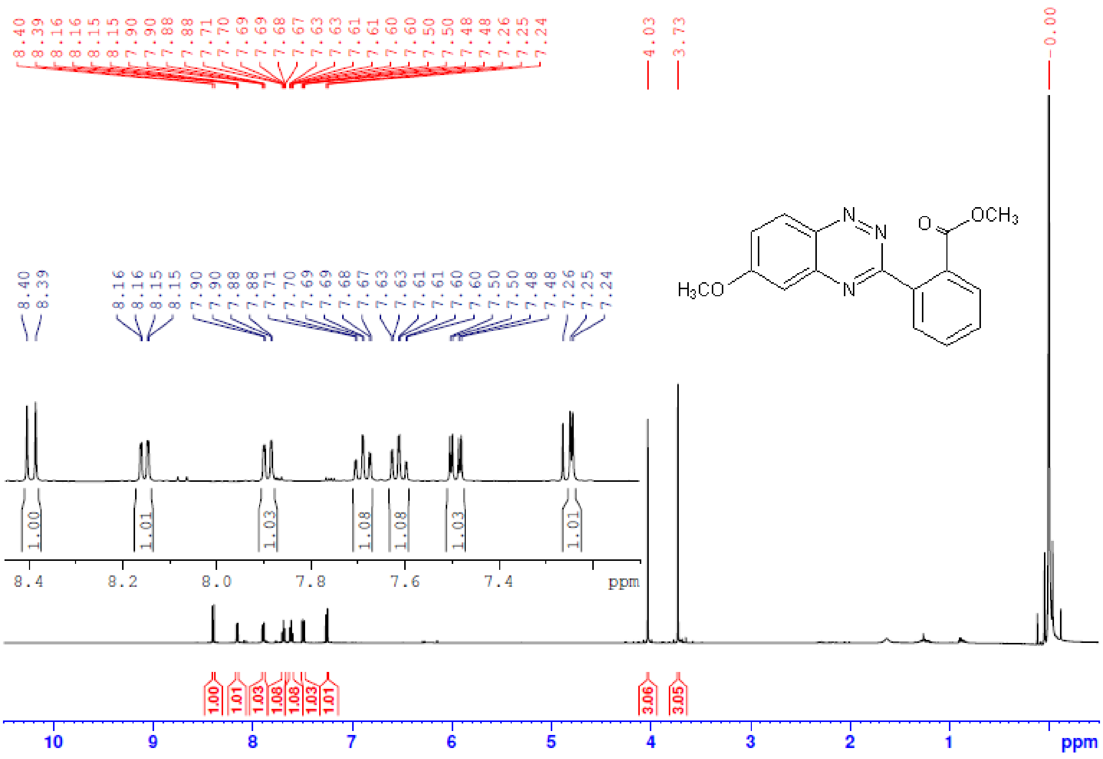

^{1}H-NMR of **16e** in $CDCl_3$ at room temperature.

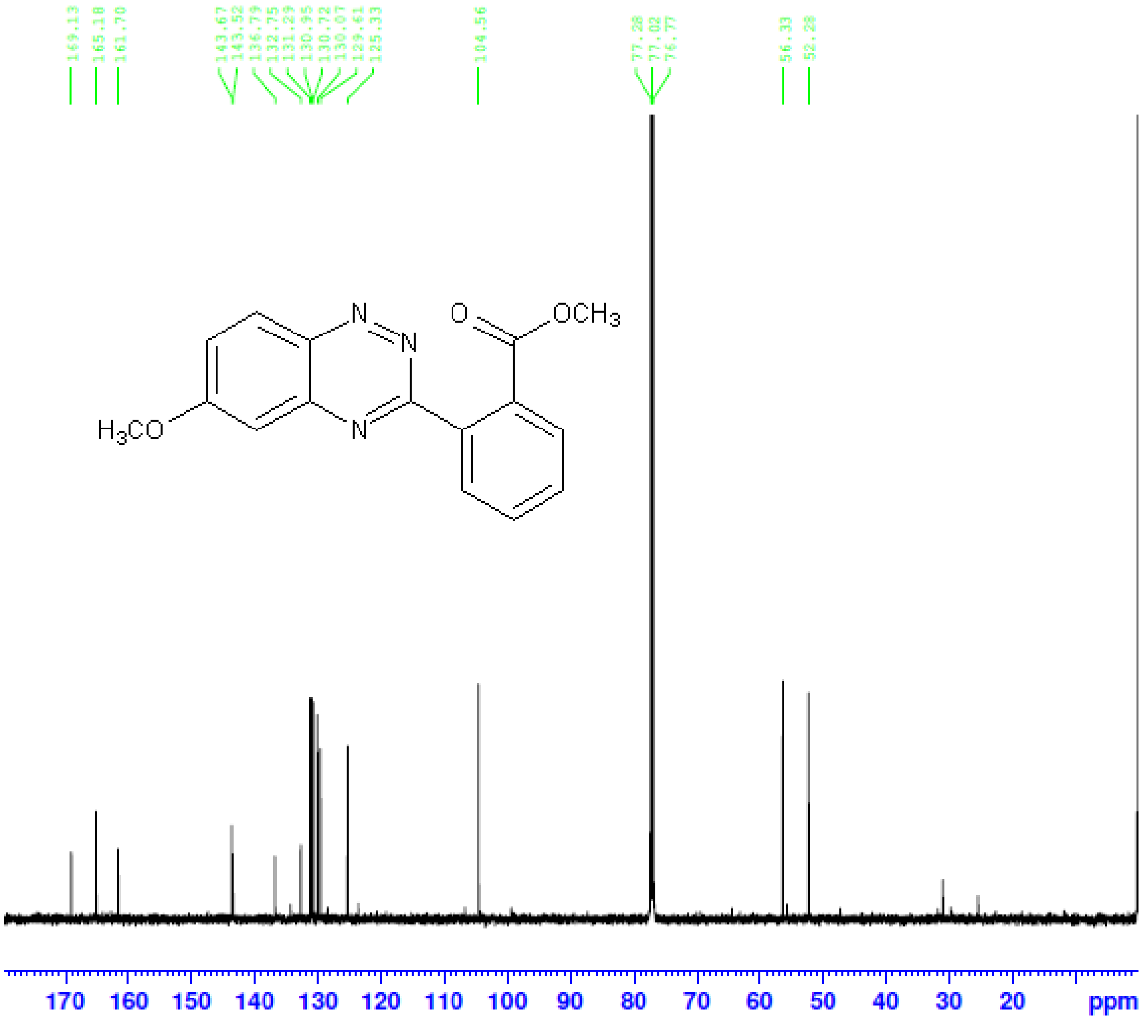

^{13}C-NMR of **16e** in $CDCl_3$ at room temperature.

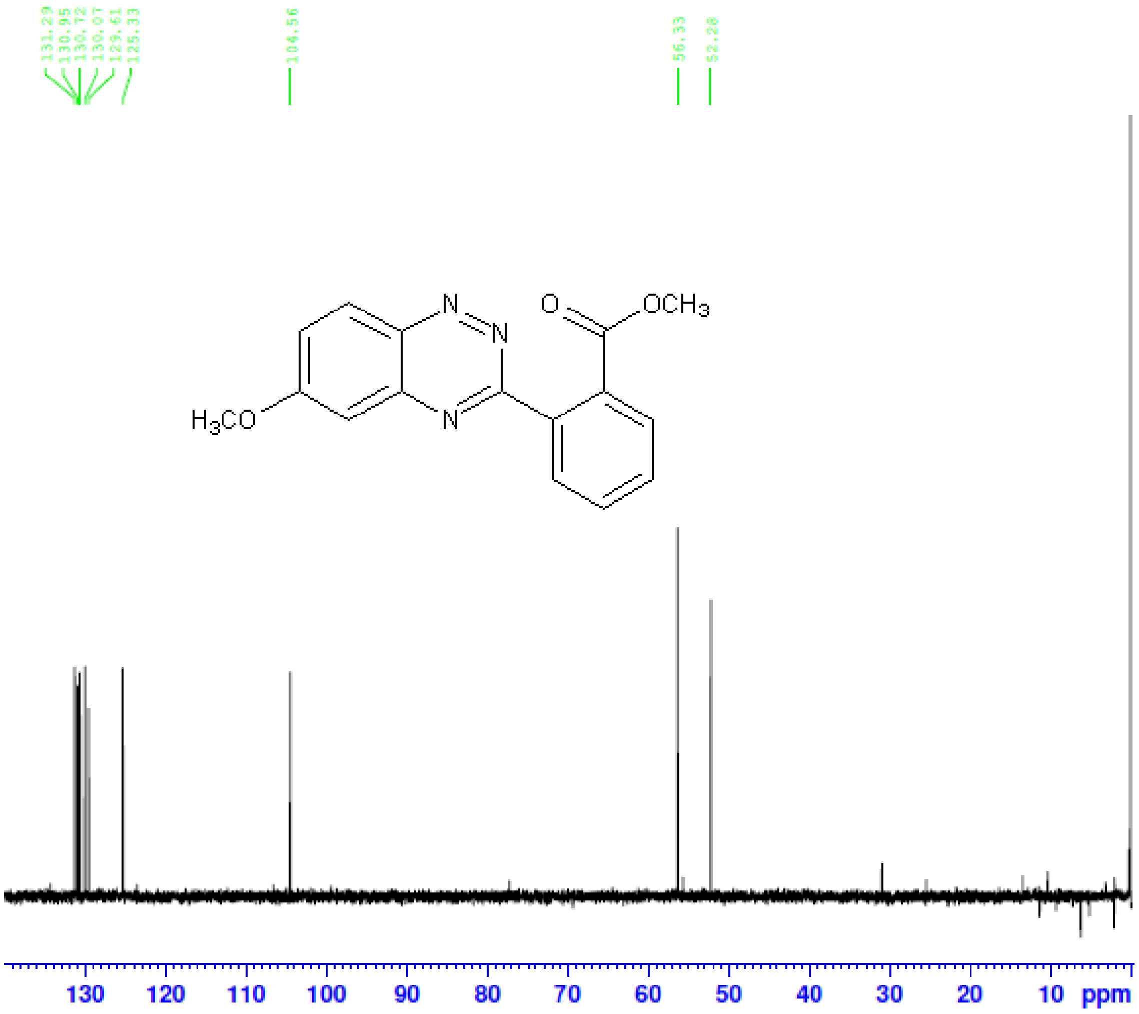

^{13}C DEPT 135-NMR of **16e** in $CDCl_3$ at room temperature.

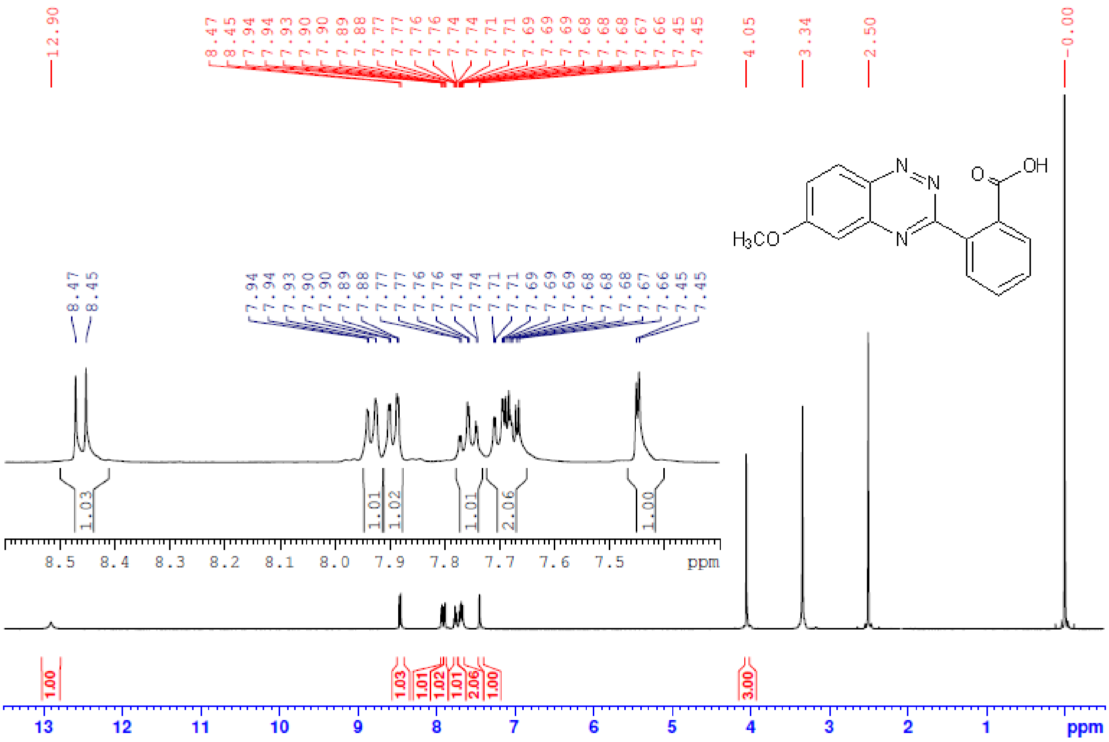

^{1}H-NMR of **17e** in DMSO-*d6* at room temperature.

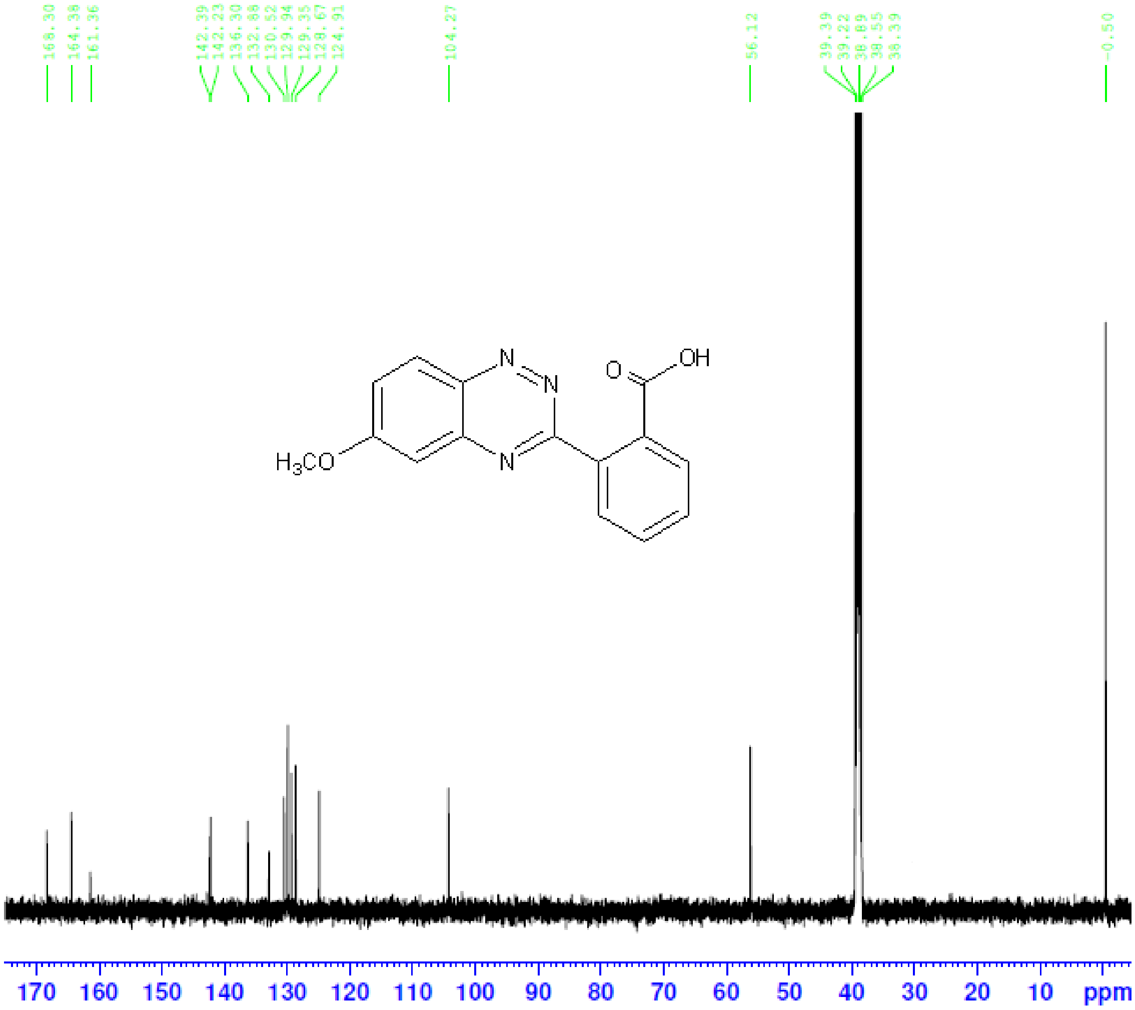

^{13}C-NMR of **17e** in DMSO-*d6* at room temperature.

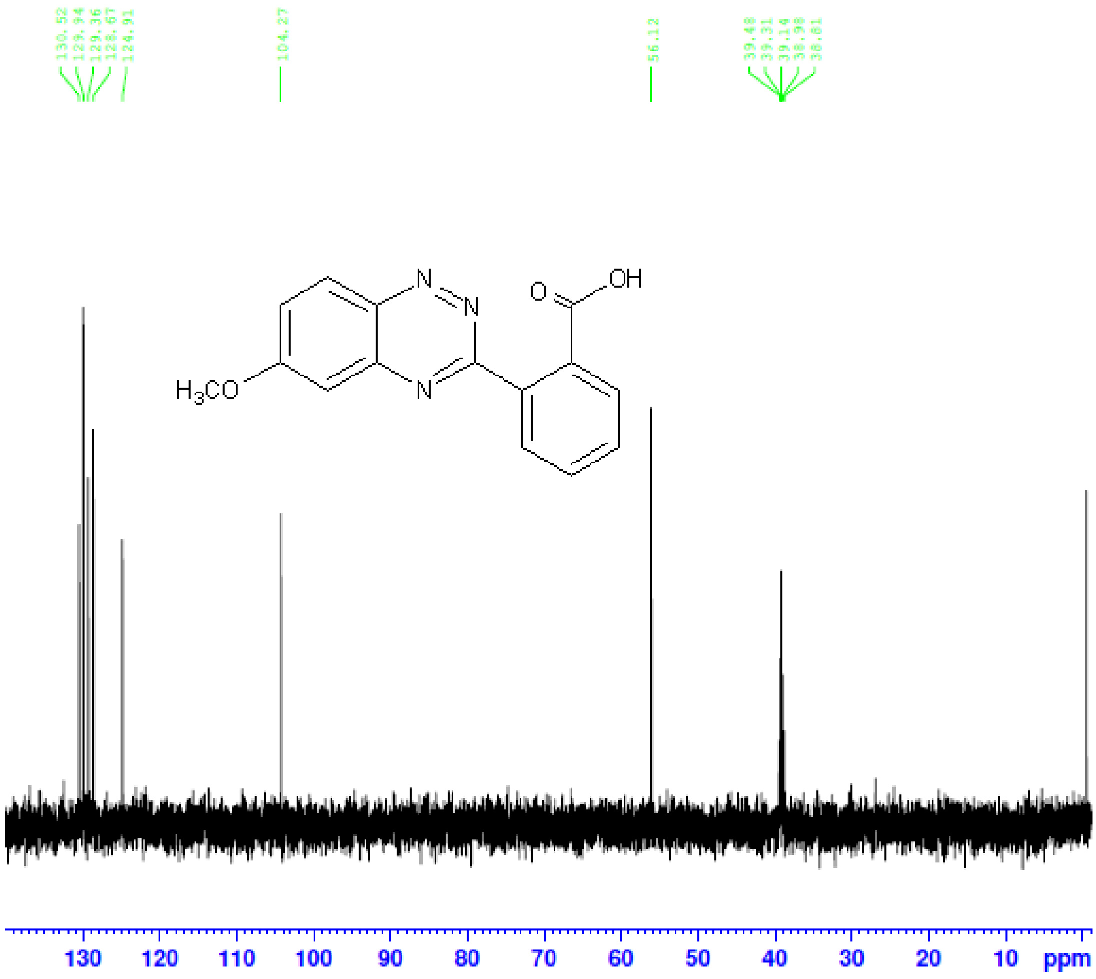

^{13}C DEPT 135-NMR of **17e** in DMSO-*d6* at room temperature.

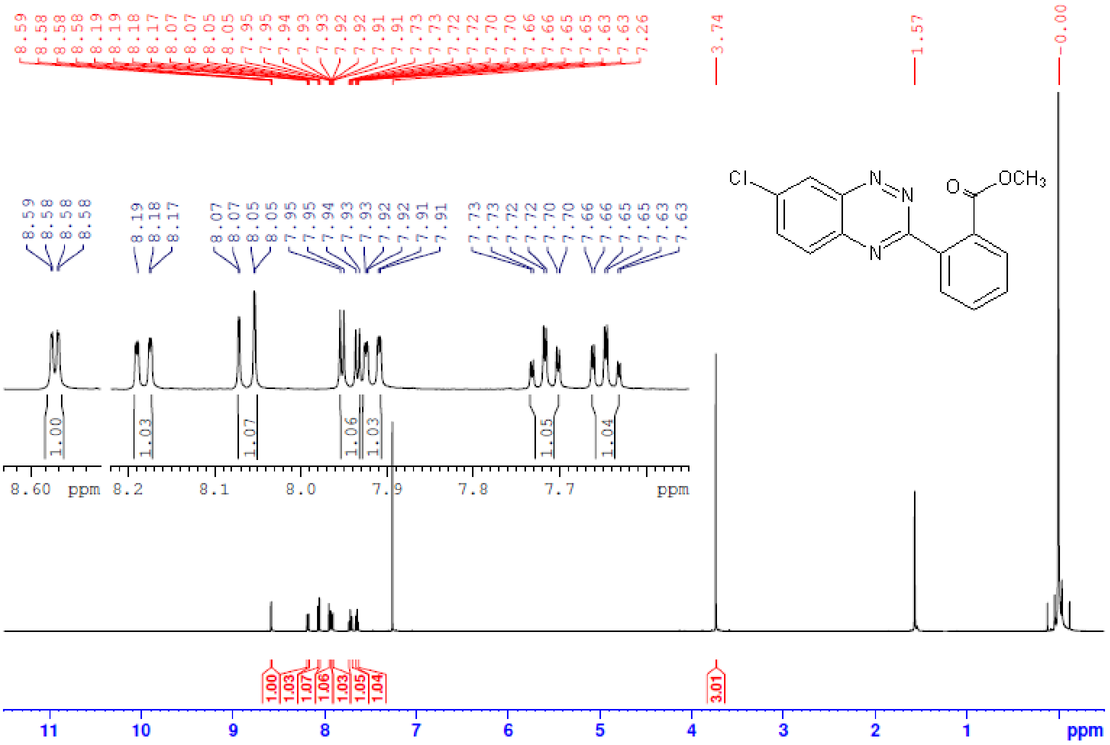

^{1}H-NMR of **16f** in $CDCl_3$ at room temperature.

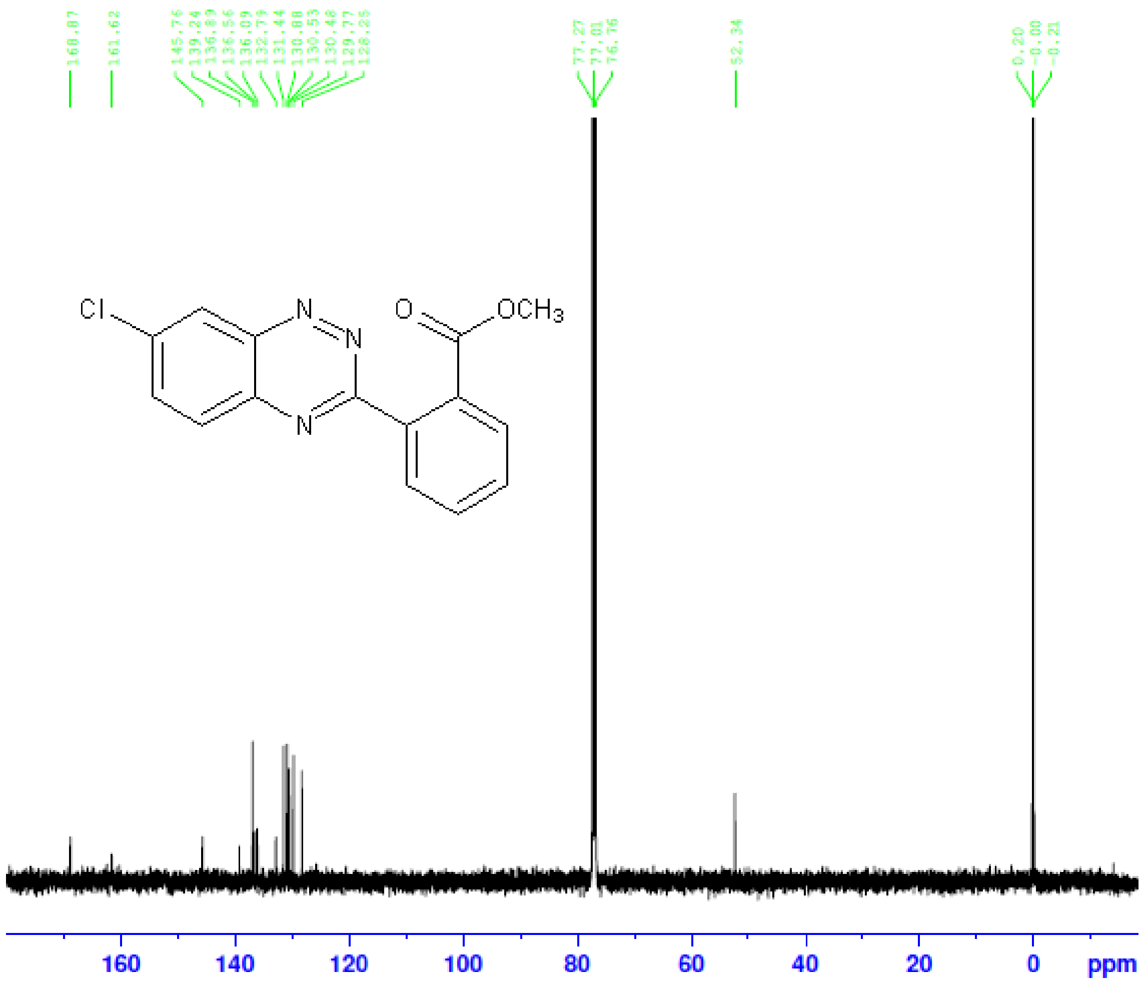

^{13}C -NMR of **16f** in $CDCl_3$ at room temperature.

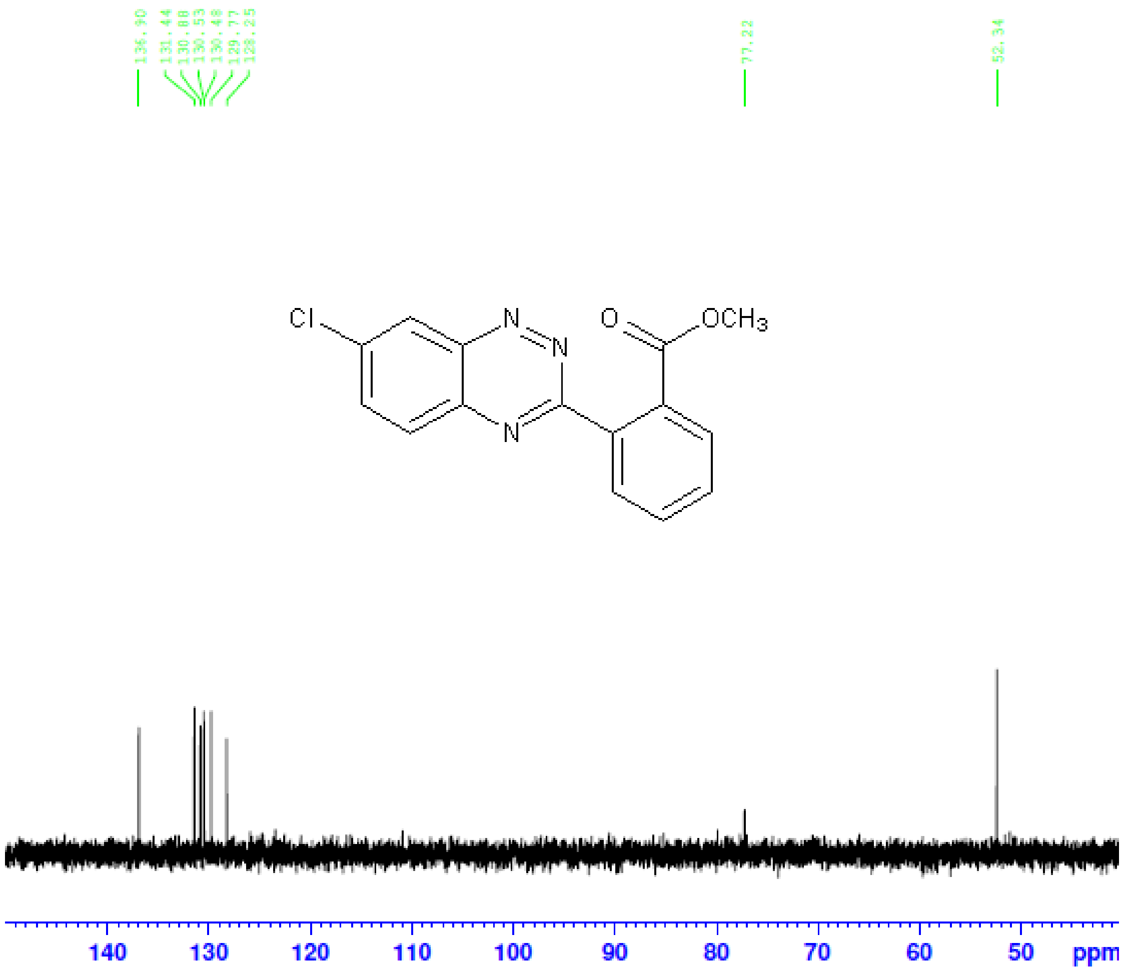

^{13}C DEPT 135-NMR of **16f** in $CDCl_3$ at room temperature.

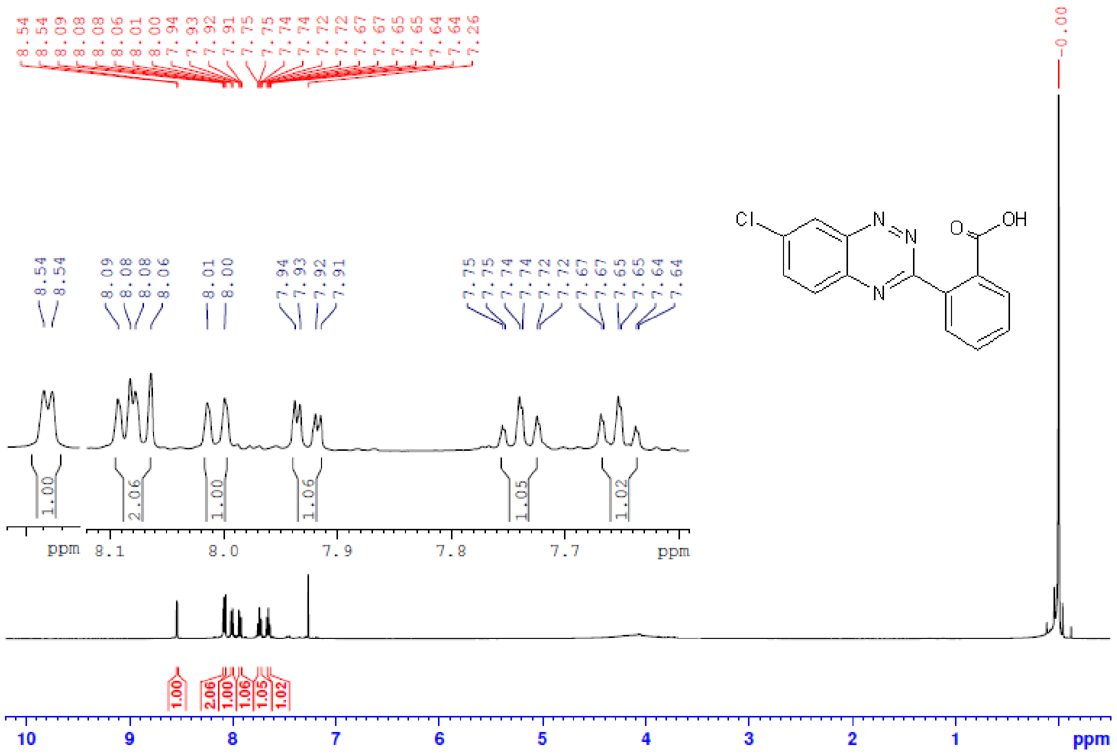

^{1}H-NMR of **17f** in $CDCl_3$ at room temperature.

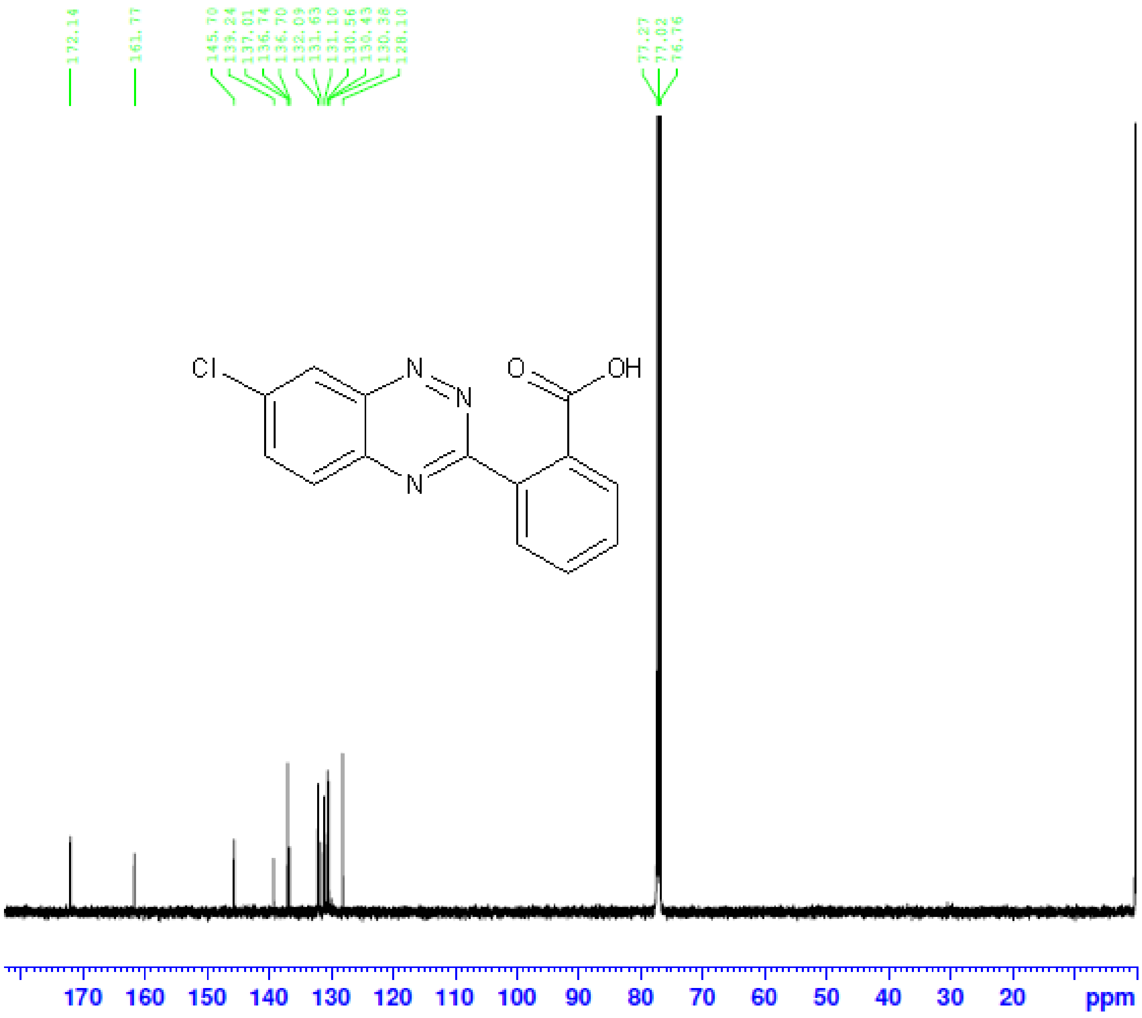

^{13}C-NMR of **17f** in $CDCl_3$ at room temperature.

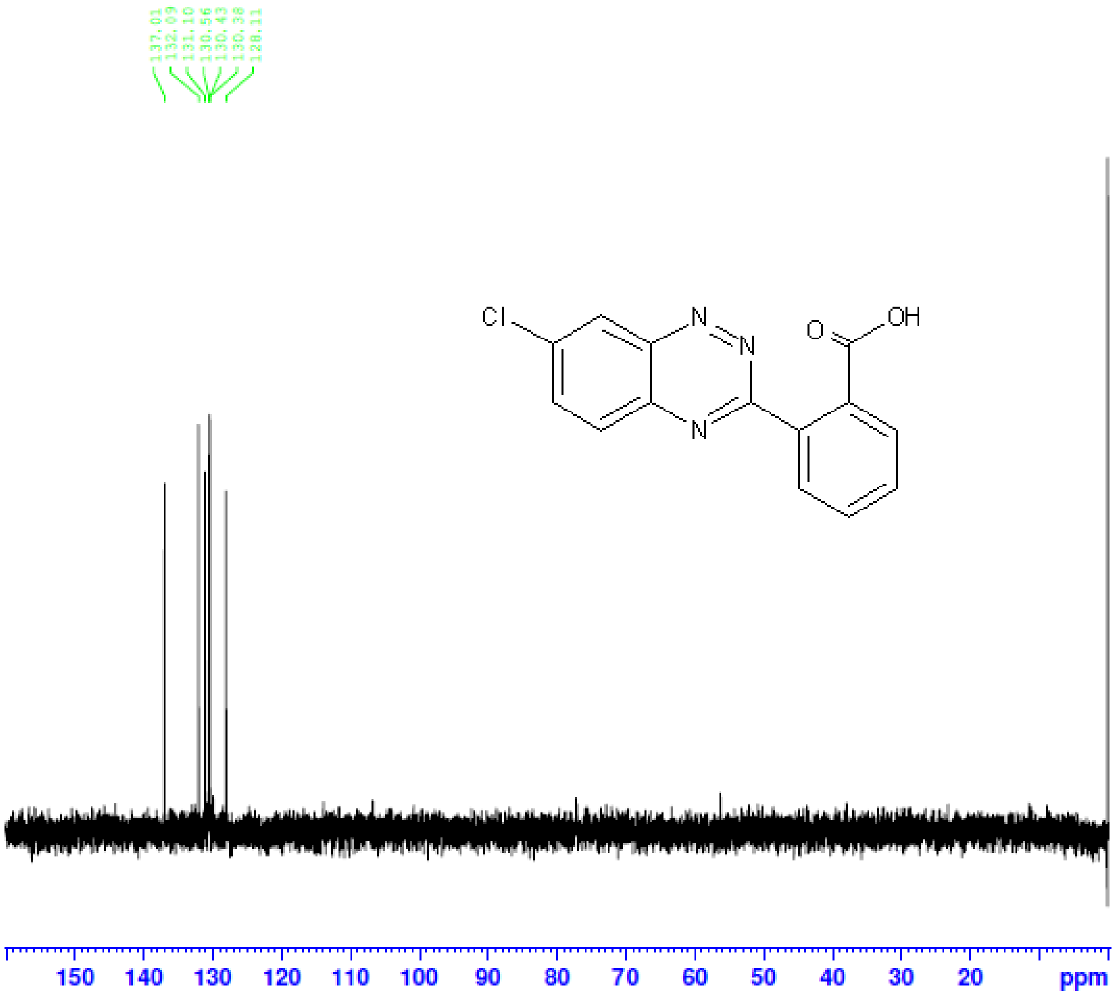

^{13}C DEPT 135-NMR of **17f** in $CDCl_3$ at room temperature.

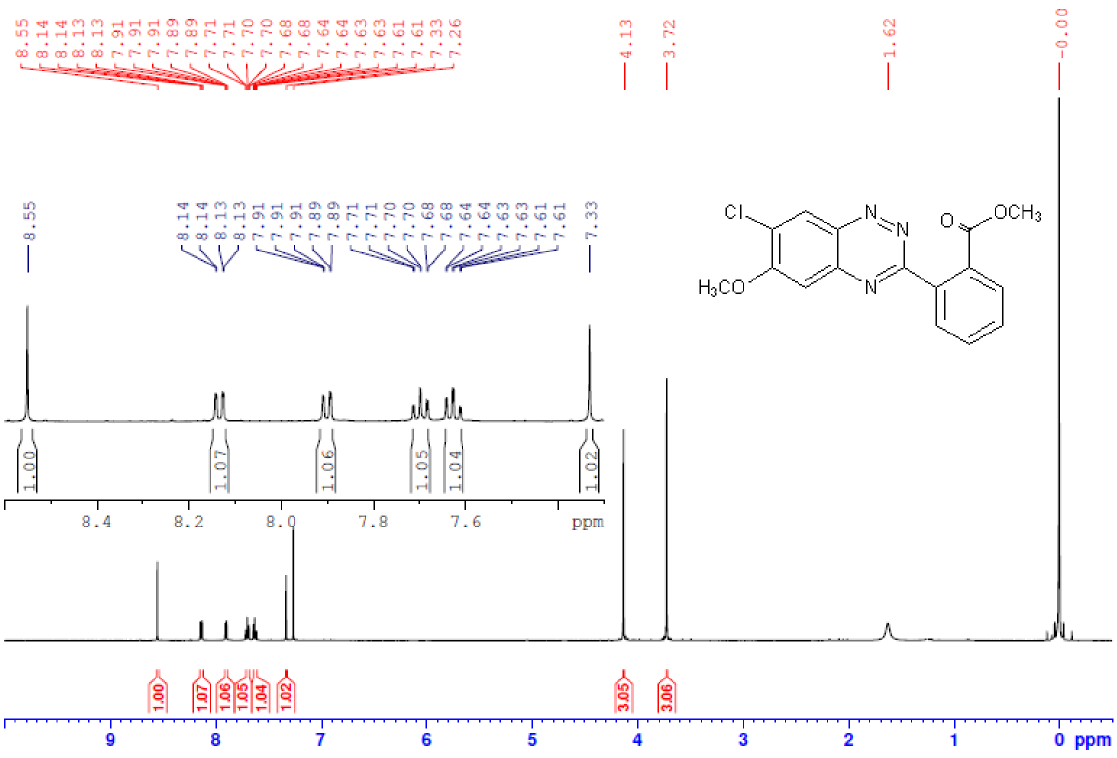

^{1}H-NMR of **16g** in $CDCl_3$ at room temperature.

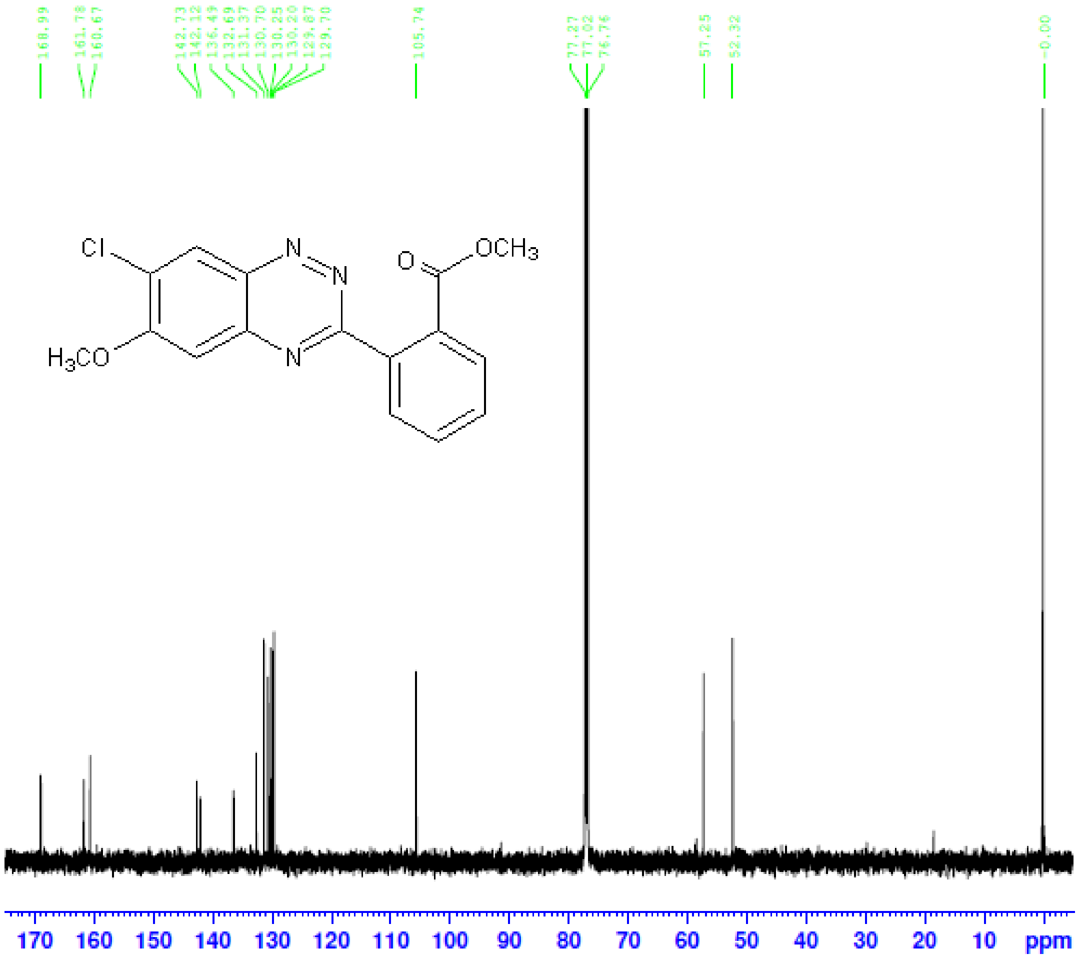

^{13}C-NMR of **16g** in $CDCl_3$ at room temperature.

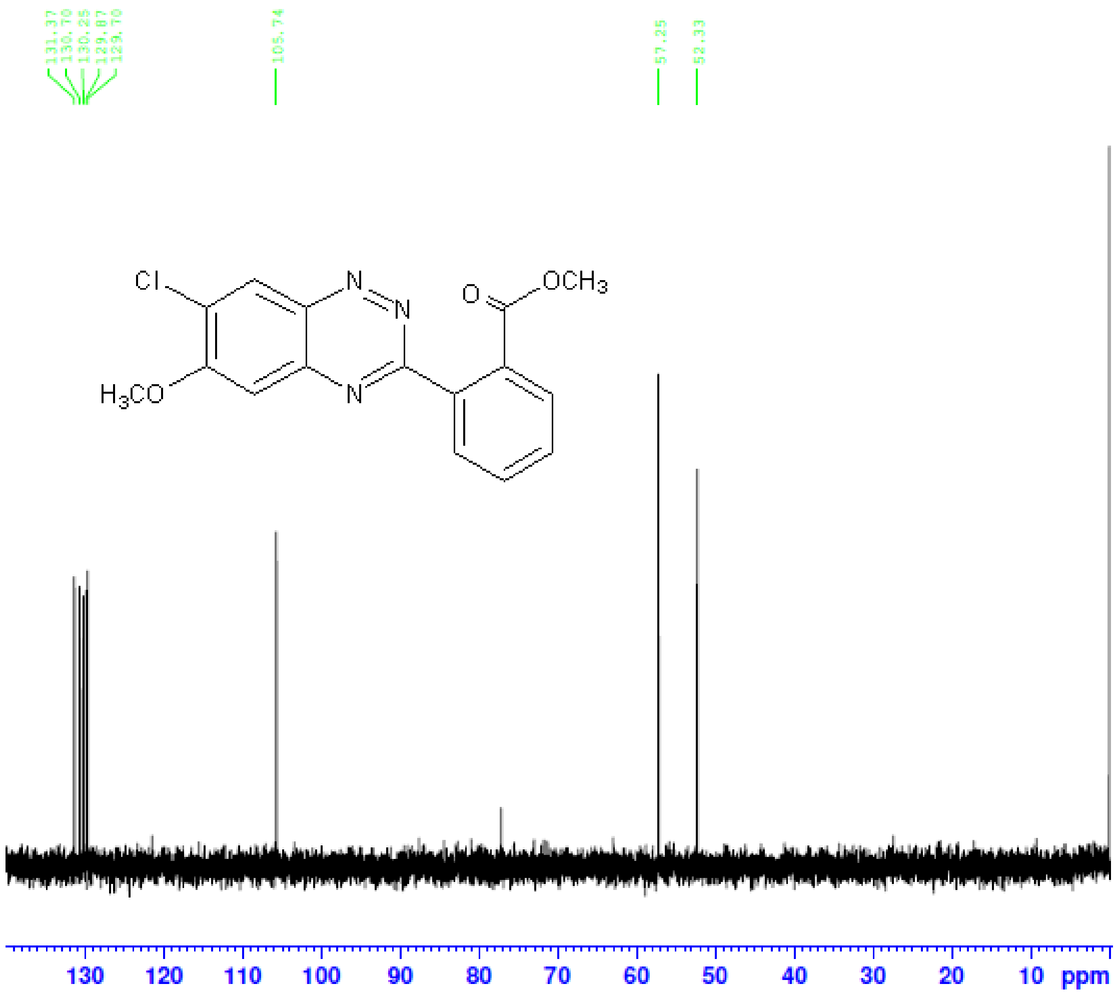

^{13}C DEPT 135-NMR of **16g** in $CDCl_3$ at room temperature.

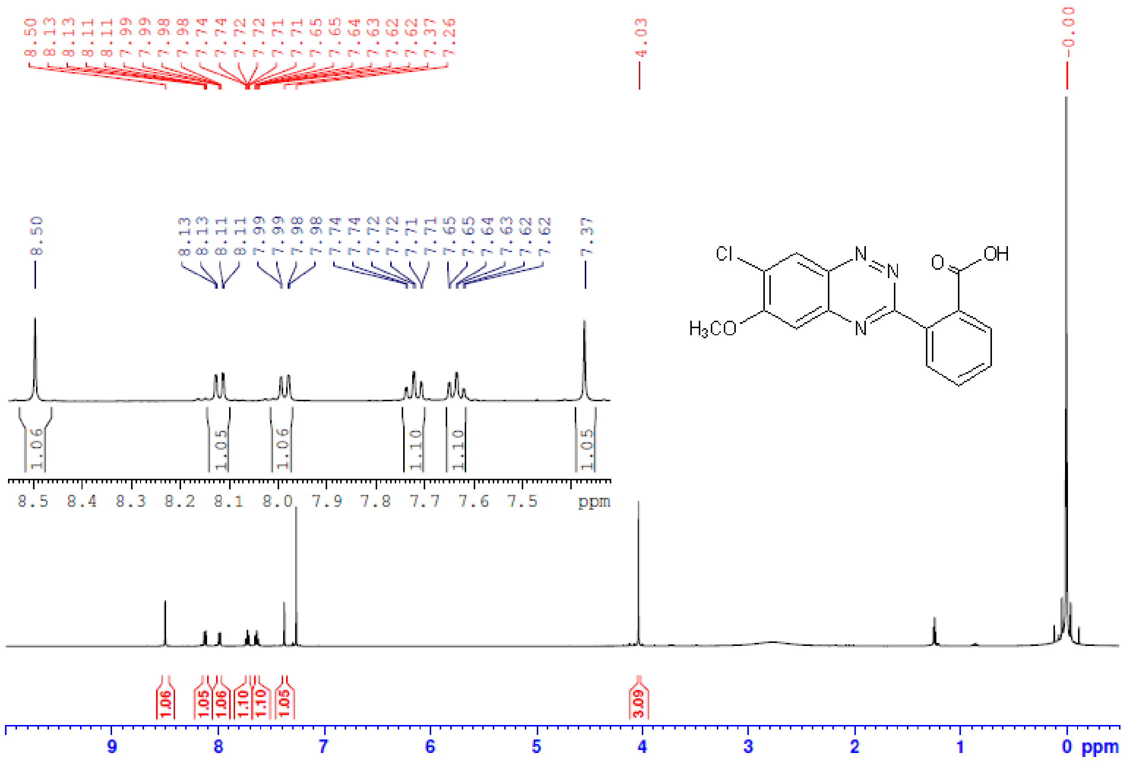

^{1}H-NMR of **17g** in $CDCl_3$ at room temperature.

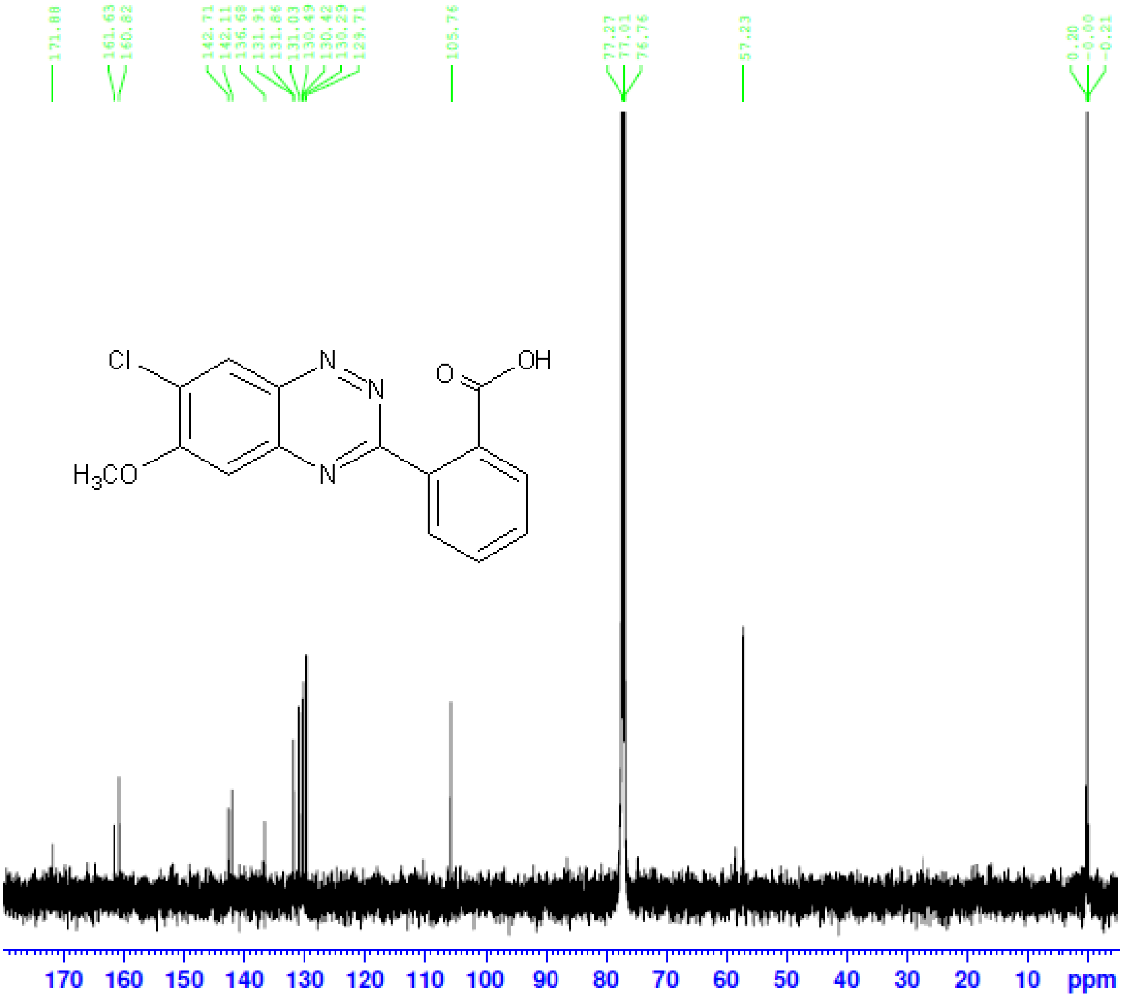

^{13}C-NMR of **17g** in $CDCl_3$ at room temperature.

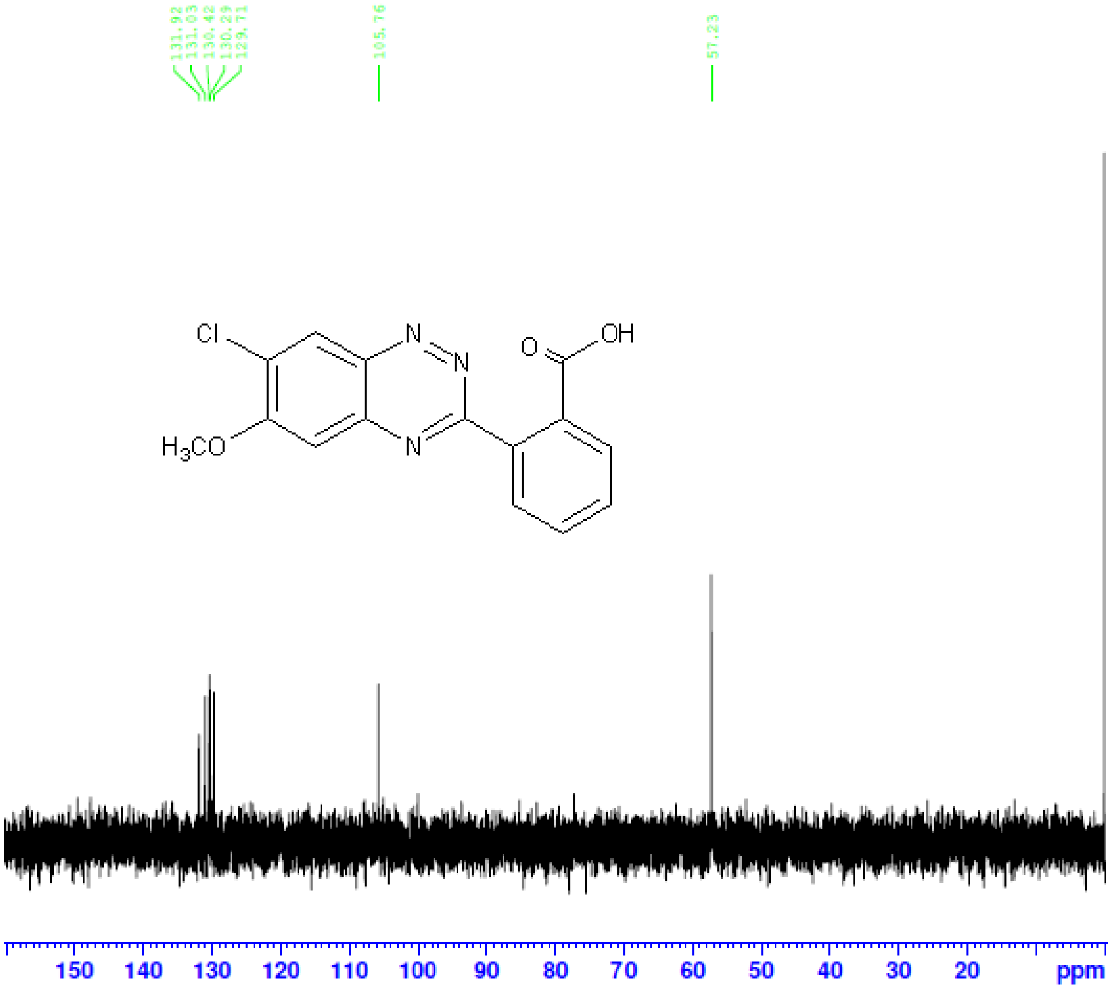

^{13}C DEPT 135-NMR of **17g** in $CDCl_3$ at room temperature.

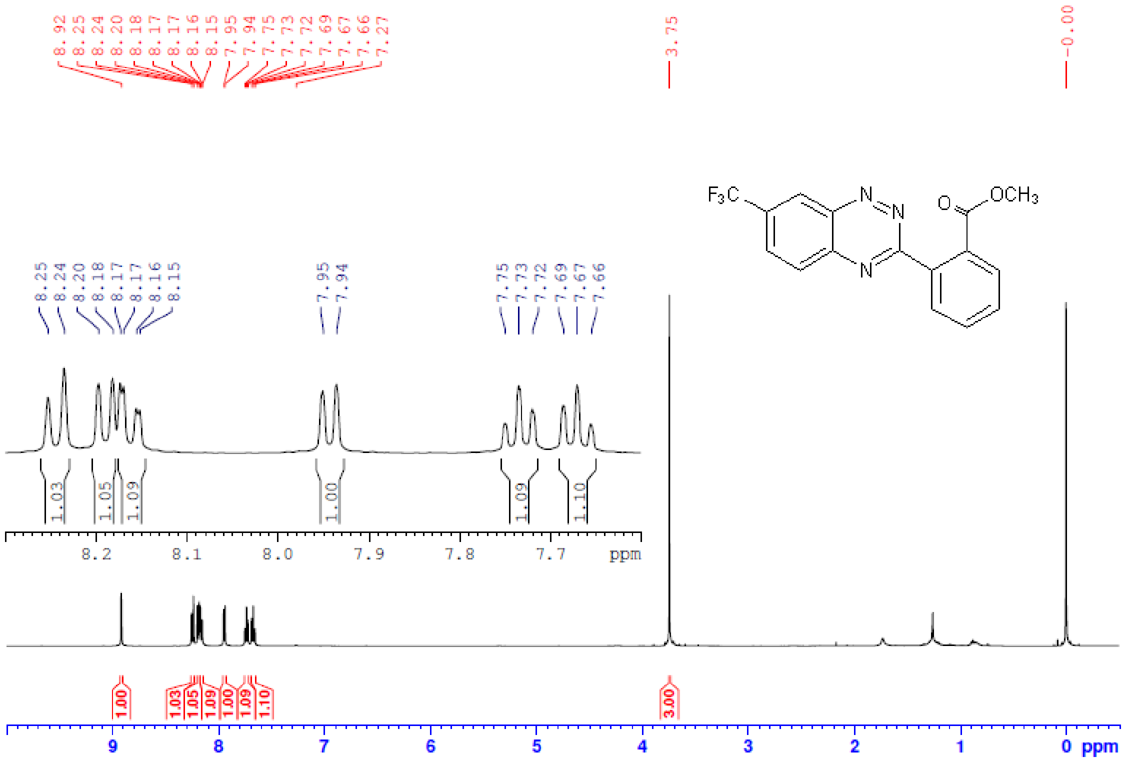

^{1}H-NMR of **16h** in $CDCl_3$ at room temperature.

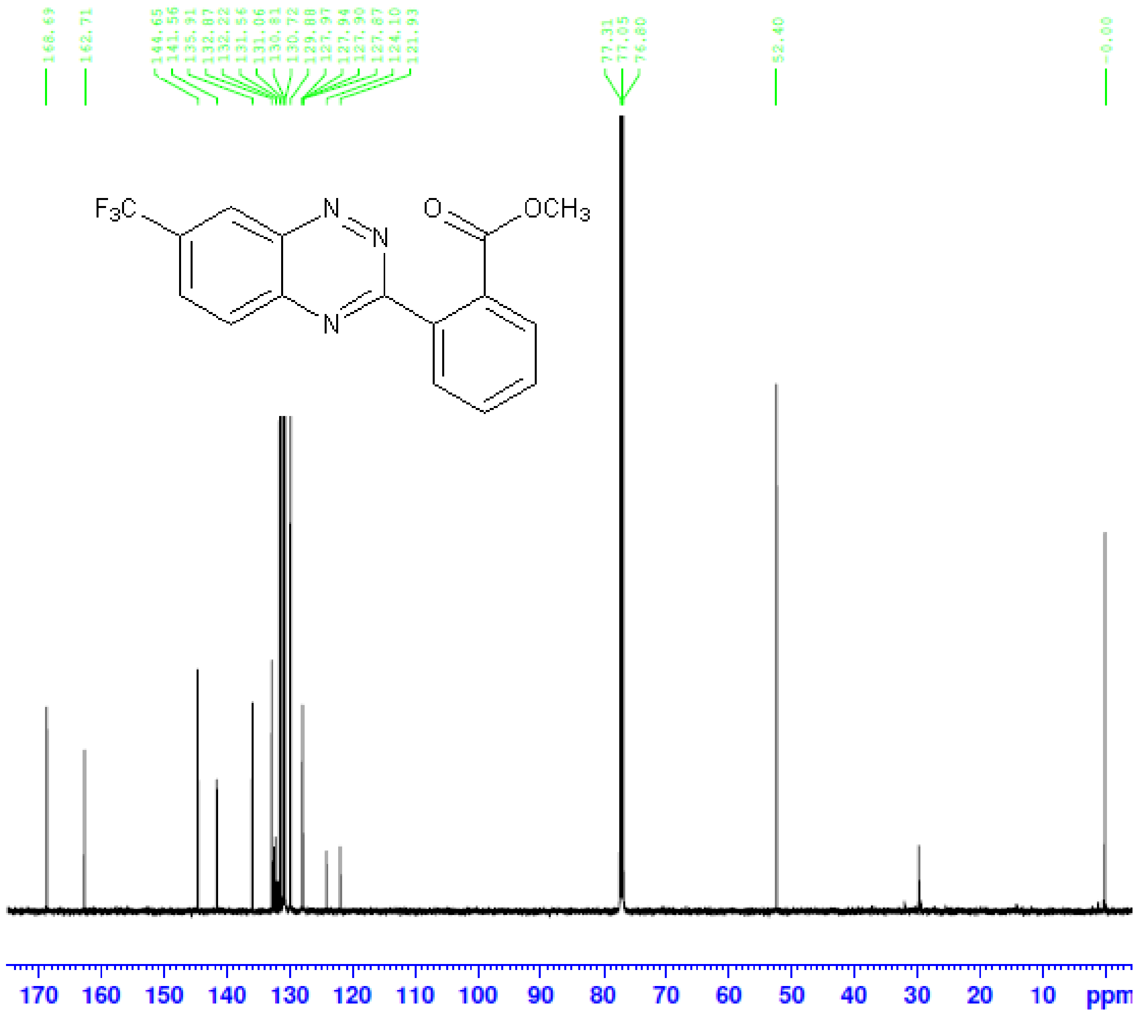

^{13}C-NMR of **16h** in $CDCl_3$ at room temperature.

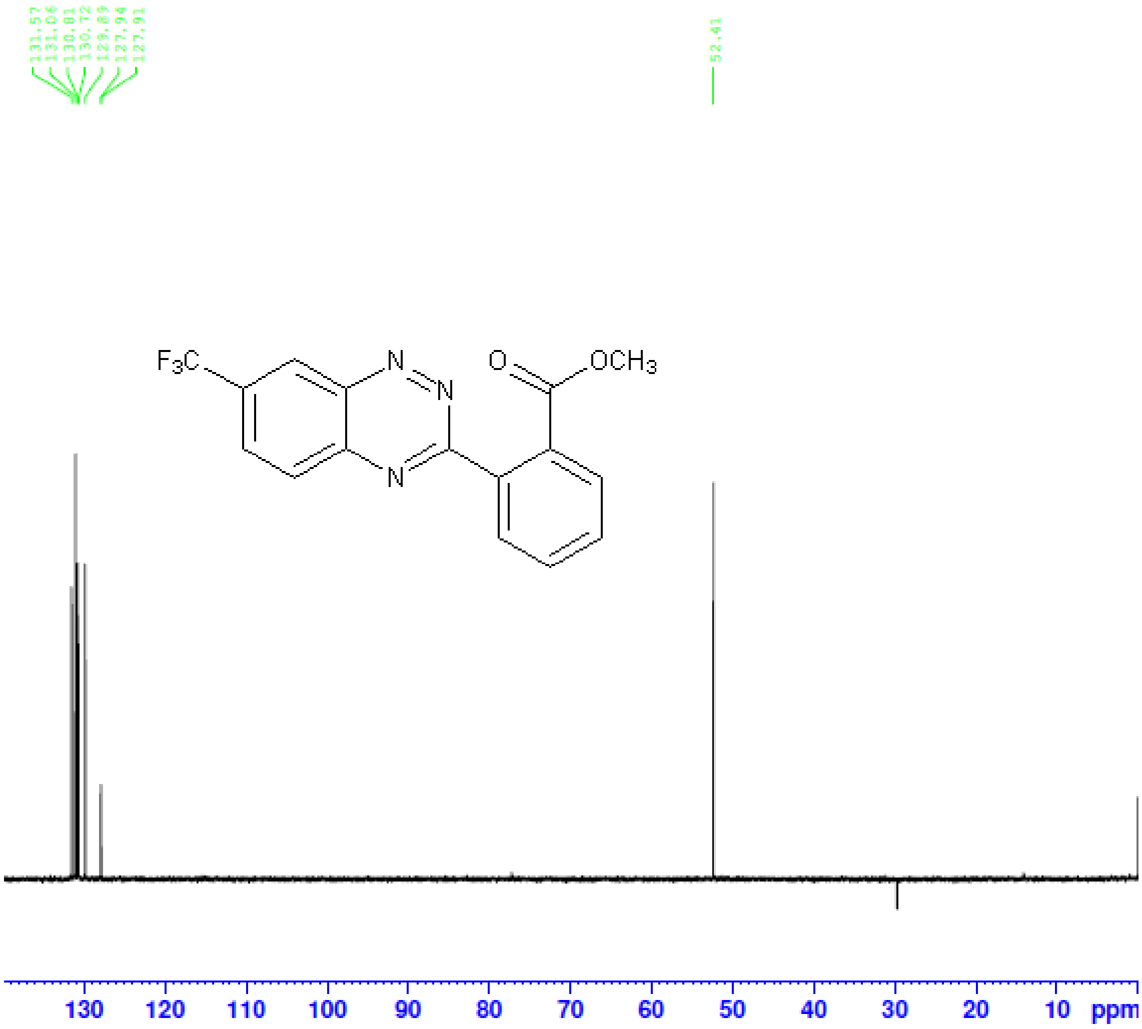

^{13}C DEPT 135-NMR of **16h** in $CDCl_3$ at room temperature.

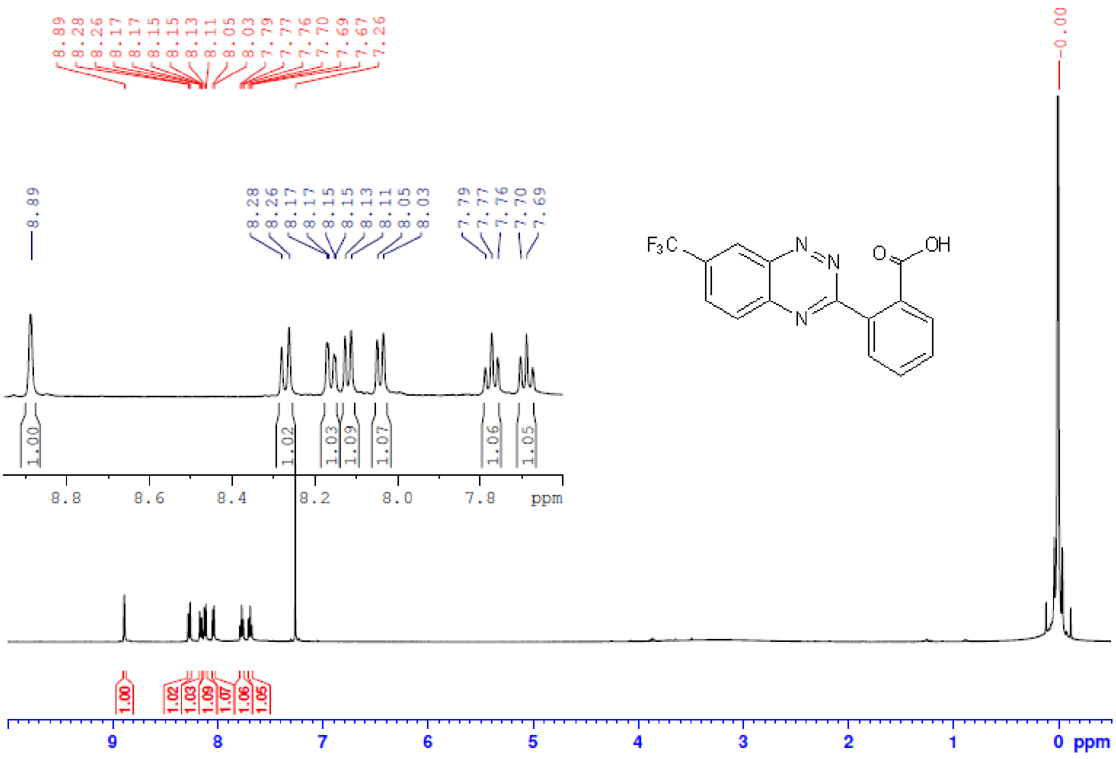

^{1}H-NMR of **17h** in $CDCl_3$ at room temperature.

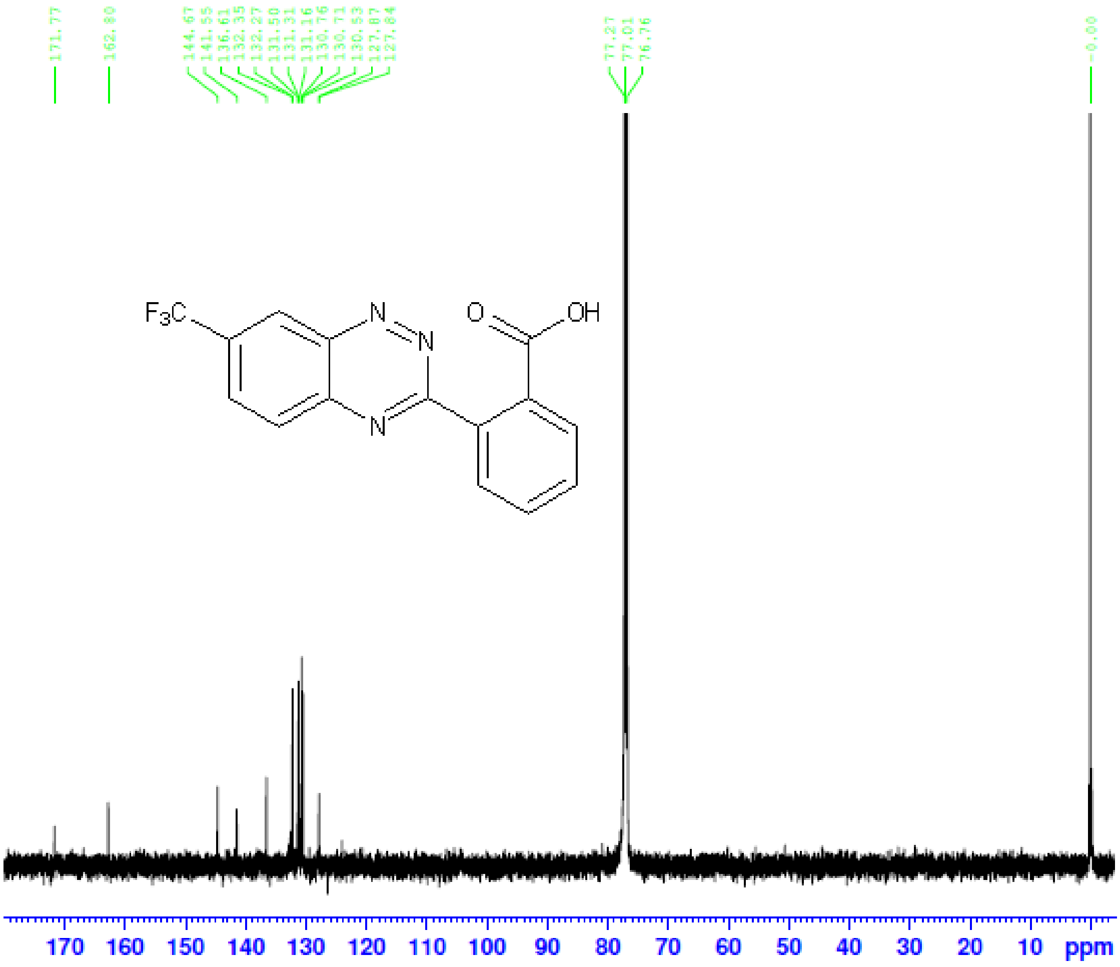

^{13}C-NMR of **17h** in $CDCl_3$ at room temperature.

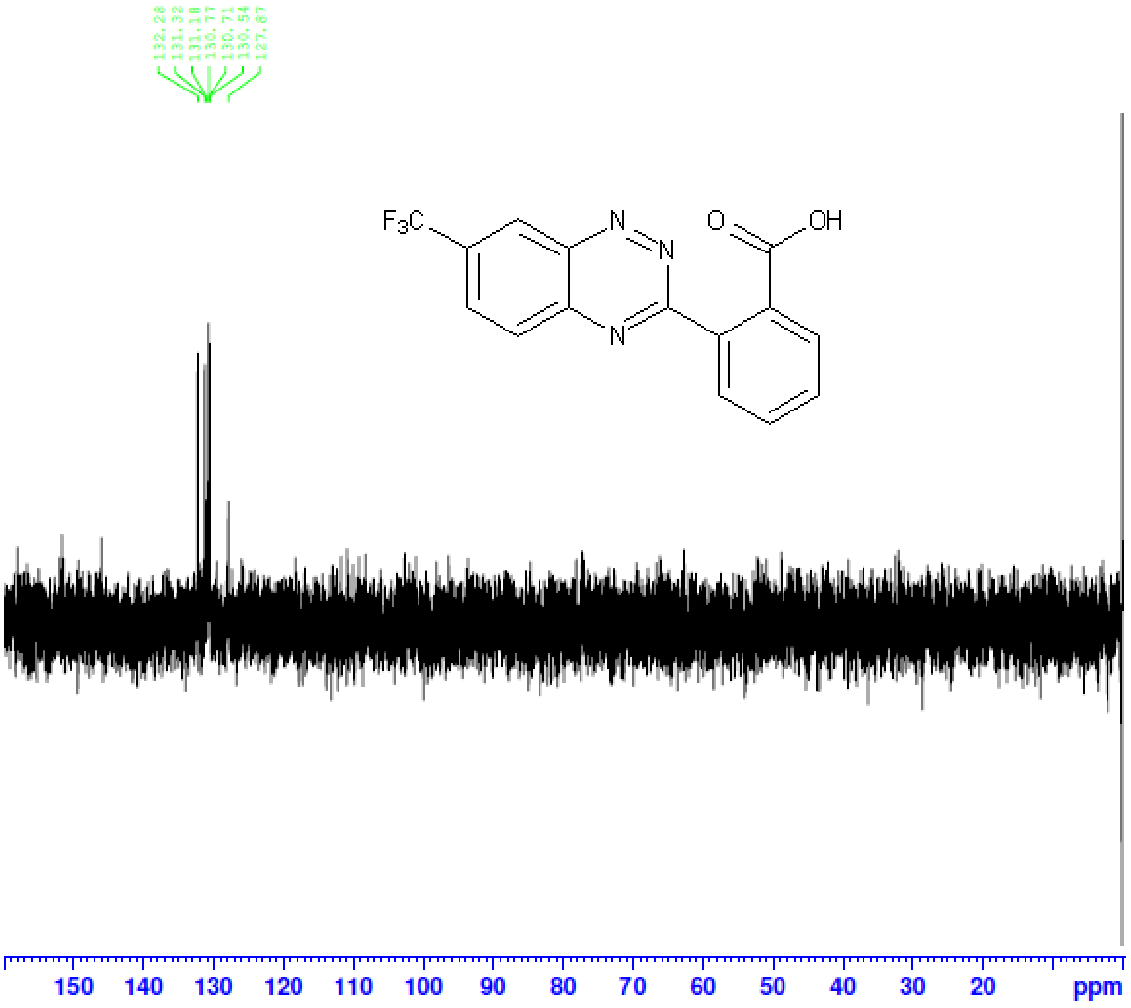

^{13}C DEPT 135-NMR of **17h** in $CDCl_3$ at room temperature.

APPENDIX B: IR SPECTROSCOPY

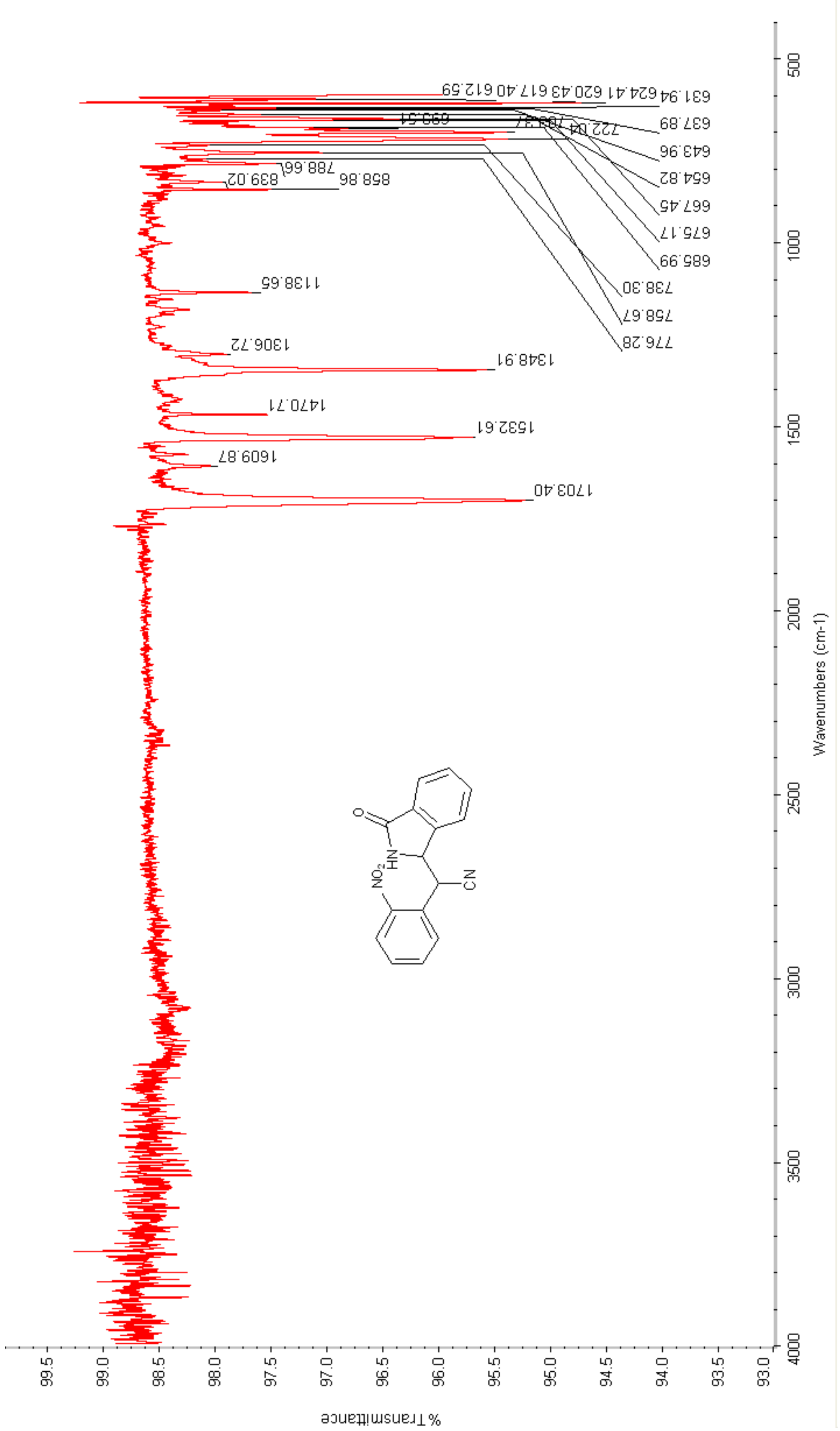

IR of **6**

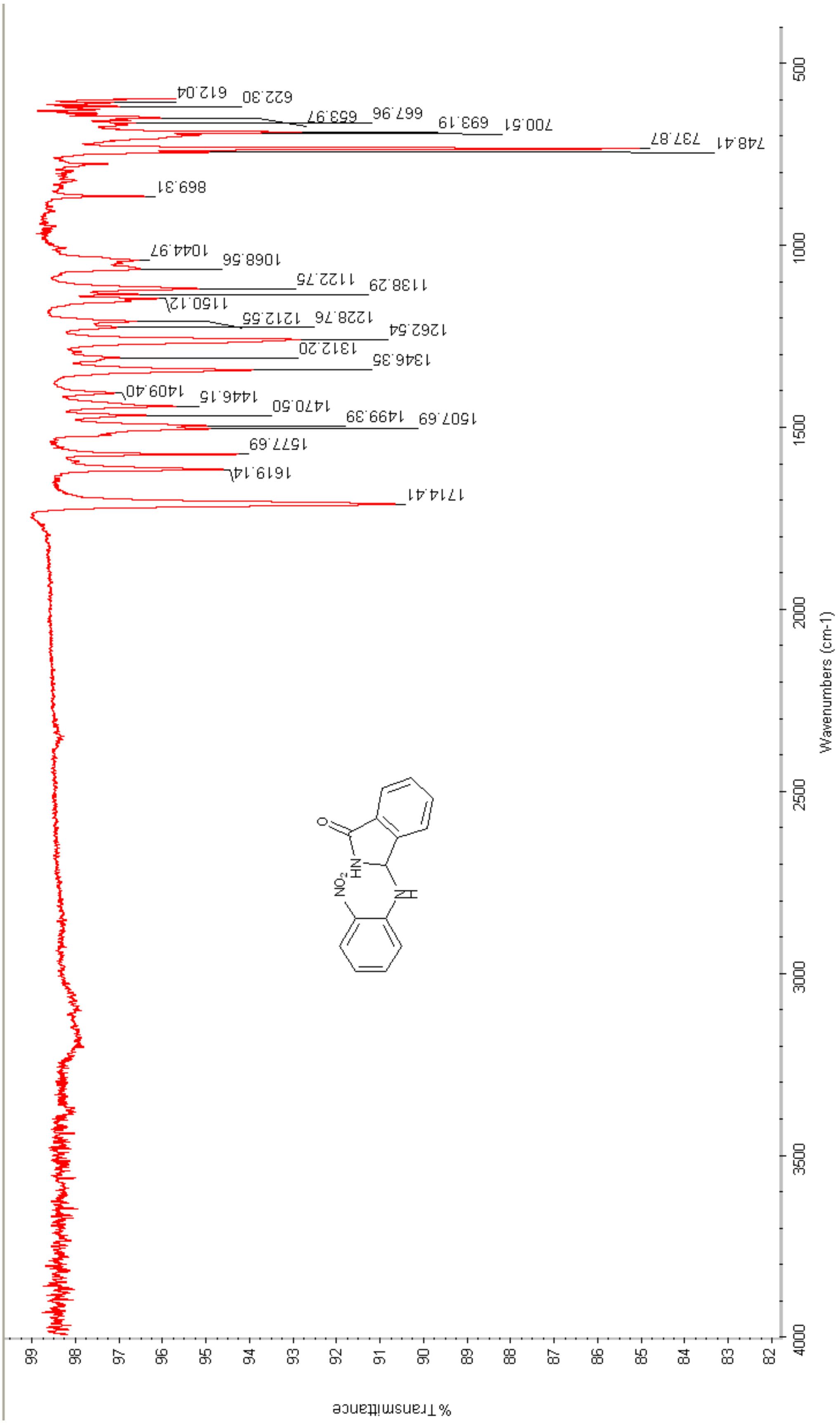

612.04
622.30
653.97
667.96
693.19
700.51
737.87
748.41
869.31
1044.97
1068.56
1122.75
1138.29
1150.12
1212.55
1228.76
1262.54
1312.20
1346.35
1409.40
1446.15
1470.50
1499.39
1507.69
1577.69
1619.14
1714.41
%Transmittance
Wavenumbers (cm-1)

IR of **10a**

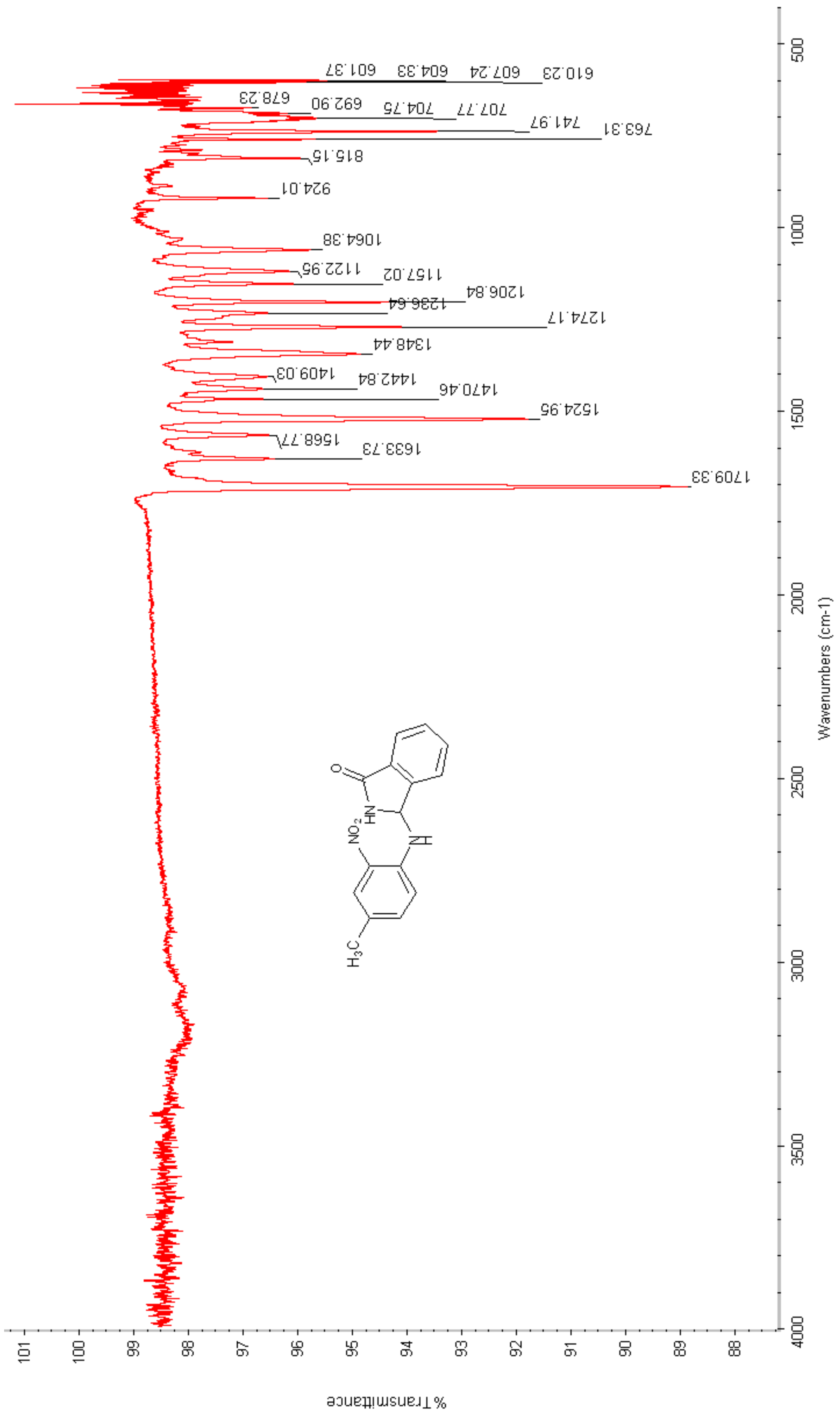
610.23
607.24
604.33
601.37
678.23
692.90
704.75
707.77
741.97
763.31
815.15
924.01
1064.38
1122.95
1157.02
1206.84
1236.64
1274.17
1348.44
1409.03
1442.84
1470.46
1524.95
1568.77
1633.73
1709.33
Wavenumbers (cm-1)
%Transmittance

IR of **10b**

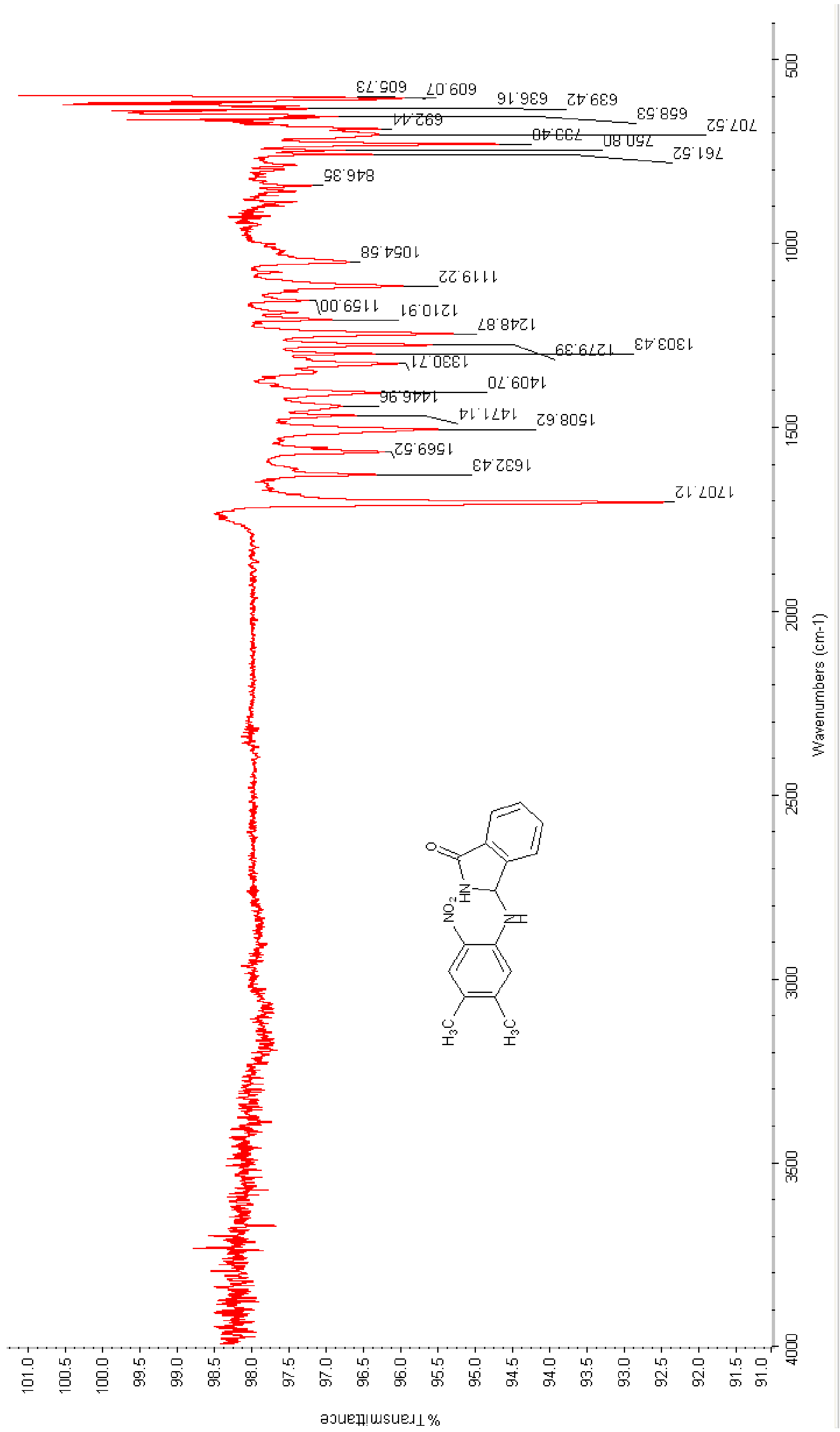

%Transmittance
Wavenumbers (cm-1)
1707.12
1632.43
1569.52
1508.62
1471.14
1446.96
1409.70
1330.71
1303.43
1279.39
1248.87
1210.91
1159.00
1119.22
1054.58
846.35
761.52
750.80
733.40
707.52
692.44
658.53
639.42
636.16
609.07
605.73

IR of **10c**

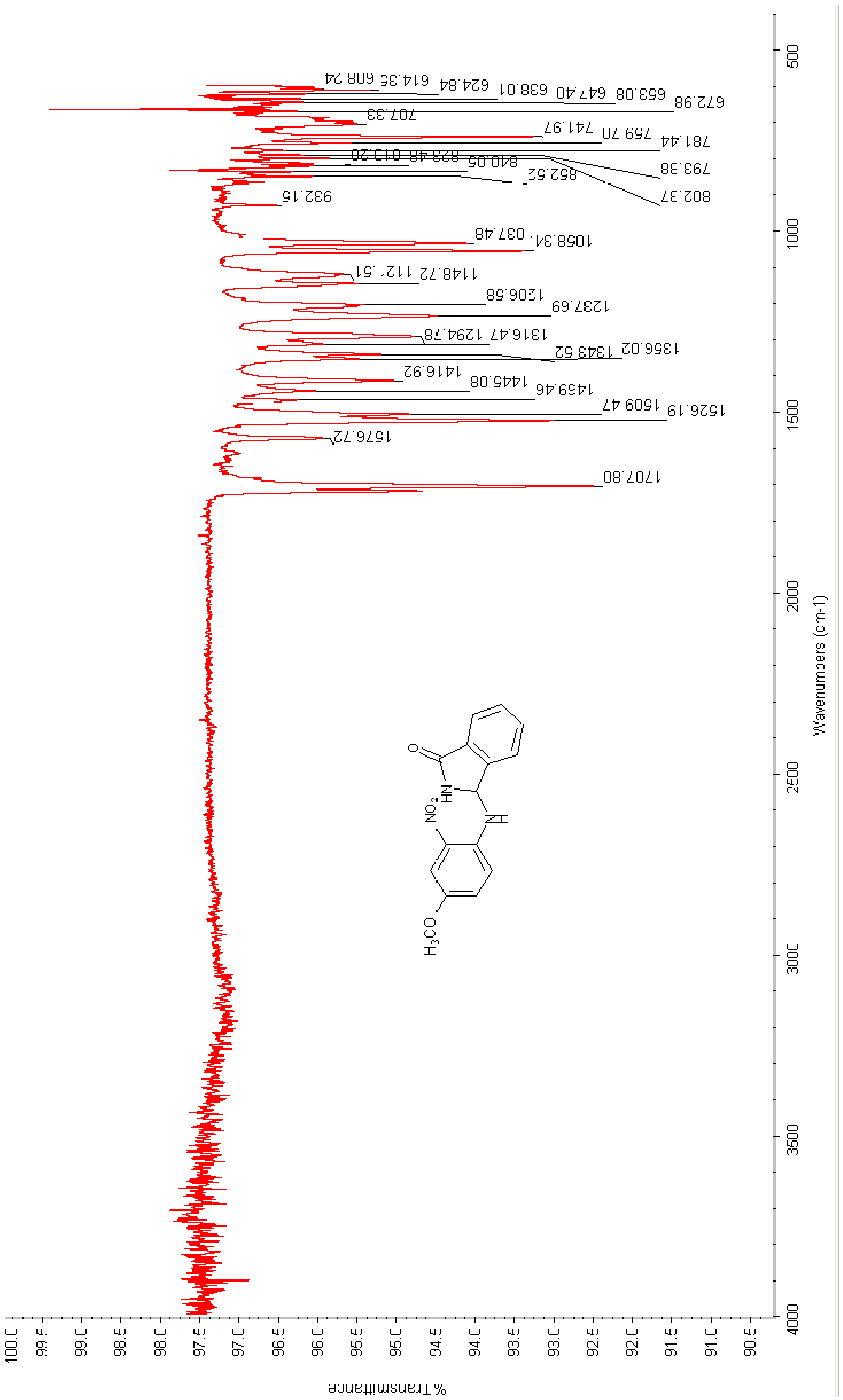

%Transmittance
Wavenumbers (cm-1)
608.24
614.35
624.84
638.01
647.40
653.08
672.98
707.33
741.97
759.70
781.44
793.88
802.37
810.20
823.48
840.05
852.52
932.15
1037.48
1058.34
1121.51
1148.72
1206.58
1237.69
1294.78
1316.47
1343.52
1356.02
1416.92
1445.08
1469.46
1509.47
1526.19
1576.72
1707.80

IR of **10d**

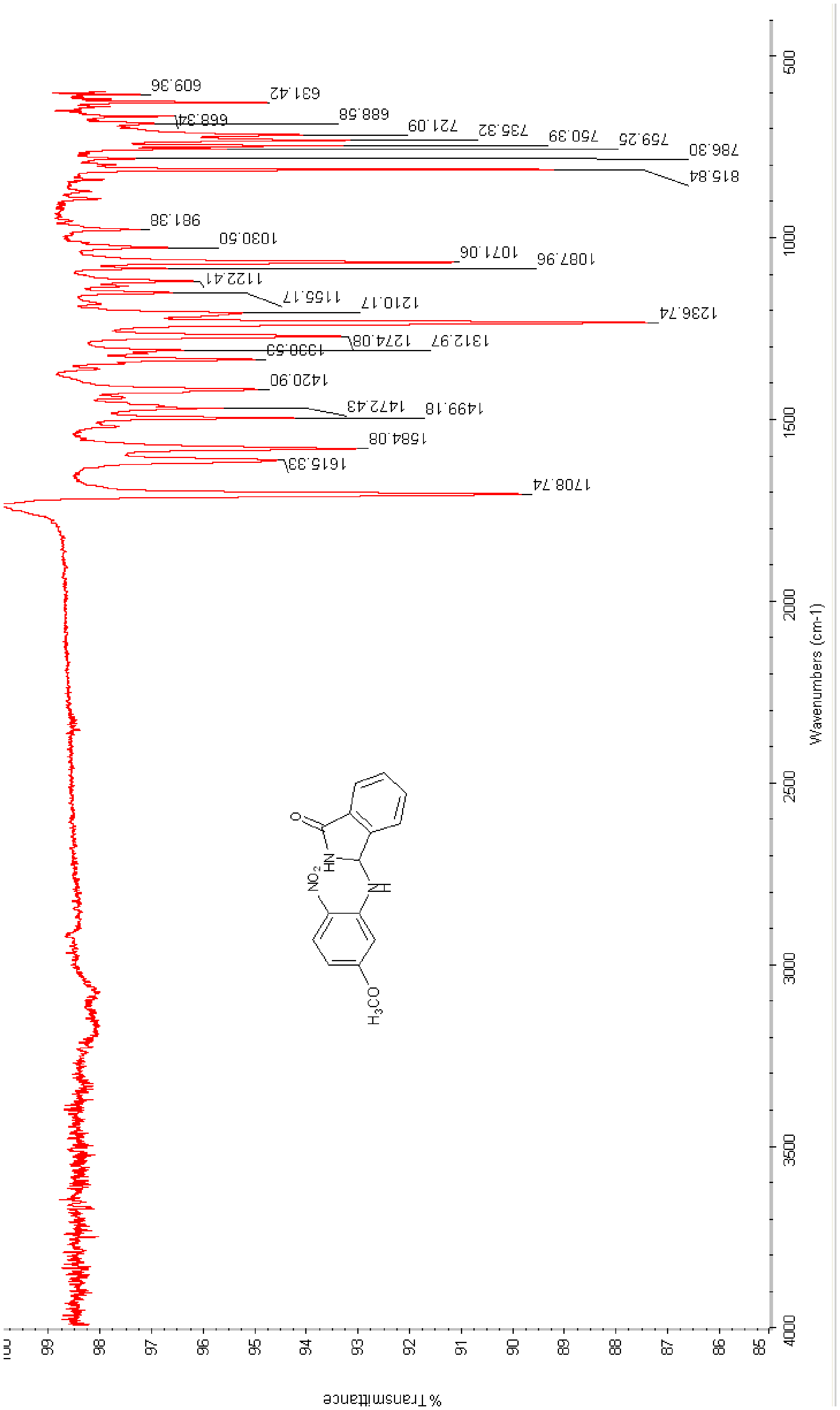
609.36
631.42
668.34
688.58
721.09
735.32
750.39
759.25
786.30
815.84
981.38
1030.50
1071.06
1087.96
1122.41
1155.17
1210.17
1236.74
1274.08
1312.97
1330.53
1420.90
1472.43
1499.18
1584.08
1615.33
1708.74
Wavenumbers (cm-1)
%Transmittance

IR of **10e**

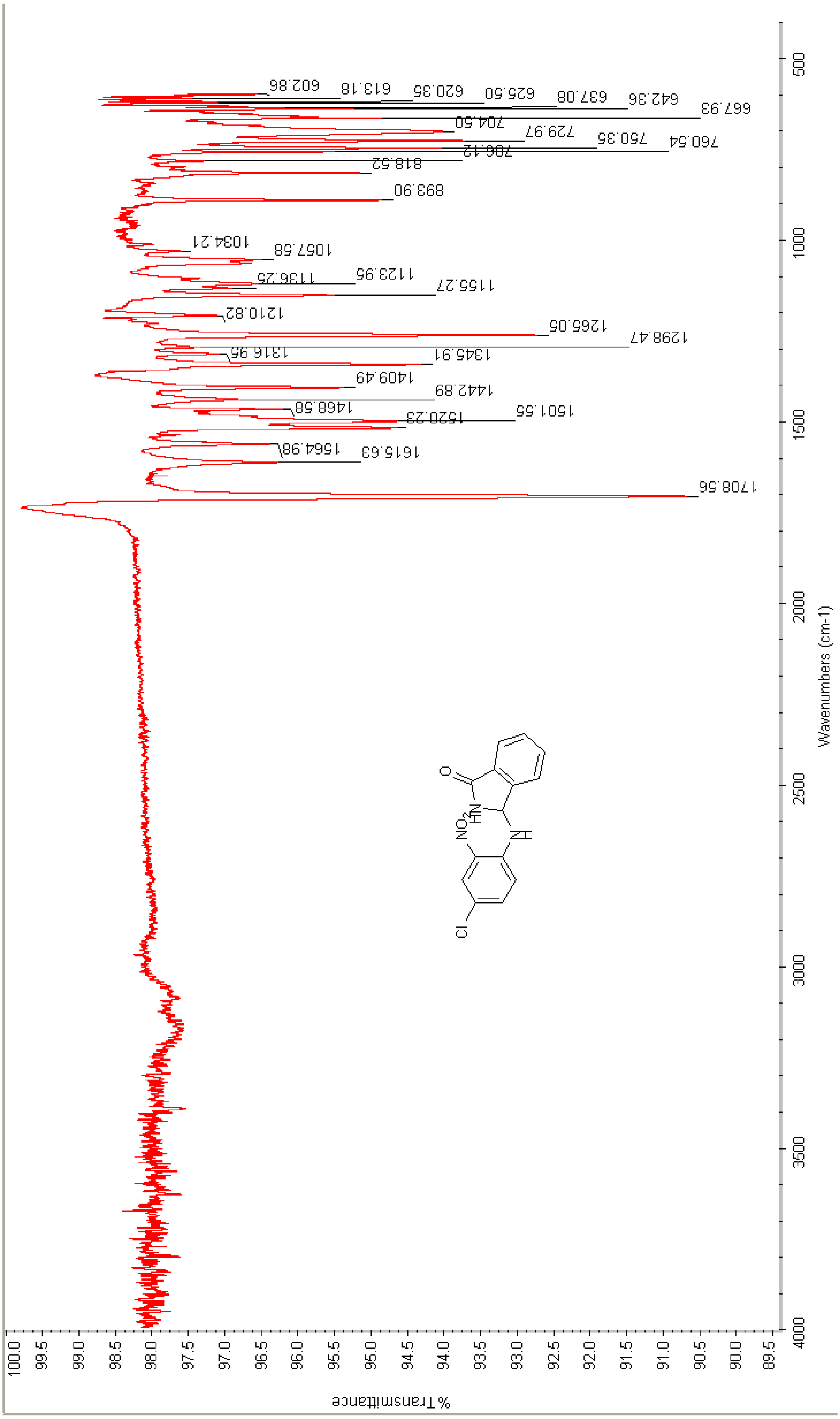

%Transmittance
Wavenumbers (cm-1)
1708.56
1615.63
1564.98
1520.23
1501.55
1468.58
1442.89
1409.49
1345.91
1316.95
1298.47
1265.05
1210.82
1155.27
1136.25
1123.95
1057.58
1034.21
893.90
818.52
796.12
760.54
750.35
729.97
704.50
667.93
642.36
637.08
625.50
620.35
613.18
602.86

IR of **10f**

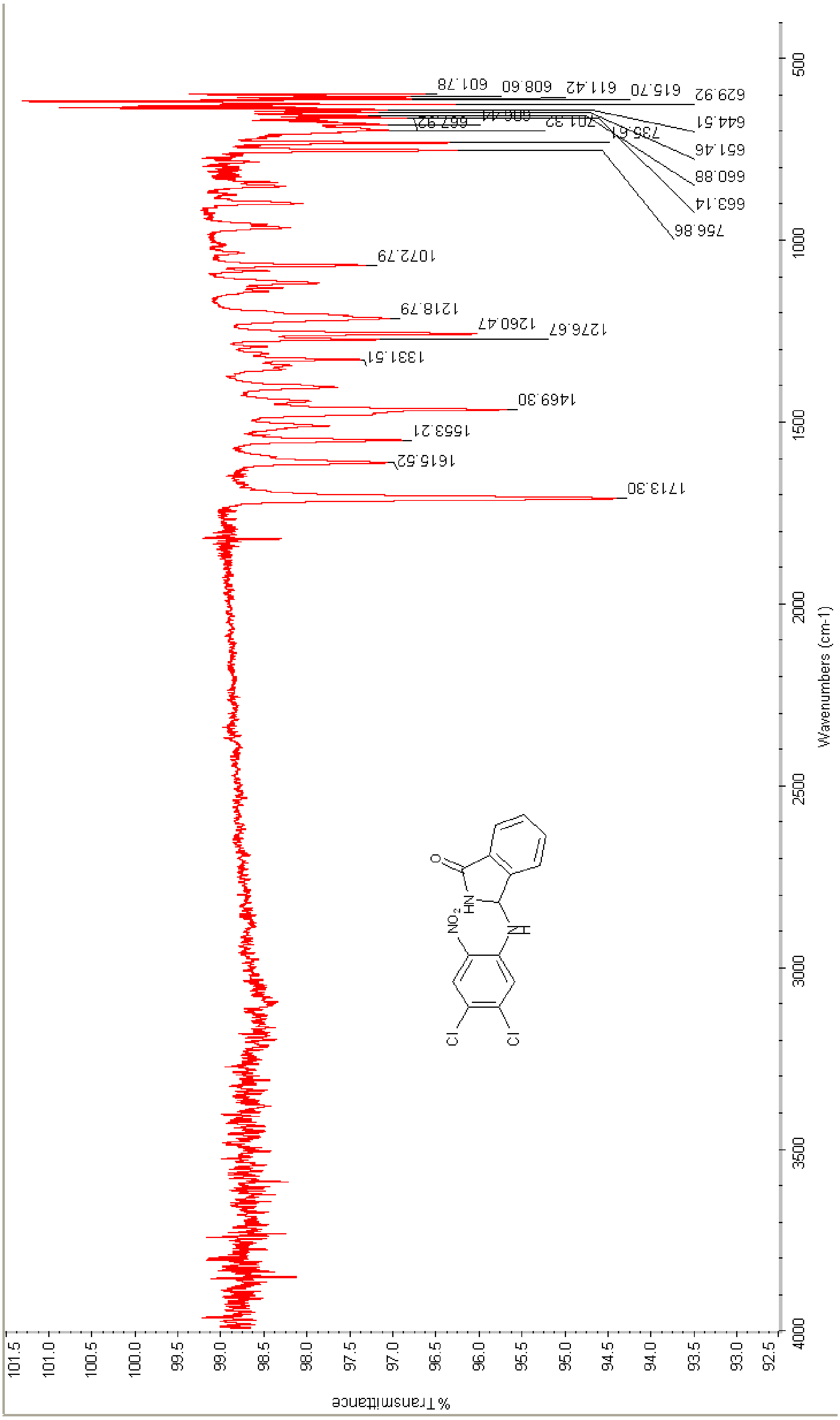
%Transmittance
Wavenumbers (cm-1)
1713.30
1615.52
1553.21
1469.30
1331.51
1276.67
1260.47
1218.79
1072.79
756.86
735.61
701.32
667.92
663.14
660.88
651.46
644.51
629.92
615.70
611.42
608.60
601.78
Cl
Cl
NO2
HN
H
O

IR of **10g**

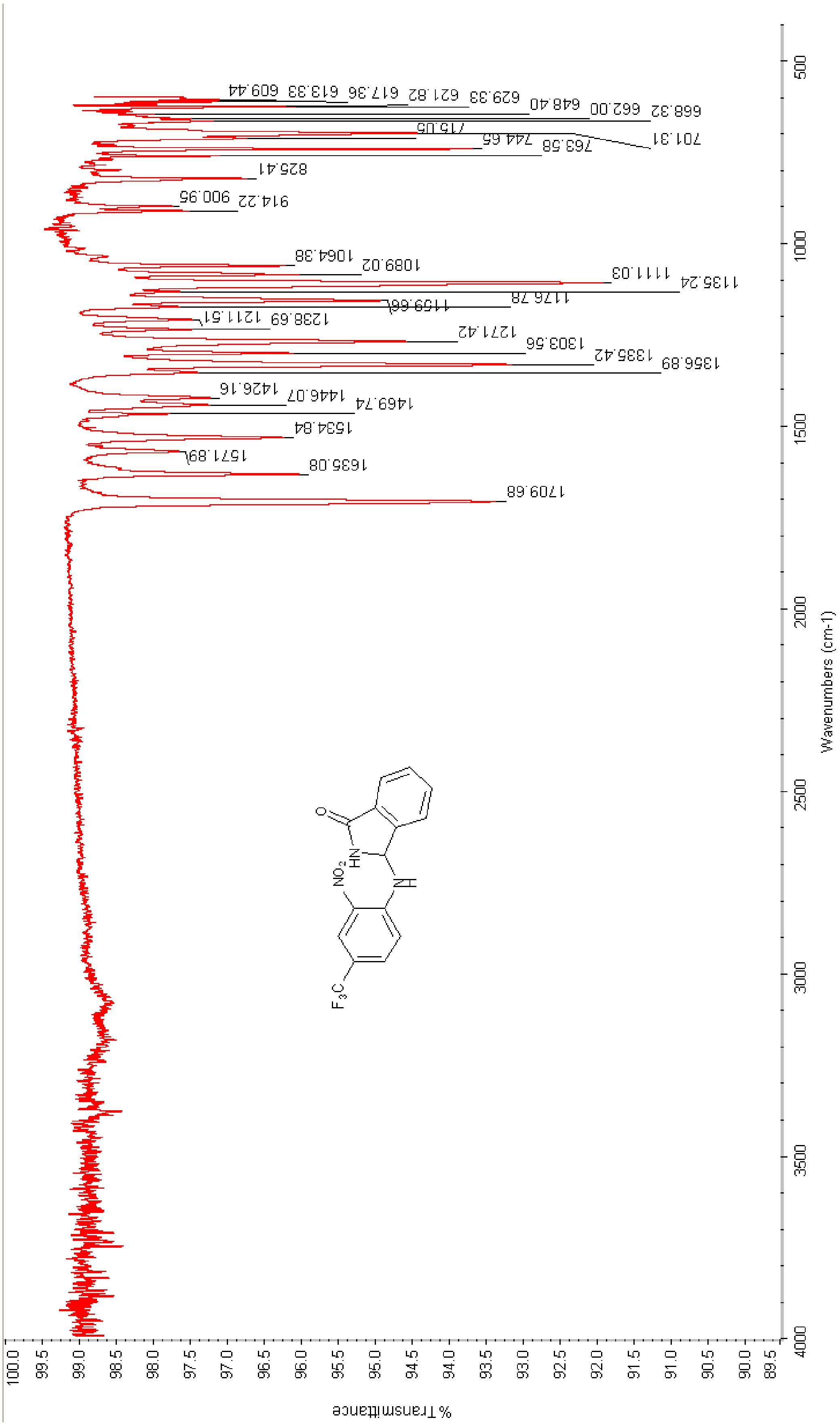
%Transmittance
Wavenumbers (cm-1)
1709.68
1635.08
1571.89
1534.84
1469.74
1446.07
1426.16
1356.89
1335.42
1303.56
1271.42
1238.69
1211.51
1176.78
1159.66
1135.24
1111.03
1089.02
1064.38
914.22
900.95
825.41
763.58
744.65
715.05
701.31
668.32
662.00
648.40
629.33
621.82
617.36
613.33
609.44

IR of **10h**

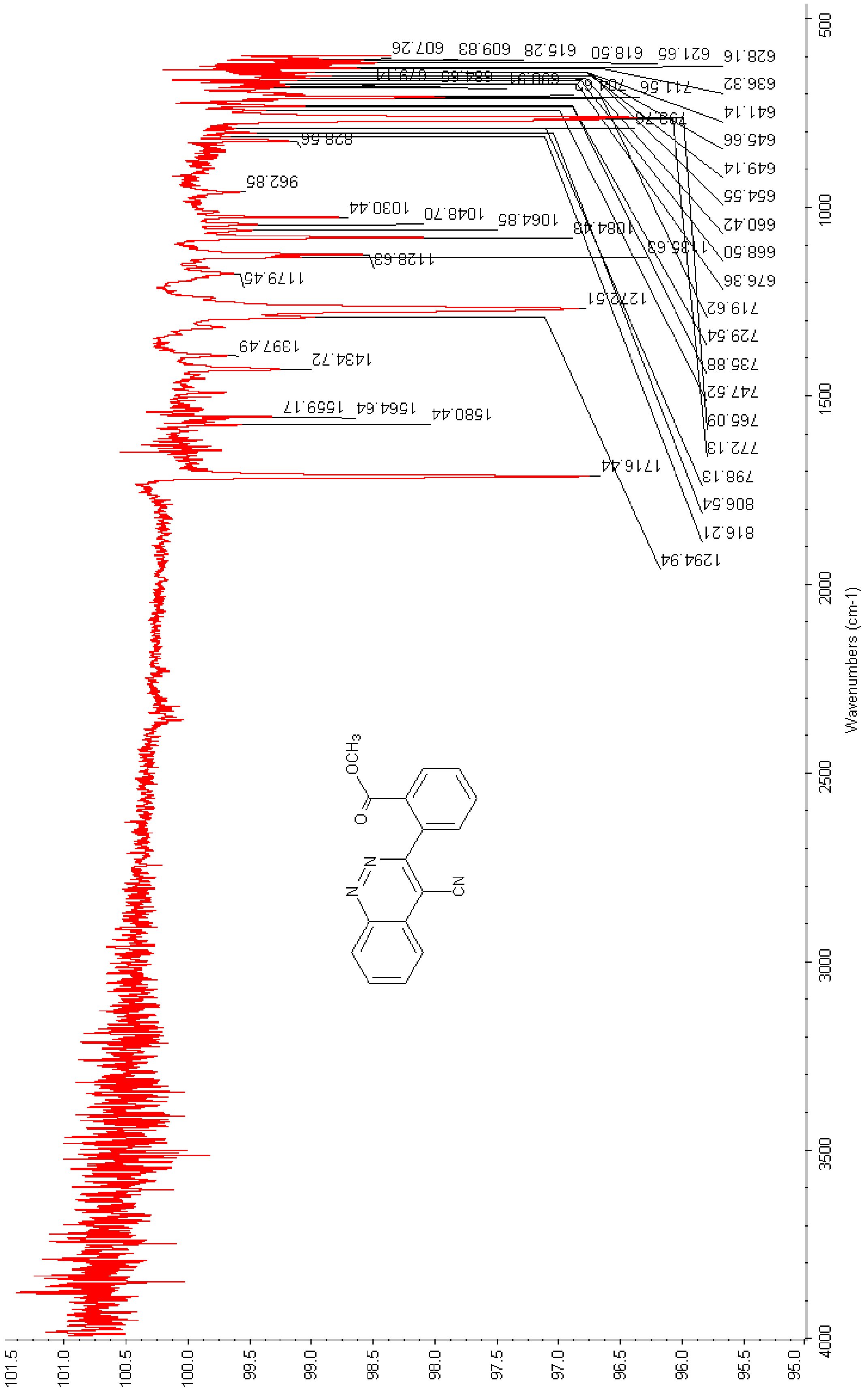

%Transmittance
Wavenumbers (cm-1)
OCH3
O
N
N
CN
1716.44
1580.44
1564.64
1559.17
1434.72
1397.49
1294.94
1272.51
1179.45
1128.63
1084.48
1064.85
1048.70
1030.44
962.85
828.56
816.21
806.54
798.13
772.13
765.09
747.52
735.88
729.54
719.62
676.36
668.50
660.42
654.55
649.14
645.66
641.14
636.32
628.16
621.65
618.50
615.28
609.83
607.26

IR of **14**

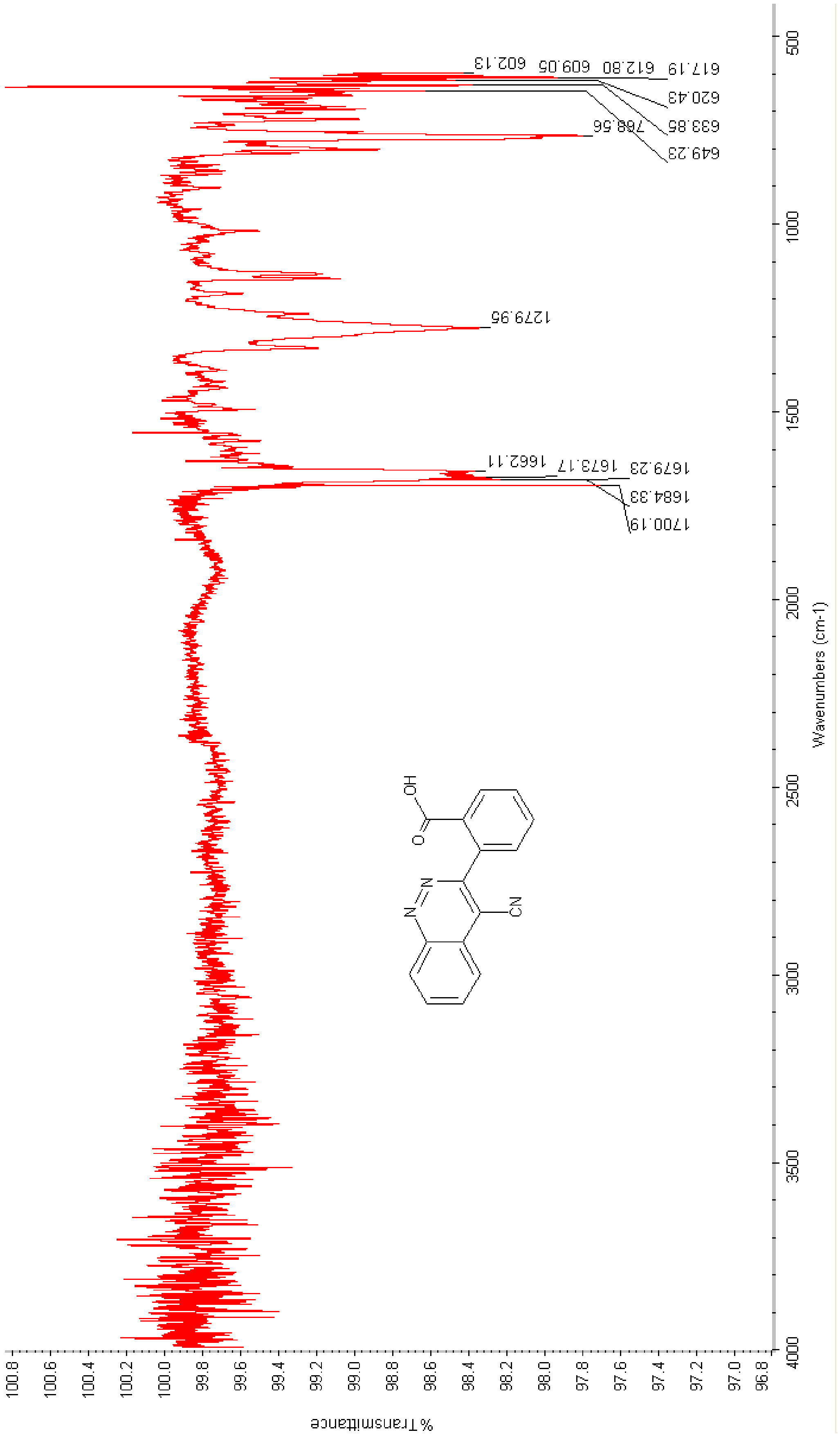

602.13
609.05
612.80
617.19
620.43
633.85
768.56
649.23
1279.95
1662.11
1673.17
1679.23
1684.33
1700.19
CN
N
N
O
OH
Wavenumbers (cm-1)
% Transmittance

IR of **15**

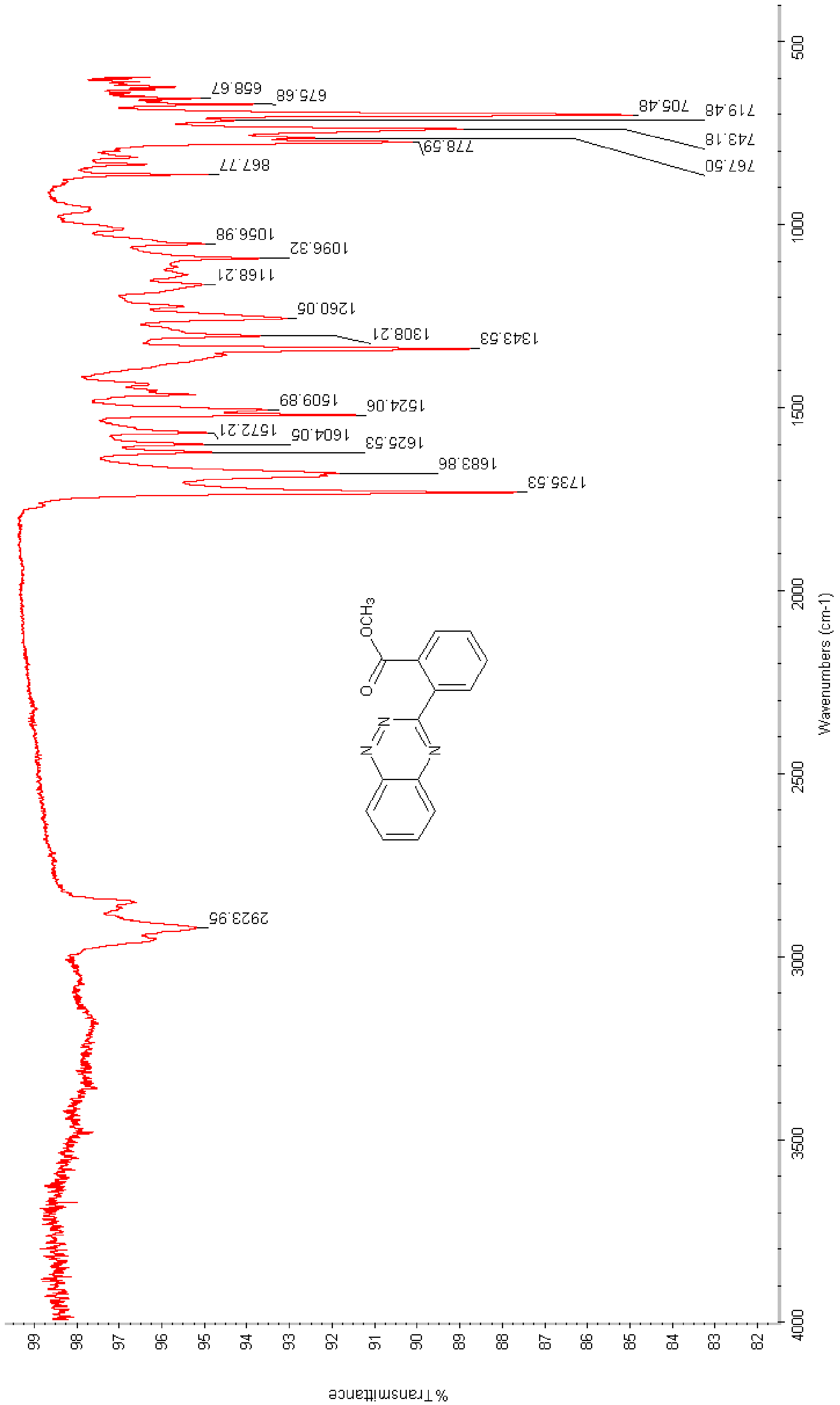
658.67
675.68
705.48
719.48
743.18
767.50
778.59
867.77
1056.98
1096.32
1168.21
1260.05
1308.21
1343.53
1509.89
1524.06
1572.21
1604.05
1625.53
1683.86
1735.53
2923.95
Wavenumbers (cm-1)
%Transmittance
OCH3
O
N
N

IR of **16a**

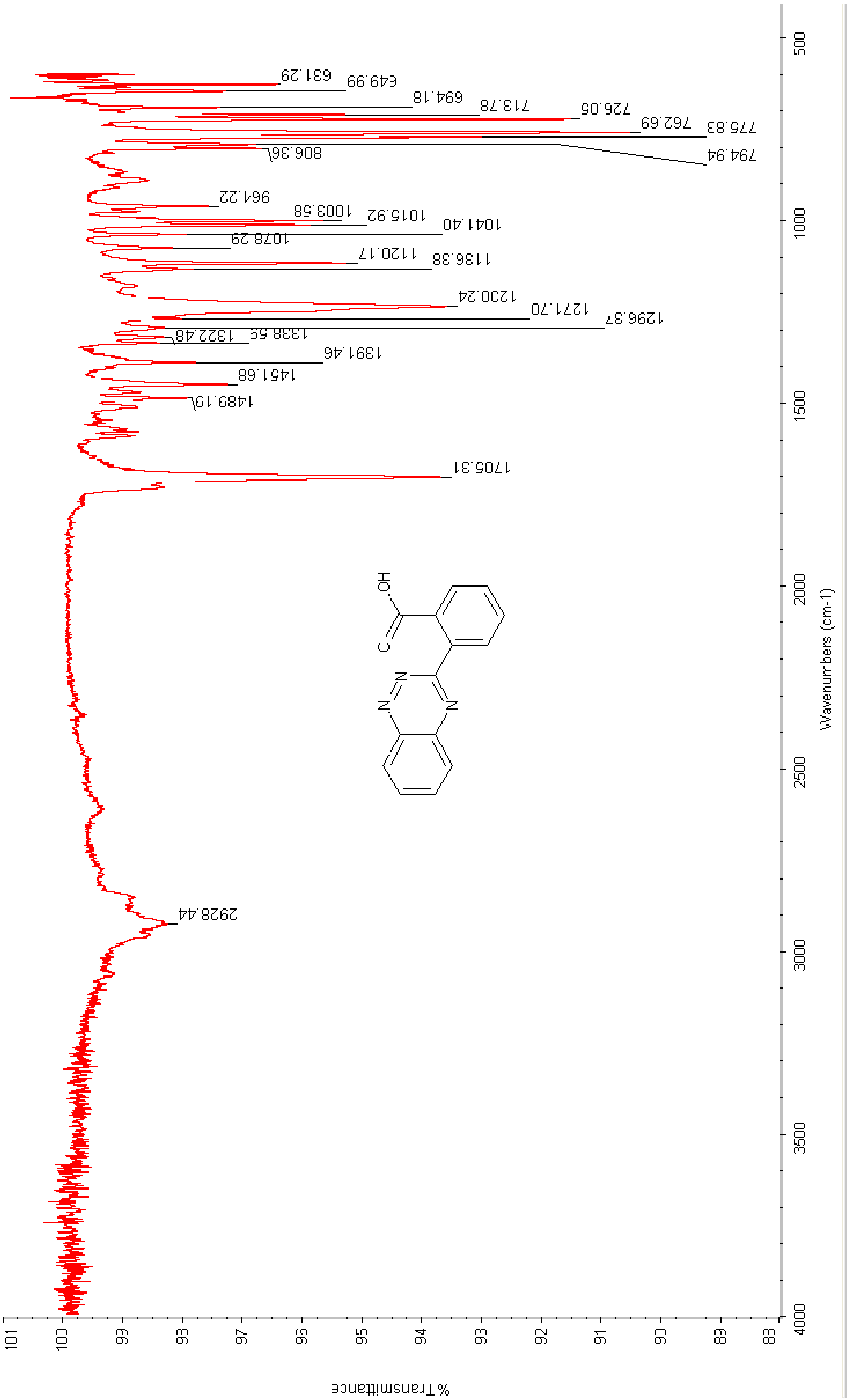
%Transmittance
Wavenumbers (cm-1)
2928.44
1705.31
1489.19
1451.68
1391.46
1338.59
1322.48
1296.37
1271.70
1238.24
1136.38
1120.17
1078.29
1041.40
1015.92
1003.58
964.22
806.36
794.94
775.83
762.69
726.05
713.78
694.18
649.99
631.29

IR of **17a**

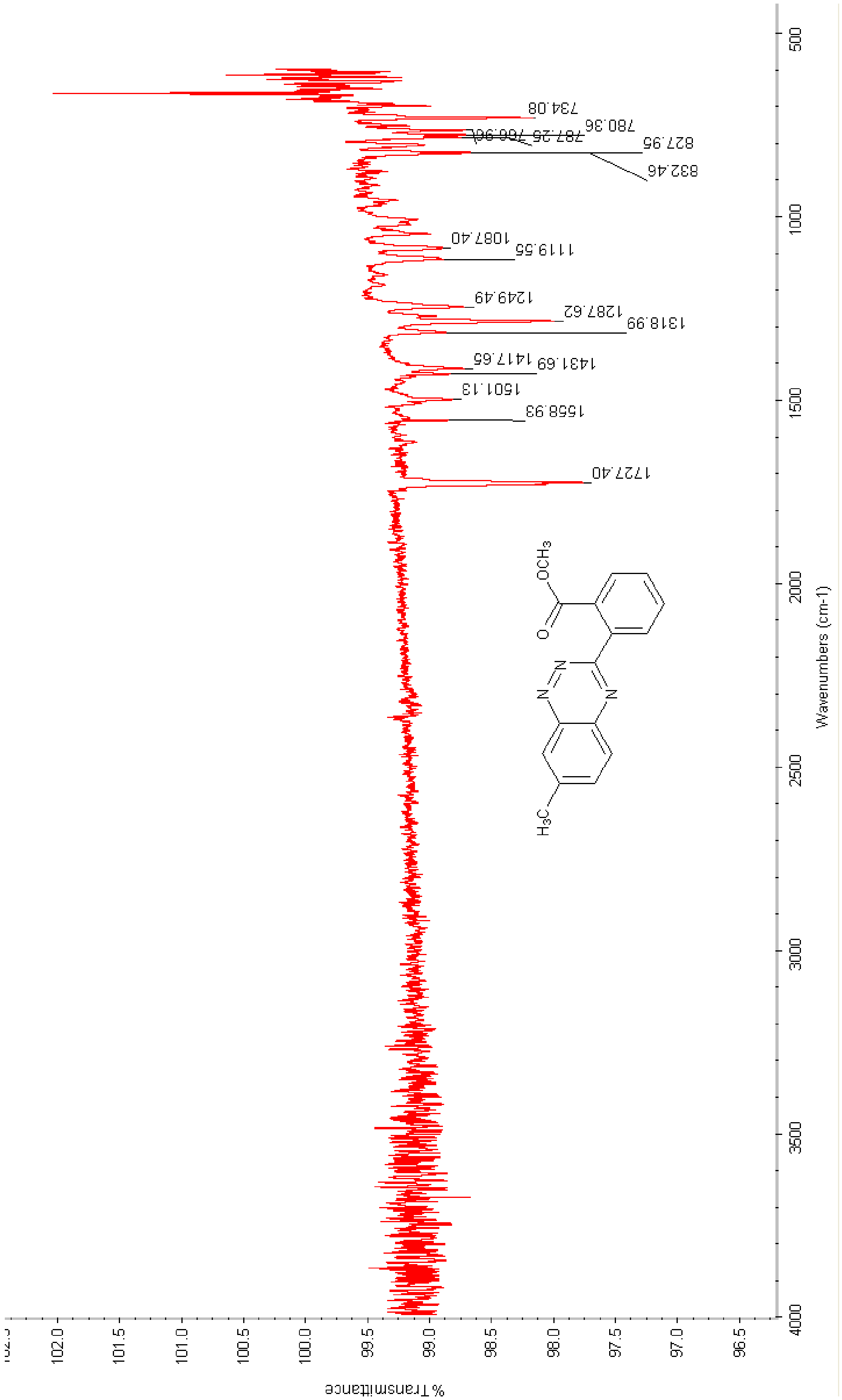

%Transmittance
Wavenumbers (cm-1)
1727.40
1558.93
1501.13
1431.69
1417.65
1318.99
1287.62
1249.49
1119.55
1087.40
832.46
827.95
787.25
780.36
734.08
OCH3

IR of **16b**

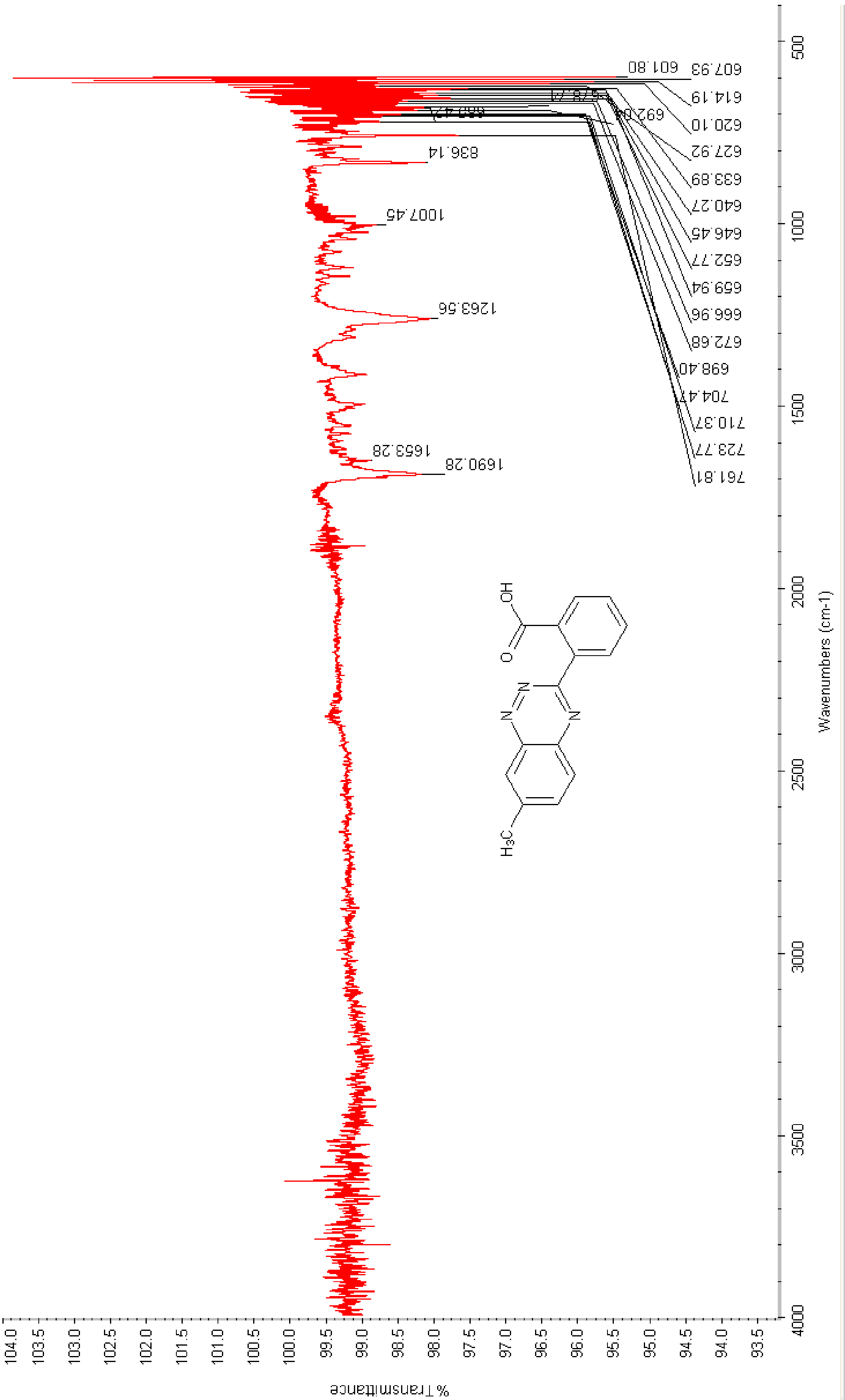
%Transmittance
Wavenumbers (cm-1)
607.93
601.80
614.19
620.10
627.92
633.89
640.27
646.45
652.77
659.94
666.96
672.68
698.40
710.37
723.77
761.81
836.14
1007.45
1263.56
1653.28
1690.28
H_3C
OH
O
N

IR of **17b**

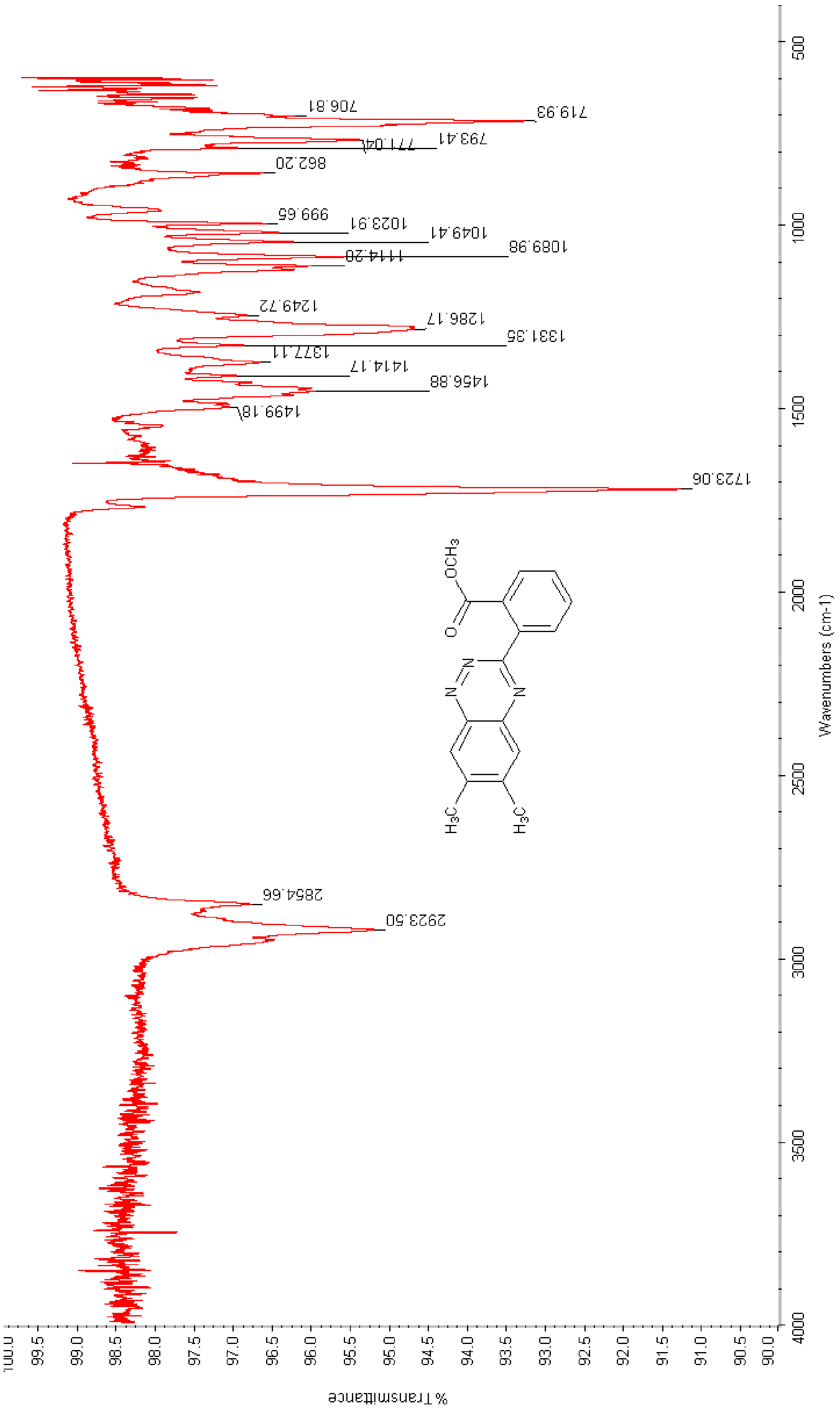
%Transmittance
Wavenumbers (cm-1)
2923.50
2854.66
1723.06
1499.18
1456.88
1414.17
1377.11
1331.35
1286.17
1249.72
1114.20
1089.98
1049.41
1023.91
999.65
862.20
793.41
771.04
719.93
706.81

IR of **16c**

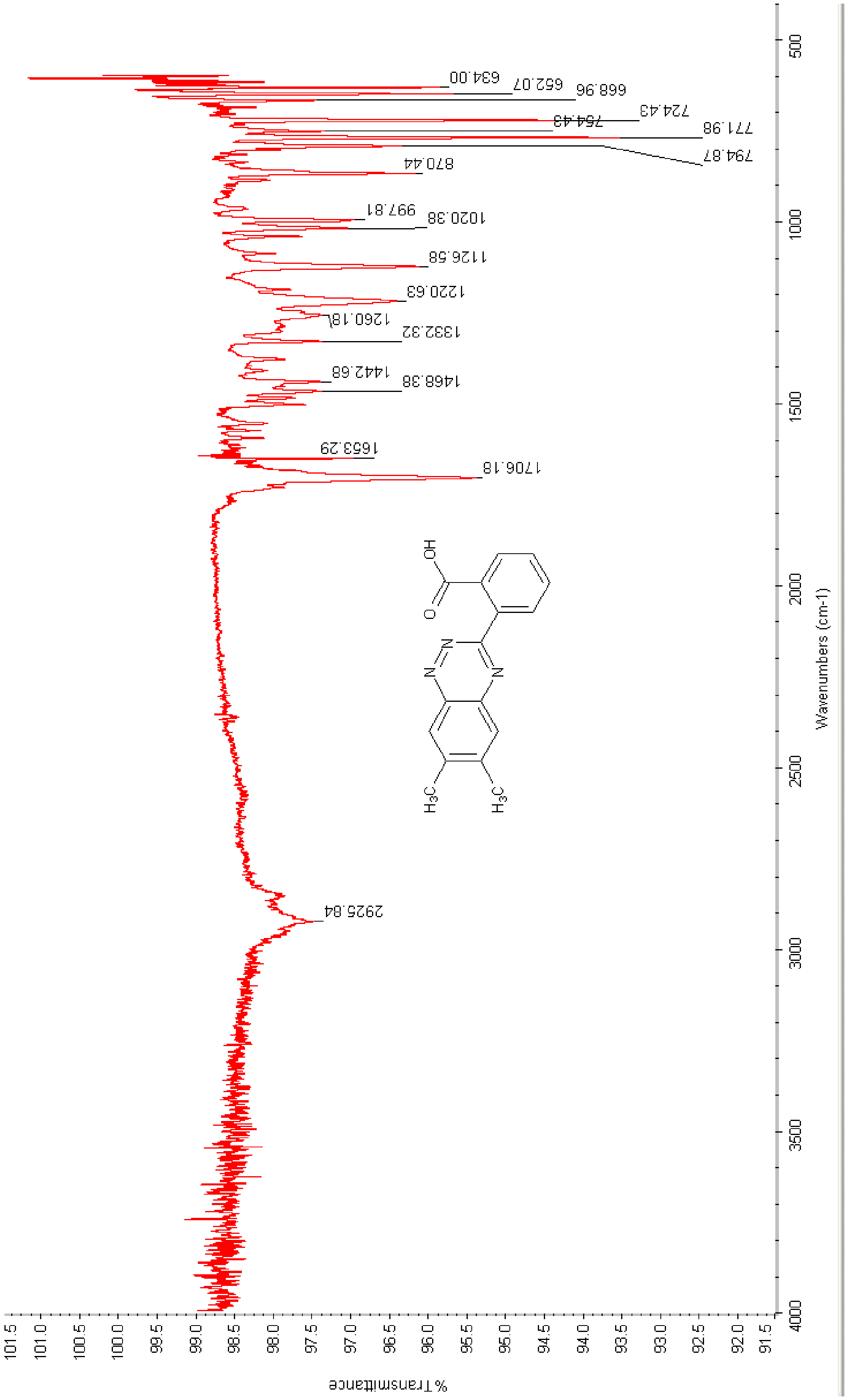
634.00
652.07
668.96
724.43
754.43
771.98
794.87
870.44
997.81
1020.38
1126.58
1220.63
1260.18
1332.32
1442.68
1468.38
1653.29
1706.18
2925.84
%Transmittance
Wavenumbers (cm-1)

IR of **17c**

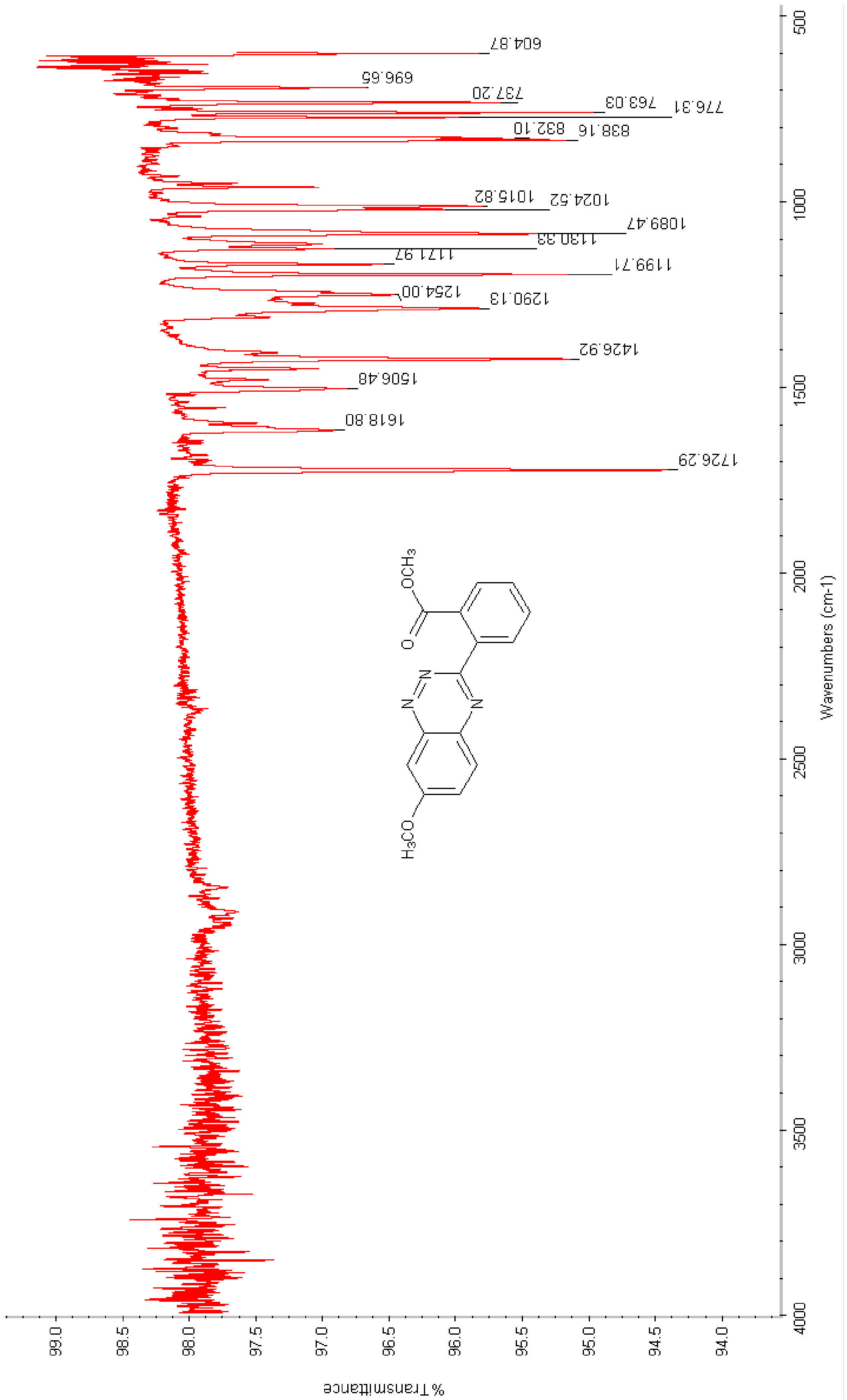
604.87
696.65
737.20
763.03
776.31
832.10
838.16
1015.82
1024.52
1089.47
1130.33
1171.97
1199.71
1254.00
1290.13
1426.92
1506.48
1618.80
1726.29
Wavenumbers (cm-1)
%Transmittance

IR of **16d**

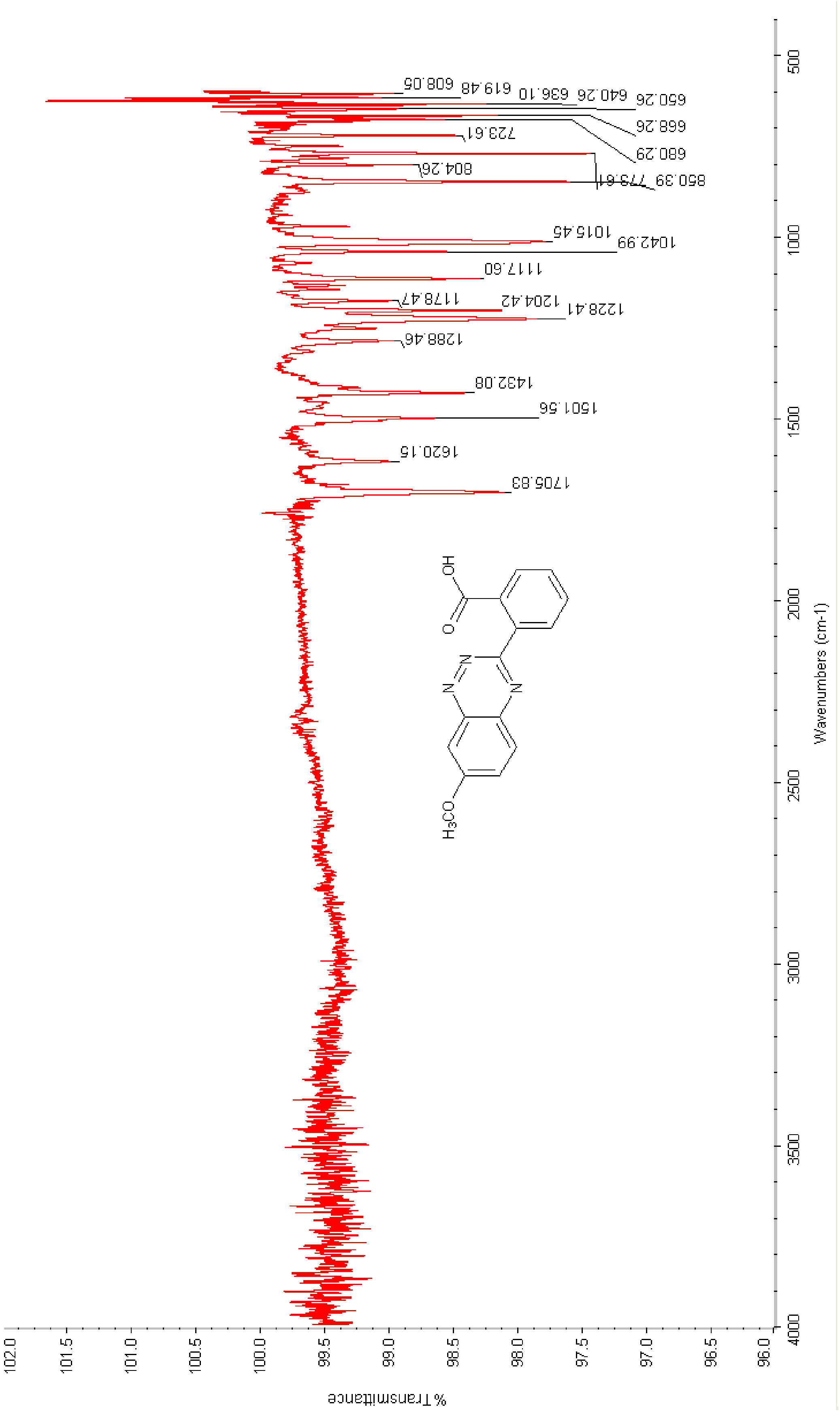
%Transmittance
Wavenumbers (cm-1)
1705.83
1620.15
1501.56
1432.08
1288.46
1228.41
1204.42
1178.47
1117.60
1042.99
1015.45
850.39
804.26
773.61
723.61
680.29
668.26
650.26
640.26
636.10
619.48
608.05

IR of **17d**

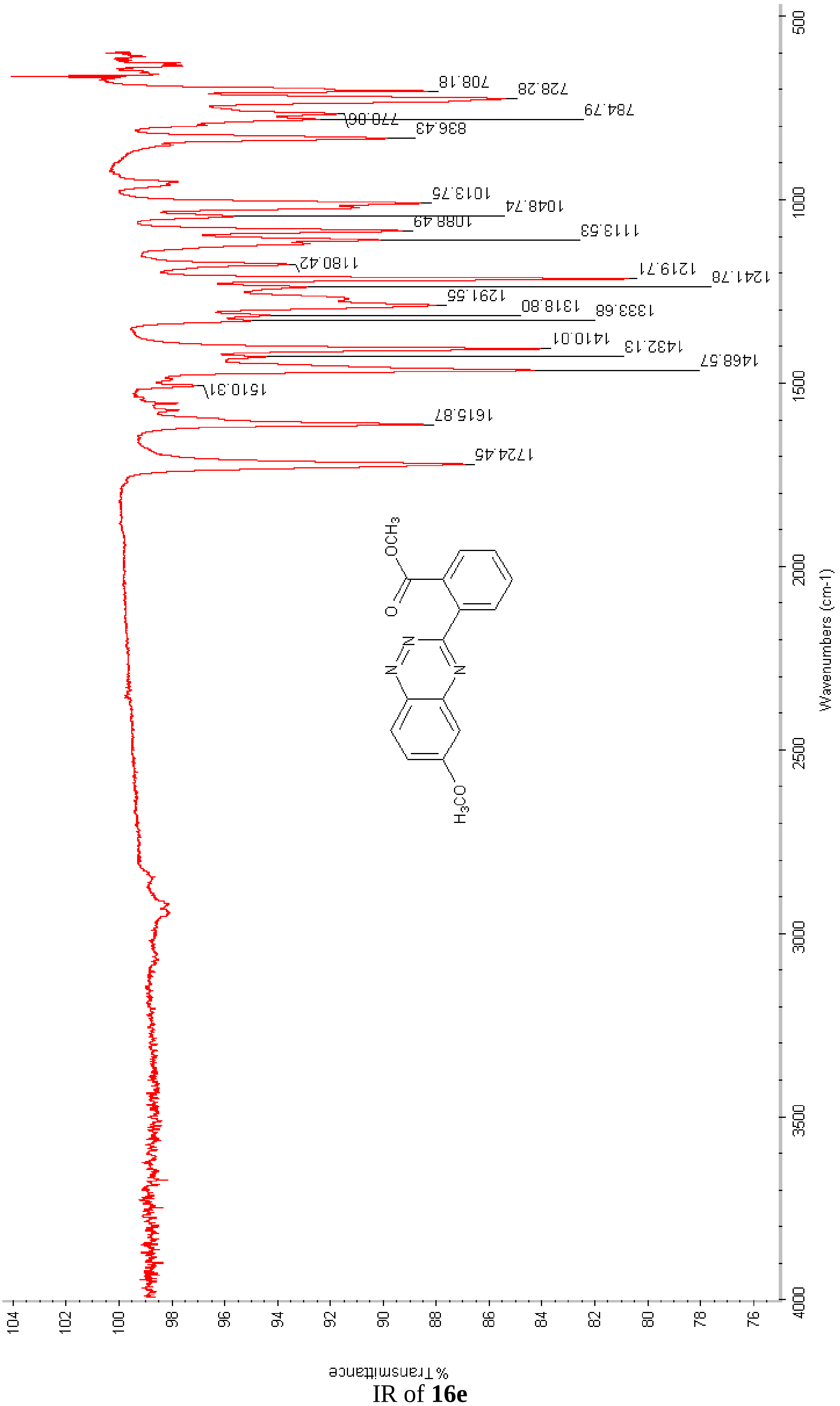

708.18
728.28
784.79
770.06
836.43
1013.75
1048.74
1088.49
1113.53
1180.42
1219.71
1241.78
1291.55
1318.80
1333.68
1410.01
1432.13
1468.57
1510.31
1615.87
1724.45
Wavenumbers (cm-1)
%Transmittance

IR of **16e**

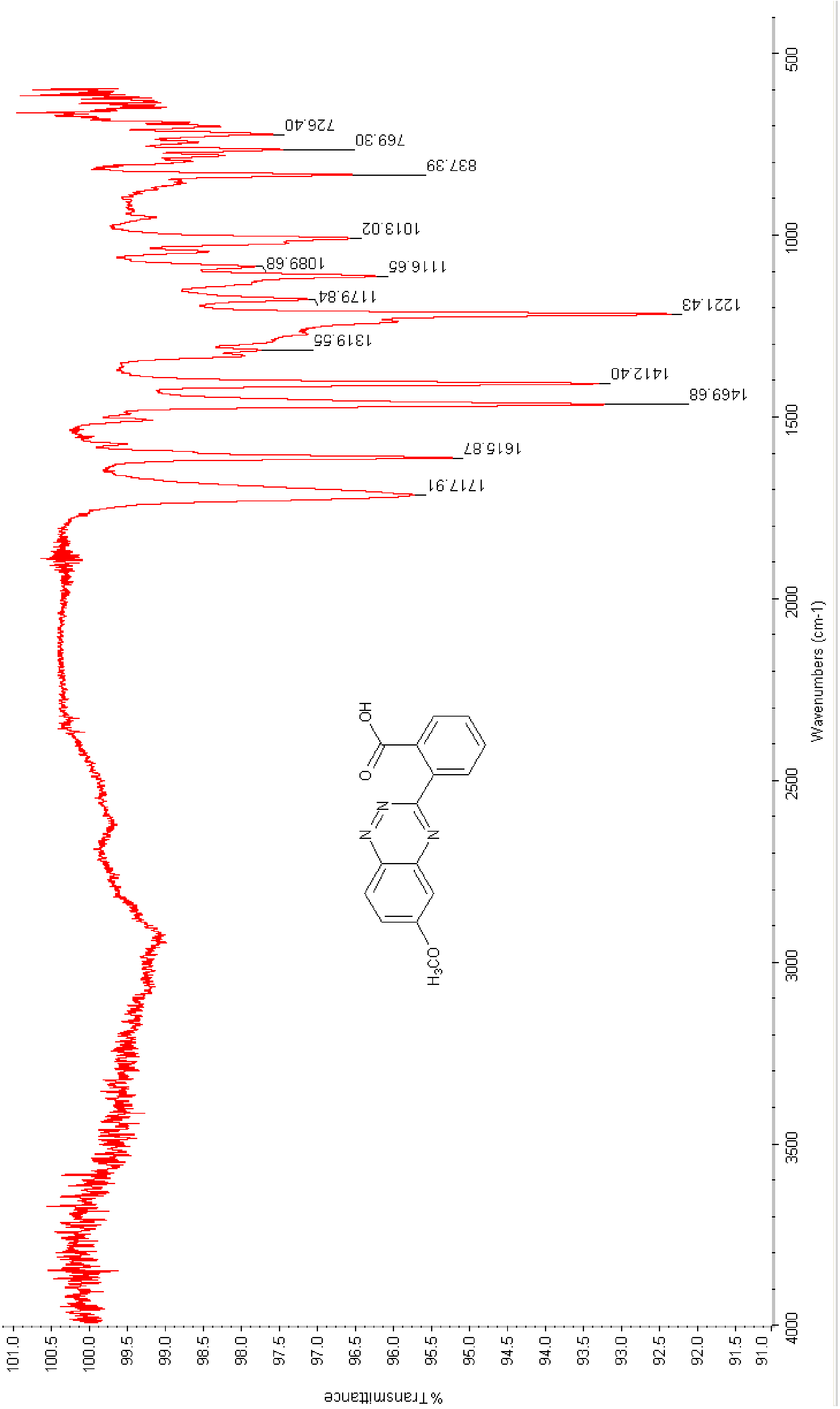

1717.91
1615.87
1469.68
1412.40
1319.55
1221.43
1179.84
1116.65
1089.68
1013.02
837.39
769.30
726.40
Wavenumbers (cm-1)
%Transmittance
H3CO
N
N
O
OH

IR of **17e**

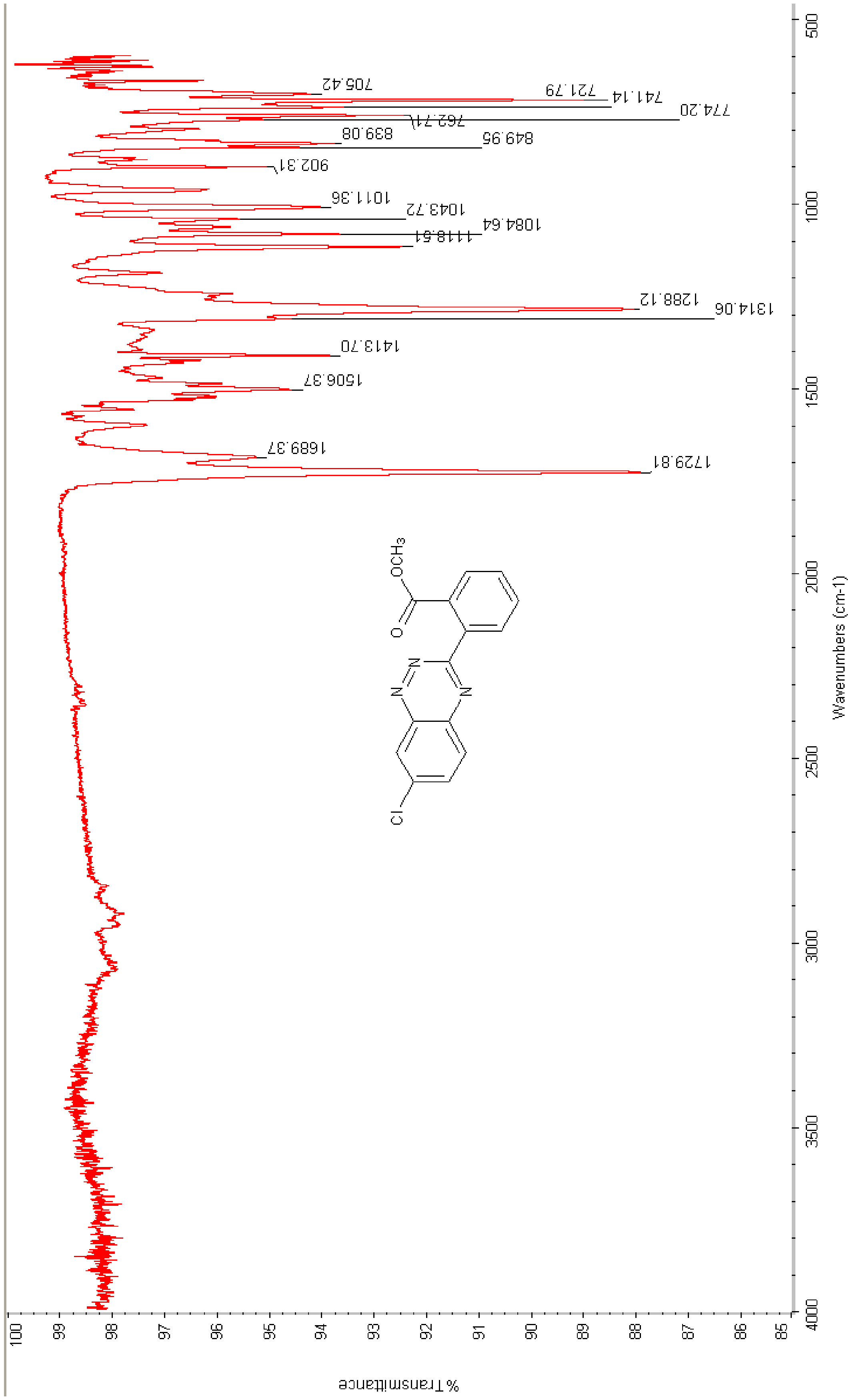

705.42
721.79
741.14
774.20
762.71
839.08
849.95
902.31
1011.36
1043.72
1084.64
1118.51
1288.12
1314.06
1413.70
1506.37
1689.37
1729.81
OCH3
O
N
N
N
N
Cl
Wavenumbers (cm-1)
%Transmittance

IR of **16f**

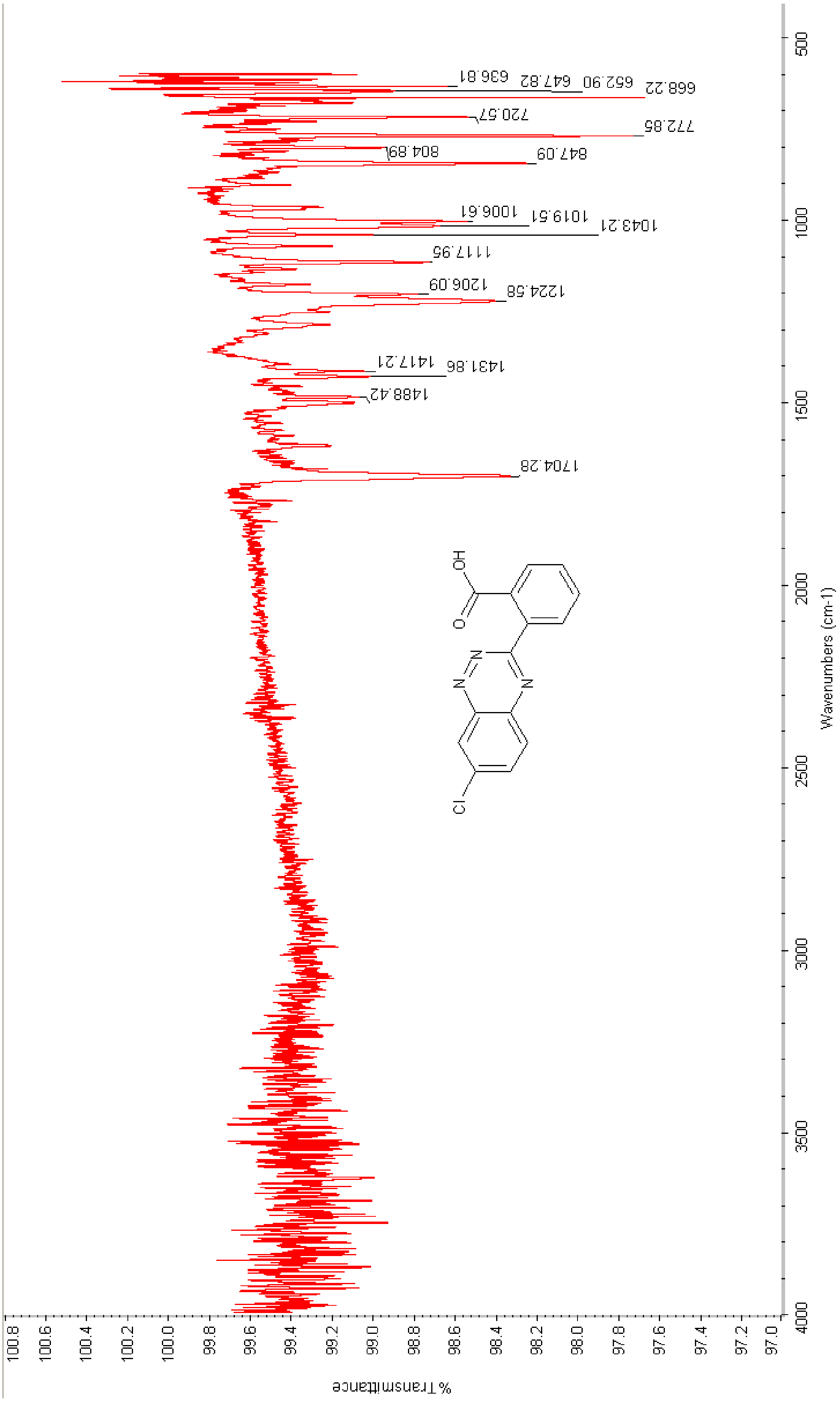
1704.28
1488.42
1431.86
1417.21
1224.58
1206.09
1117.95
1043.21
1019.51
1006.61
847.09
804.89
772.85
720.57
668.22
652.90
647.82
636.81
%Transmittance
Wavenumbers (cm-1)

IR of **17f**

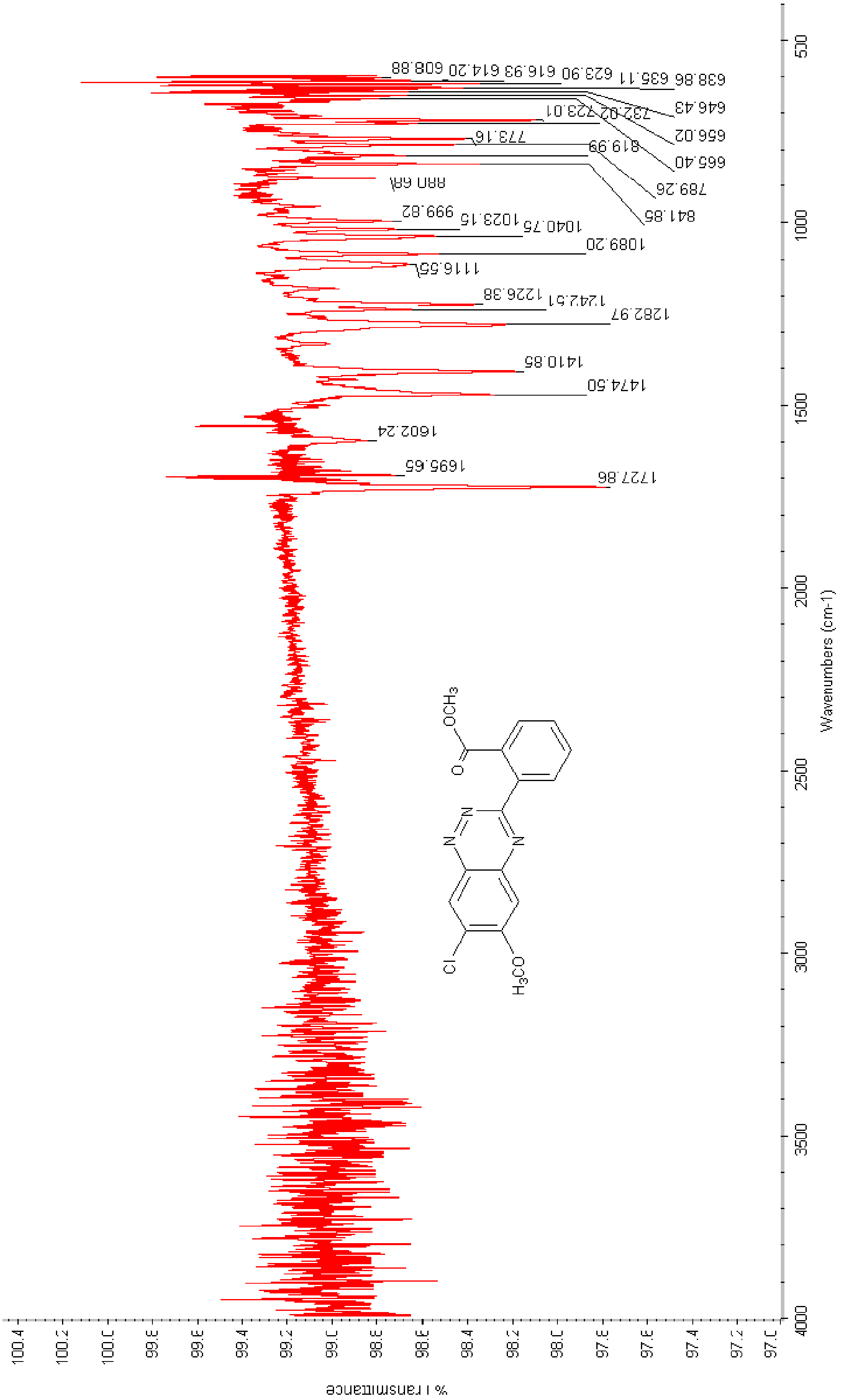
% Transmittance
Wavenumbers (cm-1)
1727.86
1695.65
1602.24
1474.50
1410.85
1282.97
1242.51
1226.38
1116.55
1089.20
1040.75
1023.15
999.82
880.68
841.85
819.99
789.26
773.16
732.02
723.01
665.40
656.02
646.43
638.86
635.11
623.90
616.93
614.20
608.88
Cl
H3CO
N
OCH3
O

IR of **16g**

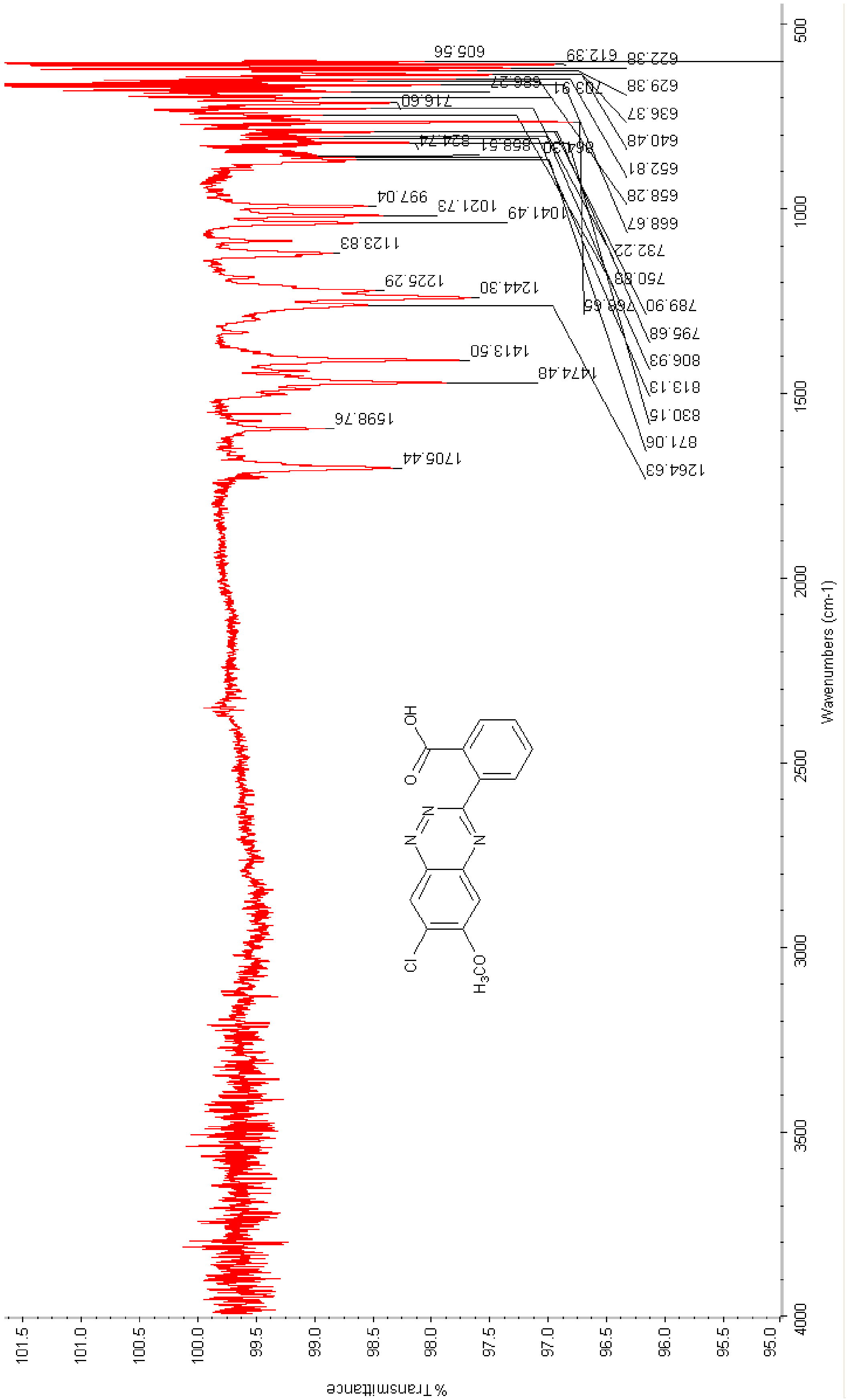

%Transmittance
Wavenumbers (cm-1)
1705.44
1598.76
1474.48
1413.50
1244.30
1225.29
1123.83
1041.49
1021.73
997.04
868.51
824.74
716.60
686.27
703.91
605.56
612.39
622.38
629.38
636.37
640.48
652.81
658.28
668.67
732.22
750.88
789.90
795.68
806.93
813.13
830.15
871.06
1264.63

IR of **17g**

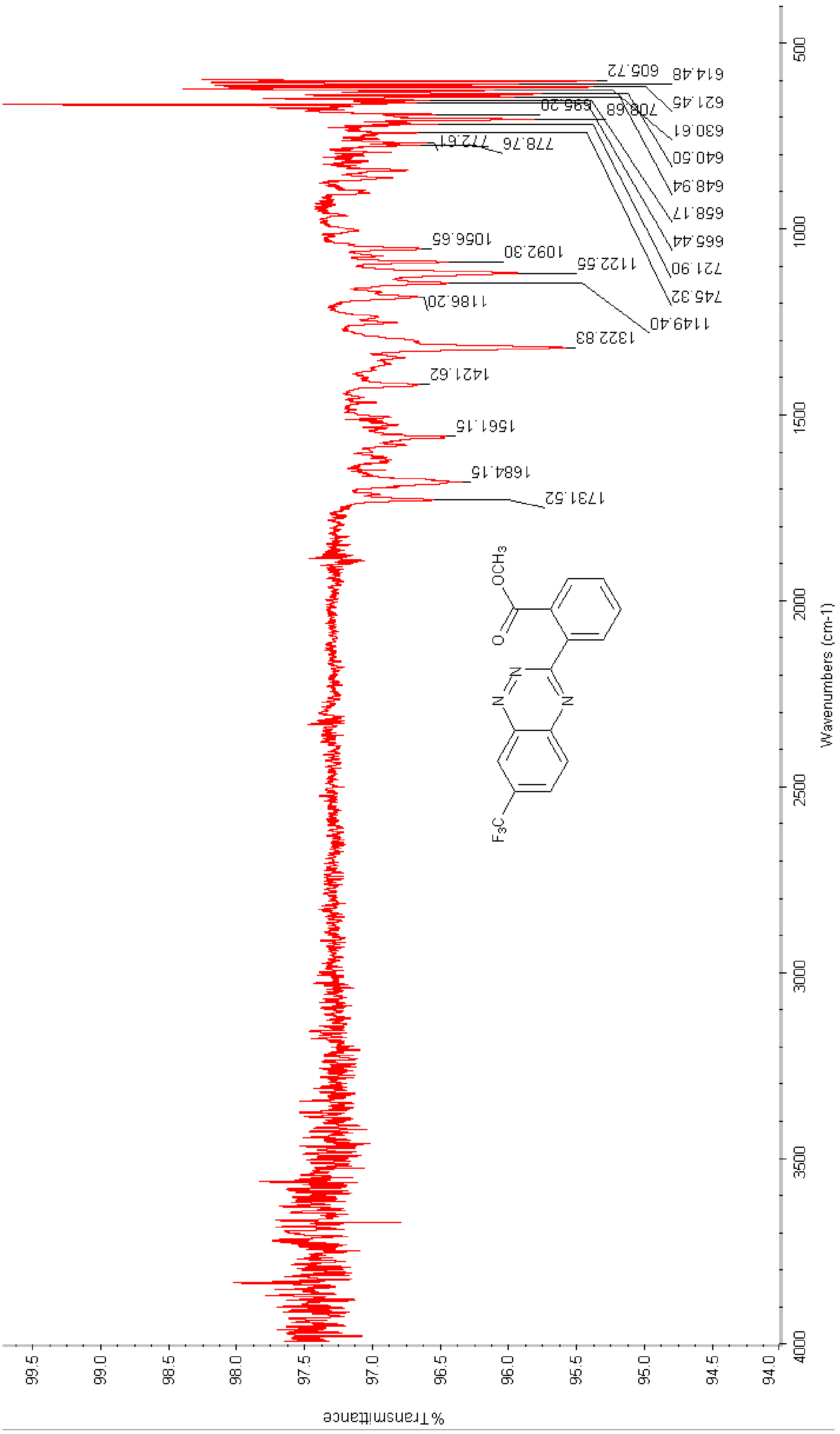
605.72
614.48
621.45
630.61
640.50
648.94
658.17
665.44
695.20
708.68
721.90
745.32
772.61
778.76
1056.65
1092.30
1122.55
1149.40
1186.20
1322.83
1421.62
1561.15
1684.15
1731.52
Wavenumbers (cm-1)
%Transmittance

IR of **16h**

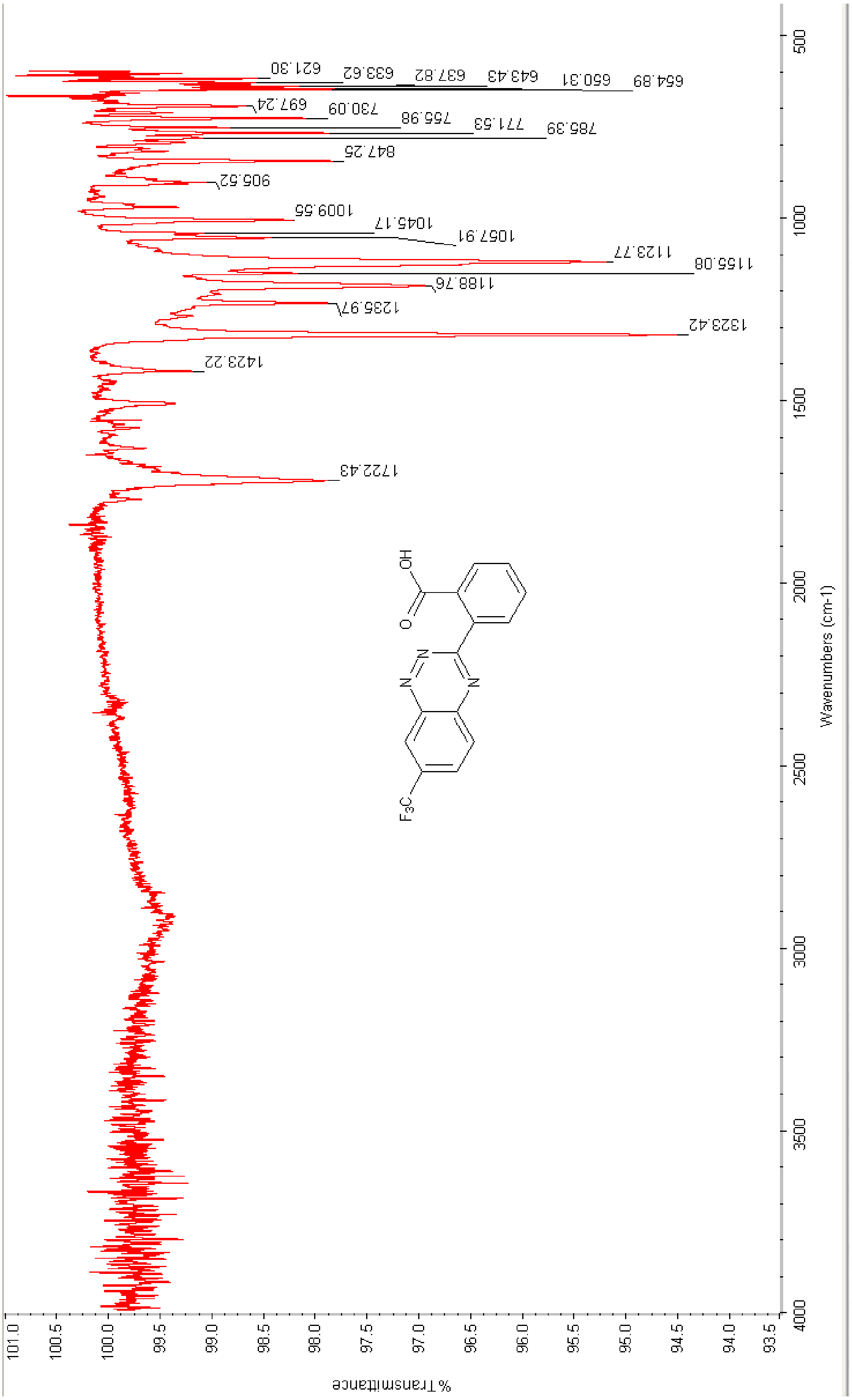
1722.43
1423.22
1323.42
1235.97
1188.76
1155.08
1123.77
1057.91
1045.17
1009.55
905.52
847.25
785.39
771.53
755.98
730.09
697.24
654.89
650.31
643.43
637.82
633.62
621.30
%Transmittance
Wavenumbers (cm-1)

IR of **17h**

APPENDIX C: HR-MS SPECTROSCOPY:

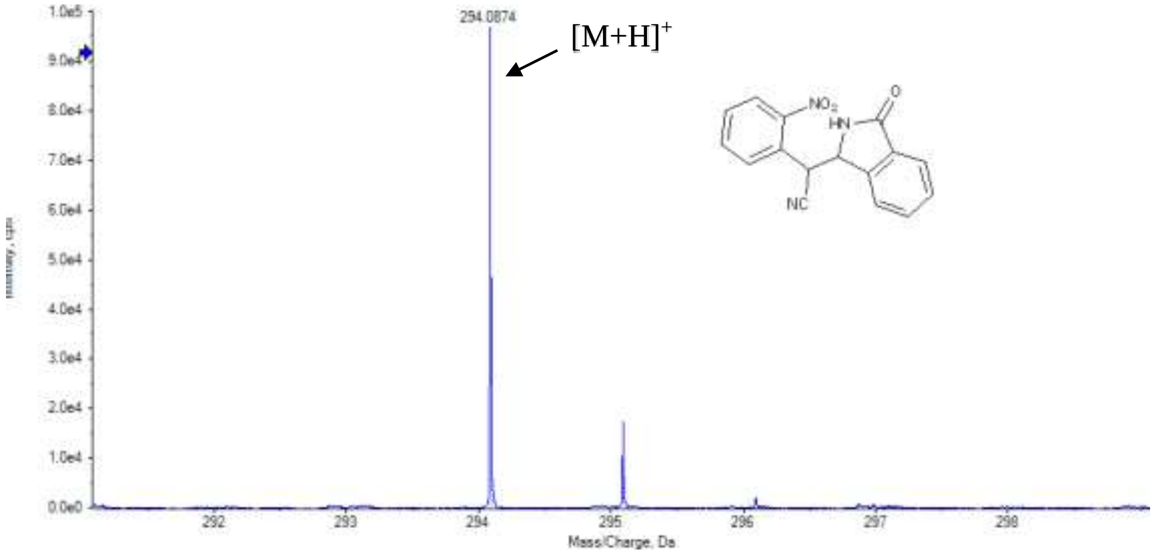

HRMS of **6** (ESI-Orbitrap) in methanol

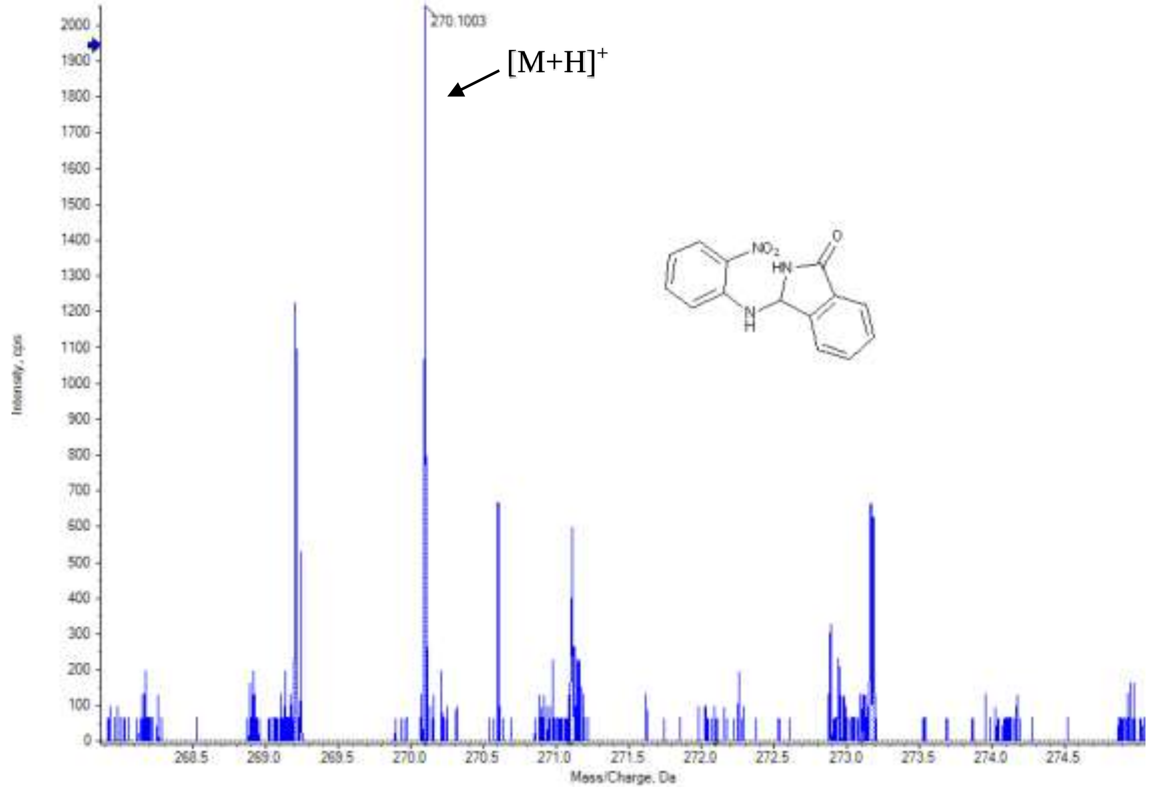

HRMS of **10a** (ESI-Orbitrap) in methanol

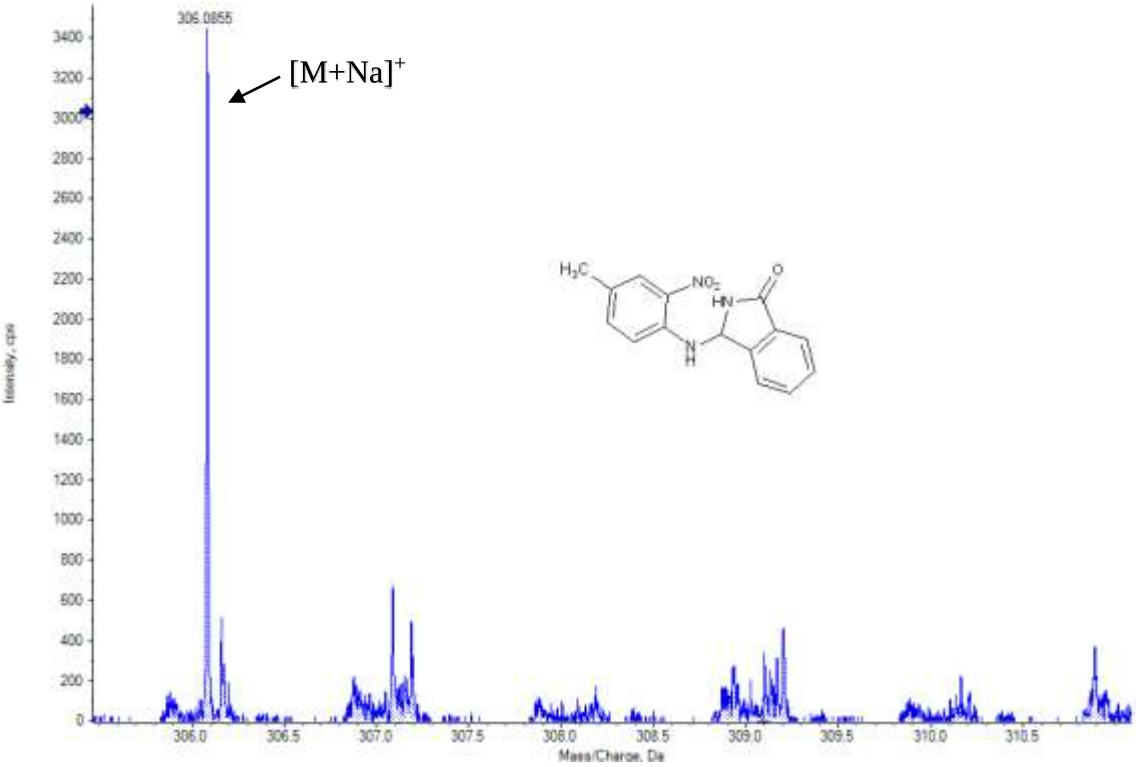

HRMS of **10b** (ESI-Orbitrap) in methanol

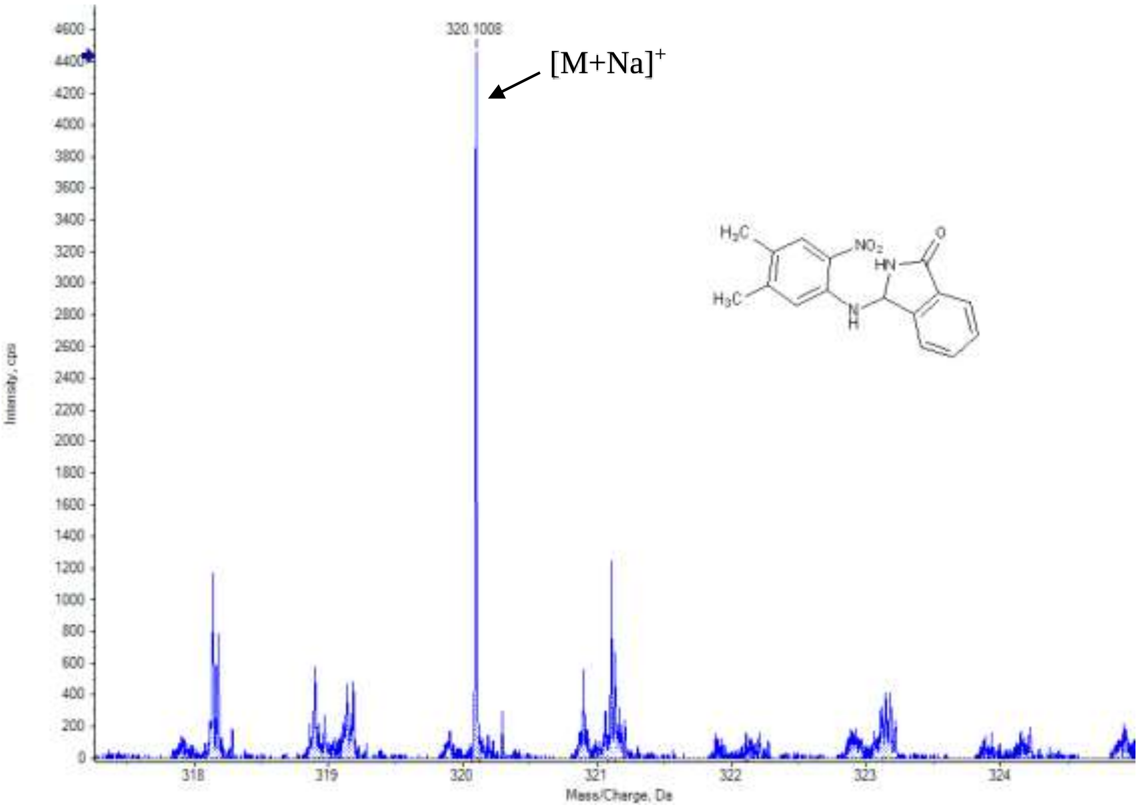

HRMS of **10c** (ESI-Orbitrap) in methanol

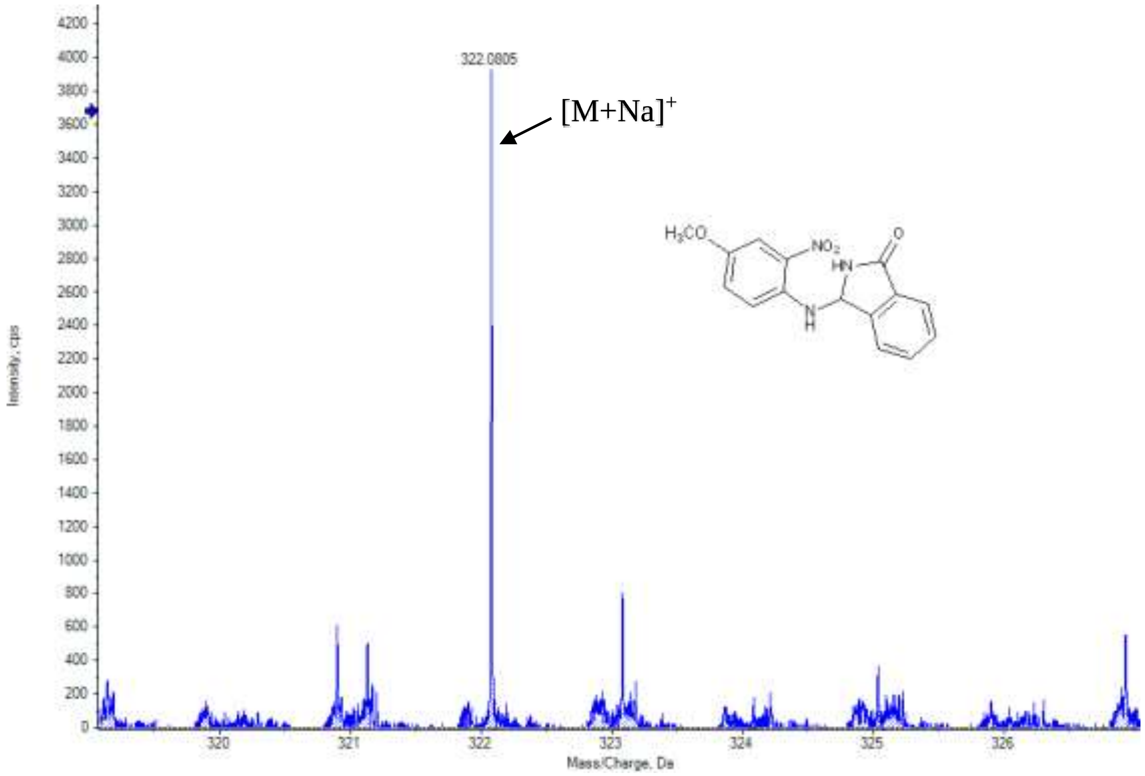

HRMS of **10d** (ESI-Orbitrap) in methanol

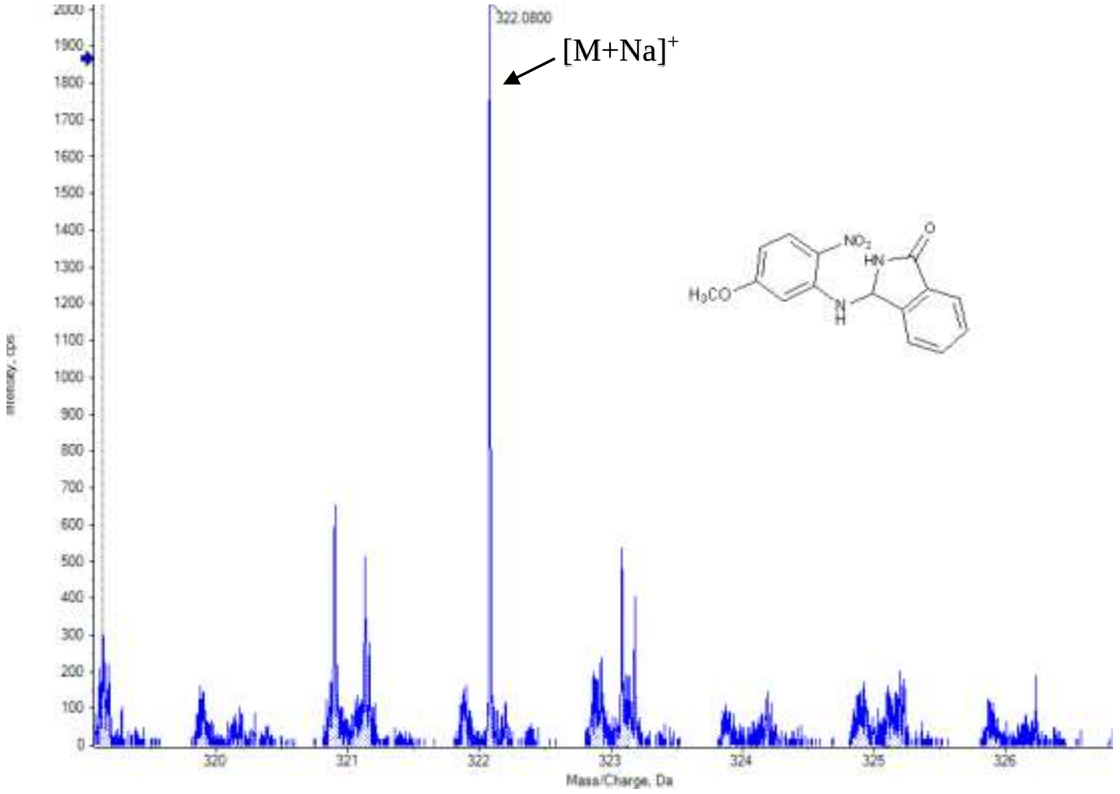

HRMS of **10e** (ESI-Orbitrap) in methanol

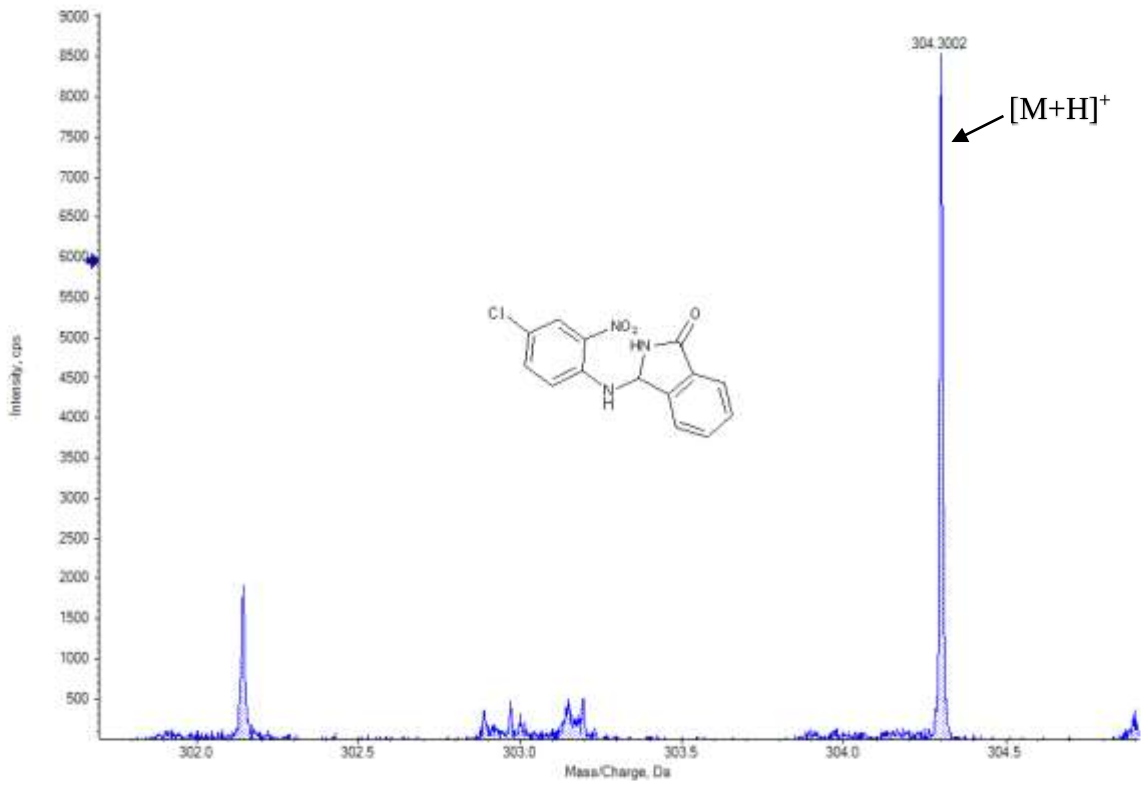

HRMS of **10f** (ESI-Orbitrap) in methanol

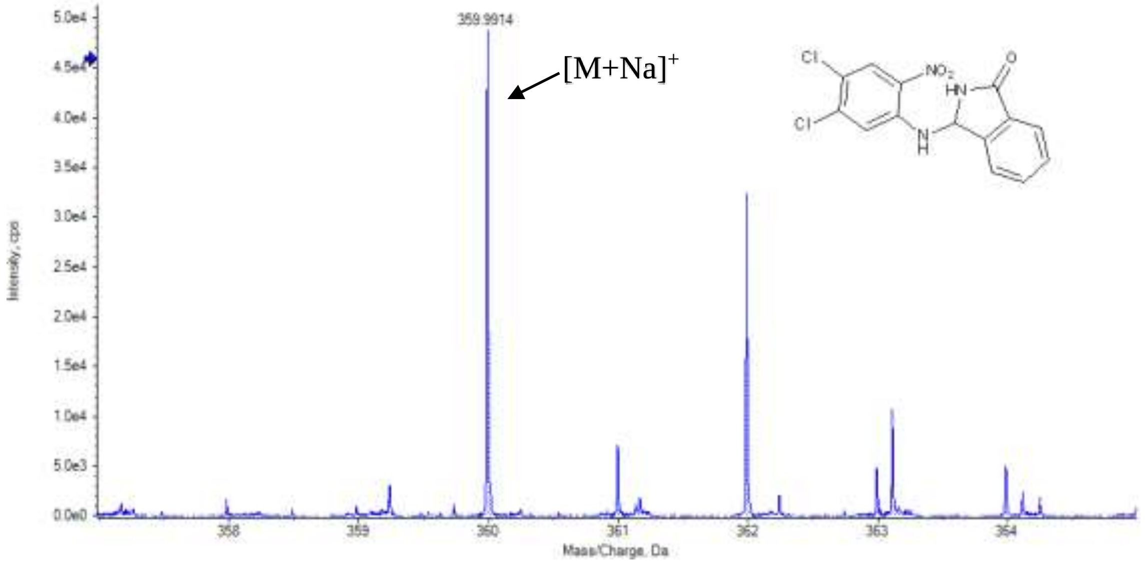

HRMS of **10g** (ESI-Orbitrap) in methanol

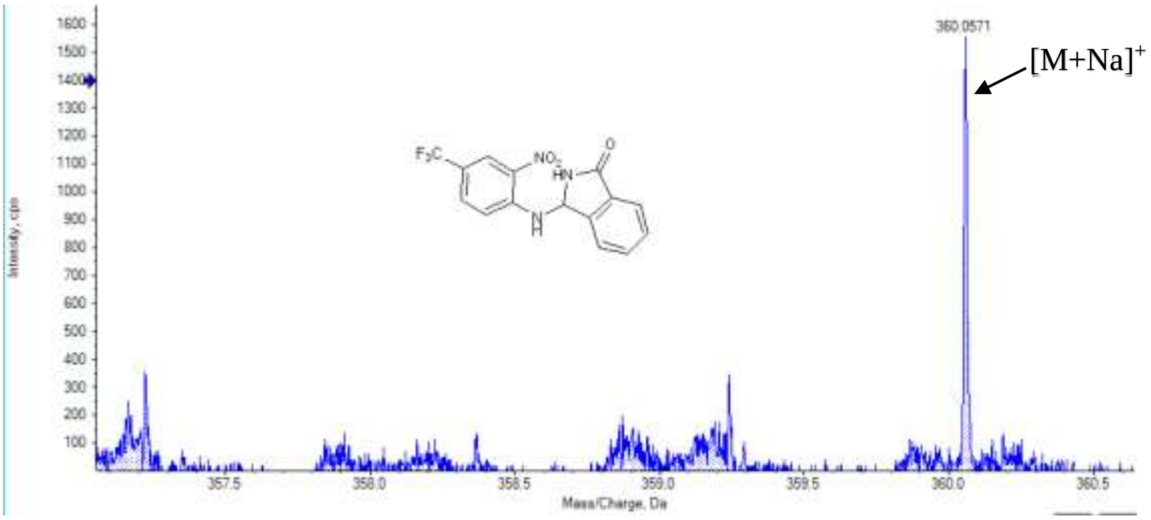

HRMS of **10h** (ESI-Orbitrap) in methanol

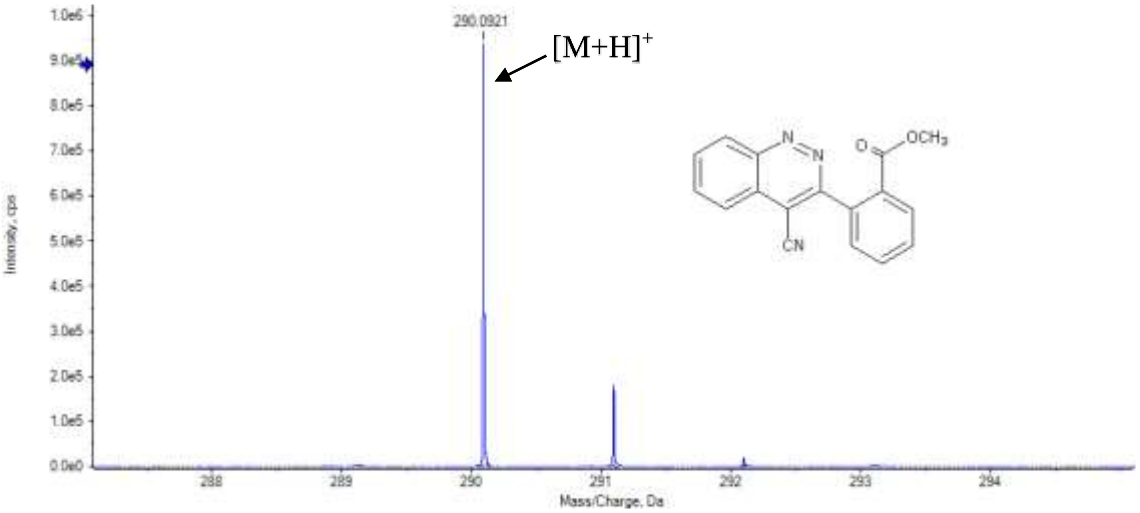

HRMS of **14** (ESI-Orbitrap) in methanol

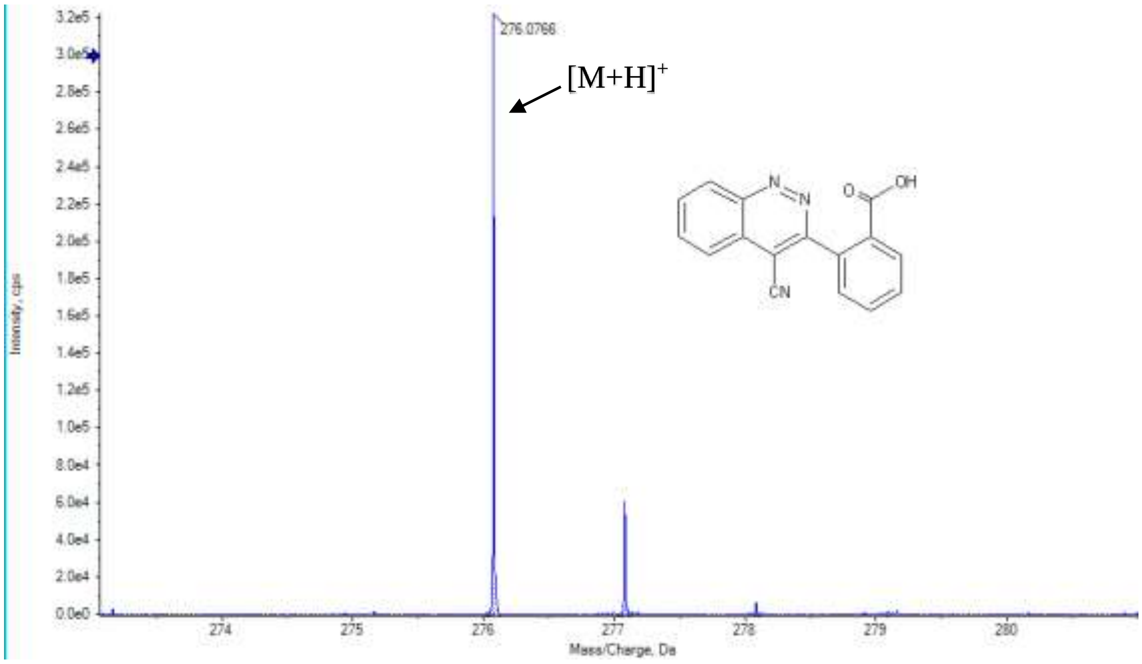

HRMS of **15** (ESI-Orbitrap) in methanol

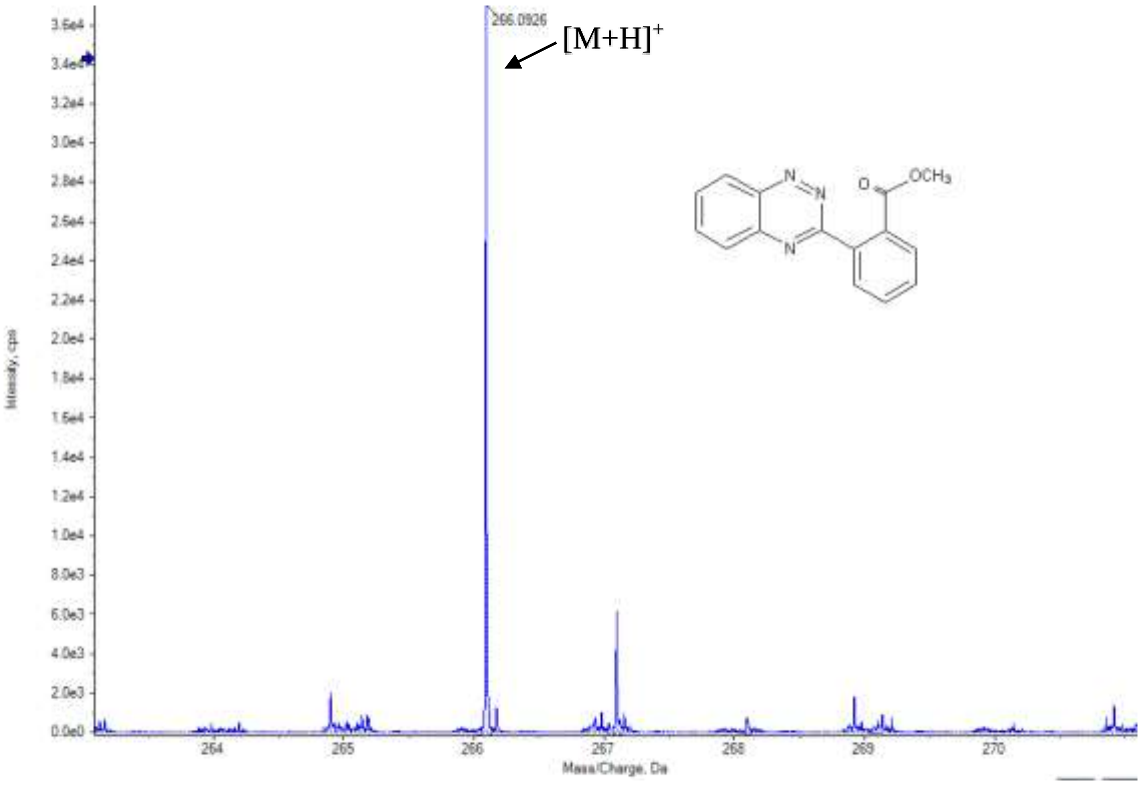

HRMS of **16a** (ESI-Orbitrap) in mehanol

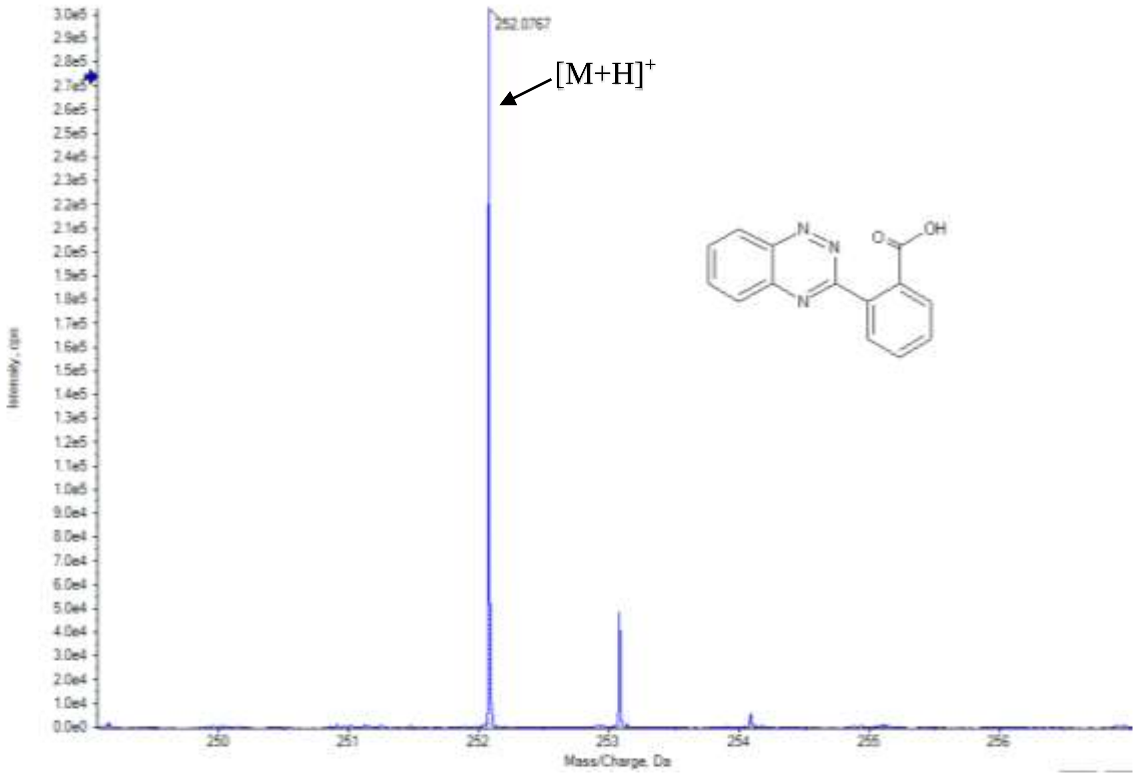

HRMS of **17a** (ESI-Orbitrap) in methanol

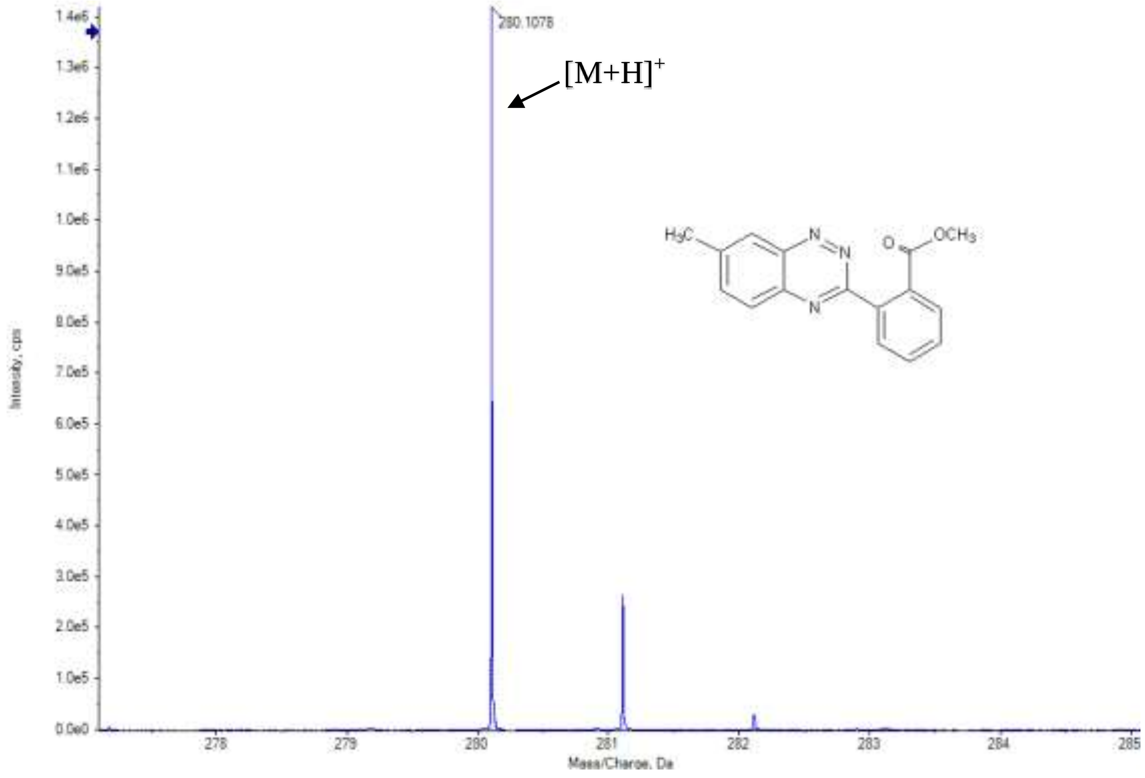

HRMS of **16b** (ESI-Orbitrap) in methanol

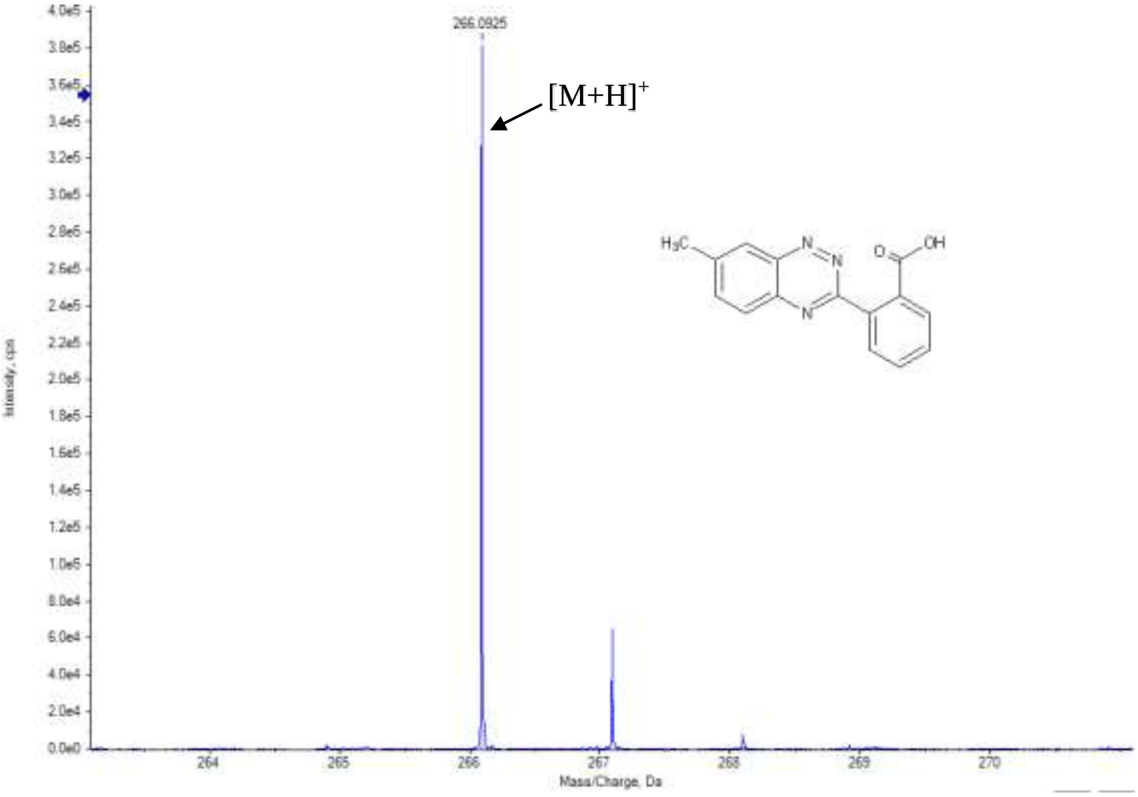

HRMS of **17b** (ESI-Orbitrap) in methanol

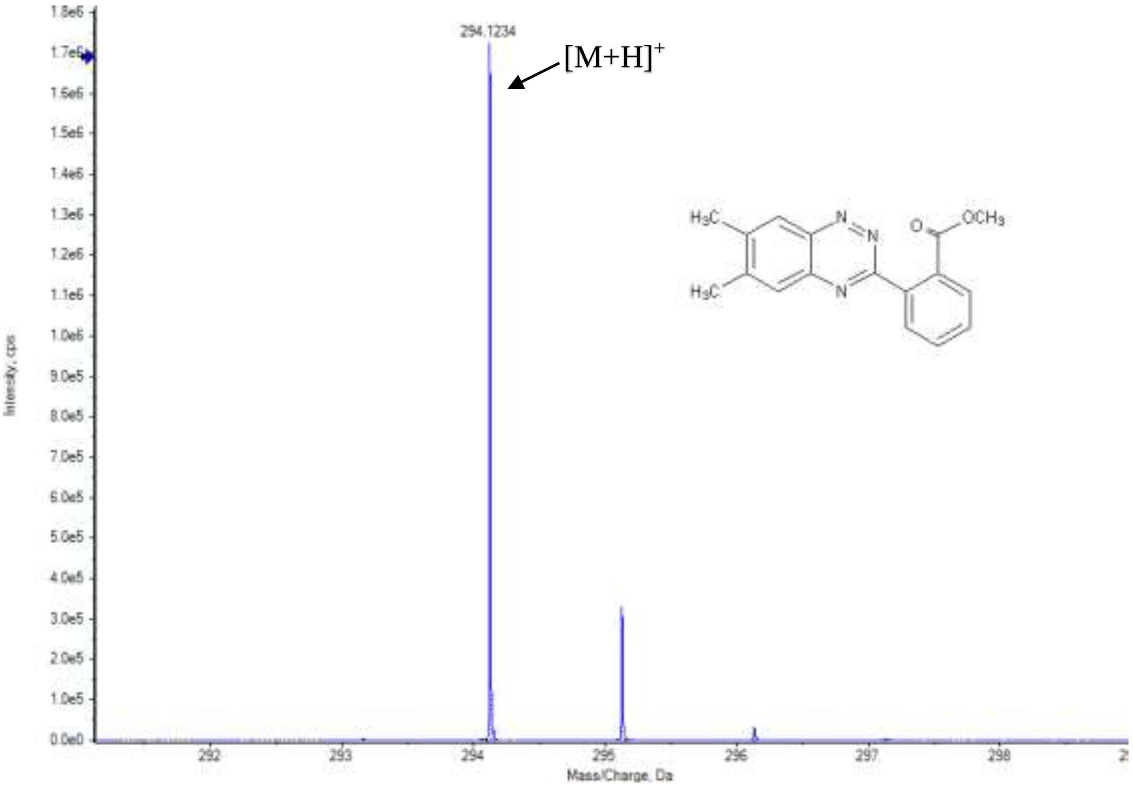

HRMS of **16c** (ESI-Orbitrap) in methanol

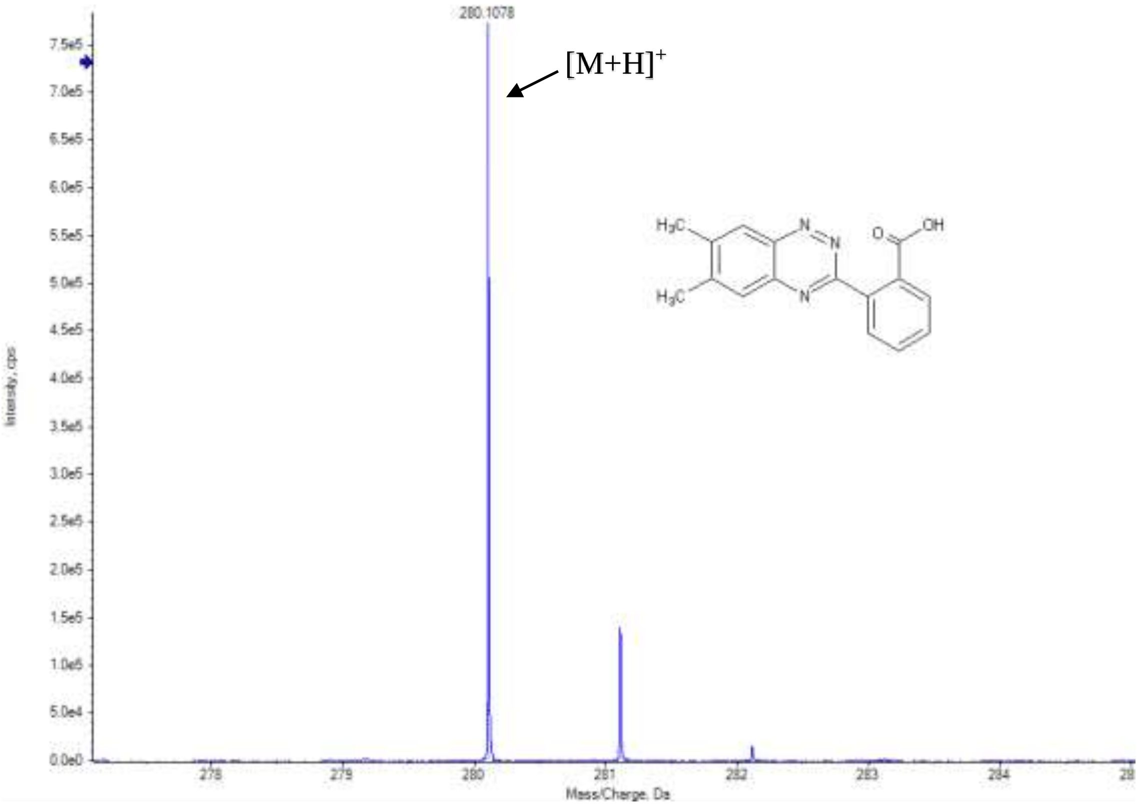

HRMS of **17c** (ESI-Orbitrap) in methanol

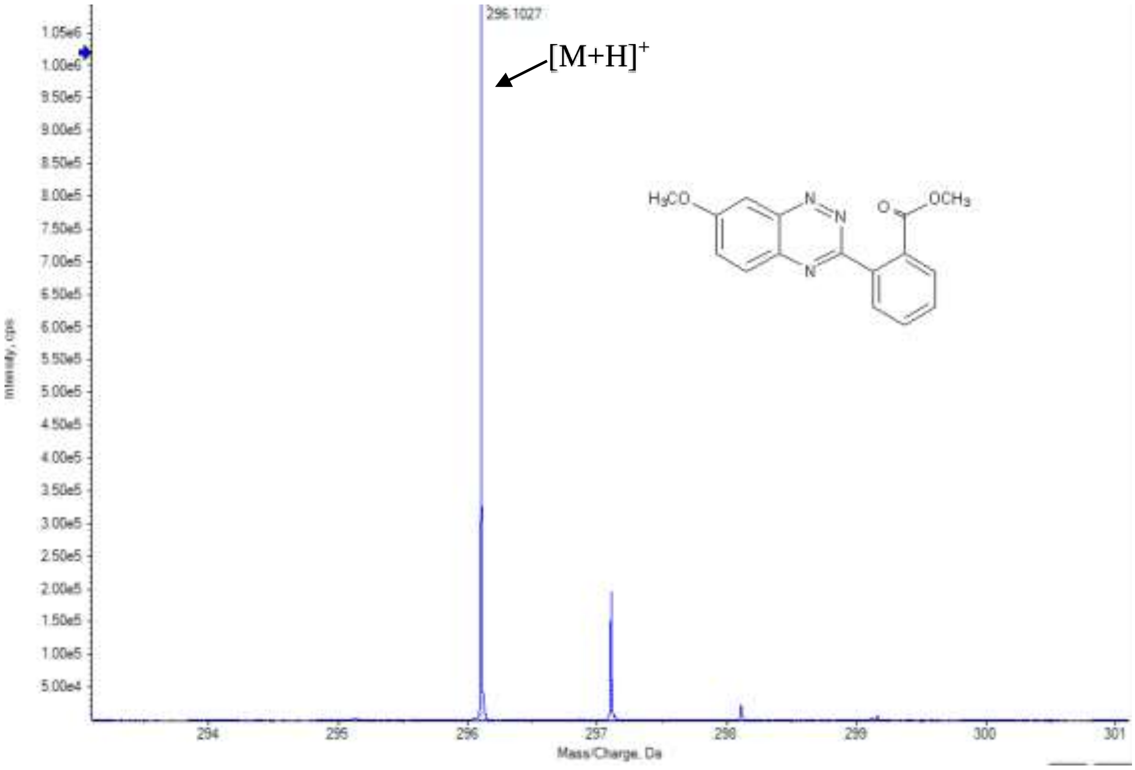

HRMS of **16d** (ESI-Orbitrap) in methanol

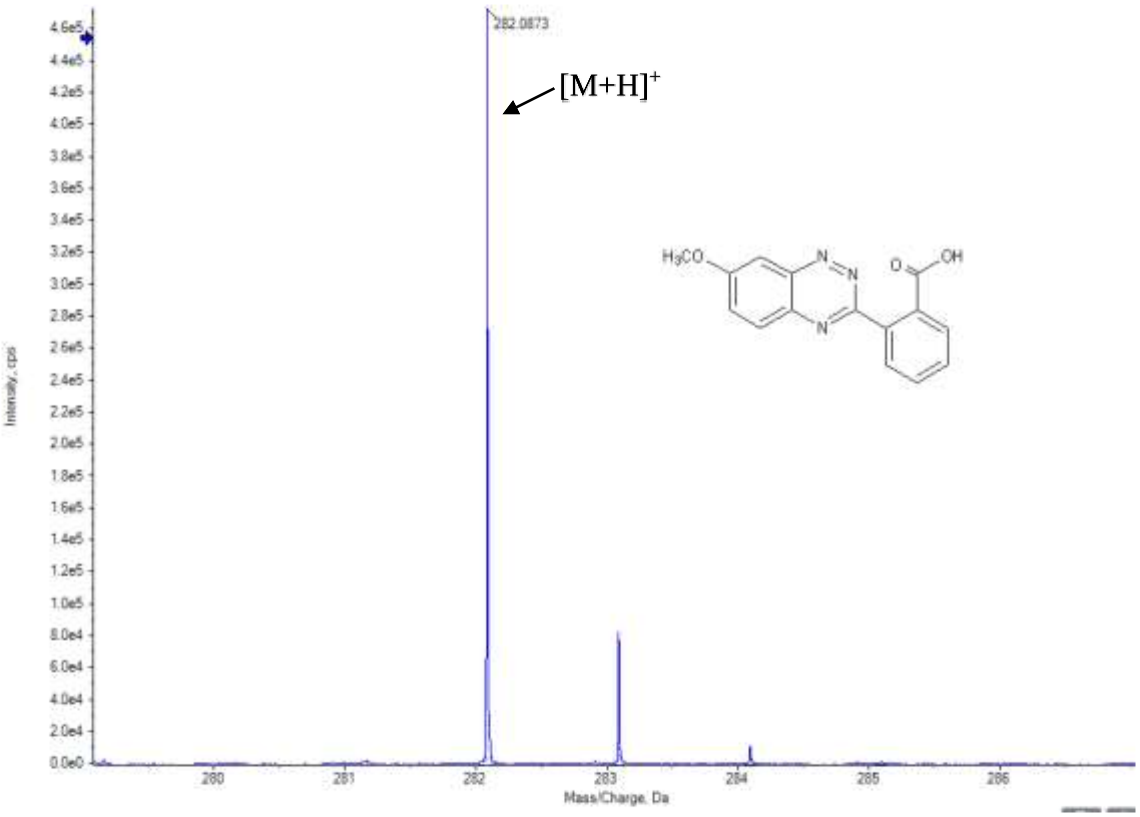

HRMS of **17d** (ESI-Orbitrap) in methanol

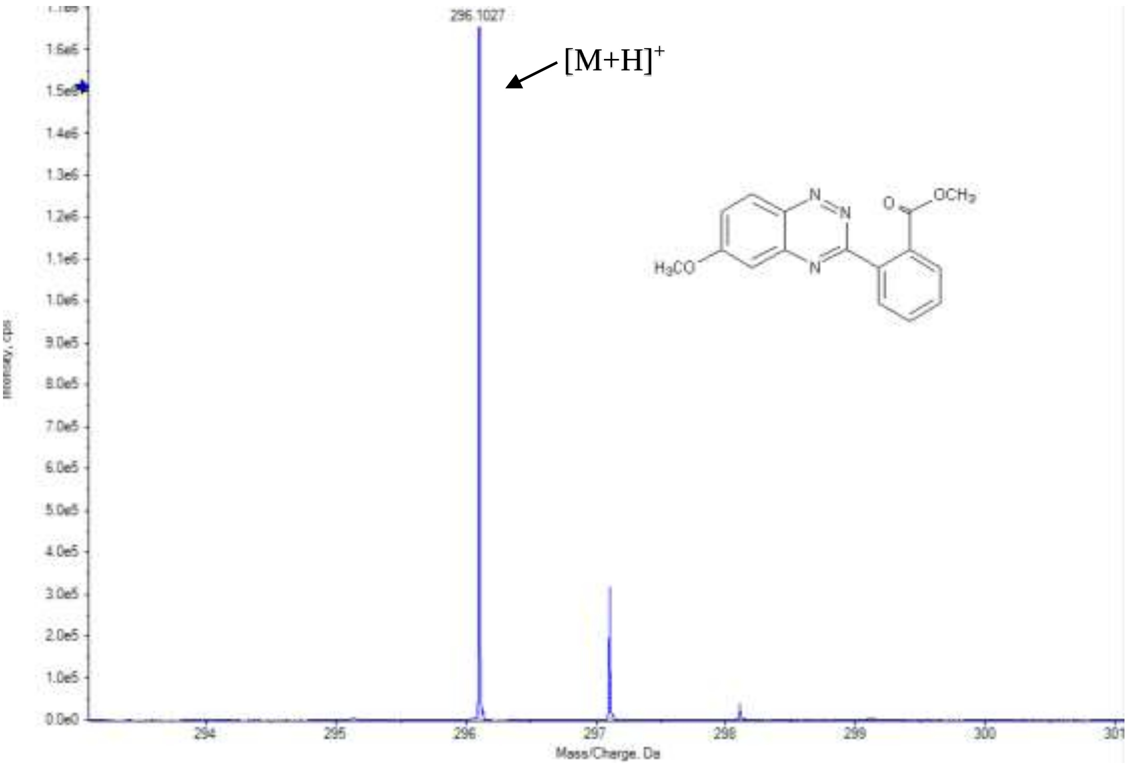

HRMS of **16e** (ESI-Orbitrap) in methanol

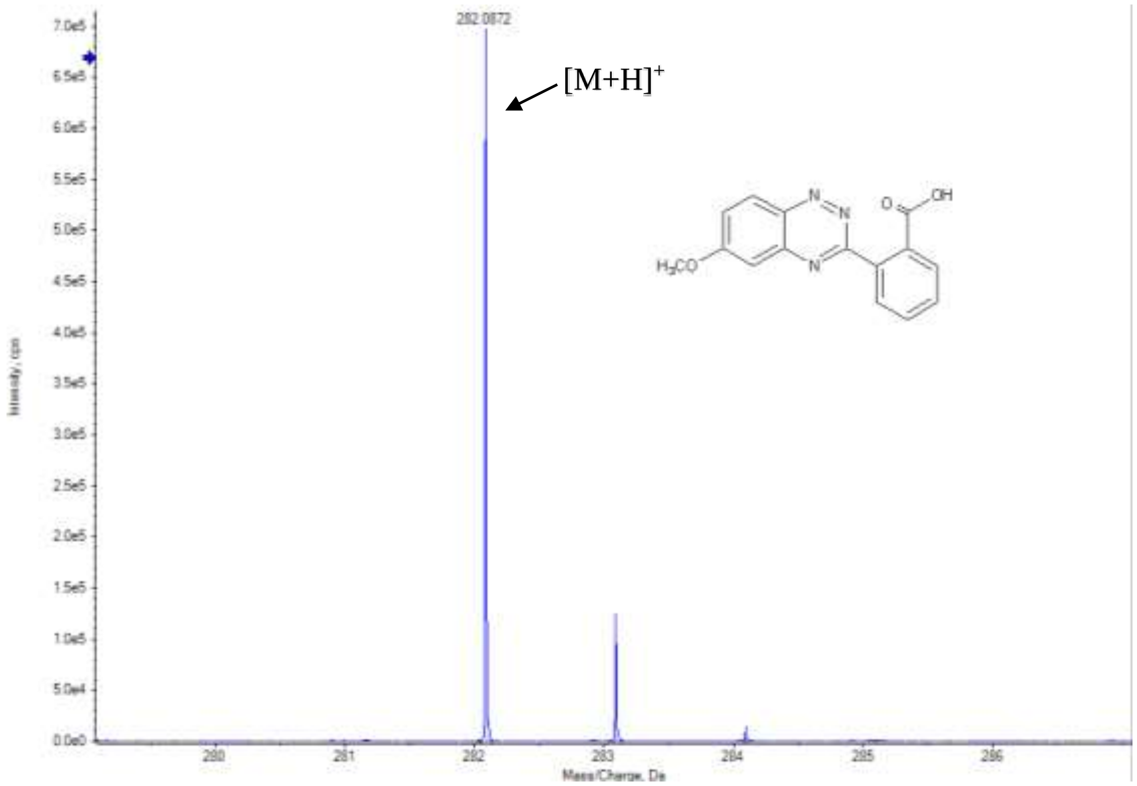

HRMS of **17e** (ESI-Orbitrap) in methanol

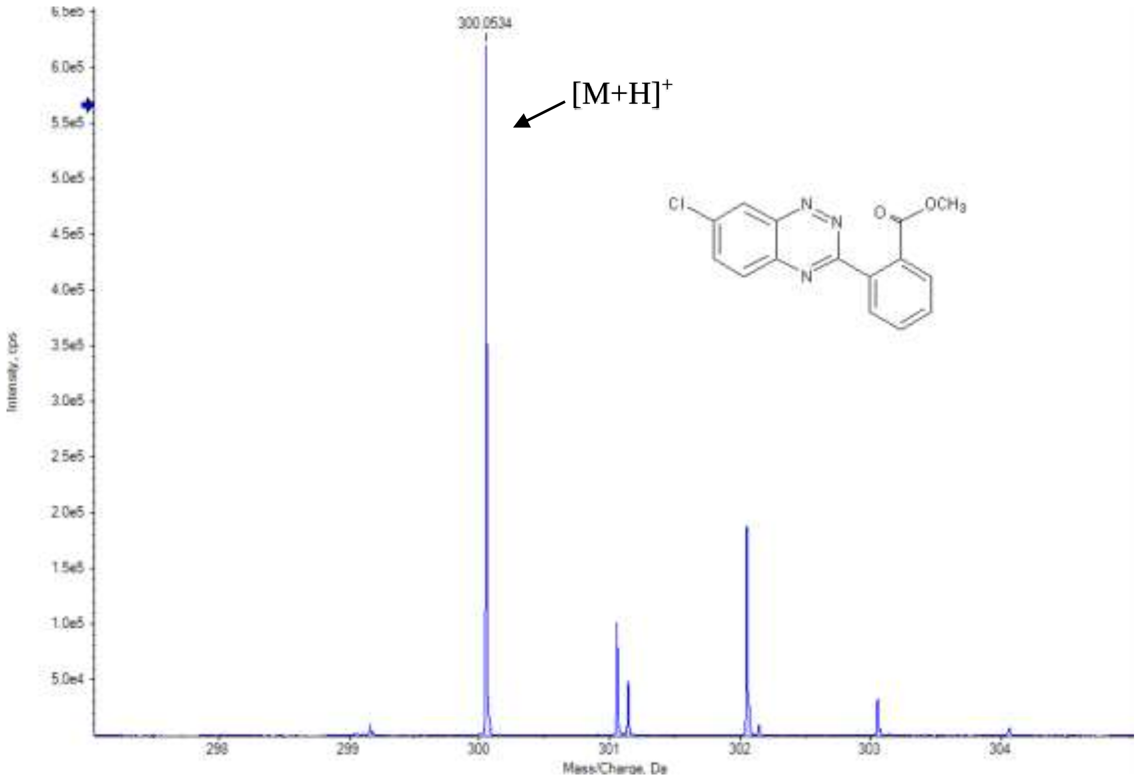

HRMS of **16f** (ESI-Orbitrap) in methanol

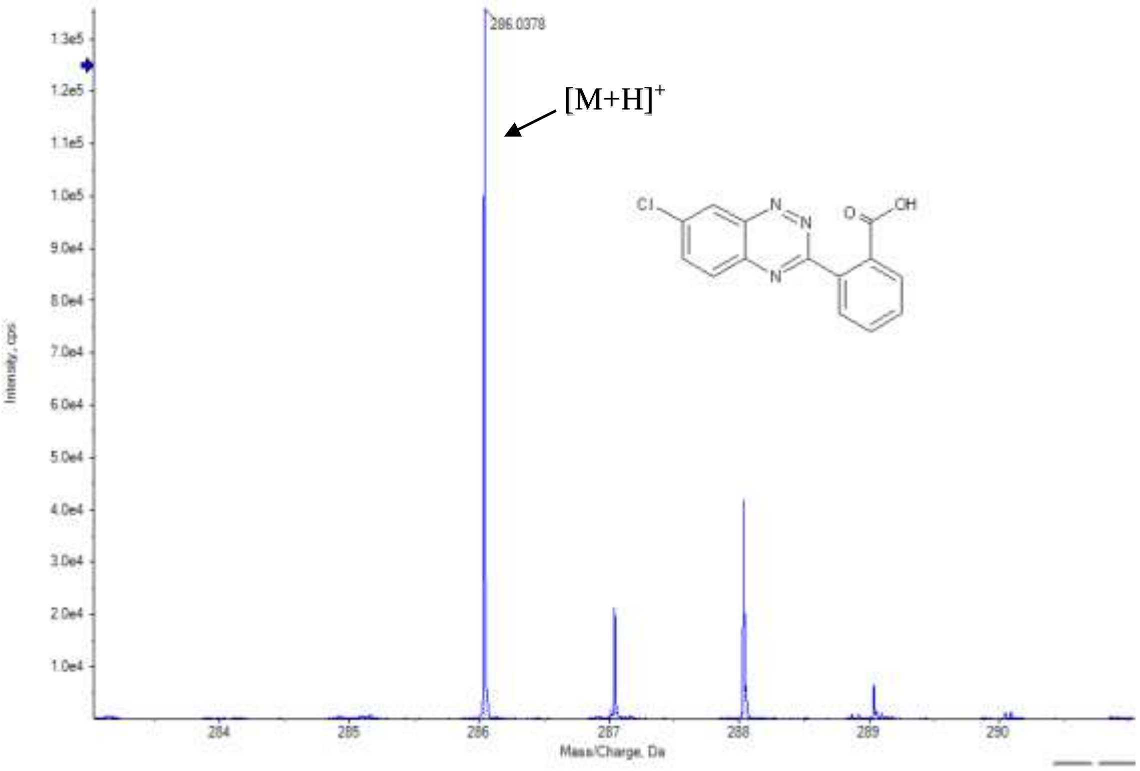

HRMS of **17f** (ESI-Orbitrap) in methanol

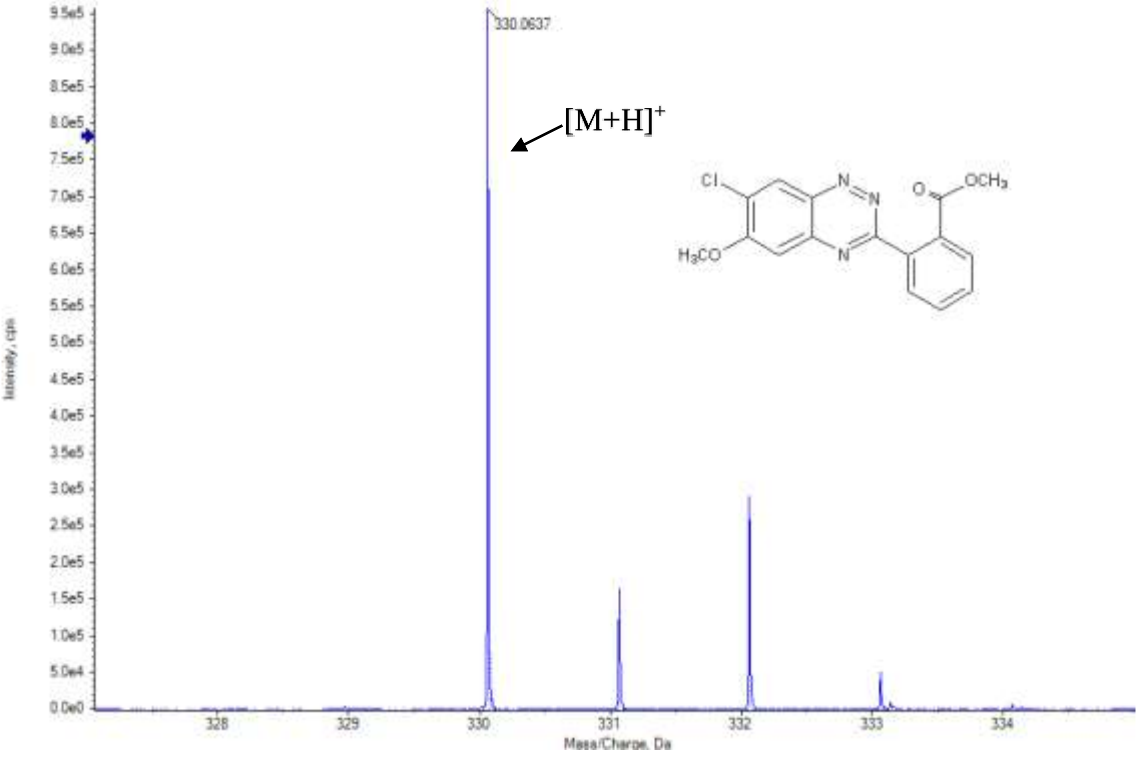

HRMS of **16g** (ESI-Orbitrap) in methanol

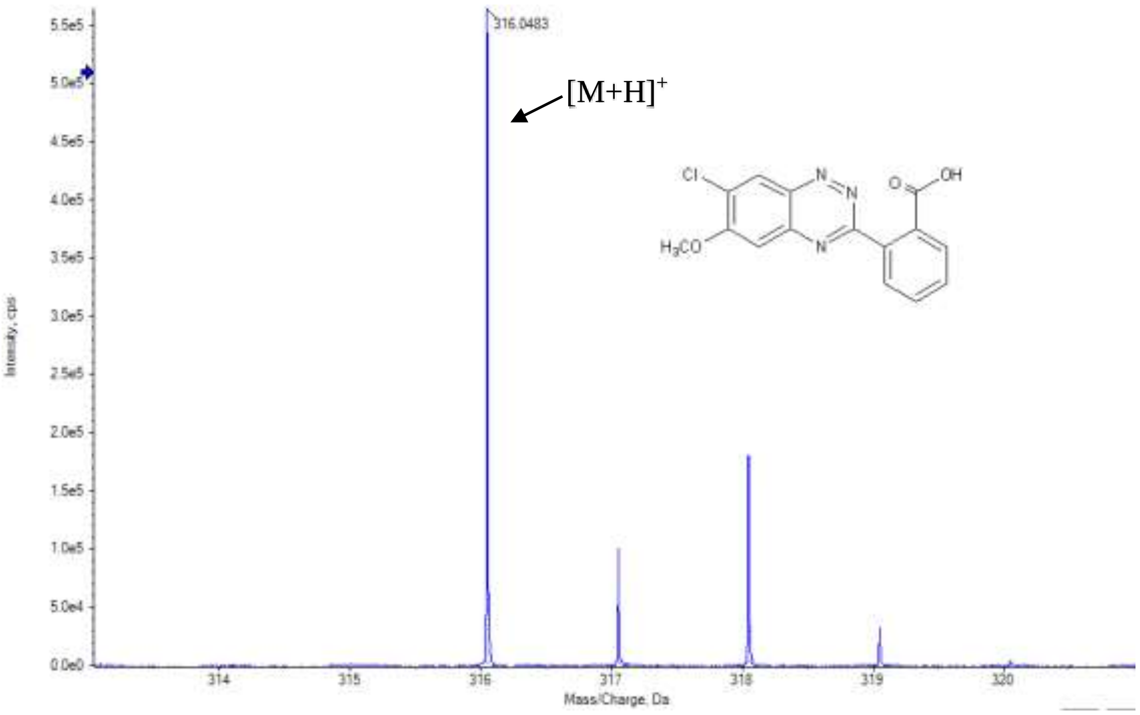

HRMS of **17g** (ESI-Orbitrap) in methanol

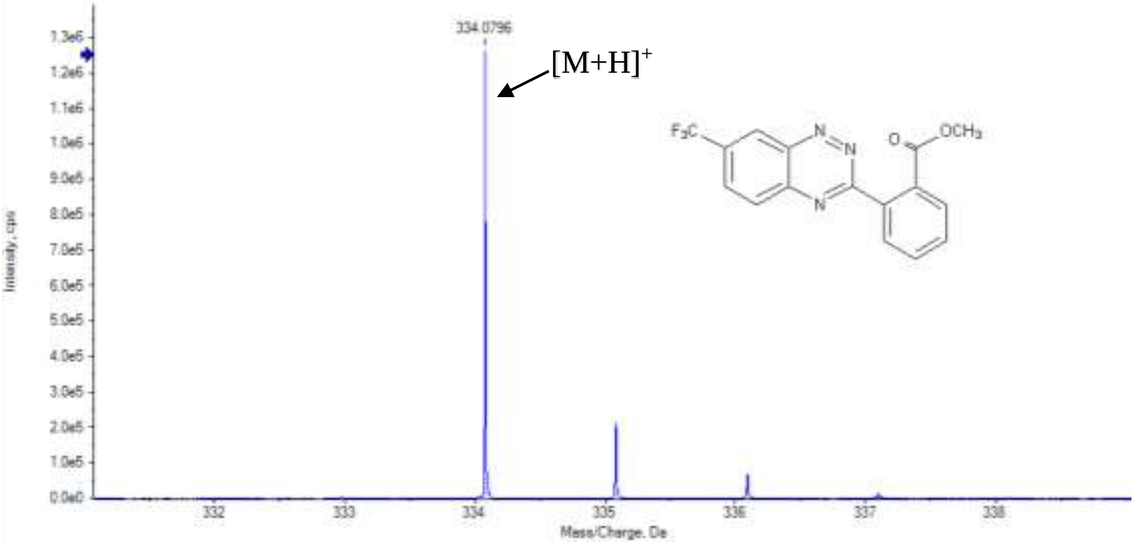

HRMS of **16h** (ESI-Orbitrap) in methanol

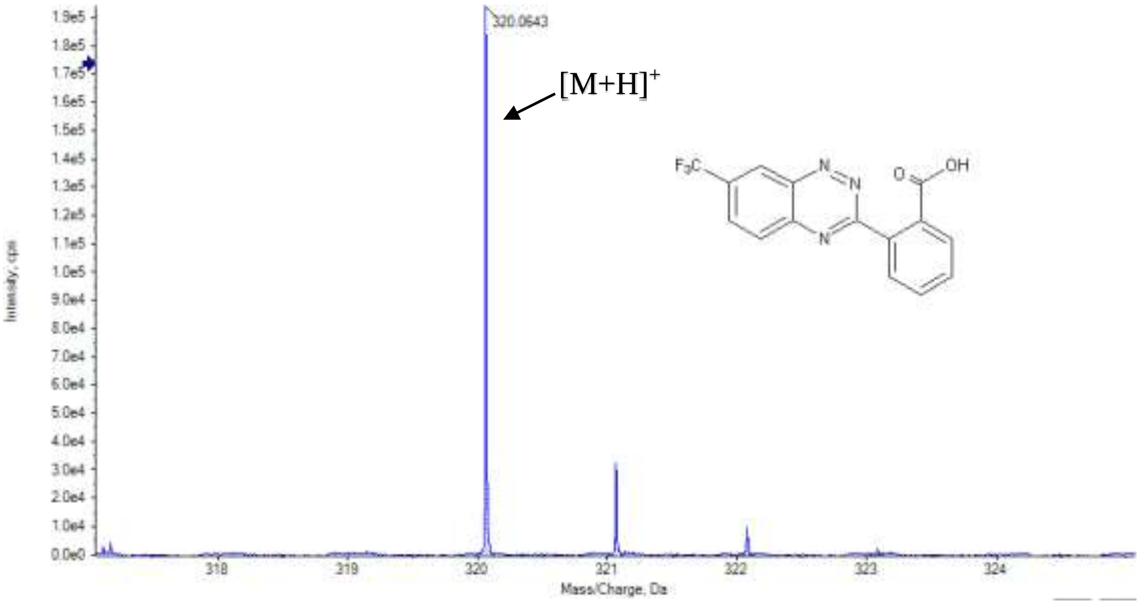

HRMS of **17h** (ESI-Orbitrap) in methanol

APPENDIX D: X-RAY CRYSTALLOGRAPHY OF 16D:

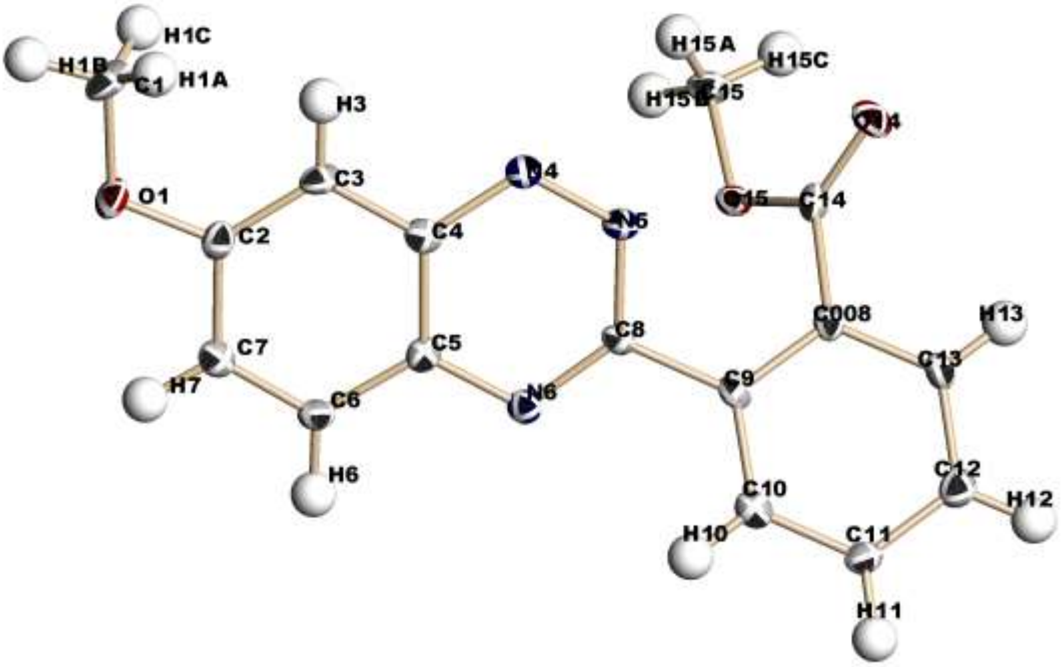

Supplement: Supplementary file 1 — ao2c03045_si_001.pdf [file ao2c03045_si_001.pdf]
